# Local injections of β-NGF accelerates endochondral fracture repair by promoting cartilage to bone conversion

**DOI:** 10.1038/s41598-020-78983-y

**Published:** 2020-12-17

**Authors:** Kevin O. Rivera, Fabrizio Russo, Ryan M. Boileau, Ryan E. Tomlinson, Theodore Miclau, Ralph S. Marcucio, Tejal A. Desai, Chelsea S. Bahney

**Affiliations:** 1 Graduate Program in Oral and Craniofacial Sciences, School of Dentistry, University of California, San Francisco (UCSF), San Francisco, CA USA; 2 Department of Orthopaedic Surgery, Orthopaedic Trauma Institute, University of California, San Francisco (UCSF), San Francisco, CA USA; 3 Department of Bioengineering and Therapeutic Sciences, University of California, San Francisco (UCSF), San Francisco, CA USA; 4 Department of Orthopaedics and Trauma Surgery, Campus Bio-Medico University, Rome, Italy; 5 The Eli and Edythe Broad Center of Regeneration Medicine and Stem Cell Research, Center for Reproductive Sciences, University of California, San Francisco (UCSF), San Francisco, CA USA; 6 Department of Urology, University of California, San Francisco (UCSF), San Francisco, CA USA; 7 Department of Orthopaedic Surgery, Thomas Jefferson University, Philadelphia, PA USA; 8 The Steadman Philippon Research Institute (SPRI), 181 W Meadows Drive, Suite 1000, Vail, CO 81657 USA

**Keywords:** Bone, Cartilage, Musculoskeletal models, Recombinant protein therapy, Bone, Preclinical research

## Abstract

There are currently no pharmacological approaches in fracture healing designed to therapeutically stimulate endochondral ossification. In this study, we test nerve growth factor (NGF) as an understudied therapeutic for fracture repair. We first characterized endogenous expression of *Ngf* and its receptor tropomyosin receptor kinase A *(TrkA)* during tibial fracture repair, finding that they peak during the cartilaginous phase. We then tested two injection regimens and found that local β-NGF injections during the endochondral/cartilaginous phase promoted osteogenic marker expression. Gene expression data from β-NGF stimulated cartilage callus explants show a promotion in markers associated with endochondral ossification such as *Ihh*, *Alpl*, and *Sdf-1*. Gene ontology enrichment analysis revealed the promotion of genes associated with Wnt activation, PDGF- and integrin-binding. Subsequent histological analysis confirmed Wnt activation following local β-NGF injections. Finally, we demonstrate functional improvements to bone healing following local β-NGF injections which resulted in a decrease in cartilage and increase of bone volume. Moreover, the newly formed bone contained higher trabecular number, connective density, and bone mineral density. Collectively, we demonstrate β-NGF’s ability to promote endochondral repair in a murine model and uncover mechanisms that will serve to further understand the molecular switches that occur during cartilage to bone transformation.

## Introduction

Worldwide, bone fractures are associated with significant disability and morbidity, while imposing substantial financial burden on injured individuals^1^. While bone has the capacity to fully regenerate, delayed healing or nonunion occurs in approximately 5–10% of cases^2^. However, delayed healing rates increase to almost 50% in patients with vascular damage or high co-morbidity burdens such as diabetes, increased age, smoking, and obesity^3,4^. Current standard of care for malunions is surgical intervention to increase stability or promote healing through the application of bone grafts. There are currently no pharmacological agents approved to accelerate fracture healing or treat nonunions. The development of novel orthobiologics to accelerate fracture repair could significantly improve patient outcomes.

Bone fractures heal primarily through endochondral ossification (EO), a process by which an avascular, aneural cartilage intermediate transforms into vascularized and innervated bone. Endochondral fracture repair is a dynamic regenerative process that proceeds through four overlapping phases^5–8^. First, following the fracture, a hematoma forms to stop the bleeding, contain debris, and activate a pro-inflammatory response that initiates repair within the first 3–5 days in a murine fracture model^9,10^. During this pro-inflammatory phase, osteoprogenitor cells along the bone surfaces undergo direct osteogenic differentiation (intramembranous ossification) to form new bone along the existing bone adjacent to the fracture site. Later, in the fracture gap, periosteal progenitor cells migrate across the fibrin matrix of the hematoma to differentiate into chondrocytes and generate a provisional cartilaginous matrix starting around 5 days post-fracture^11,12^. Chondrocytes then mature through hypertrophy to promote vascular invasion, mineralization, and transformation into the osteoblasts that form the new bone (7–21 days)^13–15^. The newly-formed trabecular bone continues remodeling in the subsequent weeks into a cortical bone indistinguishable from native bone in form and function^6,7^.

While the molecular pathways that regulate chondrogenesis and hypertrophy are well described^16,17^, the mechanisms regulating conversion of cartilage to bone are not completely understood. Initial chondrogenic differentiation is established through expression of transforming growth factor β (TGFβ), which stabilizes the transcription factor Sox9 required for cartilage formation^18^. Expression of Sox9 is critical for preventing osteogenic specification by suppressing Runx2 and canonical Wnt signaling^19^. The process of chondrocyte hypertrophy is pivotal in the endochondral conversion of cartilage to bone and is a process tightly regulated through a complex negative feedback loop between Indian hedgehog (Ihh) and parathyroid hormone related protein (PTHrP)^20^. Ihh is a key regulator of chondrocyte hypertrophy and bone formation^21,22^ with expression of Ihh observed in both hypertrophic chondrocytes and osteoblasts at the chondro-osseous transition zone (TZ) of fracture calluses^23,24^. Current evidence suggests that subsequent transformation of these hypertrophic chondrocytes into osteoblasts is regulated by the loss of Sox9 expression and activation of canonical Wnt signaling^25–29^.

Despite the importance of endochondral ossification to successful fracture repair, therapeutic approaches to bone regeneration have traditionally focused on promoting intramembranous ossification through the use of bone morphogenetic proteins (BMPs), which forms bone through direct osteoblast differentiation of osteochondroprogenitors^30^. BMP-2 is the only osteoinductive growth factor with FDA approval for treatment of problematic fractures with a very narrow indication window associated with surgical implantation within a carrier scaffold^7,31^. However, clinical application of BMP-2 in fracture healing has become very limited due to uncertain efficacy, high cost, and a growing profile of side effects that has forced the FDA to issue a warning of potential serious complications associated with BMP-2^32^. As such, there remains an unmet clinical need for alternative biologics that stimulate fracture healing. Importantly, we believe there is an opportunity to develop novel therapeutic approaches to fracture repair that builds on the molecular and cellular foundations of endochondral ossification.

While it has long been understood that bone is a highly innervated organ system^33,34^ the functional role of innervation in bone development, homeostasis and fracture repair is complex and evolving. Nerve growth factor (NGF) was first discovered in the early 1950s and following decades of research it is now established for a role in regulating differentiation, growth, survival and plasticity of cholinergic neurons in the central and peripheral systems^35,36^. NGF exerts its trophic function primarily by binding to the high affinity tropomyosin receptor kinase A (TrkA). During development, the NGF-TrkA pathway is essential for load-induced bone formation in mice and exogenous NGF delivery was shown to stimulate angiogenesis and activate Wnt/β-catenin signaling to promote osteogenic lineage progression^37,38^.

Recently, NGF-TrkA signaling was shown to be acutely upregulated following stress fracture, and required for triggering reinnervation, vascularization, and osteoblastic activity during repair^39^. Stress fractures, tiny cracks in the bone caused by repetitive stress over time, are unique in that they heal through intramembranous ossification, rather than through endochondral ossification described above. In this study, we aimed to understand endogenous signaling patterns of NGF-TrkA during endochondral ossification and test whether NGF could be used therapeutically to promote healing. We hypothesized that local injections of NGF, timed to endogenous expression patterns, would promote endochondral fracture repair. In testing our hypothesis, this study is the first to identify an array of molecular switches that NGF acts upon in hypertrophic cartilage. Importantly, our data suggest NGF acts mechanistically to promote cartilage to bone conversion thus improving fracture repair.

## Results

### NGF and TRKA are expressed at the chondro-osseous transition zone during endogenous endochondral fracture repair

**Figure 1:**
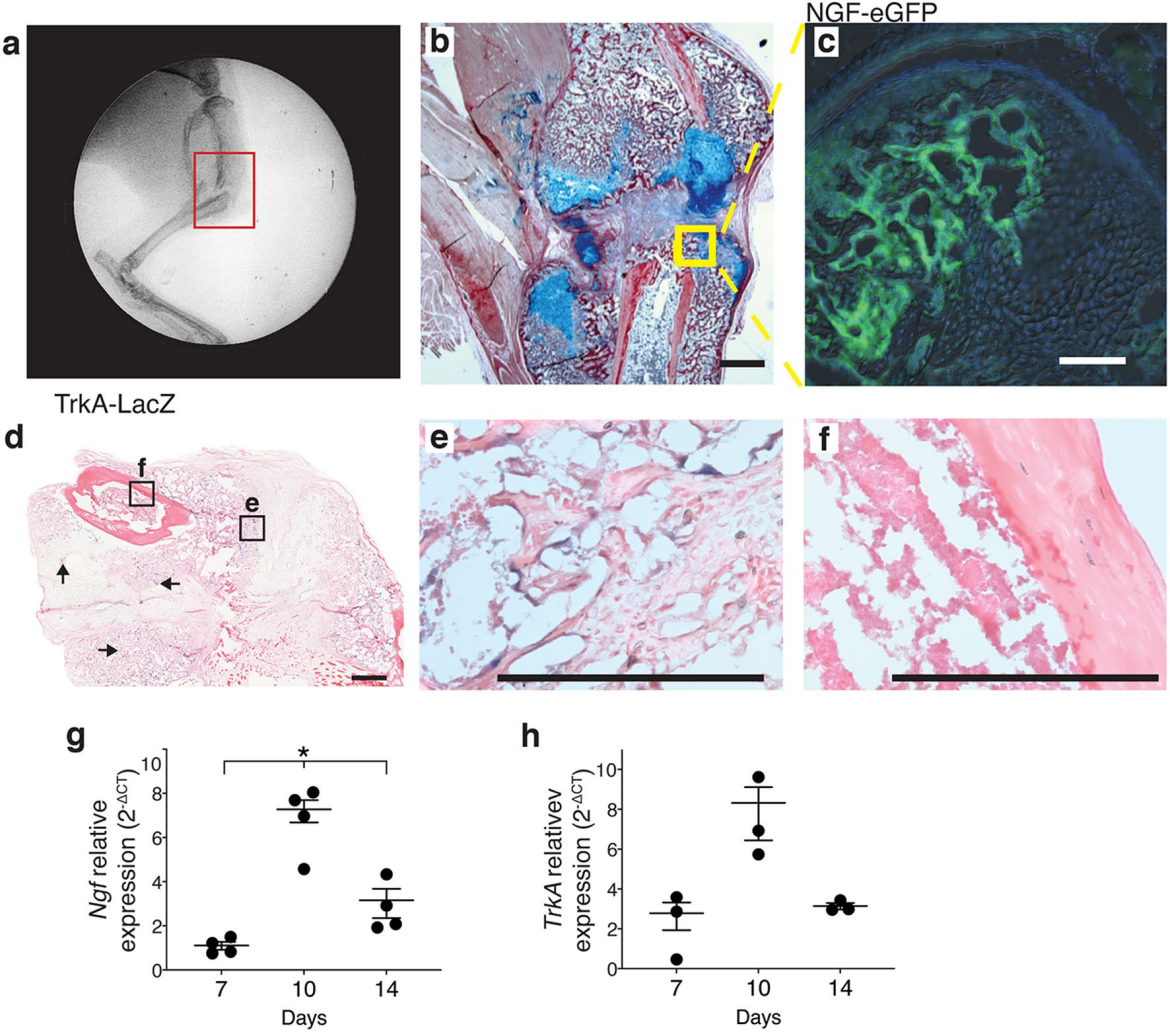
Endogenous expression of Nerve growth factor (NGF) and its receptor Tropomyosin receptor kinase A (TRKA) within fracture callus during endochondral repair. (**a**) A gross fluoroscope image of the entire tibia with the red frame indicating the mid diaphyseal, unstabilized bone fracture. (**b**) Representative image of HBQ stained section of tibia fracture callus 14 days post-fracture (p.f.), (n = 4). Scale bar: 1 mm (**c**) Fluorescence image NGF-eGFP with DAPI of chondro-osseous transition zone (TZ) 14 days p.f. (n = 4). Scale bar: 200 μm. (**d**) Brightfield image of X-GAL stained callus 14 days p.f. (n = 4). Arrows indicate additional areas of LACZ + cells within callus. Scale bar: 500 μm (**e**) Higher magnification image of TZ within fracture callus. (**f**) Higher magnification image of cortical bone shows no staining. (**e**,**f**) Scale bars: 200 μm (**g**) Relative expression (2^-ΔCT^) normalized to *Gapdh* of *Ngf* and (**h**) *TrkA* harvested from fracture callus at 7, 10, and 14 days p.f. Error bars represent SEM. *p < 0.05; determined by one-way ANOVA with Tukey’s multiple comparison test.

We first sought to determine the spatiotemporal parameters of endogenous NGF and TrkA expression during endochondral fracture repair in a murine model of long bone healing. Tibia fractures were created using a three-point bending device to create closed, mid-shaft fractures in the right tibia of adult wild type mice (Fig. 1a). As demonstrated previously^14,15^, these non-stabilized fractures generate a robust cartilage callus, as visualized by Hall and Brunt Quadruple (HBQ)-stained sections (cartilage = blue, bone = red) of tibiae harvested 14 days post-fracture, (Fig. 1b). By utilizing NGF-eGFP reporter mice, we were able to visualize the expression domain of NGF within the chondro-osseous transition zone (TZ) of the fracture callus via fluorescence microscopy (Fig. 1c). TrkA expression appeared in fewer cells but could also be found within cells at this transition zone utilizing TrkA-LacZ reporter mice (Fig. 1d–f).

After establishing the spatial expression patterns of NGF and TrkA at the TZ using histology, we aimed to define the temporal expression patterns of NGF and TrkA using gene expression. Fracture calluses were isolated 7, 10, and 14 days following fracture, mRNA isolated using TRIzol and RT-PCR used to quantify expression of *Ngf* and *TrkA*. Our data show similar temporal expression patterns of *Ngf* and *TrkA* with a peak 10 days post fracture (Fig. 1g,h).

### Endochondral delivery of β-NGF is more osteogenic than early in fracture repair

**Figure 2:**
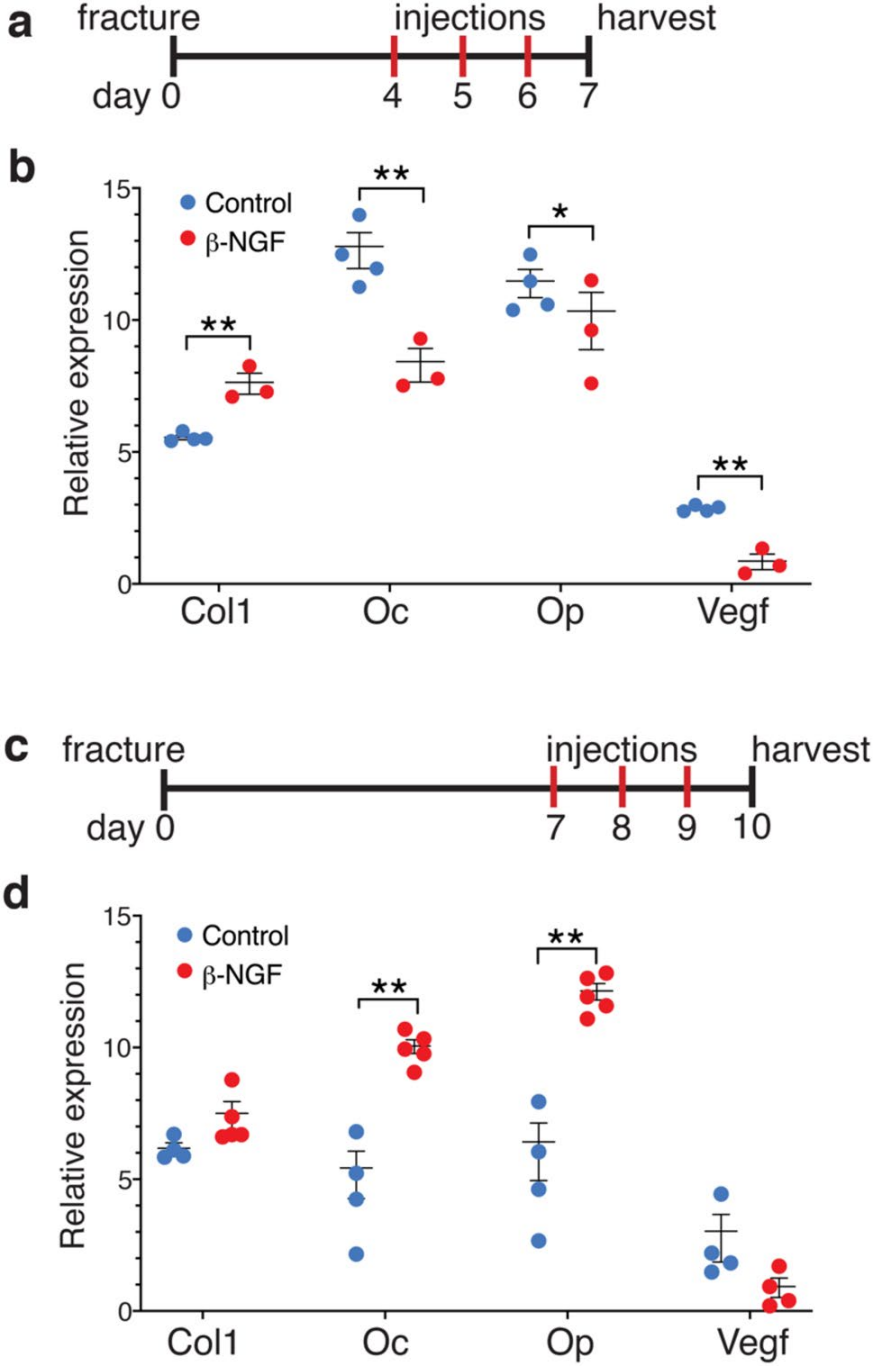
Local β-NGF injections during hypertrophic cartilage phase promotes osteogenic marker expression. (**a**) Timeline schematic of fracture and three daily injections 0.5 μg β-NGF vs control (media injected) starting at 4 days post-fracture. (**b**) Expression levels of selected osteogenic and angiogenic markers from whole-callus tissue harvested 24 h after final injection. (**c**) Timeline of fracture and three daily injections 0.5 μg β-NGF vs control) starting at 7 days post-fracture. (**d**) Expression levels of osteogenic and angiogenic markers from whole-callus tissue harvested 24 h after final injection. All expression levels are relative to *Gapdh*; calculated by 2^-ΔCT^. Error bars represent SEM. *p < 0.05, **p < 0.01; determined by 2-tailed t test.

We next aimed to test the therapeutic efficacy of exogenous β-NGF in long bone fracture healing. When developing novel therapies for fracture healing the majority of drugs are given immediately after fracture by default. However, based on the endogenous spatiotemporal expression patterns of NGF-TrkA correlating with the conversion of cartilage to bone, we wanted to understand if matching therapeutic delivery to the timing of this endogenous expression pattern would be more efficacious. Therefore, we tested two different time points of β-NGF injections: early, during the pro-inflammatory and intramembranous phase of repair (day 4–6, Fig. 2a), or later, during the endochondral phase of cartilage maturation (day 7–9, Fig. 2c). Local delivery was performed on isoflurane anesthetized animals by injecting 0.5 μg β-NGF, or basal media as a control. Early β-NGF injections, resulted in significantly increased relative expression of *collagen 1* (*Col1*) (Fig. 2b). However, there were significant decreases in osteogenic markers *osteocalcin* (*Oc*) and *osteopontin* (*Op*); and the pro-angiogenic *vascular endothelial growth factor* (*Vegf*) (Fig. 2b). Interestingly, later β-NGF injections, robustly stimulated expression of osteogenic markers *Oc* and *Op* (Fig. 2d). We observed non-significant changes in mRNA expression of *Col1* (p = 0.06) and *Vegf* (p = 0.06) following β-NGF injections on the endochondral regimen (Fig. 2d).

### β-NGF stimulation of fracture-callus derived cartilage explants promotes programs associated with endochondral ossification

**Figure 3:**
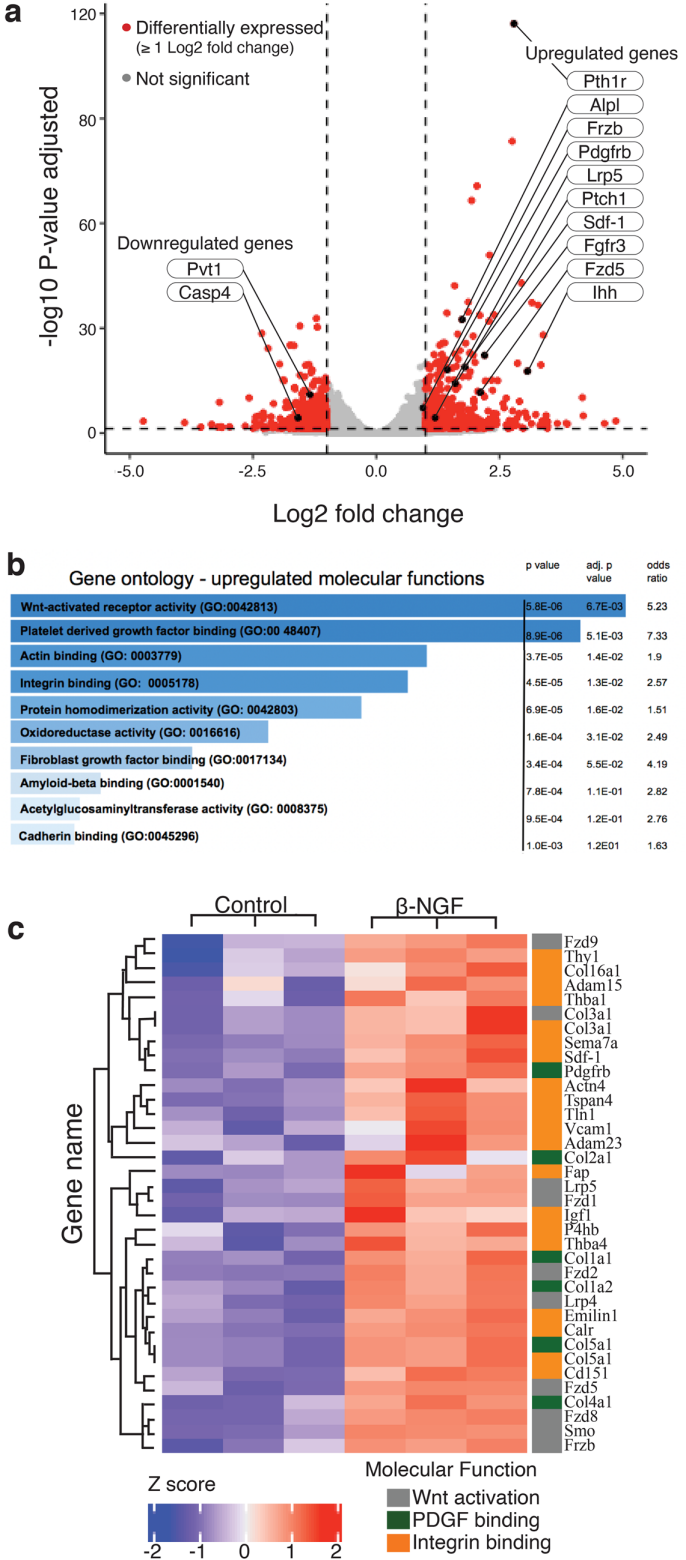
Recombinant human β-NGF (β-NGF) promotes gene expression profile for endochondral bone formation. (**a**) Volcano plot of differentially expressed genes in hypertrophic cartilage stimulated with β-NGF. Threshold set to ≥ 1 log2 fold change (equal to ≥ twofold change), endochondral ossification-associated markers are denoted (n = 3). (**b**) Upregulated molecular function categories generated by EnrichR (maayanlab.cloud/Enrichr/), gene ontology terms are sorted by p values with corresponding adjusted p value and odds ratio (**c**) Heatmap depicting relative expression of genes associated with Wnt activation, PDGF binding, and integrin binding. p < 0.05, Benjamini–Hochberg method. Panel a was generated by the R package ggplot2 (version 3.2.1)^84^. Panel c was generated by Complexheatmap (version 2.0) on Bioconductor (Bioconductor.org).

The endogenous spatiotemporal expression patterns of NGF-TrkA in the TZ and enhanced osteogenic response of cartilage to β-NGF suggested to us that hypertrophic cartilage could be responsive to NGF. To test this, we isolated the cartilage from day 7 fracture calluses, as done previously^14,15^. Explants were cultured to hypertrophy in vitro and treated with or without 0.5 μg/mL recombinant human β-NGF, the biologically active form of NGF^40^, for 24 h followed by RNA-sequencing (RNAseq). Similar to the in vivo study we found that the osteogenic marker *Oc* was significantly upregulated in the cartilage explant study (p = 1.88E−24, Supplemental Table 1). Additional analysis revealed a number of other significantly upregulated genes established to play a role in endochondral ossification, such as, *Indian hedgehog (Ihh), alkaline phosphatase (Alpl), parathyroid hormone 1 receptor (Pth1r),* Wnt receptors *(Lrp5, Frzd5)* and angiogenic receptors *(Pdgfrb)* (Fig. 3a). Of the downregulated genes, the two of most interest were *plasmacytoma variant translocation 1* (*Pvt1*) and *caspase 4* (*Casp4*) (Fig. 3a), both known to modulate apoptosis. A complete summary of differentially expressed genes is provided in Supplemental Table 2.

Subsequent functional enrichment analysis using EnrichR showed multiple categories of molecular functions that were associated with endochondral ossification, fracture repair, and tissue remodeling. The three most significantly upregulated molecular function categories were: Wnt activation (p = 0.0067), Platelet-derived growth factor (PDGF) binding (p = 0.0051), and integrin binding (p = 0.013) (Fig. 3b). With additional enrichment analysis, we then created a heat cluster map of differentially expressed genes according to these molecular function categories (Fig. 3c).

**Figure 4:**
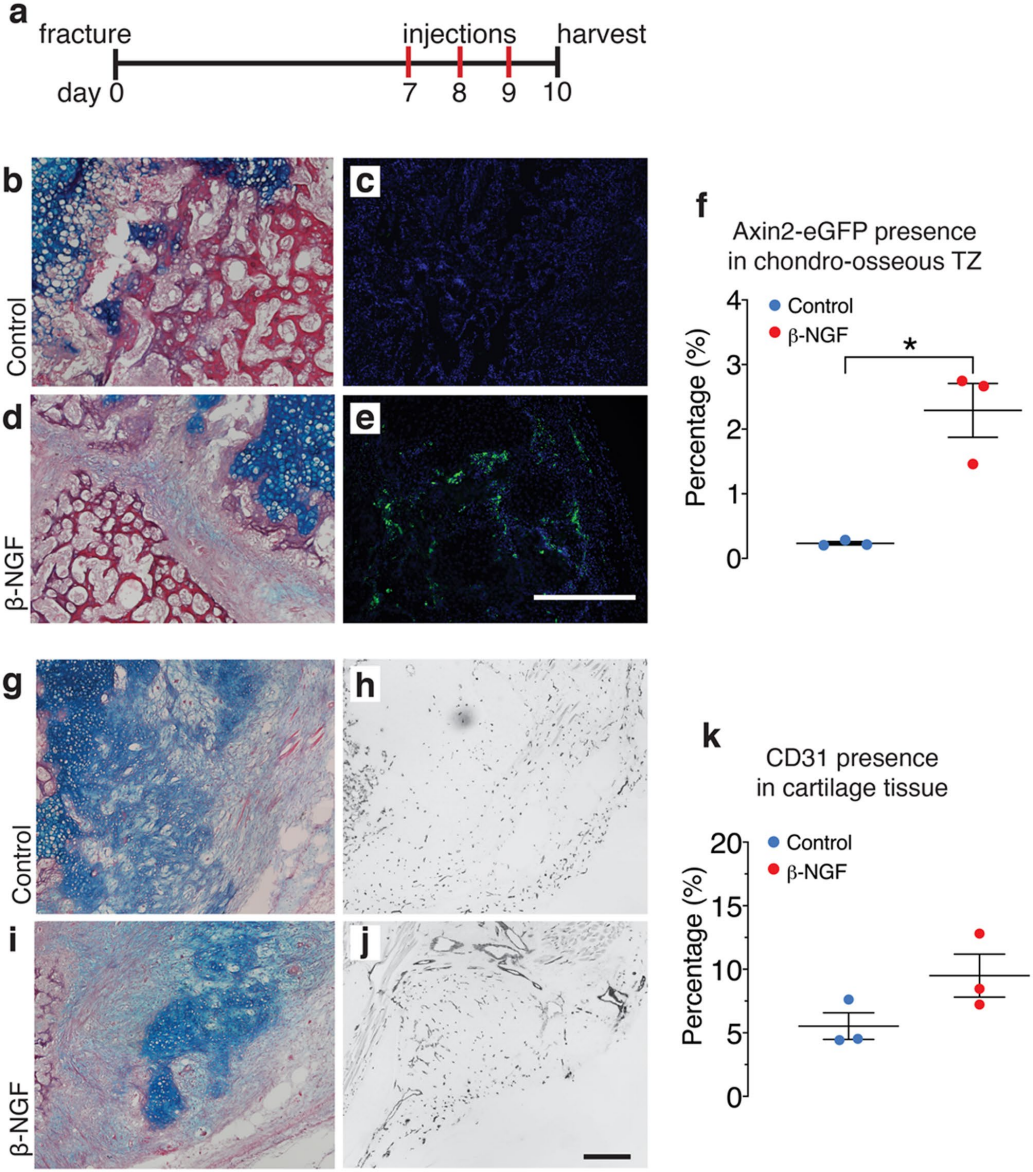
Local injections of β-NGF induce Wnt activation in the TZ and nominal increase of endothelial cell infiltration of cartilage callous. (**a**) Timeline schematic of fracture and subsequent daily injections of β-NGF. (**b**) Representative image of HBQ stained section of the chondro-osseous transition zone (TZ) from control group (media injections) with (**c**) a corresponding fluorescent DAPI-stained image of adjacent slide**.** (**d**) Image of HBQ stained TZ from β-NGF treated mice with corresponding (**e**) fluorescent DAPI stained image of an adjacent slide (**f**) Quantification of Axin2-eGFP presence within TZ of fracture callus as percentage (%) of area. Images of (**g**) HBQ stained section of cartilage tissue within fracture callus from control group and corresponding image (**h**) of Anti-CD31 Diaminobenzidine (DAB) stained section. (**i**) HBQ stained section from β-NGF treated group and corresponding (**j**) CD31-DAB stained section. (**k**) Quantification of DAB stain within cartilaginous tissue as percentage (%) of area. All scale bars = 500 μm. Error bars represent SEM. **p < 0.01; determined by 2-tailed t test.

To confirm our RNAseq data suggesting Wnt was the most significantly upregulated molecular function following β-NGF treatment of cartilage ex vivo (Fig. 3b,c), we utilized a murine Axin2-eGFP reporter model to compare Wnt expression in vivo in mice treated with β-NGF to those without. Tibia fractures were made in the Axin2-eGFP mouse as described previously and β-NGF was injected days 7–9 post-fracture (Fig. 4a). Visually, our control mice showed no major presence of Axin2-eGFP positive cells in the TZ (Fig. 4b,c). However, there was an induction of Axin2-eGFP in cells at the TZ of β-NGF treated mice (Fig. 4d,e). Quantification by Image-J confirmed the induction of Axin2-eGFP after β-NGF treatment compared with the lack of Axin2-eGFP presence in the control group (Fig. 4f).

Given literature evidence that NGF signaling precedes and coordinates vascularization of bone tissue^38^, we wanted to measure if local β-NGF injections promoted the infiltration of endothelial cells into the cartilage callus. Therefore, angiogenesis was quantified using immunohistochemistry performed to the CD31 endothelial cell marker day 10 post-fracture in wild type mice that received the endochondral delivery of β-NGF. Vascular invasion to the cartilage callus is observed in both the controls (Fig. 4g,h), with slightly more intense staining in the β-NGF group (Fig. 4i,j). Quantification by Image-J indicates only a nominal increase in CD31-positive cells in cartilage tissue of β-NGF treated mice (p = 0.12) (Fig. 4k).

### Local β-NGF injections accelerates endochondral bone formation by 14 days post-fracture

**Figure 5:**
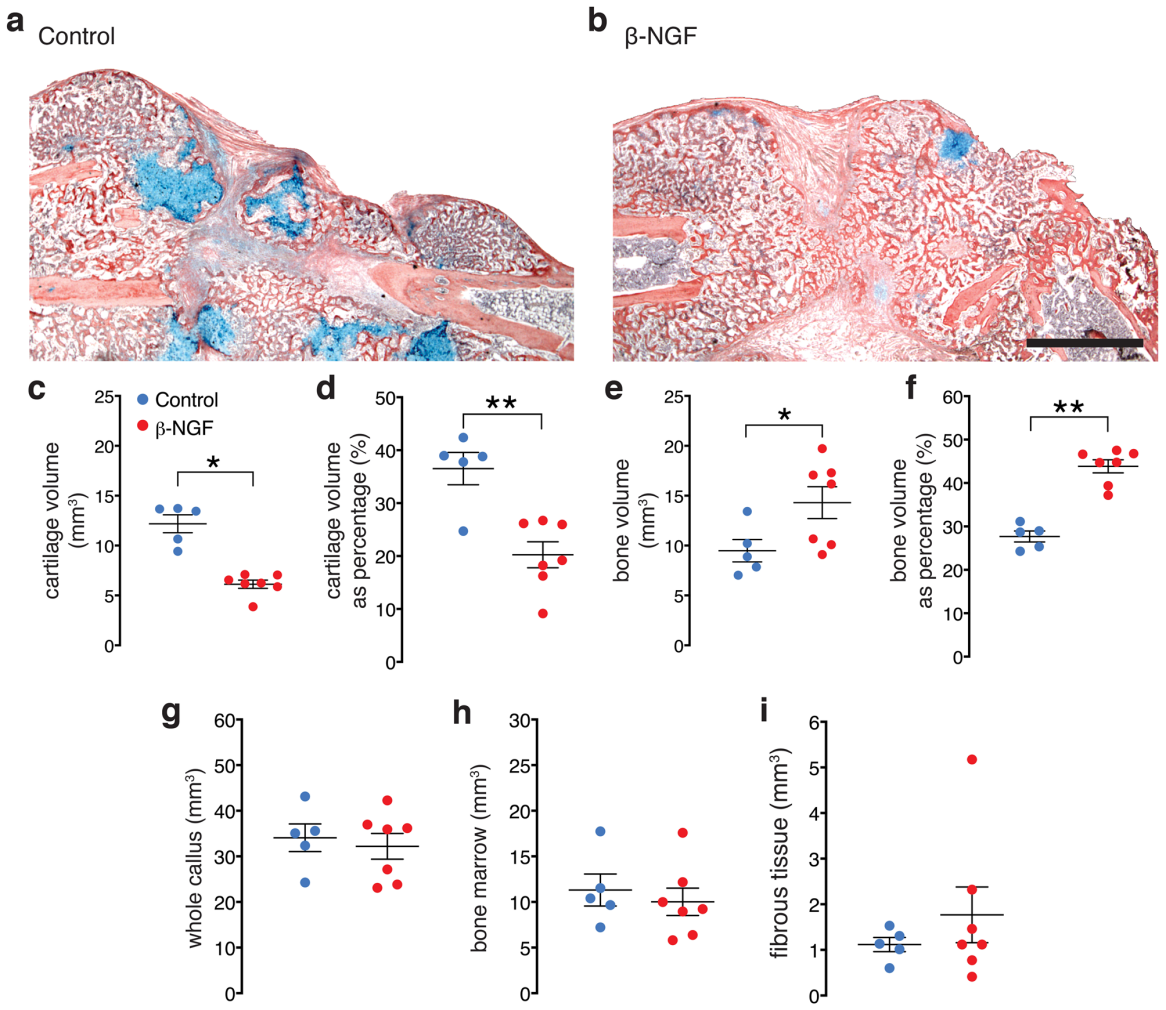
Local injections of β-NGF results in less cartilage and more bone. Representative images of HBQ stained section of fracture callus from (**a**) control group and (**b**) β-NGF group, 14 days post fracture. Scale bar: 500 μm. Quantification of cartilage volume in both treatment groups, shown as (**c**) absolute volume and as (**d**) percent composition of the total callus volume. Quantification of bone volume in both treatment groups shown as (**e**) absolute volume and (**f**) percent composition. Quantification of (**g**) whole-callus volume (**h**) bone marrow and (**i**) fibrous tissue. All measured by stereology. Error bars represent SEM. *p < 0.05; **p < 0.01 determined by 2-tailed t test.

We next tested functional outcomes of fracture healing with the endochondral delivery of therapeutic β-NGF using histomorphometric and quantitative μCT analysis on treated and control tibias 14 days post fracture. Histology clearly shows the increased formation of trabecular bone (red) and decreased cartilage (blue) in fractures receiving β-NGF relative to control (Fig. 5a,b). Quantification of the cartilage tissue showed an almost 50% decrease in absolute volume (Fig. 5c) and percent composition (cartilage volume/total volume) within the callus of β-NGF treated mice (Fig. 5d). Conversely, quantification of trabecular bone confirmed a similar increase in absolute bone volume (Fig. 5e) and composition (bone volume/total volume) of the callus after β-NGF treatment (Fig. 5f). Importantly, there was no difference in volume of the callus as a whole between controls and β-NGF treated mice (Fig. 5g). There were also no differences in volume of bone marrow (p = 0.59) (Fig. 5h) or fibrous tissue (p = 0.40) between groups (Fig. 5i) suggesting that the conversion of cartilage to bone was accelerated in the experimental group.

**Figure 6:**
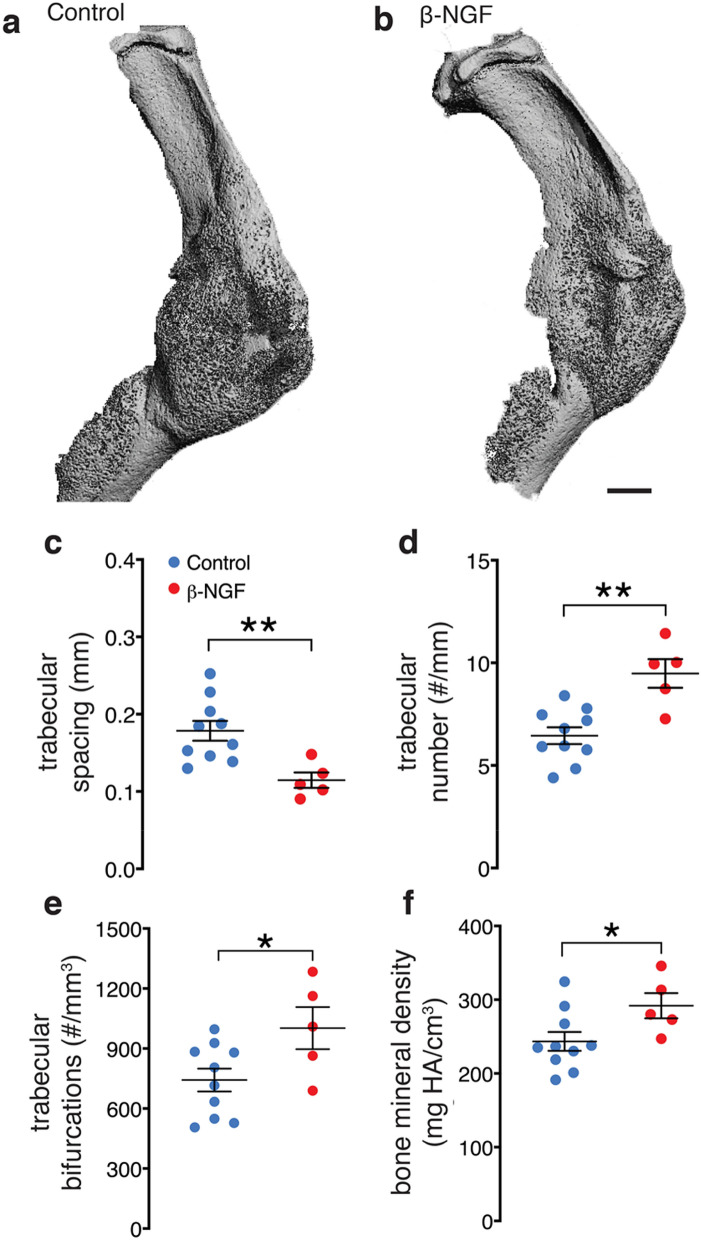
Local injections of β-NGF results in highly connected trabecular bone. μCT images of tibias from (**a**) control and (**b**) β-NGF treated mice, 14 days post fracture. Scale bar = 1 mm. Quantification of (**c**) trabecular spacing (**d**) trabecular number (**e**) trabecular connective density and (**f**) bone mineral density. Error bars represent SEM. *p < 0.05; **p < 0.01 determined by 2-tailed t test.

μCT analysis was performed in parallel to histomorphometry on control and β-NGF treated mice 14 days post fracture. Gross examination of μCT images provide no obvious differences between treatment groups (Figs. 6a,b). However, quantitative assessment of structural indices showed a stark difference in bone architecture. β-NGF treated mice exhibited a 35% decrease in trabecular spacing compared to the controls (Fig. 6c), with trabecular number (Fig. 6d) and trabecular connective density showing dramatic increases of over 40% (Fig. 6e). Bone mineral density measurements also significantly increased, ~ 20%, in the fracture callus of β-NGF treated mice (Fig. 6f). Taken together, μCT data depict highly connected and structurally superior bone architecture in β-NGF treated mice indicative of a later stage of endochondral repair.

## Discussion

There is an unmet clinical need for novel therapeutic approaches to accelerate fracture repair as there currently are no pharmacologic agents approved for this indication. Bone morphogenetic protein-2 (BMP-2) is the only US FDA approved biologic in fracture healing with a very narrow indication window: surgical implantation into acute open tibial shaft fractures stabilized with an intramedullary nail and treated within 14 days of the initial injury. Clinical use of BMP in fracture healing has significantly decreased and is now typically reserved to the most problematic fractures due to the high cost, limited evidence of clinical efficacy, and risk for severe off-target effects^41–44^. A number of new pharmacologic agents have been explored for application in fracture healing, but almost all have focused on promoting intramembranous bone repair by stimulating osteogenesis^7^.

This study aimed to test the therapeutic efficacy of locally administered β-NGF in accelerating long bone fracture repair. NGF is an understudied orthobiologic for bone regeneration. In the limited number of studies that have utilized recombinant NGF for promoting fracture repair, NGF was administered at very high doses, immediately following fractures and the osteogenic potential was attributed to increased neuroactivity with no comprehensive structural analysis of the newly-formed bone^45,46^. More recently, an elegant study utilized genetic models to show NGF-TrkA is required in stress fracture repair, stimulating intramembranous healing by promoting peripheral nerve innervation, angiogenesis, and osteogenesis^39^.

Uniquely, in our study we demonstrate that β-NGF was most efficacious in promoting long bone fracture healing when the drug was administered during the cartilaginous phase of repair, days 7 to 9 post fracture, reflecting the upregulation in endogenous *Ngf* and *TrkA* gene expression that we observed. Histological data utilizing NGF-eGFP and TrkA-LacZ reporter mice provide the first genetic labeling of the expression pattern within the callus of tibia fractures. Most notably, we find NGF and TrkA are localized predominantly at the chondro-osseous transition zone, where cartilage undergoes hypertrophy and transforms to bone adjacent to the invading vasculature^6,15^. Our genetic models of endogenous NGF and TrkA localization support previous studies observing peak expression of neurotrophins and their receptors during the hypertrophic cartilage phase of repair^47–49^. While our mRNA expression data show a peak at day 10, possible delays in mRNA-to-protein synthesis were considered when harvesting samples at day 14 for histological analysis. Histological visualization of NGF and TrkA expression at this timepoint demonstrate a broad and robust presence in the chondro-osseus transition zone of tibial fracture calluses.

Based on this endogenous spatiotemporal map of NGF-TrkA expression during long bone fracture healing, we hypothesized that hypertrophic cartilage was responsive to NGF stimulation. We tested this ex vivo by isolating the cartilaginous portion of day 7 fracture calluses, culturing the explants to hypertrophy, and treating with or without β-NGF. Utilizing RNAseq, we were able to detect over 3,000 differentially expressed genes in β-NGF stimulated cartilage. Of the significantly upregulated genes, many clumped into molecular pathways known to play a role in cartilage maturation, endochondral ossification, and tissue mineralization.

Within the pathways regulating cartilage maturation, those associated with *Indian hedgehog signaling* (*Ihh*) were strongly stimulated. Ihh is a known regulator of chondrocyte differentiation and the expression of Ihh has been observed at the transition zone of fracture calluses^23,24^. Within this cluster *Ptch1*, a receptor for Ihh, and *Pth1r*, the receptor for both parathyroid hormone (Pth) and parathyroid hormone-related peptide (Pthrp), were also significantly upregulated. Ptch1 and Pth/Pthrp are established transcriptional targets of Ihh^23,50^. Ihh signaling was shown to be required for the generation of the osteoblast lineage in endochondral ossification and disruption of the pathway produces abnormalities in bone development^21,22^. Sdf-1 expression, modulated by Pth, is also produced by osteoblasts and is known to influence stem cell homing, tissue repair, and tissue regeneration in various contexts including bone repair^51,52^. Furthermore, one of the earliest reports of a chondrocyte-derived cell associated with trabecular bone was identified by conditional ablation of Ihh signaling in chondrocytes^53^, emphasizing the importance of highly coordinated chondrocyte maturation to effective endochondral ossification. In this study we also found that β-NGF stimulated the expression of *alkaline phosphatase* (*Alpl*) which is associated with chondrocyte hypertrophy and essential for cartilage mineralization and endochondral bone formation^54,55^. *Alpl* is among the first functional genes expressed during tissue calcification and is a commonly screened marker of osteoblast differentiation^56,57^.

The osteogenic transformation of chondrocytes into osteoblasts during bone development and fracture repair is associated with the upregulation of traditional programs regulating osteogenesis and mineralization. In fact, we found that the canonical osteogenic marker *osteocalcin* was strongly upregulated by β-NGF when delivered both in vivo to the fracture site or to the cartilage callus explant. Through gene ontology enrichment analysis, we identified several molecular functions associated with osteogenesis were upregulated, with Wnt activation as the most significantly upregulated following β-NGF treatment. Importantly, we have recently shown that Wnt/β-catenin signaling regulates cell fate decisions in hypertrophic chondrocytes at the transition zone and that Wnt signaling within the cartilage is required for endochondral fracture repair^27^. This corroborates numerous published reports on the requisite of Wnt signaling in endochondral bone formation during development, and specifically for chondrocyte-derived osteoblastogenesis^25,28,29^. NGF signaling has also been shown to modulate Wnt activation in several non-skeletal cell types^58,59^. However, this is the first study in which this relationship has been noted in cartilage after in vitro stimulation with β-NGF. We further confirmed Wnt activation in vivo by histological analysis using Axin2-eGFP mice following local c injections. Taken together these data suggests β-NGF treatment stimulates Wnt-mediated cartilage to bone conversion during endochondral fracture repair. Additional studies are needed to determine whether Wnt activation by NGF is direct or indirect, and if it is Wnt ligand dependent or independent^58^.

Additional gene ontology enrichment analysis resulted in PDGF binding as one of the most upregulated molecular function. In chondrocytes, PDGF stimulates proliferation but prevents endochondral maturation however, PDGF expression has principally been seen in osteoblasts during normal fracture repair^60,61^. Integrin binding activity was also observed to be upregulated in cartilage explants stimulated with β-NGF. Integrin interactions in chondrocytes are known to regulate chondrocyte proliferation and apoptosis however, integrin signaling events in osteoblasts regulate mineralization and has been a therapeutic target for bone regeneration^62,63^. Although we were able to determine that integrin binding as a molecular function was promoted in cartilage explants following β-NGF stimulation, we were unable to determine specific integrin subunits that were engaged. Future work consisting of blocking specific integrin subunits could prove useful in determining NGF-mediated activation of specific integrin subunits.

Of the downregulated genes *Pvt1* and *Casp4* drew our attention given that Pvt1 is known to modulate apoptosis and Casp4 is strongly implicated in an inflammatory form of apoptosis^64,65^. Historically, hypertrophic chondrocytes were considered terminally differentiated and believed to undergo apoptosis during endochondral bone formation. However, our group and others have demonstrated that chondrocytes re-enter the cell cycle and through lineage tracing show that chondrocytes survive and give rise to osteoblasts in the growth plate and bone regenerate^14,15,20,22,66,67^. Therefore, NGF-TrkA signaling in the fracture callus may play a role in promoting cell survival in the hypertrophic chondrocytes.

Our previous studies suggest that mineralization of the cartilage anlage and transformation of chondrocytes to osteoblasts during endochondral fracture healing is mediated by the invading vasculature^17,18^. Interestingly, NGF was found to be critical to promoting angiogenesis and tissue mineralization during long bone development^38,45^. Our RT-qPCR data of whole callus tissue show a decrease of VEGF following the earlier β-NGF injections versus no change in VEGF expression following the later regimen of β-NGF. However, when we measured changes to vascularization in β-NGF treated fractures, we did see an increase in endothelial cell presence within the cartilage callus, albeit nominal. The progression of fracture healing from the inflammatory phase to the cartilaginous phase involves many different cell types that are sensitive to a huge array of paracrine and autocrine factors^6–12^. Because NGF’s receptor is expressed in many cell types and the heterogenous composition of the fracture callus, it’s difficult to directly correlate VEGF expression and endothelial cell presence. Nonetheless, like fracture healing, angiogenesis is a highly complex process and there remains a number of angiogenic factors that could be measured for in future studies^68^. What we found most compelling is that β-NGF stimulation of cartilage explants significantly promoted endochondral ossification-associated pathways in the absence of a vasculature.

Given the molecular pathways modulated by β-NGF stimulation of cartilage explants, it is not surprising that timing of injections proved to be important in dictating the best therapeutic window of β-NGF. We found a stronger osteogenic effect of β-NGF when delivered during the endochondral phase of repair, as opposed to early, during the pro-inflammatory response and intramembranous healing. With endochondral delivery β-NGF, histomorphometric analyses of callus tissue resulted in a reduction in cartilage and increase in bone tissue compared to control. Furthermore, we did not see a change in the total volume of the fracture callus, therefore these data support the hypothesis that β-NGF accelerates cartilage to bone conversion. In addition to histomorphometry, μCT data further illustrates the high connectivity and high mineral density of the newly formed trabeculated bone.

Coincidentally, an increase in NGF and TrkA expression has also been observed in osteoarthritic cartilage^69^. This is an important observation considering many processes observed in osteoarthritis occur in endochondral ossification, such as, cartilage tissue vascularization, innervation, and cartilage matrix degradation^70,71^. The molecular pathways underlying these processes mirror those seen in our data, most notably the upregulation of *Ihh*, *Alpl*, *Ptch1*, and *Pth1r*^72,73^ with a recent study reporting on NGF-mediated upregulation of Ihh signaling and cartilage calcification of articular chondrocytes^74^. Wnt and NGF signaling have been major therapeutic targets to inhibit the progression and pain associated with osteoarthritis^72,75,76^. Our group has previously exploited these parallels by engineering osteoarthritic cartilage to promote endochondral bone repair^77^.

Discovering and validating novel therapeutic targets that stimulate bone regeneration has the potential to significantly improve clinical outcomes in fracture healing. Gene expression analysis, histomorphometry and μCT data collectively demonstrate that β-NGF treatment during the endochondral phase of fracture repair stimulates osteogenesis to produce more bone tissue and that the newly formed bone is more connected and of higher architectural quality. A possible limitation on NGF’s clinical translation lies in its hyperalgesic effects. Yet, diverging from previous studies that administered NGF for 7 days^45,46^, our study was able to narrow NGF’s therapeutic window to 3 days, during the endochondral phase of repair, thus limiting the subjects’ exposure to exogenous NGF and ensuing hyperalgesia. Exogenous NGF’s hyperalgesic effects seems to peak within an hour of administration therefore, this could be further studied by simple co-injection of an analgesic such as lidocaine^78^. Very little work has been done on relevant cell types to optimize the therapeutic dosing of NGF while minimizing hyperalgesia. Preclinical studies in murine models have utilized up to 20 μg of exogenous NGF per day yet, in vivo work has demonstrated that hyperalgesia in mice can be experienced with daily injections of 100 ng^79,80^. Balancing the trophic benefit of NGF therapy and minimizing hyperalgesia in this context will prove to be another key area of research in the future.

Nevertheless, this study lays the foundation for utilizing recombinant β-NGF in endochondral fracture repair by providing comprehensive tissue analysis of tibial fracture calluses after therapeutic administration. Importantly, we add new mechanistic data that increases our understanding of the processes that regulate and promote cartilage to bone conversion during endochondral fracture repair, opening new avenues of mechanistic exploration.

## Materials and methods

### Study approval

#### Tibia fracture model

Approval was obtained from the University of California, San Francisco (UCSF) Institutional Animal Care and Use Committee (IACUC) prior to performing the mouse studies, the methods were carried out in accordance with relevant guidelines and regulations. Briefly, adult (10–16 weeks) male mice were anesthetized via inhalant isoflurane, and closed non-stable fractures were made mid-diaphysis of the tibia via three-point bending fracture device^14^. Fractures were not stabilized as this method promotes robust endochondral repair. After fractures are created, animals were provided with post-operative analgesics (buprenorphine sustained-release). Animals were socially housed and allowed to ambulate freely.

### Mice

Studies involving wildtype mice were conducted on the C57BL/6J strain obtained from Jackson Labs (Stock #000664). NGF-eGFP, which express eGFP under the control of the mouse NGF promoter, were generously donated by the Dr. Ryan Tomlinson at Thomas Jefferson University^81^. TrkA-LacZ mice, which have a LacZ sequence inserted immediately following the ATG in exon 1 of the mouse Ntrk1 gene, were also kindly provided by Dr. Tomlinson and are commercially available from Jackson Labs (Stock #004837)^82^. Axin2-eGFP mice, which express eGFP under the Axin2 promoter/intron 1 sequences, were generously donated by the Dr. Jeremy Reiter at UCSF^83^.

### β-NGF and control injections

Two time points were initially tested to compare osteogenic marker expression within fracture calluses. Injections were administered once daily for 3 days beginning either four days or seven days post-fracture, (Fig. 2c,d). Experimental groups consisted of 0.5 μg of recombinant human β-NGF in 20 μL of basal media versus control injections of basal media-only (DMEM basal media, Gibco cat #A1443001) using a Hamilton syringe guided by fluoroscopy. A dosage of 0.5 μg/day was derived from earlier protocols wherein our dose lies between the 0.1–1.4 μg/day previously used^46,79^.

### mRNA isolation and RT-qPCR

After β-NGF administration, calluses were harvested 24 h following the final injection. After callus dissections, tissue samples were homogenized in Trizol then mRNA was extracted from tissue lysates by use of RNeasy Mini Kit following the manufacturer’s instructions (Qiagen cat# 74104). cDNA was reverse transcribed with Superscript III (Invitrogen cat# 18080), and RT-qPCR was performed using SYBR Green and primers as listed (Table 1). Relative gene expression was calculated by normalizing to *Gapdh* and are shown as 2^-ΔCT^(Fig. 2c,d).

**Table 1:** Primer list.

Primer sequences		
	Forward (5′ to 3′)	Reverse (3′ to 5′)
Gapdh	TGATGACATCAAGAAGGTGGTGAAG	CCTTGGAGGCCATGTAGGCCAT
Ngf	ACAGTGTATTCAGACAGTACTTTTTTGAGA	GAGTTCCAGTGTTTGGAGTCGAT
TrkA	AGAGTGGCCTCCGCTTTGT	CGCATTGGAGGACAGATTCA
Col1	CCCAGAACATCACCTATCAC	TTGGTCACGTTCAGTTGGTC
Oc	CGCTCTGTCTCTCTGACCTC	TCACAAGCAGGGTTAAGCTC
Op	GCACTCCAACTGCCCAAGA	TTTTGGAGCCCTGCTTTCTG
Vegf	CTGTGCAGGCTGCTGTAACG	GTTCCCGAAACCCTGAGGAG

### Histology

Fractured tibiae were fixed in 4% paraformaldehyde (PFA) then decalcified in 19% ethylenediaminetetraacetic acid (EDTA) for 14 days. Mice were processed for paraffin histology through a graded ethanol series and cleared in xylene prior to embedding in paraffin tissue blocks. Serial sections were cut at 8–10 μm for histological analysis. Every 10th slide was stained with standard histological protocols for Hall and Brunt’s Quadruple staining (HBQ) to visualize bone (red) and cartilage (blue) were used. Tissues from NGF-eGFP and Axin2-eGFP reporter strains were embedded in OCT and sectioned using a cryostat. Axin2-eGFP fluorescence was amplified by utilizing antibody conjugated to AlexaFluor488 (see section on IHC protocol). X-Gal staining was performed following an adapted protocol from previous work^37^: samples were fixed in 4% PFA and after washing in PBS, samples were incubated in fresh X-Gal staining solution for 36 h at 32 °C. After PBS washes, samples were post-fixed in 4% PFA at 4 °C for 16–24 h, decalcified, and embedded in OCT for cryosectioning and staining as previously described^37^.

### In vitro cartilage explant culture

A method previously described that reliably yields tissue that is highly cartilaginous, with no evidence of bone of stem cell markers was employed^14,15^. Cartilage explants were isolated from the central portion of the day 7 fracture callus using a dissecting microscope to remove any adherent non-cartilaginous tissues. Explants were minced, pooled, then separated randomly into treatment groups. Explants were grown in vitro for one week in serum-free hypertrophic chondrogenic medium [high glucose DMEM, 1% penicillin–streptomycin, 1% ITS + Premix (BD Biosciences Cat #354352), 1 mM sodium pyruvate, 100 ng/ml ascorbate-2- phosphate and 10^−7^ M dexamethasone] to promote hypertrophic maturation^14,15^. Hypertrophic cartilage explants were then stimulated with or without 200 ng/mL recombinant human β-NGF (Peprotech cat# 450-01) for 24 h, collected in TRIzol, then mRNA was extracted using RNeasy Mini Kit following the manufacturer’s instructions (Qiagen cat# 74104).

### RNA sequencing and analysis

After mRNA extraction from hypertrophic cartilage, samples were then further purified by sodium acetate and isopropanol precipitation. 200 ng RNA input from each sample was used with Quantseq 3′ mRNA-seq Library Prep Kit FWD (Lexogen, SKU:015.24). Approximately 20 million single-end 50 bp reads were generated for each library on a HiSeq 4000. Reads were first trimmed for adapters with Cutadapt version 2.5 and then mapped to the mouse mm10 genome using STAR version 2.5.3a. Following alignment, reads were counted using featureCount version 1.6.4. We then performed differential gene expression analysis using the DESeq2 package version 1.24 and R version 3.6.1. Significantly upregulated or downregulated genes (P < 0.05, Benjamini–Hochberg corrected) upon treatment were entered into Enrichr (https://amp.pharm.mssm.edu/Enrichr/) for gene ontology classification. Differentially expressed genes and genes of interest were visualized using a combination of R, ggplot2 version 3.2.1, EnhancedVolcano, and Complexheatmap version 2.0^84^.

### Immunohistochemistry (IHC)

β-NGF or control injections were administered once daily for 3 days beginning 7 days post-fracture into Axin2-eGFP mice. Fractured tibias were harvested 24 h after the final injection (10 days post-fracture), fixed in 4% paraformaldehyde (PFA) and decalcified in 19% ethylenediaminetetraacetic acid (EDTA) for 5 days. Samples were OCT-embedded then cryosections were made at a width of 8–10 μm. Cryosections were carefully rinsed in PBS and blocked with 5% bone serum albumin for an hour. Primary antibodies were applied to sections overnight. Full antibody information is given below (Table 2). Species-specific secondary antibodies were detected using the VectaStain ABC Kit (Vector, PK-4000) and 3,3′- diaminobenzidine (DAB) colorimetric reaction was used to visualize CD31 + cells. Because of (d2)eGFP’s rapid degradation, Axin2-eGFP fluorescence was stabilized by using species-specific Alexa-Fluor-488 conjugated secondary antibody (Table 2).

**Table 2:** Antibody database.

Antibody reference	
Host and target	Resource identification
Rat anti-mouse CD31	BD Biosciences Cat# 553370, RRID: AB_394816
Goat anti-rat Ig (biotinylated)	BD Biosciences Cat# 559286, RRID: AB_397214
Rabbit anti-GFP	Cell Sig Tech Cat# 2555, RRID:AB_10692764
Goat anti-rabbit IgG	Thermo Sci Cat# R-37116, RRID: AB_2556544

### Histomorphometry

β-NGF and control injections were administered once daily for 3 days beginning 7 days post-fracture. Tibias were harvested 14 days post-fracture, fixed in 4% PFA and decalcified in 19% EDTA for 5 days. Mice were processed for paraffin histology, serial sections were cut at 8–10 μm for histomorphometric analysis using stereological principles^15^. Quantification of callus composition (cartilage, bone, fibrous, marrow space) was determined using an Olympus CAST system (Center Valley, PA) and software by Visiopharm (Hørsholm, Denmark). For quantification of the tissues, 10 μm serial sections (three per slide) were taken through the entire leg. Tissue was stained with HBQ as described above, and the first section from every 10th slide analyzed such that sections were 300 μm apart. Volume of specific tissue types was determined in reference to the entire fracture callus by summing the individual compositions relative to the whole as previously described^15^.

### micro-computed tomography (μCT)

μCT analysis was performed as previously described^15,85^. Fracture tibias were dissected free of attached muscle 14 days post-fracture, fixed in 4% PFA and stored in 70% ethanol. Fracture calluses were analyzed using the Scanco μCT50 scanner (Scanco Medical AG, Basserdorf, Switzerland) with 10 μm voxel size and X-ray energies of 55 kVp and 109 μA. A lower excluding threshold of 400 mg hydroxyapatite (HA)/mm3 was applied to segment total mineralized bone matrix from soft tissue in studies of control and β-NGF treated mice. Linear attenuation was calibrated using a Scanco hydroxyapatite phantom. The regions of interest (ROI) included the entire callus without existing cortical clearly distinguished by its anatomical location and much higher mineral density. μCT reconstruction and quantitative analyses were performed to obtain the following structural parameters: trabecular spacing (mm), trabecular number (#/mm), trabecular connective density as trabecular bifurcations (#/mm^3^), bone mineral density (mg HA/cm^3^), bone volume (as %), trabecular thickness (mm), and tissue mineral density (mg HA/cm^3^).

### Statistical analysis

Individual dots on graphs represent biological replicates, error bars represent standard error of the mean (SEM). Measurements were taken from distinct samples. All in vivo data were analyzed using GraphPad Prism (version 8, GraphPad Software, San Diego, CA). For RNAseq data please see section on RNAsequencing and analysis. Statistical tests used to compare between groups are specified in the corresponding figure legends, significant differences were defined at p < 0.05.

## Supplementary Information

Supplementary Information

## Data Availability

All data needed to evaluate the conclusions are present in the paper and/or Supplementary Materials. Additional information related to this paper may be requested from the authors. RNA-seq data has been deposited into NCBI’s Gene Expression Omnibus (GEO) under accession code GSE150092.
