# Supplementary material for: Local injections of β-NGF accelerates endochondral fracture repair by promoting cartilage to bone conversion
Source: Sci Rep. 2020 Dec 17;10:22241. doi: 10.1038/s41598-020-78983-y (PMC7747641; doi:10.1038/s41598-020-78983-y)
Supplement: Supplementary file 1 — Supplementary Information [file 41598_2020_78983_MOESM1_ESM.pdf]

Supplemental Materials

Local injections of β-NGF accelerates endochondral fracture repair by promoting cartilage to bone conversion

Kevin O. Rivera[1,2,3], Fabrizio Russo[4], Ryan M. Boileau[5,6], Ryan Tomlinson[7], Theodore Miclau[2], Ralph S. Marcucio[1,2], Tejal A. Desai[1,3], Chelsea S. Bahney[1,2,8]

[1]Graduate Program in Oral and Craniofacial Sciences, School of Dentistry, University of California, San Francisco (UCSF), San Francisco, CA, USA
[2]Department of Orthopaedic Surgery, Orthopaedic Trauma Institute, University of California, San Francisco (UCSF), San Francisco, CA, USA
[3]Department of Bioengineering and Therapeutic Sciences, University of California, San Francisco (UCSF), San Francisco, CA, USA
[4]Department of Orthopaedics and Trauma Surgery, University Campus Bio-Medico, Rome, Italy
[5]The Eli and Edythe Broad Center of Regeneration Medicine and Stem Cell Research, Center for Reproductive Sciences, University of California, San Francisco (UCSF), San Francisco, CA, USA
[6]Department of Urology, University of California, San Francisco (UCSF), San Francisco, CA, USA
[7]Department of Orthopaedic Surgery, Thomas Jefferson University, Philadelphia, PA, USA
[8]The Steadman Philippon Research Institute (SPRI), Vail, CO, USA

Address for correspondence:
Chelsea S. Bahney
The Steadman Philippon Research Institute (SPRI)
181 W Meadows Drive, Suite 1000
Vail CO 81657
cbahney@sprivail.org or Chelsea.Bahney@UCSF.edu

Supplemental Table 1. Expression levels of genes of interest from β-NGF vs non-stimulated cartilage explants, generated by RNA sequencing

Gene Symbol	Log2 Fold Change – β-NGF vs Control	Log2FC Standard Error	P value	P adj	Ensembl Gene ID
Bglap (Oc)	1.66086954	0.16275	1.88E-24	6.34E-22	ENSMUSG00000074483
Bmp2	0.70113423	0.35781	5.00E-02	1.39E-01	ENSMUSG00000027358
Col1a1	0.9261786	0.11038	4.82E-17	6.55E-15	ENSMUSG00000001506
Csgalnact1	-0.7924748	0.26449	2.73E-03	1.47E-02	ENSMUSG00000036356
Csgalnact2	-1.019172	0.22075	3.90E-06	6.54E-05	ENSMUSG00000042042
Runx2	0.73622873	0.25738	4.23E-03	2.09E-02	ENSMUSG00000039153
Sox9	0.03472161	0.12185	7.76E-01	8.73E-01	ENSMUSG00000000567
Spp1 (Op)	-0.8230715	0.14671	2.02E-08	6.56E-07	ENSMUSG00000029304
Tgfb1	0.96324622	0.12627	2.38E-14	2.32E-12	ENSMUSG00000002603
Vegfa	0.19972043	0.16362	2.22E-01	3.97E-01	ENSMUSG00000023951

a

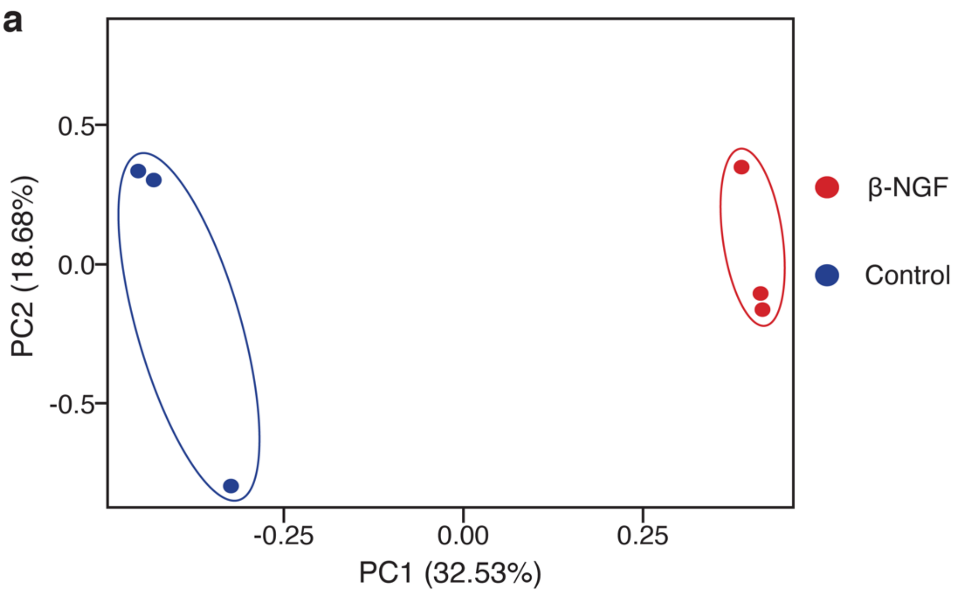

b

Gene ontology - downregulated molecular functions	p value	adj. p value	odds ratio
**RNA binding (GO: 003723)**	1.2E-87	1.4E-84	3.04
**mRNA binding (GO: 003729)**	1.7E-15	9.7E-13	3.44
**Cadherin binding (GO: 0045296)**	3.5E-14	1.3E-11	2.66
**Translation initiation factor activity (GO: 0003743)**	4.2E-13	1.2E-10	6.70
**Translation factor activity, RNA binding (GO: 0008135)**	4.2E-11	9.7E-09	4.50
**Ubiquitin protein ligase binding (GO: 0031625)**	6.4E-09	1.2E-06	2.29
**Ubiquitin-like protein ligase binding (GO: 0044389)**	1.2E-08	2.0E-07	2.23
**Ubiquitin protein ligase activity (GO: 0061630)**	1.3E-07	1.9E-05	2.45
**Ubiquitin-like protein ligase activity (GO: 0061659)**	4.7E-07	6.0E-05	2.40
**Ubiquitin-protein transferase activity (GO: 0004842)**	1.1E-06	1.3E-04	1.85

Supplemental Figure S1. Enrichment analysis of β-NGF stimulated hypertrophic cartilage explants. (**a**) Principal component analysis (PCA) for each biological replicate of β-NGF and non-stimulated controls. Gene ontology (GO) categorical grids for (**b**) downregulated molecular functions. To generate data: Cartilaginous tissue was excised from tibia fracture 7 days post-fracture and cultured to hypertrophy for 7 days then stimulated with or without recombinant human β-NGF. Samples were collected after 24 hours for RNA-seq analysis (n=3). GO terms, p values, and odds ratios were generated and computed by Enrichr. GO terms are sorted by p value, adjusted p value determined by Benjamini-Hochberg method.

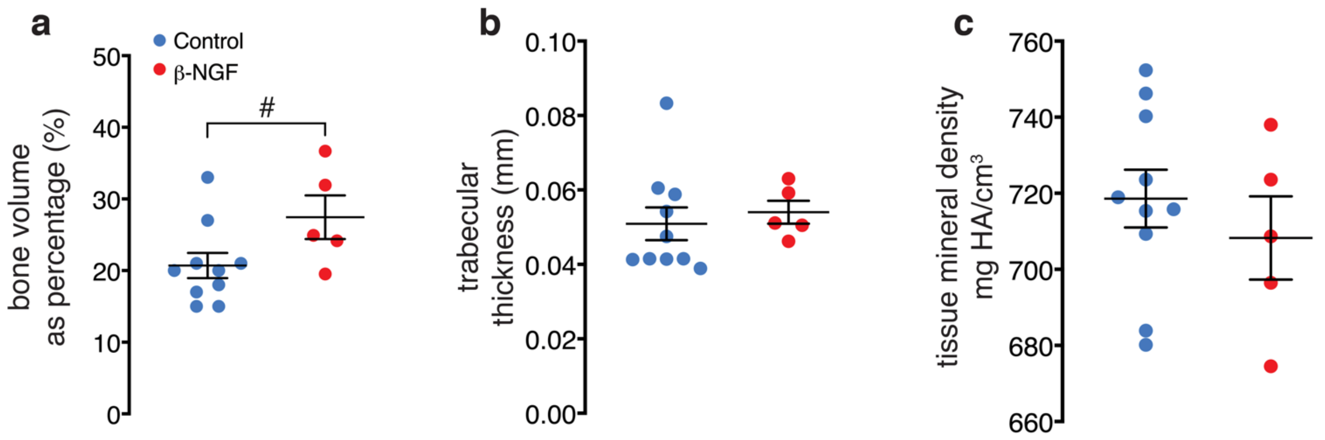

Supplemental Figure S2. µCT analysis of trabecular bone within fracture callus: Local injections of media (control) or 0.5 µg β-NGF were administered once daily at 7, 8, and 9 days post-fracture (p.f.), tibias were then harvested 14 days p.f. for µCT analysis. Quantification of (**a**) bone volume as percent composition (**b**) average trabecular thickness and (**c**) tissue mineral density is shown for control (n=10) and β-NGF (n=5) treated mice. # = p <0.1, determined by 2-tailed t test.

Supplemental Table 2. Differentially expressed genes of β-NGF vs non-stimulated cartilage explants, generated by RNA sequencing

Gene name	Log2 Fold Change – β-NGF vs Control	Log2FC Standard Error	P Value	P adj	Ensembl Gene ID
A330009N23Rik	1.227381921	0.47837013	1.03E-02	4.21E-02	ENSMUSG00000097908
A630072M18Rik	-3.146527536	1.04462289	2.59E-03	1.41E-02	ENSMUSG00000100826
A930001C03Rik	1.848550536	0.7287824	1.12E-02	4.49E-02	ENSMUSG00000087132
A930005H10Rik	0.684610342	0.21224849	1.26E-03	7.97E-03	ENSMUSG00000054426
Aagab	0.374100595	0.1461269	1.05E-02	4.27E-02	ENSMUSG00000037257
Aamdc	0.492125764	0.14936317	9.85E-04	6.52E-03	ENSMUSG00000035642
Aamp	0.457893946	0.14064317	1.13E-03	7.33E-03	ENSMUSG00000006299
Aasdhppt	-0.58647175	0.17843155	1.01E-03	6.67E-03	ENSMUSG00000025894
Abat	1.462800077	0.38383812	1.38E-04	1.30E-03	ENSMUSG00000057880
Abca2	0.671978095	0.20490783	1.04E-03	6.82E-03	ENSMUSG00000026944
Abcb10	0.828390328	0.28519378	3.68E-03	1.87E-02	ENSMUSG00000031974
Abcb8	1.426504588	0.38628182	2.22E-04	1.92E-03	ENSMUSG00000028973
Abcb9	2.050368173	0.54629864	1.75E-04	1.58E-03	ENSMUSG00000029408
Abcc1	-0.827942631	0.11507962	6.27E-13	5.32E-11	ENSMUSG00000023088
Abcd1	0.929308784	0.16595417	2.15E-08	6.89E-07	ENSMUSG00000031378
Abce1	-1.312052404	0.50970818	1.00E-02	4.14E-02	ENSMUSG00000058355
Abhd12	0.692115194	0.14394131	1.52E-06	2.93E-05	ENSMUSG00000032046
Abhd14a	0.502993161	0.15938403	1.60E-03	9.60E-03	ENSMUSG00000042210
Abhd14b	1.133227599	0.28043941	5.32E-05	6.05E-04	ENSMUSG00000042073
Abhd16a	1.215725497	0.15453559	3.63E-15	3.95E-13	ENSMUSG00000007036
Abhd17b	-0.716627064	0.22206637	1.25E-03	7.94E-03	ENSMUSG00000047368
Abhd5	-0.475643533	0.13106007	2.84E-04	2.35E-03	ENSMUSG00000032540
Abi1	-0.504462893	0.17964288	4.98E-03	2.39E-02	ENSMUSG00000058835
Abi2	-0.751482213	0.25430118	3.13E-03	1.64E-02	ENSMUSG00000026782
Abraxas2	-0.930632171	0.26277161	3.98E-04	3.11E-03	ENSMUSG00000030965
AC149090.1	-3.214344874	1.08216819	2.98E-03	1.58E-02	ENSMUSG00000094942
Acaa1a	0.419107511	0.13730742	2.27E-03	1.27E-02	ENSMUSG00000036138
Acaa2	0.619276685	0.11146257	2.76E-08	8.61E-07	ENSMUSG00000036880
Acadl	-0.449263377	0.13178367	6.52E-04	4.67E-03	ENSMUSG00000026003
Acadvl	0.41257815	0.13410348	2.09E-03	1.19E-02	ENSMUSG00000018574
Acan	0.826505329	0.14639636	1.65E-08	5.47E-07	ENSMUSG00000030607
Acap2	-0.818537866	0.23368203	4.60E-04	3.51E-03	ENSMUSG00000049076
Acap3	0.647627317	0.18929874	6.23E-04	4.49E-03	ENSMUSG00000029033
Acat2	0.889638497	0.16772822	1.13E-07	2.95E-06	ENSMUSG00000023832
Acbd3	-0.865652692	0.18982061	5.11E-06	8.23E-05	ENSMUSG00000026499
Acbd5	-0.758083079	0.27063407	5.09E-03	2.43E-02	ENSMUSG00000026781
Ace	0.490336803	0.16489127	2.94E-03	1.56E-02	ENSMUSG00000020681
Acot13	-0.57455246	0.12290454	2.94E-06	5.14E-05	ENSMUSG00000006717

Acp1	-0.533980761	0.14632506	2.63E-04	2.21E-03	ENSMUSG00000044573
Acp2	0.801248407	0.25270288	1.52E-03	9.22E-03	ENSMUSG00000002103
Acsl4	-1.010458375	0.17406692	6.44E-09	2.35E-07	ENSMUSG00000031278
Acta2	0.735187108	0.12900834	1.21E-08	4.21E-07	ENSMUSG00000035783
Actb	0.605897218	0.09901006	9.38E-10	4.30E-08	ENSMUSG00000029580
Actn4	0.279889635	0.10490155	7.63E-03	3.34E-02	ENSMUSG00000054808
Actr2	-0.739799971	0.204478	2.97E-04	2.44E-03	ENSMUSG00000020152
Actr3b	0.749998404	0.25277958	3.01E-03	1.59E-02	ENSMUSG00000056367
Acy3	1.274988754	0.30572246	3.04E-05	3.75E-04	ENSMUSG00000024866
Acyp2	1.749659991	0.56415403	1.93E-03	1.11E-02	ENSMUSG00000060923
Adam15	0.377648192	0.13887447	6.54E-03	2.96E-02	ENSMUSG00000028041
Adam19	0.864258499	0.23364865	2.16E-04	1.88E-03	ENSMUSG00000011256
Adam23	0.505778657	0.19053128	7.94E-03	3.44E-02	ENSMUSG00000025964
Adamts10	0.720909124	0.18119216	6.93E-05	7.50E-04	ENSMUSG00000024299
Adamts15	2.292570584	0.51262891	7.74E-06	1.17E-04	ENSMUSG00000033453
Adamts17	1.494637077	0.58944788	1.12E-02	4.50E-02	ENSMUSG00000058145
Adamts2	0.513008201	0.14316149	3.39E-04	2.73E-03	ENSMUSG00000036545
Adamts5	-1.137852045	0.21461949	1.15E-07	2.98E-06	ENSMUSG00000022894
Adamtsl1	0.924447229	0.23063128	6.11E-05	6.79E-04	ENSMUSG00000066113
Adck1	0.811818213	0.24233848	8.08E-04	5.59E-03	ENSMUSG00000021044
Adcy3	0.604928627	0.23248453	9.27E-03	3.88E-02	ENSMUSG00000020654
Adcy5	1.594189712	0.4940954	1.25E-03	7.95E-03	ENSMUSG00000022840
Adcy9	1.132682185	0.40378432	5.03E-03	2.41E-02	ENSMUSG00000005580
Add1	0.587088597	0.15146529	1.06E-04	1.05E-03	ENSMUSG00000029106
Adgrd1	1.455503039	0.3660751	7.01E-05	7.56E-04	ENSMUSG00000044017
Adgre5	0.794826615	0.21876298	2.80E-04	2.32E-03	ENSMUSG00000002885
Adh7	-0.578742113	0.19207673	2.59E-03	1.41E-02	ENSMUSG00000055301
Adipoq	2.421235445	0.68397561	4.00E-04	3.12E-03	ENSMUSG00000022878
Adrb3	3.411478625	0.98136567	5.08E-04	3.80E-03	ENSMUSG00000031489
Adss	-0.807542832	0.17585075	4.39E-06	7.24E-05	ENSMUSG00000015961
Aebp1	0.35295284	0.11102681	1.48E-03	9.00E-03	ENSMUSG00000020473
Aebp2	-0.788252822	0.24879912	1.53E-03	9.30E-03	ENSMUSG00000030232
Aes	0.459724042	0.13145574	4.70E-04	3.57E-03	ENSMUSG00000054452
Aff4	-0.812154454	0.17953639	6.08E-06	9.56E-05	ENSMUSG00000049470
Afg3l1	0.664180929	0.16764864	7.44E-05	7.95E-04	ENSMUSG00000031967
Afg3l2	-0.463119408	0.17714537	8.94E-03	3.77E-02	ENSMUSG00000024527
Aftph	-0.970068398	0.27988803	5.28E-04	3.92E-03	ENSMUSG00000049659
Agfg1	-0.464365609	0.1777036	8.97E-03	3.78E-02	ENSMUSG00000026159
Ago2	-0.652706882	0.18817867	5.23E-04	3.89E-03	ENSMUSG00000036698
Agpat2	0.58086638	0.17772203	1.08E-03	7.04E-03	ENSMUSG00000026922
Agpat3	0.720640407	0.12746778	1.57E-08	5.27E-07	ENSMUSG00000001211
Agpat4	0.326393957	0.11092341	3.26E-03	1.70E-02	ENSMUSG00000023827
Agt	0.562059434	0.12169394	3.86E-06	6.51E-05	ENSMUSG00000031980

Agtpbp1	-0.700577501	0.25990009	7.03E-03	3.14E-02	ENSMUSG00000021557
Agtrap	0.919526107	0.17671452	1.96E-07	4.77E-06	ENSMUSG00000029007
Ahctf1	-1.040477995	0.32813758	1.52E-03	9.22E-03	ENSMUSG00000026491
Ahdc1	0.517176622	0.15930467	1.17E-03	7.53E-03	ENSMUSG00000037692
Ahr	-1.733330964	0.49749559	4.94E-04	3.71E-03	ENSMUSG00000019256
Ahsa2	-0.560363146	0.20945361	7.46E-03	3.28E-02	ENSMUSG00000020288
Ahsg	7.621590868	1.53131031	6.45E-07	1.38E-05	ENSMUSG00000022868
AI413582	0.374369987	0.12107483	1.99E-03	1.14E-02	ENSMUSG00000062753
Aimp1	-0.582887148	0.13254869	1.09E-05	1.57E-04	ENSMUSG00000028029
Ak1	0.584090041	0.1306009	7.74E-06	1.17E-04	ENSMUSG00000026817
Ak3	0.607771909	0.12591075	1.39E-06	2.68E-05	ENSMUSG00000024782
Ak4	0.545874914	0.17105466	1.42E-03	8.71E-03	ENSMUSG00000028527
Ak6	-0.456964638	0.16513572	5.65E-03	2.64E-02	ENSMUSG00000078941
Akap12	-0.837924421	0.29150364	4.05E-03	2.01E-02	ENSMUSG00000038587
Akr1b10	1.057941266	0.30358282	4.92E-04	3.70E-03	ENSMUSG00000061758
Akr1c18	2.957520795	1.05703955	5.14E-03	2.45E-02	ENSMUSG00000021214
Akr7a5	0.372057413	0.13374335	5.40E-03	2.54E-02	ENSMUSG00000028743
Akt1	0.486775246	0.13500505	3.11E-04	2.54E-03	ENSMUSG00000001729
Akt3	-1.141011673	0.23655352	1.41E-06	2.73E-05	ENSMUSG00000019699
Alas1	0.617026268	0.17227312	3.41E-04	2.74E-03	ENSMUSG00000032786
Aldh16a1	1.231641581	0.27273816	6.31E-06	9.86E-05	ENSMUSG00000007833
Aldh1l2	-1.114102964	0.24510857	5.48E-06	8.74E-05	ENSMUSG00000020256
Aldh3a1	0.789576749	0.3023238	9.01E-03	3.79E-02	ENSMUSG00000019102
Aldh3b1	1.178207369	0.31453541	1.80E-04	1.61E-03	ENSMUSG00000024885
Aldh4a1	1.100951388	0.28908599	1.40E-04	1.31E-03	ENSMUSG00000028737
Aldh5a1	1.128921329	0.29811936	1.53E-04	1.41E-03	ENSMUSG00000035936
Aldh7a1	0.72614424	0.19244418	1.61E-04	1.47E-03	ENSMUSG00000053644
Aldoa	0.580422381	0.0941621	7.09E-10	3.35E-08	ENSMUSG00000030695
Aldoc	1.157741851	0.16752117	4.81E-12	3.54E-10	ENSMUSG00000017390
Alg10b	-1.066176678	0.38779151	5.97E-03	2.75E-02	ENSMUSG00000075470
Alg11	-1.056267715	0.26839413	8.30E-05	8.63E-04	ENSMUSG00000063362
Alkbh1	-0.60897971	0.19321861	1.62E-03	9.71E-03	ENSMUSG00000079036
Alox5ap	1.402288029	0.33222003	2.43E-05	3.10E-04	ENSMUSG00000060063
Alpl	1.742527994	0.13881255	3.82E-36	3.14E-33	ENSMUSG00000028766
Als2cl	1.540805555	0.23870363	1.08E-10	6.20E-09	ENSMUSG00000044037
Amd1	-1.080494723	0.38901371	5.48E-03	2.57E-02	ENSMUSG00000075232
Amdhd2	0.732780612	0.20976009	4.77E-04	3.60E-03	ENSMUSG00000036820
Ammecr1	-0.616296404	0.18389812	8.04E-04	5.57E-03	ENSMUSG00000042225
Amn1	-0.967212051	0.30148834	1.34E-03	8.33E-03	ENSMUSG00000068250
Amotl2	0.880084557	0.16388415	7.87E-08	2.14E-06	ENSMUSG00000032531
Ampd3	-1.456346958	0.18905228	1.32E-14	1.34E-12	ENSMUSG00000005686
Anapc13	0.690348136	0.13926329	7.15E-07	1.50E-05	ENSMUSG00000035048
Anapc16	-0.551682967	0.12859803	1.79E-05	2.40E-04	ENSMUSG00000020107

Anapc5	0.32764636	0.12178083	7.14E-03	3.17E-02	ENSMUSG00000029472
Angpt4	1.455207376	0.21179305	6.38E-12	4.61E-10	ENSMUSG00000027460
Angptl2	-0.644951024	0.1368678	2.45E-06	4.41E-05	ENSMUSG00000004105
Angptl4	0.484209213	0.130281	2.02E-04	1.78E-03	ENSMUSG00000002289
Angptl6	-0.60719927	0.16667694	2.70E-04	2.25E-03	ENSMUSG00000038742
Angptl7	1.497052541	0.2477407	1.51E-09	6.43E-08	ENSMUSG00000028989
Ankhd1	-0.892235533	0.28500457	1.74E-03	1.03E-02	ENSMUSG00000024483
Ankrd11	-0.898629127	0.19717164	5.17E-06	8.30E-05	ENSMUSG00000035569
Ankrd12	-1.125132361	0.35335978	1.45E-03	8.89E-03	ENSMUSG00000034647
Ankrd13a	0.493375475	0.14097015	4.66E-04	3.54E-03	ENSMUSG00000041870
Ankrd17	-0.800796038	0.20930291	1.30E-04	1.24E-03	ENSMUSG00000055204
Ankrd37	1.74415742	0.27313874	1.71E-10	9.32E-09	ENSMUSG00000050914
Ankrd46	0.710504718	0.14094408	4.63E-07	1.02E-05	ENSMUSG00000048307
Ankrd52	1.771572728	0.62278837	4.45E-03	2.18E-02	ENSMUSG00000014498
Ano10	0.512836008	0.20377666	1.18E-02	4.70E-02	ENSMUSG00000037949
Ano8	0.813362289	0.28378144	4.15E-03	2.06E-02	ENSMUSG00000034863
Anp32a	-0.50972407	0.17989271	4.60E-03	2.24E-02	ENSMUSG00000032249
Anp32b	-0.571123473	0.10940449	1.79E-07	4.40E-06	ENSMUSG00000028333
Antxr2	0.506245026	0.17747949	4.34E-03	2.13E-02	ENSMUSG00000029338
Anxa2	0.392128974	0.10099453	1.03E-04	1.03E-03	ENSMUSG00000032231
Aoc3	0.724903975	0.25885597	5.10E-03	2.43E-02	ENSMUSG00000019326
Aopep	1.299811952	0.14391416	1.69E-19	3.04E-17	ENSMUSG00000021458
Ap1b1	0.486920088	0.19200022	1.12E-02	4.49E-02	ENSMUSG00000009090
Ap1g2	0.920004141	0.29991244	2.16E-03	1.22E-02	ENSMUSG00000040701
Ap1s1	0.625637663	0.12834529	1.09E-06	2.18E-05	ENSMUSG00000004849
Ap2s1	0.48531418	0.18359991	8.21E-03	3.52E-02	ENSMUSG00000008036
Ap3d1	0.675256357	0.13678432	7.95E-07	1.65E-05	ENSMUSG00000020198
Ap3m1	-0.749147168	0.20512279	2.60E-04	2.19E-03	ENSMUSG00000021824
Ap5b1	2.70095288	0.62718313	1.66E-05	2.25E-04	ENSMUSG00000049562
Ap5s1	0.755628988	0.21736189	5.08E-04	3.80E-03	ENSMUSG00000068264
Ap5z1	0.777749124	0.27870504	5.26E-03	2.49E-02	ENSMUSG00000039623
Apbb1	0.950308365	0.264297	3.24E-04	2.63E-03	ENSMUSG00000037032
Apex1	0.543098853	0.15816302	5.95E-04	4.32E-03	ENSMUSG00000035960
Api5	-0.523701412	0.1524935	5.94E-04	4.32E-03	ENSMUSG00000027193
Apln	1.545034411	0.2179496	1.35E-12	1.05E-10	ENSMUSG00000037010
Aplp1	0.622537744	0.23178088	7.23E-03	3.21E-02	ENSMUSG00000006651
Apmap	1.085975994	0.24084171	6.51E-06	1.01E-04	ENSMUSG00000033096
Apobec1	-0.663700979	0.23298089	4.39E-03	2.15E-02	ENSMUSG00000040613
Apobr	1.977400631	0.43736681	6.15E-06	9.63E-05	ENSMUSG00000042759
Apod	0.423171645	0.15220489	5.43E-03	2.56E-02	ENSMUSG00000022548
Apoe	0.744274368	0.16595996	7.30E-06	1.12E-04	ENSMUSG00000002985
Apol6	2.851398975	0.61265823	3.25E-06	5.61E-05	ENSMUSG00000033576
Appl1	-0.86381603	0.22114611	9.38E-05	9.54E-04	ENSMUSG00000040760

Aqp1	-0.786719865	0.14276436	3.58E-08	1.08E-06	ENSMUSG00000004655
Aqp7	3.477376174	1.08339292	1.33E-03	8.29E-03	ENSMUSG00000028427
Araf	0.550188284	0.16480523	8.43E-04	5.78E-03	ENSMUSG00000001127
Arap1	0.902304376	0.18411212	9.54E-07	1.95E-05	ENSMUSG00000032812
Arap2	-0.845241058	0.2936105	3.99E-03	1.99E-02	ENSMUSG00000037999
Arf3	0.512096286	0.14813722	5.46E-04	4.03E-03	ENSMUSG00000051853
Arfgap2	0.791497087	0.29884612	8.08E-03	3.48E-02	ENSMUSG00000027255
Arfgef1	-1.242035036	0.24227522	2.95E-07	6.80E-06	ENSMUSG00000067851
Arfip1	-0.675407698	0.15855717	2.05E-05	2.69E-04	ENSMUSG00000074513
Arg2	-1.681642067	0.63775099	8.37E-03	3.58E-02	ENSMUSG00000021125
Arhgap1	0.581849626	0.16387489	3.84E-04	3.02E-03	ENSMUSG00000027247
Arhgap10	-0.629710146	0.20467273	2.09E-03	1.19E-02	ENSMUSG00000037148
Arhgap21	-0.683403791	0.2633	9.44E-03	3.94E-02	ENSMUSG00000036591
Arhgap23	0.71456901	0.17084817	2.88E-05	3.58E-04	ENSMUSG00000049807
Arhgap44	-0.690722002	0.26907149	1.03E-02	4.20E-02	ENSMUSG00000033389
Arhgap45	1.345397463	0.53815702	1.24E-02	4.86E-02	ENSMUSG00000035697
Arhgap5	-1.010597549	0.2134618	2.20E-06	4.03E-05	ENSMUSG00000035133
Arhgdia	0.336411257	0.12648713	7.82E-03	3.40E-02	ENSMUSG00000025132
Arhgdib	0.472080084	0.12170823	1.05E-04	1.04E-03	ENSMUSG00000030220
Arhgef1	0.523937114	0.15859263	9.54E-04	6.37E-03	ENSMUSG00000040940
Arhgef17	0.539881197	0.15481071	4.88E-04	3.68E-03	ENSMUSG00000032875
Arhgef25	0.461264292	0.15169315	2.36E-03	1.31E-02	ENSMUSG00000019467
Arhgef40	0.815695588	0.19262439	2.29E-05	2.95E-04	ENSMUSG00000004562
Arid4a	-0.867504976	0.33271281	9.12E-03	3.83E-02	ENSMUSG00000048118
Arid4b	-1.365958555	0.30580877	7.94E-06	1.19E-04	ENSMUSG00000039219
Arid5b	-0.803198538	0.18509721	1.43E-05	1.98E-04	ENSMUSG00000019947
Arih1	-0.868540478	0.18132281	1.67E-06	3.17E-05	ENSMUSG00000025234
Arl1	-1.059490438	0.15079535	2.13E-12	1.61E-10	ENSMUSG00000060904
Arl14ep	-0.701162505	0.23982645	3.46E-03	1.78E-02	ENSMUSG00000027122
Arl14epl	-4.725950461	1.13429897	3.09E-05	3.81E-04	ENSMUSG00000073568
Arl15	-1.02840313	0.31784052	1.21E-03	7.76E-03	ENSMUSG00000042348
Arl2	0.390536991	0.13327161	3.39E-03	1.75E-02	ENSMUSG00000024944
Arl4d	-1.282100541	0.39602071	1.21E-03	7.73E-03	ENSMUSG00000034936
Armc1	-0.478155526	0.14945668	1.38E-03	8.54E-03	ENSMUSG00000027599
Armc10	-0.864499241	0.26089643	9.21E-04	6.22E-03	ENSMUSG00000038525
Armc5	0.94281376	0.30741678	2.16E-03	1.22E-02	ENSMUSG00000042178
Armcx2	0.516218478	0.17116393	2.56E-03	1.40E-02	ENSMUSG00000033436
Armcx3	-0.676127209	0.23836058	4.56E-03	2.22E-02	ENSMUSG00000049047
Arpc1a	0.359530185	0.13406071	7.32E-03	3.24E-02	ENSMUSG00000029621
Arpc1b	0.360422721	0.12839183	5.00E-03	2.39E-02	ENSMUSG00000029622
Arpc4	0.391005518	0.1125294	5.11E-04	3.82E-03	ENSMUSG00000079426
Arpc5l	-0.483539912	0.13204615	2.50E-04	2.12E-03	ENSMUSG00000026755
Arpin	0.763437168	0.23554964	1.19E-03	7.66E-03	ENSMUSG00000039043

Arrb1	1.269302673	0.23759586	9.18E-08	2.45E-06	ENSMUSG00000018909
Arrdc1	0.880213841	0.3132412	4.95E-03	2.38E-02	ENSMUSG00000026972
Arrdc4	-0.685232332	0.12677052	6.47E-08	1.81E-06	ENSMUSG00000042659
Arsg	1.350377157	0.39866312	7.06E-04	4.99E-03	ENSMUSG00000020604
Arvcf	2.866163899	0.28891461	3.39E-23	1.04E-20	ENSMUSG00000000325
Arxes2	1.2791248	0.42198634	2.44E-03	1.35E-02	ENSMUSG00000048040
Asap1	-0.632940128	0.21420486	3.13E-03	1.64E-02	ENSMUSG00000022377
Asb1	0.780772973	0.31361925	1.28E-02	4.97E-02	ENSMUSG00000026311
Asna1	0.410234118	0.14083712	3.58E-03	1.83E-02	ENSMUSG00000052456
Asns	-0.839262495	0.1185995	1.48E-12	1.13E-10	ENSMUSG00000029752
Asnsd1	-0.810136905	0.17360383	3.06E-06	5.33E-05	ENSMUSG00000026095
Aspa	0.975290687	0.38772806	1.19E-02	4.71E-02	ENSMUSG00000020774
Asxl2	-1.133433678	0.30982799	2.54E-04	2.14E-03	ENSMUSG00000037486
Atad1	-0.985185003	0.21364884	4.00E-06	6.69E-05	ENSMUSG00000013662
Atad2b	-0.895584898	0.28000117	1.38E-03	8.55E-03	ENSMUSG00000052812
Ate1	-0.58763971	0.19823066	3.03E-03	1.60E-02	ENSMUSG00000030850
Atf1	-0.719713264	0.20042999	3.30E-04	2.67E-03	ENSMUSG00000023027
Atf4	-0.524150885	0.117093	7.59E-06	1.15E-04	ENSMUSG00000042406
Atf6	-0.710794492	0.27642159	1.01E-02	4.16E-02	ENSMUSG00000026663
Atf6b	0.611574242	0.192391	1.48E-03	9.00E-03	ENSMUSG00000015461
Atf7ip	-0.881620103	0.23062276	1.32E-04	1.25E-03	ENSMUSG00000030213
Atg12	-0.662757567	0.14870603	8.32E-06	1.24E-04	ENSMUSG00000032905
Atg4c	-0.824843186	0.30310616	6.50E-03	2.94E-02	ENSMUSG00000028550
Atg5	-0.650834281	0.18162506	3.39E-04	2.73E-03	ENSMUSG00000038160
Atl2	-1.128626804	0.2324727	1.20E-06	2.37E-05	ENSMUSG00000059811
Atoh8	1.97863204	0.29667402	2.57E-11	1.66E-09	ENSMUSG00000037621
Atp13a1	0.626706493	0.16955967	2.19E-04	1.90E-03	ENSMUSG00000031862
Atp13a2	0.783870575	0.29503433	7.89E-03	3.42E-02	ENSMUSG00000036622
Atp13a3	-1.25162704	0.1766161	1.37E-12	1.06E-10	ENSMUSG00000022533
Atp2b1	-0.725318932	0.2344305	1.98E-03	1.13E-02	ENSMUSG00000019943
Atp5b	0.314972168	0.10531808	2.78E-03	1.49E-02	ENSMUSG00000025393
Atp5g3	0.451777602	0.13518166	8.32E-04	5.71E-03	ENSMUSG00000018770
Atp5k	0.462664899	0.10368638	8.11E-06	1.21E-04	ENSMUSG00000050856
Atp6ap1	0.537975666	0.14667093	2.45E-04	2.08E-03	ENSMUSG00000019087
Atp6ap2	-0.479469995	0.15440681	1.90E-03	1.10E-02	ENSMUSG00000031007
Atp6v0a1	0.90798547	0.19061255	1.90E-06	3.56E-05	ENSMUSG00000019302
Atp6v0d1	0.391764346	0.14226003	5.89E-03	2.72E-02	ENSMUSG00000013160
Atp6v0d2	-1.487893765	0.37793483	8.25E-05	8.60E-04	ENSMUSG00000028238
Atp6v0e	0.300001424	0.10030756	2.78E-03	1.49E-02	ENSMUSG00000015575
Atp6v1a	-0.545192195	0.15796359	5.58E-04	4.10E-03	ENSMUSG00000052459
Atp6v1c1	-0.595092846	0.1479919	5.79E-05	6.46E-04	ENSMUSG00000022295
Atp6v1f	0.359993916	0.11244858	1.37E-03	8.49E-03	ENSMUSG00000004285
Atp7a	-0.899112218	0.33591693	7.44E-03	3.28E-02	ENSMUSG00000033792

Atp8b1	-1.501948708	0.43851816	6.15E-04	4.44E-03	ENSMUSG00000039529
Atp9a	0.738601499	0.19697074	1.77E-04	1.59E-03	ENSMUSG00000027546
Atp9b	0.698520377	0.23461433	2.91E-03	1.55E-02	ENSMUSG00000024566
Atpsckmt	0.898621056	0.20456846	1.12E-05	1.60E-04	ENSMUSG00000039065
Atraid	0.569772171	0.11212934	3.75E-07	8.43E-06	ENSMUSG00000013622
Atrx	-0.650774643	0.2557472	1.09E-02	4.42E-02	ENSMUSG00000031229
Atxn7l3	0.398145489	0.1482995	7.26E-03	3.21E-02	ENSMUSG00000059995
AU020206	-1.462465897	0.57151219	1.05E-02	4.28E-02	ENSMUSG00000097392
AV099323	1.202627592	0.43530957	5.73E-03	2.67E-02	ENSMUSG00000087377
Aven	0.79298155	0.25308494	1.73E-03	1.02E-02	ENSMUSG00000003604
Avil	0.581856243	0.21093187	5.81E-03	2.70E-02	ENSMUSG00000025432
Avpi1	-0.433136878	0.13029261	8.86E-04	6.02E-03	ENSMUSG00000018821
Azi2	-0.705617389	0.20167698	4.67E-04	3.55E-03	ENSMUSG00000039285
Azin1	-0.564292031	0.16574041	6.62E-04	4.72E-03	ENSMUSG00000037458
B230219D22Rik	-0.734516251	0.21926445	8.08E-04	5.59E-03	ENSMUSG00000045767
B3galt6	0.705116969	0.28063958	1.20E-02	4.74E-02	ENSMUSG00000050796
B3gnt8	2.268365755	0.40054033	1.49E-08	5.01E-07	ENSMUSG00000059479
B3gnt9	0.781844403	0.2609447	2.73E-03	1.47E-02	ENSMUSG00000069920
B4galnt1	0.353984438	0.14035241	1.17E-02	4.63E-02	ENSMUSG00000006731
B4galt1	0.62218487	0.12532519	6.89E-07	1.45E-05	ENSMUSG00000028413
B4gat1	1.122035446	0.16336133	6.49E-12	4.64E-10	ENSMUSG00000047379
B9d1	1.17869999	0.21367206	3.46E-08	1.05E-06	ENSMUSG00000001039
Babam1	0.321618221	0.11663475	5.82E-03	2.70E-02	ENSMUSG00000031820
Bace1	0.549827515	0.14430961	1.39E-04	1.31E-03	ENSMUSG00000032086
Bad	0.525612441	0.12321884	1.99E-05	2.64E-04	ENSMUSG00000024959
Bag3	0.602558916	0.15471243	9.83E-05	9.90E-04	ENSMUSG00000030847
Bag6	0.348774021	0.13159822	8.04E-03	3.47E-02	ENSMUSG00000024392
Bambi	0.999548442	0.21333072	2.79E-06	4.95E-05	ENSMUSG00000024232
Baz1a	-0.855078365	0.22604335	1.55E-04	1.43E-03	ENSMUSG00000035021
Bbx	-0.76076647	0.29129384	9.01E-03	3.79E-02	ENSMUSG00000022641
BC002059	-1.669164148	0.60659672	5.93E-03	2.74E-02	ENSMUSG00000060149
BC004004	0.393859821	0.11417786	5.62E-04	4.12E-03	ENSMUSG00000052712
BC005537	-0.691105167	0.15511	8.37E-06	1.25E-04	ENSMUSG00000019132
BC005561	-0.663787586	0.22583285	3.29E-03	1.71E-02	ENSMUSG00000079065
BC029722	1.068645728	0.16211279	4.34E-11	2.76E-09	ENSMUSG00000074649
BC031181	0.306274452	0.11558605	8.06E-03	3.48E-02	ENSMUSG00000036299
Bcar1	0.557407018	0.15209251	2.47E-04	2.10E-03	ENSMUSG00000031955
Bcar3	1.545651259	0.39324731	8.48E-05	8.77E-04	ENSMUSG00000028121
Bcas2	-0.377188937	0.147194	1.04E-02	4.24E-02	ENSMUSG00000005687
Bcat1	-0.501638696	0.14572025	5.76E-04	4.22E-03	ENSMUSG00000030268
Bcat2	0.358204611	0.14234385	1.19E-02	4.70E-02	ENSMUSG00000030826
Bccip	-0.565706873	0.21175043	7.55E-03	3.31E-02	ENSMUSG00000030983
Bckdk	0.839885794	0.16640724	4.48E-07	9.94E-06	ENSMUSG00000030802

Bcl2l1	-0.475939979	0.12016512	7.47E-05	7.98E-04	ENSMUSG00000007659
Bcl6	-0.574187971	0.16502284	5.02E-04	3.76E-03	ENSMUSG00000022508
Bcl9l	0.868413619	0.18646725	3.21E-06	5.54E-05	ENSMUSG00000063382
Bclaf3	-1.397988598	0.46140415	2.45E-03	1.35E-02	ENSMUSG00000044150
Bcs1l	0.546072251	0.1871637	3.53E-03	1.81E-02	ENSMUSG00000026172
Bdp1	-1.087177218	0.32524512	8.30E-04	5.70E-03	ENSMUSG00000049658
Bend6	-0.981685822	0.38888675	1.16E-02	4.61E-02	ENSMUSG00000042182
Best1	1.065763367	0.31630798	7.53E-04	5.28E-03	ENSMUSG00000037418
Bet1	-0.677169431	0.14816715	4.87E-06	7.92E-05	ENSMUSG00000032757
Bet1l	-0.423222546	0.11465567	2.23E-04	1.93E-03	ENSMUSG00000025484
Bfar	-0.698584645	0.22454805	1.86E-03	1.09E-02	ENSMUSG00000022684
Bglap	1.660869543	0.16274596	1.88E-24	6.34E-22	ENSMUSG00000074483
Bglap2	1.597764934	0.15147686	5.19E-26	2.07E-23	ENSMUSG00000074486
Bicral	-0.827565862	0.30061739	5.91E-03	2.73E-02	ENSMUSG00000036568
Birc6	-0.696253101	0.21989563	1.54E-03	9.33E-03	ENSMUSG00000024073
Bloc1s3	1.245808486	0.46800302	7.77E-03	3.38E-02	ENSMUSG00000057667
Blvra	0.410466297	0.15963677	1.01E-02	4.16E-02	ENSMUSG00000001999
Blvrb	-0.769523739	0.10533164	2.76E-13	2.45E-11	ENSMUSG00000040466
Blzf1	-1.000190518	0.28673992	4.86E-04	3.67E-03	ENSMUSG00000026577
Bmi1	-0.721352014	0.28298585	1.08E-02	4.37E-02	ENSMUSG00000026739
Bmp1	1.089366648	0.16589483	5.15E-11	3.23E-09	ENSMUSG00000022098
Bmp3	1.493257239	0.45155882	9.43E-04	6.32E-03	ENSMUSG00000029335
Bmp4	0.758487849	0.13181768	8.71E-09	3.13E-07	ENSMUSG00000021835
Bmp6	-0.983183129	0.39467033	1.27E-02	4.95E-02	ENSMUSG00000039004
Bmper	0.517456613	0.16946632	2.26E-03	1.26E-02	ENSMUSG00000031963
Bms1	-0.788401023	0.21610542	2.64E-04	2.21E-03	ENSMUSG00000030138
Bmyc	1.283419517	0.20265188	2.40E-10	1.28E-08	ENSMUSG00000049086
Bnip2	-0.349516603	0.13332125	8.75E-03	3.71E-02	ENSMUSG00000011958
Bnip3	0.741639305	0.14347823	2.35E-07	5.58E-06	ENSMUSG00000078566
Boc	0.417853509	0.13704129	2.30E-03	1.28E-02	ENSMUSG00000022687
Bod1l	-1.057952969	0.28530213	2.09E-04	1.82E-03	ENSMUSG00000061755
Borcs6	0.697563101	0.17126836	4.64E-05	5.39E-04	ENSMUSG00000045176
Borcs8	0.934389424	0.1465979	1.84E-10	9.95E-09	ENSMUSG00000002345
Bphl	0.599607048	0.1810844	9.29E-04	6.27E-03	ENSMUSG00000038286
Bptf	-0.61878639	0.2474631	1.24E-02	4.86E-02	ENSMUSG00000040481
Braf	-1.077786564	0.25279259	2.01E-05	2.66E-04	ENSMUSG00000002413
Brcc3	-0.659779562	0.26352505	1.23E-02	4.82E-02	ENSMUSG00000031201
Brd3	-0.403568552	0.14800289	6.40E-03	2.91E-02	ENSMUSG00000026918
Brd4	-0.366779486	0.13762254	7.70E-03	3.36E-02	ENSMUSG00000024002
Brix1	-1.244271451	0.20198303	7.26E-10	3.41E-08	ENSMUSG00000022247
Bscl2	1.344377835	0.1314258	1.47E-24	5.08E-22	ENSMUSG00000071657
Bsg	0.67977288	0.10914723	4.72E-10	2.34E-08	ENSMUSG00000023175
Btaf1	-0.677494608	0.23091466	3.35E-03	1.73E-02	ENSMUSG00000040565

Btbd10	-0.894234646	0.3523377	1.11E-02	4.48E-02	ENSMUSG00000038187
Btbd19	0.912474293	0.29947728	2.31E-03	1.29E-02	ENSMUSG00000073771
Btf3l4	-0.92053809	0.35678023	9.88E-03	4.08E-02	ENSMUSG00000028568
Btg2	-0.674471341	0.20168414	8.25E-04	5.68E-03	ENSMUSG00000020423
Bzw2	-0.615825094	0.11366089	6.02E-08	1.70E-06	ENSMUSG00000020547
C030006K11Rik	0.358946695	0.10444181	5.89E-04	4.29E-03	ENSMUSG00000115987
C130021I20Rik	0.966169409	0.29236826	9.51E-04	6.36E-03	ENSMUSG00000052951
C130050O18Rik	1.950915079	0.59518208	1.05E-03	6.85E-03	ENSMUSG00000044092
C1d	-0.586918451	0.16688342	4.37E-04	3.36E-03	ENSMUSG00000000581
C1galt1	-1.167475207	0.3005838	1.03E-04	1.02E-03	ENSMUSG00000042460
C1qa	2.141226671	0.28297223	3.82E-14	3.67E-12	ENSMUSG00000036887
C1qb	2.098971842	0.16416632	1.97E-37	1.85E-34	ENSMUSG00000036905
C1qbp	-0.512252841	0.12342353	3.32E-05	4.06E-04	ENSMUSG00000018446
C1qc	2.215406827	0.25767847	8.14E-18	1.20E-15	ENSMUSG00000036896
C1qtnf1	1.323419541	0.2434449	5.44E-08	1.55E-06	ENSMUSG00000017446
C1qtnf12	0.646316884	0.14132074	4.80E-06	7.82E-05	ENSMUSG00000023571
C1qtnf3	-0.646367322	0.16850415	1.25E-04	1.20E-03	ENSMUSG00000058914
C1qtnf6	1.033779756	0.2069597	5.88E-07	1.27E-05	ENSMUSG00000022440
C1ra	0.598119836	0.20753453	3.95E-03	1.98E-02	ENSMUSG00000055172
C1rl	1.685936751	0.26411727	1.73E-10	9.43E-09	ENSMUSG00000038527
C77080	-0.718083342	0.25898329	5.56E-03	2.61E-02	ENSMUSG00000050390
Cacna1g	1.039162129	0.3619054	4.09E-03	2.03E-02	ENSMUSG00000020866
Cacul1	-0.507053291	0.20252617	1.23E-02	4.82E-02	ENSMUSG00000033417
Cacybp	-1.313759622	0.24253546	6.07E-08	1.70E-06	ENSMUSG00000014226
Cadm1	1.20028647	0.22792138	1.39E-07	3.54E-06	ENSMUSG00000032076
Cald1	-0.789702854	0.29144633	6.74E-03	3.03E-02	ENSMUSG00000029761
Calhm2	1.000798747	0.2818579	3.84E-04	3.02E-03	ENSMUSG00000033033
Calhm5	0.630535592	0.24394264	9.74E-03	4.03E-02	ENSMUSG00000049872
Calm3	0.590169544	0.11186306	1.32E-07	3.39E-06	ENSMUSG00000019370
Calr	0.825405703	0.09872463	6.24E-17	8.29E-15	ENSMUSG00000003814
Caly	-3.341654429	0.94095864	3.83E-04	3.02E-03	ENSMUSG00000025468
Camk1	0.479511982	0.14730346	1.13E-03	7.34E-03	ENSMUSG00000030272
Camk2b	1.572228093	0.58703955	7.40E-03	3.27E-02	ENSMUSG00000057897
Camta2	0.917357802	0.27292693	7.76E-04	5.41E-03	ENSMUSG00000040712
Canx	-0.451354644	0.1471663	2.16E-03	1.22E-02	ENSMUSG00000020368
Capn1	0.829324156	0.21428174	1.09E-04	1.07E-03	ENSMUSG00000024942
Capns1	0.544843769	0.09245776	3.79E-09	1.46E-07	ENSMUSG00000001794
Caprin1	-0.790836319	0.1775218	8.39E-06	1.25E-04	ENSMUSG00000027184
Capza1	-0.789606811	0.29441987	7.32E-03	3.24E-02	ENSMUSG00000070372
Capza2	-0.485551587	0.16119406	2.59E-03	1.41E-02	ENSMUSG00000015733
Car5b	1.091796468	0.20692234	1.32E-07	3.39E-06	ENSMUSG00000031373
Car6	-1.259822466	0.24543736	2.85E-07	6.65E-06	ENSMUSG00000028972
Car9	0.938209564	0.24988594	1.74E-04	1.57E-03	ENSMUSG00000028463

Cars	-0.605013949	0.15015534	5.60E-05	6.30E-04	ENSMUSG00000010755
Cars2	0.878869932	0.31173497	4.81E-03	2.32E-02	ENSMUSG00000056228
Casp12	-0.768837383	0.233776	1.01E-03	6.64E-03	ENSMUSG00000025887
Casp3	-1.010670459	0.2631231	1.23E-04	1.18E-03	ENSMUSG00000031628
Casp4	-1.589255672	0.3363018	2.29E-06	4.19E-05	ENSMUSG00000033538
Cast	-0.855093475	0.21423843	6.57E-05	7.17E-04	ENSMUSG00000021585
Castor1	0.73974659	0.22265207	8.92E-04	6.06E-03	ENSMUSG00000020424
Cav2	-1.176773072	0.22520812	1.74E-07	4.29E-06	ENSMUSG00000000058
Cavin1	0.432584403	0.12118032	3.57E-04	2.84E-03	ENSMUSG00000004044
Cavin3	0.558655789	0.11343889	8.45E-07	1.75E-05	ENSMUSG00000037060
Cbll1	-1.09720757	0.29886472	2.41E-04	2.06E-03	ENSMUSG00000020659
Cbr2	1.700590626	0.15805897	5.36E-27	2.28E-24	ENSMUSG00000025150
Cbr3	-0.427467772	0.12565639	6.69E-04	4.75E-03	ENSMUSG00000022947
Cbx1	-0.615265621	0.12147245	4.08E-07	9.16E-06	ENSMUSG00000018666
Cbx3	-1.520272538	0.41704458	2.67E-04	2.23E-03	ENSMUSG00000029836
Cbx6	0.418626345	0.12867835	1.14E-03	7.38E-03	ENSMUSG00000089715
Cc2d1b	0.584062975	0.20097361	3.66E-03	1.86E-02	ENSMUSG00000028582
Cc2d2a	-1.139460353	0.41411386	5.93E-03	2.74E-02	ENSMUSG00000039765
Ccbe1	-0.955640495	0.27140695	4.30E-04	3.32E-03	ENSMUSG00000046318
Ccdc107	0.544755112	0.14168116	1.21E-04	1.17E-03	ENSMUSG00000028461
Ccdc117	-0.686523125	0.22273463	2.05E-03	1.17E-02	ENSMUSG00000020482
Ccdc12	0.617785281	0.14346387	1.66E-05	2.25E-04	ENSMUSG00000019659
Ccdc127	-0.520726141	0.16832793	1.98E-03	1.13E-02	ENSMUSG00000021578
Ccdc159	3.446054145	0.68173527	4.31E-07	9.60E-06	ENSMUSG00000006241
Ccdc167	0.897542586	0.19456732	3.97E-06	6.65E-05	ENSMUSG00000024018
Ccdc17	2.027979881	0.59731015	6.86E-04	4.86E-03	ENSMUSG00000034035
Ccdc186	-1.101949279	0.31663188	5.01E-04	3.75E-03	ENSMUSG00000035173
Ccdc22	0.886731992	0.2742959	1.23E-03	7.83E-03	ENSMUSG00000031143
Ccdc28a	0.932553815	0.34223676	6.43E-03	2.92E-02	ENSMUSG00000059554
Ccdc28b	0.881851182	0.23476471	1.72E-04	1.56E-03	ENSMUSG00000028795
Ccdc3	0.382369988	0.15068821	1.12E-02	4.48E-02	ENSMUSG00000026676
Ccdc34	-0.602298091	0.17420097	5.45E-04	4.03E-03	ENSMUSG00000027160
Ccdc43	-0.595279369	0.2292533	9.42E-03	3.93E-02	ENSMUSG00000020925
Ccdc47	-1.015974124	0.19653893	2.35E-07	5.58E-06	ENSMUSG00000078622
Ccdc50	-0.489670007	0.19202321	1.08E-02	4.37E-02	ENSMUSG00000038127
Ccdc59	-0.769397523	0.16295889	2.34E-06	4.24E-05	ENSMUSG00000019897
Ccdc6	-0.506221939	0.17182427	3.22E-03	1.68E-02	ENSMUSG00000048701
Ccdc71l	0.758260119	0.1763863	1.72E-05	2.31E-04	ENSMUSG00000090946
Ccdc77	-1.520057691	0.49111671	1.97E-03	1.13E-02	ENSMUSG00000030177
Ccdc8	0.410823688	0.15734847	9.03E-03	3.80E-02	ENSMUSG00000041117
Ccn1	0.767069524	0.21892015	4.59E-04	3.50E-03	ENSMUSG00000028195
Ccnb2	1.052097055	0.33380292	1.62E-03	9.71E-03	ENSMUSG00000032218
Ccnd1	1.590059761	0.11146698	3.62E-46	6.81E-43	ENSMUSG00000070348

Ccnd2	-0.42530142	0.14843942	4.17E-03	2.06E-02	ENSMUSG00000000184
Ccnd3	0.501638872	0.15067993	8.71E-04	5.94E-03	ENSMUSG00000034165
Ccng1	-0.594809588	0.1663352	3.49E-04	2.79E-03	ENSMUSG00000020326
Ccnt1	-0.871446248	0.21890328	6.86E-05	7.44E-04	ENSMUSG00000011960
Ccpg1os	0.820064859	0.21790145	1.68E-04	1.52E-03	ENSMUSG00000086158
Ccser2	-0.522134946	0.20123081	9.47E-03	3.94E-02	ENSMUSG00000058690
Cct2	-0.425068483	0.10296957	3.66E-05	4.37E-04	ENSMUSG00000034024
Cct4	-0.981011427	0.14509651	1.37E-11	9.49E-10	ENSMUSG00000007739
Cct5	-0.630868136	0.11492266	4.03E-08	1.20E-06	ENSMUSG00000022234
Cct6a	-0.311137497	0.11081012	4.99E-03	2.39E-02	ENSMUSG00000029447
Cct8	-0.628595172	0.13002009	1.33E-06	2.60E-05	ENSMUSG00000025613
Cd109	-0.943702423	0.22455875	2.64E-05	3.33E-04	ENSMUSG00000046186
Cd151	0.415925174	0.11198637	2.04E-04	1.79E-03	ENSMUSG00000025510
Cd1d1	0.882002993	0.26244618	7.77E-04	5.42E-03	ENSMUSG00000028076
Cd200	0.881252161	0.14432451	1.02E-09	4.54E-08	ENSMUSG00000022661
Cd200r4	-1.934481084	0.59548576	1.16E-03	7.49E-03	ENSMUSG00000062082
Cd248	1.967163701	0.18814359	1.38E-25	5.05E-23	ENSMUSG00000056481
Cd320	0.536507929	0.17197471	1.81E-03	1.06E-02	ENSMUSG00000002308
Cd36	-1.260265362	0.33955116	2.06E-04	1.80E-03	ENSMUSG00000002944
Cd47	-0.443326421	0.14325015	1.97E-03	1.13E-02	ENSMUSG00000055447
Cd53	-0.626395181	0.22546256	5.46E-03	2.57E-02	ENSMUSG00000040747
Cd59a	0.693572319	0.25270348	6.06E-03	2.78E-02	ENSMUSG00000032679
Cd74	-0.94656962	0.2628109	3.16E-04	2.57E-03	ENSMUSG00000024610
Cd81	0.467756568	0.09603276	1.11E-06	2.21E-05	ENSMUSG00000037706
Cd9	0.540334503	0.14055744	1.21E-04	1.17E-03	ENSMUSG00000030342
Cdc16	-0.379944134	0.1482212	1.04E-02	4.24E-02	ENSMUSG00000038416
Cdc34	-0.336911358	0.1289029	8.96E-03	3.78E-02	ENSMUSG00000020307
Cdc37l1	-0.441230305	0.16573392	7.76E-03	3.38E-02	ENSMUSG00000024780
Cdc42ep1	1.616658003	0.19947963	5.30E-16	6.46E-14	ENSMUSG00000049521
Cdc42ep2	1.256629706	0.32229005	9.66E-05	9.77E-04	ENSMUSG00000045664
Cdc42ep3	1.146707704	0.19671602	5.57E-09	2.07E-07	ENSMUSG00000036533
Cdc42ep4	0.336884572	0.13491575	1.25E-02	4.89E-02	ENSMUSG00000041598
Cdc42se1	0.537730042	0.13924125	1.13E-04	1.10E-03	ENSMUSG00000046722
Cdc5l	-1.172825803	0.30284921	1.08E-04	1.06E-03	ENSMUSG00000023932
Cdc73	-0.841909069	0.24136446	4.86E-04	3.67E-03	ENSMUSG00000026361
Cdh11	0.831178475	0.24224806	6.01E-04	4.36E-03	ENSMUSG00000031673
Cdipt	0.382767609	0.14441632	8.04E-03	3.47E-02	ENSMUSG00000030682
Cdk10	0.516001321	0.19293441	7.48E-03	3.29E-02	ENSMUSG00000033862
Cdk11b	-0.859447399	0.19753424	1.36E-05	1.89E-04	ENSMUSG00000029062
Cdk13	-0.824292649	0.20826697	7.56E-05	8.02E-04	ENSMUSG00000041297
Cdk17	-0.812585037	0.28429324	4.26E-03	2.10E-02	ENSMUSG00000020015
Cdk2ap2	-0.600311104	0.1265163	2.09E-06	3.85E-05	ENSMUSG00000024856
Cdk5	0.790915114	0.15636215	4.23E-07	9.46E-06	ENSMUSG00000028969

Cdk5rap2	-1.338747175	0.40673176	9.97E-04	6.58E-03	ENSMUSG00000039298
Cdk7	-0.686378783	0.19399424	4.03E-04	3.14E-03	ENSMUSG00000069089
Cdkn2a	0.518322415	0.17284841	2.71E-03	1.46E-02	ENSMUSG00000044303
Cdr2	-0.949745929	0.20392247	3.20E-06	5.54E-05	ENSMUSG00000030878
Cdsn	1.245298381	0.1446272	7.28E-18	1.09E-15	ENSMUSG00000039518
Cdt1	-0.995588369	0.21261273	2.83E-06	5.00E-05	ENSMUSG00000006585
Cdv3	-0.741629334	0.26651454	5.39E-03	2.54E-02	ENSMUSG00000032803
Cdyl	-0.556148098	0.21000737	8.09E-03	3.48E-02	ENSMUSG00000059288
Cebpa	1.016022342	0.22591067	6.88E-06	1.06E-04	ENSMUSG00000034957
Cebpd	-0.436054381	0.12635915	5.59E-04	4.10E-03	ENSMUSG00000071637
Cebpg	-0.748292695	0.13928234	7.77E-08	2.13E-06	ENSMUSG00000056216
Cebpz	-1.175226218	0.27265642	1.63E-05	2.22E-04	ENSMUSG00000024081
Cela1	0.87667373	0.24148564	2.83E-04	2.34E-03	ENSMUSG00000023031
Celf4	-1.582205836	0.38614835	4.18E-05	4.94E-04	ENSMUSG00000024268
Cenpb	0.526874262	0.10832914	1.15E-06	2.28E-05	ENSMUSG00000068267
Cenpt	1.3132843	0.4307336	2.30E-03	1.28E-02	ENSMUSG00000036672
Cenpx	0.510585287	0.12258948	3.11E-05	3.83E-04	ENSMUSG00000025144
Cep350	-1.228619714	0.35156081	4.74E-04	3.59E-03	ENSMUSG00000033671
Cep57	-1.097854488	0.39311216	5.23E-03	2.48E-02	ENSMUSG00000031922
Cep83	-1.687605796	0.52127803	1.21E-03	7.73E-03	ENSMUSG00000020024
Cercam	0.848947444	0.19953656	2.09E-05	2.74E-04	ENSMUSG00000039787
Cerk	0.820117096	0.21606316	1.47E-04	1.37E-03	ENSMUSG00000035891
Cers5	0.610908739	0.15687096	9.85E-05	9.91E-04	ENSMUSG00000023021
Cetn2	0.525584112	0.16350433	1.31E-03	8.20E-03	ENSMUSG00000031347
Cetn3	-0.532930285	0.17497549	2.32E-03	1.29E-02	ENSMUSG00000021537
Cfdp1	-0.475713618	0.1343401	3.98E-04	3.11E-03	ENSMUSG00000031954
Cfl2	-0.658521847	0.12037273	4.48E-08	1.31E-06	ENSMUSG00000062929
Cgas	-0.912171686	0.32625055	5.18E-03	2.46E-02	ENSMUSG00000032344
Cggbp1	-0.524446938	0.16479401	1.46E-03	8.92E-03	ENSMUSG00000054604
Chad	0.744266633	0.24059044	1.98E-03	1.13E-02	ENSMUSG00000039084
Chchd1	-0.441725739	0.13770225	1.34E-03	8.33E-03	ENSMUSG00000063787
Chchd10	-1.054708557	0.13414301	3.76E-15	4.06E-13	ENSMUSG00000049422
Chchd3	-0.3560283	0.14273256	1.26E-02	4.91E-02	ENSMUSG00000053768
Chd1	-1.192524146	0.26900715	9.29E-06	1.35E-04	ENSMUSG00000023852
Chd2	-0.90067751	0.25237039	3.59E-04	2.85E-03	ENSMUSG00000078671
Chd7	-1.833821754	0.3000001	9.79E-10	4.43E-08	ENSMUSG00000041235
Chic1	-1.035511171	0.36678328	4.75E-03	2.30E-02	ENSMUSG00000031327
Chic2	-0.36644129	0.13865355	8.22E-03	3.53E-02	ENSMUSG00000029229
Chid1	0.381317761	0.15187182	1.20E-02	4.75E-02	ENSMUSG00000025512
Chil1	-1.842256116	0.4379909	2.60E-05	3.28E-04	ENSMUSG00000064246
Chka	-0.550451625	0.19625884	5.04E-03	2.41E-02	ENSMUSG00000024843
Chm	-0.93154874	0.28659782	1.15E-03	7.44E-03	ENSMUSG00000025531
Chmp1b	-0.385184258	0.12929465	2.89E-03	1.54E-02	ENSMUSG00000109511

Chmp2b	-0.64746172	0.15090692	1.78E-05	2.39E-04	ENSMUSG00000004843
Chmp5	-0.348598818	0.12796923	6.45E-03	2.92E-02	ENSMUSG00000028419
Chordc1	-0.853020486	0.18097846	2.44E-06	4.39E-05	ENSMUSG00000001774
Chpf	0.547028396	0.18747393	3.52E-03	1.81E-02	ENSMUSG00000032997
Chpf2	0.606625421	0.16135145	1.70E-04	1.54E-03	ENSMUSG00000038181
Chst12	0.605035774	0.14608598	3.45E-05	4.17E-04	ENSMUSG00000036599
Chst14	0.591461935	0.17791628	8.86E-04	6.02E-03	ENSMUSG00000074916
Churc1	1.023177994	0.30512984	7.99E-04	5.54E-03	ENSMUSG00000090258
Cip2a	-1.764139469	0.63038361	5.13E-03	2.44E-02	ENSMUSG00000033031
Cir1	-1.414924235	0.41513944	6.54E-04	4.68E-03	ENSMUSG00000041777
Cirbp	1.107456814	0.18574064	2.49E-09	1.01E-07	ENSMUSG00000045193
Cisd3	0.878059349	0.29370447	2.79E-03	1.50E-02	ENSMUSG00000078695
Ckap4	0.538604271	0.11459957	2.60E-06	4.65E-05	ENSMUSG00000046841
Ckb	1.479464033	0.23453289	2.82E-10	1.47E-08	ENSMUSG00000001270
Clcn3	-1.094677456	0.26514359	3.65E-05	4.37E-04	ENSMUSG00000004319
Cldn1	2.021600548	0.46105459	1.16E-05	1.65E-04	ENSMUSG00000022512
Cldn10	0.890343786	0.16915694	1.41E-07	3.59E-06	ENSMUSG00000022132
Cldn2	2.271299994	0.80027195	4.54E-03	2.21E-02	ENSMUSG00000047230
Clec11a	1.316066052	0.14487996	1.05E-19	1.94E-17	ENSMUSG00000004473
Clec14a	2.041843613	0.77015039	8.02E-03	3.47E-02	ENSMUSG00000045930
Clec16a	1.473885678	0.45667713	1.25E-03	7.93E-03	ENSMUSG00000068663
Clec4a1	1.112170585	0.41241977	7.00E-03	3.13E-02	ENSMUSG00000049037
Clec4d	-0.911631549	0.19928344	4.77E-06	7.79E-05	ENSMUSG00000030144
Clec4e	-2.460372382	0.60204278	4.38E-05	5.13E-04	ENSMUSG00000030142
Clip1	-0.676273094	0.23824605	4.53E-03	2.21E-02	ENSMUSG00000049550
Clip3	0.441540746	0.13740877	1.31E-03	8.22E-03	ENSMUSG00000013921
Clk1	-0.747162274	0.25523786	3.42E-03	1.76E-02	ENSMUSG00000026034
Clk4	-0.970159992	0.24853651	9.48E-05	9.64E-04	ENSMUSG00000020385
Clmp	-0.358126422	0.12815289	5.20E-03	2.47E-02	ENSMUSG00000032024
Clock	-1.169962151	0.29950845	9.37E-05	9.54E-04	ENSMUSG00000029238
Clptm1	0.412098133	0.13236644	1.85E-03	1.08E-02	ENSMUSG00000002981
Clstn1	0.826891313	0.18450529	7.41E-06	1.13E-04	ENSMUSG00000039953
Cltb	0.520410132	0.11390519	4.91E-06	7.95E-05	ENSMUSG00000047547
Cltc	-0.816945792	0.15229603	8.13E-08	2.21E-06	ENSMUSG00000047126
Clu	-0.770741416	0.17010859	5.87E-06	9.27E-05	ENSMUSG00000022037
Clybl	0.417742229	0.16045673	9.23E-03	3.87E-02	ENSMUSG00000025545
Cmc2	0.430059265	0.17282283	1.28E-02	4.98E-02	ENSMUSG00000014633
Cmklr1	1.98630403	0.32721806	1.28E-09	5.57E-08	ENSMUSG00000042190
Cmtm3	0.519074557	0.1416076	2.47E-04	2.10E-03	ENSMUSG00000031875
Cmtm8	-0.619506046	0.24217676	1.05E-02	4.28E-02	ENSMUSG00000041012
Cnbp	-0.580307147	0.12263602	2.22E-06	4.07E-05	ENSMUSG00000030057
Cnih1	-0.259252716	0.10415495	1.28E-02	4.97E-02	ENSMUSG00000015759
Cnn2	1.000291524	0.12595958	2.00E-15	2.25E-13	ENSMUSG00000004665

Cnot6	-0.932238413	0.20819659	7.55E-06	1.15E-04	ENSMUSG00000020362
Cnot6l	-1.085135162	0.23174707	2.84E-06	5.00E-05	ENSMUSG00000034724
Cnot7	-0.80926142	0.20384395	7.19E-05	7.73E-04	ENSMUSG00000031601
Cnp	1.461525128	0.23792977	8.11E-10	3.76E-08	ENSMUSG00000006782
Cnppd1	0.404754873	0.13917153	3.63E-03	1.85E-02	ENSMUSG00000033159
Cnpy2	0.679431375	0.12039181	1.67E-08	5.53E-07	ENSMUSG00000025381
Cntnap1	2.126917116	0.81286469	8.88E-03	3.75E-02	ENSMUSG00000017167
Coa3	0.388032413	0.11681944	8.95E-04	6.07E-03	ENSMUSG00000017188
Cog1	0.711513982	0.26501515	7.26E-03	3.21E-02	ENSMUSG00000018661
Cog5	-0.946718938	0.36416883	9.33E-03	3.90E-02	ENSMUSG00000035933
Col13a1	4.179529271	0.58332819	7.78E-13	6.44E-11	ENSMUSG00000058806
Col16a1	0.613468721	0.1776017	5.52E-04	4.07E-03	ENSMUSG00000040690
Col1a1	0.926178598	0.11037903	4.82E-17	6.55E-15	ENSMUSG00000001506
Col1a2	0.873752356	0.11247272	7.94E-15	8.36E-13	ENSMUSG00000029661
Col22a1	1.041286061	0.34115322	2.27E-03	1.27E-02	ENSMUSG00000079022
Col27a1	0.56180362	0.15571394	3.09E-04	2.52E-03	ENSMUSG00000045672
Col2a1	0.518287371	0.13746852	1.63E-04	1.49E-03	ENSMUSG00000022483
Col3a1	0.722320394	0.13265668	5.18E-08	1.49E-06	ENSMUSG00000026043
Col4a1	0.927174318	0.13465176	5.75E-12	4.18E-10	ENSMUSG00000031502
Col4a2	0.838761068	0.14749885	1.30E-08	4.48E-07	ENSMUSG00000031503
Col5a1	1.285257246	0.11592663	1.45E-28	7.06E-26	ENSMUSG00000026837
Col5a2	0.52767595	0.10052805	1.53E-07	3.84E-06	ENSMUSG00000026042
Col6a2	0.691056743	0.16053177	1.67E-05	2.26E-04	ENSMUSG00000020241
Col6a3	0.713202742	0.13740395	2.10E-07	5.06E-06	ENSMUSG00000048126
Col8a2	1.34828467	0.18749975	6.44E-13	5.43E-11	ENSMUSG00000056174
Col9a1	-0.526253721	0.1751098	2.65E-03	1.44E-02	ENSMUSG00000026147
Comp	0.641947041	0.18973851	7.16E-04	5.05E-03	ENSMUSG00000031849
Cop1	-1.035604437	0.22676374	4.95E-06	8.02E-05	ENSMUSG00000040782
Copb1	-0.627996836	0.25040885	1.21E-02	4.78E-02	ENSMUSG00000030754
Copb2	-0.572208283	0.12572212	5.33E-06	8.50E-05	ENSMUSG00000032458
Cops2	-1.148202527	0.19745603	6.06E-09	2.23E-07	ENSMUSG00000027206
Cops4	-0.440929399	0.15365457	4.11E-03	2.04E-02	ENSMUSG00000035297
Cops7a	0.543942657	0.21777096	1.25E-02	4.88E-02	ENSMUSG00000030127
Copz1	-0.314263468	0.10660932	3.20E-03	1.67E-02	ENSMUSG00000060992
Copz2	0.562929104	0.16659791	7.28E-04	5.12E-03	ENSMUSG00000018672
Coq10b	-1.061829229	0.26387061	5.72E-05	6.40E-04	ENSMUSG00000025981
Coq5	-0.895697198	0.24642234	2.78E-04	2.31E-03	ENSMUSG00000041733
Coq8a	1.000568665	0.38233943	8.87E-03	3.75E-02	ENSMUSG00000026489
Coro1c	0.461708697	0.18157935	1.10E-02	4.43E-02	ENSMUSG00000004530
Cotl1	0.579246002	0.15863765	2.61E-04	2.19E-03	ENSMUSG00000031827
Cox15	0.510873383	0.16359574	1.79E-03	1.05E-02	ENSMUSG00000040018
Cox20	0.760147613	0.12182096	4.38E-10	2.18E-08	ENSMUSG00000026500
Cox5b	0.534959714	0.1832375	3.51E-03	1.80E-02	ENSMUSG00000061518

Cox7a1	1.042981616	0.29038767	3.29E-04	2.66E-03	ENSMUSG00000074218
Cox7a2l	-0.640633047	0.10917725	4.42E-09	1.68E-07	ENSMUSG00000024248
Cpa6	1.839244843	0.39139577	2.61E-06	4.66E-05	ENSMUSG00000042501
Cpne2	-0.416967495	0.14447117	3.90E-03	1.96E-02	ENSMUSG00000034361
Cpox	-0.669034895	0.18674953	3.40E-04	2.73E-03	ENSMUSG00000022742
Cpq	1.072465906	0.16246188	4.07E-11	2.62E-09	ENSMUSG00000039007
Cpsf6	-0.614617191	0.18497775	8.92E-04	6.06E-03	ENSMUSG00000055531
Cpt1c	0.887647591	0.21425255	3.43E-05	4.15E-04	ENSMUSG00000007783
Cptp	0.94153718	0.28531096	9.67E-04	6.44E-03	ENSMUSG00000029073
Cr1l	0.539055075	0.13677154	8.10E-05	8.49E-04	ENSMUSG00000016481
Crat	0.710955931	0.20521047	5.31E-04	3.94E-03	ENSMUSG00000026853
Crcp	-0.512176893	0.1897223	6.94E-03	3.11E-02	ENSMUSG00000025532
Creb1	-0.574996322	0.18171228	1.55E-03	9.37E-03	ENSMUSG00000025958
Creb3l1	1.096894428	0.12207472	2.58E-19	4.58E-17	ENSMUSG00000027230
Crebrf	-0.761700597	0.18906251	5.61E-05	6.30E-04	ENSMUSG00000048249
Cript	-0.496051326	0.15698434	1.58E-03	9.49E-03	ENSMUSG00000024146
Crispld2	0.658898617	0.14120952	3.07E-06	5.33E-05	ENSMUSG00000031825
Crls1	-0.750957647	0.16055963	2.91E-06	5.10E-05	ENSMUSG00000027357
Crnkl1	-1.148233703	0.32427832	3.99E-04	3.12E-03	ENSMUSG00000001767
Crtap	0.753366301	0.20745571	2.82E-04	2.33E-03	ENSMUSG00000032431
Cryab	-0.445406199	0.15142223	3.27E-03	1.70E-02	ENSMUSG00000032060
Cryba4	2.896916128	0.5435477	9.84E-08	2.59E-06	ENSMUSG00000066975
Crybb1	2.905165935	0.42826165	1.17E-11	8.16E-10	ENSMUSG00000029343
Cryl1	0.910817737	0.34208798	7.76E-03	3.38E-02	ENSMUSG00000021947
Cryz	0.856843798	0.19365777	9.67E-06	1.40E-04	ENSMUSG00000028199
Cryzl1	-0.461550842	0.17663739	8.98E-03	3.78E-02	ENSMUSG00000058240
Csad	0.54790078	0.14923858	2.41E-04	2.06E-03	ENSMUSG00000023044
Csde1	-0.9748679	0.18555867	1.49E-07	3.77E-06	ENSMUSG00000068823
Cse1l	-0.801482692	0.29946022	7.44E-03	3.28E-02	ENSMUSG00000002718
Csf1r	0.951404924	0.30179817	1.62E-03	9.69E-03	ENSMUSG00000024621
Csf2ra	0.441003933	0.15421083	4.24E-03	2.10E-02	ENSMUSG00000059326
Csgalnact1	-0.79247479	0.26449152	2.73E-03	1.47E-02	ENSMUSG00000036356
Csgalnact2	-1.019172031	0.22075279	3.90E-06	6.54E-05	ENSMUSG00000042042
Csnk1a1	-0.399971555	0.12111065	9.58E-04	6.39E-03	ENSMUSG00000024576
Csnk1e	-0.57635348	0.16717348	5.66E-04	4.15E-03	ENSMUSG00000022433
Csnk2a1	-0.472597471	0.15734097	2.67E-03	1.45E-02	ENSMUSG00000074698
Cspg4	1.187834193	0.24308948	1.03E-06	2.08E-05	ENSMUSG00000032911
Cst3	0.487911649	0.1012198	1.43E-06	2.76E-05	ENSMUSG00000027447
Ctbp1	0.385009347	0.13743782	5.09E-03	2.43E-02	ENSMUSG00000037373
Ctdsp1	0.730467164	0.19281679	1.52E-04	1.40E-03	ENSMUSG00000026176
Ctdsp2	0.391109508	0.11812824	9.30E-04	6.27E-03	ENSMUSG00000078429
Ctdspl	0.450794803	0.17781085	1.12E-02	4.50E-02	ENSMUSG00000047409
Ctf1	2.321013971	0.74632808	1.87E-03	1.09E-02	ENSMUSG00000042340

Cth	-0.841732925	0.24000806	4.53E-04	3.46E-03	ENSMUSG00000028179
Cthrc1	0.671305087	0.24168398	5.48E-03	2.57E-02	ENSMUSG00000054196
Ctla2a	-0.421898506	0.1217141	5.28E-04	3.92E-03	ENSMUSG00000044258
Ctnnbip1	0.599744362	0.13891685	1.58E-05	2.16E-04	ENSMUSG00000028988
Ctnnd1	0.705457835	0.17227276	4.22E-05	4.98E-04	ENSMUSG00000034101
Ctps	-0.57550199	0.16886216	6.54E-04	4.68E-03	ENSMUSG00000028633
Ctsa	0.519646658	0.10160892	3.15E-07	7.24E-06	ENSMUSG00000017760
Ctsd	0.555764302	0.11531767	1.44E-06	2.77E-05	ENSMUSG00000007891
Ctsk	0.716087574	0.1158157	6.29E-10	3.02E-08	ENSMUSG00000028111
Ctsl	-0.426912127	0.11526139	2.12E-04	1.85E-03	ENSMUSG00000021477
Ctss	-0.665254313	0.22031903	2.53E-03	1.39E-02	ENSMUSG00000038642
Cuedc1	0.567263613	0.19441838	3.53E-03	1.81E-02	ENSMUSG00000018378
Cuedc2	0.560763308	0.14401075	9.86E-05	9.92E-04	ENSMUSG00000036748
Cul2	-1.172654405	0.40308964	3.62E-03	1.85E-02	ENSMUSG00000024231
Cul3	-1.131992531	0.21234863	9.78E-08	2.58E-06	ENSMUSG00000004364
Cul4b	-1.424105623	0.29371374	1.24E-06	2.44E-05	ENSMUSG00000031095
Cul5	-0.986978325	0.26347626	1.80E-04	1.61E-03	ENSMUSG00000032030
Cuta	0.487724698	0.136509	3.53E-04	2.81E-03	ENSMUSG00000024194
Cwc15	-0.312975108	0.12260717	1.07E-02	4.34E-02	ENSMUSG00000004096
Cwc27	-1.263671267	0.47703485	8.07E-03	3.48E-02	ENSMUSG00000021715
Cwf19l2	-1.049014951	0.21989077	1.84E-06	3.46E-05	ENSMUSG00000025898
Cx3cr1	-0.834230574	0.26406342	1.58E-03	9.50E-03	ENSMUSG00000052336
Cxadr	-2.423597593	0.74871583	1.21E-03	7.74E-03	ENSMUSG00000022865
Cxcl1	-2.578071417	0.3614838	9.90E-13	7.99E-11	ENSMUSG00000029380
Cxcl12	1.798047335	0.18650646	5.38E-22	1.29E-19	ENSMUSG00000061353
Cxcl14	-0.679564504	0.13959877	1.13E-06	2.24E-05	ENSMUSG00000021508
Cxcl2	-1.8693768	0.52307042	3.52E-04	2.80E-03	ENSMUSG00000058427
Cxxc5	0.691857313	0.16943223	4.44E-05	5.19E-04	ENSMUSG00000046668
Cyb561	0.623734505	0.16239832	1.23E-04	1.18E-03	ENSMUSG00000019590
Cyb561d2	0.866115518	0.29268662	3.08E-03	1.62E-02	ENSMUSG00000037190
Cyb5rl	1.50431284	0.30913163	1.14E-06	2.25E-05	ENSMUSG00000028621
Cybc1	0.3915054	0.15564976	1.19E-02	4.71E-02	ENSMUSG00000039294
Cyc1	0.322566703	0.12807959	1.18E-02	4.67E-02	ENSMUSG00000022551
Cygb	1.021961821	0.16622549	7.84E-10	3.65E-08	ENSMUSG00000020810
Cyhr1	0.542812678	0.14231361	1.37E-04	1.29E-03	ENSMUSG00000053929
Cyp2d22	-0.56456324	0.17647003	1.38E-03	8.54E-03	ENSMUSG00000061740
Cyp2f2	1.663594968	0.20423035	3.77E-16	4.64E-14	ENSMUSG00000052974
Cyren	0.675583476	0.24194664	5.23E-03	2.48E-02	ENSMUSG00000046806
D16Ertd472e	-0.832756105	0.22165888	1.72E-04	1.56E-03	ENSMUSG00000022864
D17H6S53E	0.889367191	0.29144496	2.28E-03	1.27E-02	ENSMUSG00000043311
D2hgdh	1.207580951	0.46164671	8.90E-03	3.76E-02	ENSMUSG00000073609
D330023K18Rik	1.227883712	0.47617115	9.92E-03	4.09E-02	ENSMUSG00000087269
D830050J10Rik	-0.846009689	0.20130145	2.64E-05	3.33E-04	ENSMUSG00000107176

D930016D06Rik	-0.631623552	0.24263197	9.24E-03	3.87E-02	ENSMUSG00000097375
Daam1	-0.871500772	0.33196054	8.66E-03	3.68E-02	ENSMUSG00000034574
Daam2	0.761877549	0.29463622	9.71E-03	4.03E-02	ENSMUSG00000040260
Dab2ip	0.576145245	0.22934417	1.20E-02	4.74E-02	ENSMUSG00000026883
Dact3	0.621382694	0.24095567	9.91E-03	4.09E-02	ENSMUSG00000078794
Dag1	0.711954599	0.13502634	1.34E-07	3.44E-06	ENSMUSG00000039952
Dap	0.295605351	0.09902756	2.84E-03	1.51E-02	ENSMUSG00000039168
Dbf4	-1.257680999	0.38843586	1.20E-03	7.73E-03	ENSMUSG00000002297
Dbnl	0.8297724	0.13563995	9.51E-10	4.33E-08	ENSMUSG00000020476
Dbp	-0.491809841	0.12983406	1.52E-04	1.41E-03	ENSMUSG00000059824
Dcaf1	-1.622628236	0.53361714	2.36E-03	1.31E-02	ENSMUSG00000040325
Dcaf10	-0.73069903	0.24300101	2.64E-03	1.43E-02	ENSMUSG00000035572
Dcaf11	0.604548477	0.22172871	6.40E-03	2.91E-02	ENSMUSG00000022214
Dcaf13	-0.624173405	0.17112592	2.65E-04	2.22E-03	ENSMUSG00000022300
Dcaf4	0.672908639	0.20071778	8.01E-04	5.55E-03	ENSMUSG00000021222
Dcaf5	0.510174298	0.17757789	4.07E-03	2.02E-02	ENSMUSG00000049106
Dchs1	0.879026665	0.25073422	4.55E-04	3.47E-03	ENSMUSG00000036862
Dcn	-0.443058269	0.16009331	5.65E-03	2.64E-02	ENSMUSG00000019929
Dctn4	-0.338505873	0.1293511	8.87E-03	3.75E-02	ENSMUSG00000024603
Dctpp1	-0.347106743	0.13685591	1.12E-02	4.49E-02	ENSMUSG00000042462
Dcun1d1	-0.843965606	0.25055939	7.56E-04	5.30E-03	ENSMUSG00000027708
Dcxr	0.590135676	0.14362847	3.98E-05	4.72E-04	ENSMUSG00000039450
Ddi2	-0.536684716	0.15289759	4.48E-04	3.44E-03	ENSMUSG00000078515
Ddit3	-0.570889263	0.10760309	1.12E-07	2.93E-06	ENSMUSG00000025408
Ddit4	0.453813547	0.10311931	1.08E-05	1.55E-04	ENSMUSG00000020108
Ddit4l	0.452663614	0.11994199	1.61E-04	1.47E-03	ENSMUSG00000046818
Ddost	0.622263278	0.1158707	7.86E-08	2.14E-06	ENSMUSG00000028757
Ddr1	0.797423282	0.20590598	1.08E-04	1.06E-03	ENSMUSG00000003534
Ddx1	-0.675794982	0.18235046	2.11E-04	1.84E-03	ENSMUSG00000037149
Ddx10	-1.14737087	0.36582993	1.71E-03	1.01E-02	ENSMUSG00000053289
Ddx21	-0.951891051	0.20860102	5.04E-06	8.15E-05	ENSMUSG00000020075
Ddx3x	-0.710529939	0.18304177	1.04E-04	1.03E-03	ENSMUSG00000000787
Ddx3y	-0.981847981	0.22276296	1.05E-05	1.51E-04	ENSMUSG00000069045
Ddx41	0.462830075	0.14458499	1.37E-03	8.50E-03	ENSMUSG00000021494
Ddx46	-0.750182081	0.22811183	1.01E-03	6.64E-03	ENSMUSG00000021500
Ddx50	-0.96263096	0.21374533	6.68E-06	1.03E-04	ENSMUSG00000020076
Ddx52	-1.086859188	0.26745234	4.83E-05	5.56E-04	ENSMUSG00000020677
Decr1	0.45227065	0.12854668	4.34E-04	3.34E-03	ENSMUSG00000028223
Def8	0.529820771	0.13725815	1.13E-04	1.11E-03	ENSMUSG00000001482
Dek	-0.781051783	0.24053929	1.17E-03	7.52E-03	ENSMUSG00000021377
Dennd1a	0.815501261	0.219121	1.98E-04	1.75E-03	ENSMUSG00000035392
Dennd1b	-1.146017504	0.41693664	5.98E-03	2.76E-02	ENSMUSG00000056268
Dennd4a	-0.801058296	0.31413009	1.08E-02	4.37E-02	ENSMUSG00000053641

Denr	-0.772775132	0.12561788	7.66E-10	3.58E-08	ENSMUSG00000023106
Derl2	0.4384845	0.14186433	2.00E-03	1.14E-02	ENSMUSG00000018442
Dexi	0.600472795	0.23397858	1.03E-02	4.21E-02	ENSMUSG00000038055
Dgat1	0.512442473	0.19357367	8.11E-03	3.49E-02	ENSMUSG00000022555
Dgat2	1.75004446	0.56279189	1.87E-03	1.09E-02	ENSMUSG00000030747
Dgcr2	0.600983706	0.16769067	3.39E-04	2.73E-03	ENSMUSG00000003166
Dgkh	-0.927390759	0.26332593	4.29E-04	3.31E-03	ENSMUSG00000034731
Dgkz	0.698221325	0.23080715	2.49E-03	1.36E-02	ENSMUSG00000040479
Dhcr24	0.64514025	0.23649379	6.37E-03	2.90E-02	ENSMUSG00000034926
Dhcr7	0.926228303	0.15305082	1.43E-09	6.18E-08	ENSMUSG00000058454
Dhrs1	1.294328555	0.15489787	6.49E-17	8.54E-15	ENSMUSG00000002332
Dhrs3	0.745336855	0.12711615	4.53E-09	1.72E-07	ENSMUSG00000066026
Dhrs4	1.112671978	0.18209043	9.93E-10	4.48E-08	ENSMUSG00000022210
Dhrs9	-1.395171677	0.3914629	3.65E-04	2.89E-03	ENSMUSG00000027068
Dhx15	-0.604359983	0.1730922	4.80E-04	3.62E-03	ENSMUSG00000029169
Dhx36	-1.104513567	0.26537941	3.15E-05	3.87E-04	ENSMUSG00000027770
Dhx40	-0.610759818	0.21567875	4.63E-03	2.25E-02	ENSMUSG00000018425
Dhx58	1.572521343	0.60393974	9.22E-03	3.86E-02	ENSMUSG00000017830
Dhx58os	1.730478702	0.31436599	3.70E-08	1.11E-06	ENSMUSG00000085604
Dio3	2.388593118	0.18639292	1.35E-37	1.37E-34	ENSMUSG00000075707
Dio3os	-1.293462114	0.46246564	5.16E-03	2.45E-02	ENSMUSG00000112843
Dipk1b	0.619572125	0.1524857	4.84E-05	5.57E-04	ENSMUSG00000036186
Dipk1c	2.679390092	1.06584954	1.19E-02	4.72E-02	ENSMUSG00000047992
Dipk2a	0.724123841	0.23794674	2.34E-03	1.30E-02	ENSMUSG00000045414
Dis3	-0.985436191	0.30527303	1.25E-03	7.93E-03	ENSMUSG00000033166
Dkc1	-1.208168631	0.22493915	7.83E-08	2.14E-06	ENSMUSG00000031403
Dkk3	0.950385149	0.20312545	2.89E-06	5.06E-05	ENSMUSG00000030772
Dld	-0.793222922	0.2621462	2.48E-03	1.36E-02	ENSMUSG00000020664
Dlg1	-0.663080754	0.20478407	1.20E-03	7.73E-03	ENSMUSG00000022770
Dlg5	0.472697644	0.16139558	3.40E-03	1.76E-02	ENSMUSG00000021782
Dlgap4	0.515867349	0.13385915	1.16E-04	1.13E-03	ENSMUSG00000061689
Dlk2	3.021880041	0.55030845	3.99E-08	1.19E-06	ENSMUSG00000047428
Dlx3	2.756792994	0.65203217	2.36E-05	3.02E-04	ENSMUSG00000001510
Dlx5	1.576283601	0.25230747	4.17E-10	2.10E-08	ENSMUSG00000029755
Dmac1	0.395399538	0.12420206	1.45E-03	8.89E-03	ENSMUSG00000028398
Dmpk	1.545231878	0.16141741	1.04E-21	2.36E-19	ENSMUSG00000030409
Dmtf1	-0.973873568	0.31082364	1.73E-03	1.02E-02	ENSMUSG00000042508
Dmwd	0.48998797	0.19399759	1.15E-02	4.59E-02	ENSMUSG00000030410
Dnaja2	-0.54805173	0.13525427	5.08E-05	5.79E-04	ENSMUSG00000031701
Dnajb4	-0.765147703	0.24811298	2.04E-03	1.16E-02	ENSMUSG00000028035
Dnajb5	1.536544641	0.55529541	5.66E-03	2.64E-02	ENSMUSG00000036052
Dnajb6	-0.437051892	0.15985149	6.25E-03	2.85E-02	ENSMUSG00000029131
Dnajc1	-0.495470116	0.17446777	4.51E-03	2.20E-02	ENSMUSG00000026740

Dnajc12	1.517430052	0.46114378	1.00E-03	6.60E-03	ENSMUSG00000036764
Dnajc2	-1.227220461	0.25178032	1.09E-06	2.18E-05	ENSMUSG00000029014
Dnajc30	0.676266551	0.20383835	9.08E-04	6.15E-03	ENSMUSG00000061118
Dnajc8	-0.430552659	0.11189998	1.19E-04	1.16E-03	ENSMUSG00000054405
Dnase1l1	1.085379249	0.36758869	3.15E-03	1.65E-02	ENSMUSG00000019088
Dnase2a	0.796040215	0.24238433	1.02E-03	6.71E-03	ENSMUSG00000003812
Dnlz	0.539453211	0.13122535	3.94E-05	4.68E-04	ENSMUSG00000075467
Dnm1l	-1.063968006	0.34272317	1.91E-03	1.10E-02	ENSMUSG00000022789
Dnpep	0.518312796	0.14301319	2.90E-04	2.39E-03	ENSMUSG00000026209
Dnttip2	-1.427006266	0.31045369	4.30E-06	7.12E-05	ENSMUSG00000039756
Dock10	-1.653867243	0.4392987	1.67E-04	1.52E-03	ENSMUSG00000038608
Dock6	0.872455011	0.27796283	1.70E-03	1.01E-02	ENSMUSG00000032198
Dok5	1.084896578	0.3309645	1.05E-03	6.85E-03	ENSMUSG00000027560
Dolk	0.963893013	0.25830762	1.90E-04	1.69E-03	ENSMUSG00000075419
Dolpp1	1.202583397	0.26467046	5.53E-06	8.78E-05	ENSMUSG00000026856
Dpep1	1.093413913	0.11316245	4.36E-22	1.06E-19	ENSMUSG00000019278
Dpf1	1.60948067	0.59948616	7.26E-03	3.21E-02	ENSMUSG00000030584
Dpp3	0.563723978	0.1313116	1.76E-05	2.37E-04	ENSMUSG00000063904
Dpp7	0.661603894	0.16206681	4.46E-05	5.21E-04	ENSMUSG00000026958
Dst	-0.89202535	0.23401366	1.38E-04	1.30E-03	ENSMUSG00000026131
Dtwd2	1.66625232	0.61262368	6.53E-03	2.95E-02	ENSMUSG00000024505
Dtx2	0.741164688	0.25399051	3.52E-03	1.81E-02	ENSMUSG00000004947
Dus4l	-1.925023828	0.76187616	1.15E-02	4.58E-02	ENSMUSG00000020648
Dusp1	0.357117127	0.1407274	1.12E-02	4.48E-02	ENSMUSG00000024190
Dusp11	-0.602287244	0.19176483	1.69E-03	1.00E-02	ENSMUSG00000030002
Dusp12	-0.624482811	0.17611184	3.91E-04	3.06E-03	ENSMUSG00000026659
Dusp14	0.865378623	0.20448332	2.32E-05	2.97E-04	ENSMUSG00000018648
Dusp23	0.684883943	0.25413029	7.04E-03	3.14E-02	ENSMUSG00000026544
Dusp4	-1.534119248	0.52416272	3.42E-03	1.76E-02	ENSMUSG00000031530
Dusp6	-0.536618242	0.18213384	3.22E-03	1.68E-02	ENSMUSG00000019960
Dut	-1.242289564	0.14419616	6.98E-18	1.06E-15	ENSMUSG00000027203
Dync1i1	1.426879282	0.37661304	1.51E-04	1.40E-03	ENSMUSG00000029757
Dync1i2	-0.386054718	0.13653973	4.69E-03	2.28E-02	ENSMUSG00000027012
Dync2li1	0.750572974	0.25103684	2.79E-03	1.50E-02	ENSMUSG00000024253
Dynll1	0.684853793	0.14432104	2.08E-06	3.85E-05	ENSMUSG00000009013
Dynlrb2	2.807806788	0.84368672	8.75E-04	5.96E-03	ENSMUSG00000034467
Dysf	0.887335965	0.21084222	2.57E-05	3.25E-04	ENSMUSG00000033788
Dzip3	-1.330492732	0.45605901	3.53E-03	1.81E-02	ENSMUSG00000064061
Ebna1bp2	-0.504244081	0.12961593	1.00E-04	1.00E-03	ENSMUSG00000028729
Ebp	0.427405122	0.12435705	5.88E-04	4.29E-03	ENSMUSG00000031168
Ebpl	0.836247761	0.13110776	1.79E-10	9.70E-09	ENSMUSG00000021928
Ece1	0.478091832	0.15925119	2.68E-03	1.45E-02	ENSMUSG00000057530
Echdc1	-0.799425067	0.24850501	1.30E-03	8.15E-03	ENSMUSG00000019883

Edem1	0.441208011	0.16398562	7.13E-03	3.17E-02	ENSMUSG00000030104
Edem2	0.735685829	0.24608948	2.79E-03	1.50E-02	ENSMUSG00000038312
Eea1	-0.960526241	0.30073261	1.40E-03	8.65E-03	ENSMUSG00000036499
Eed	-0.810630347	0.21933388	2.19E-04	1.90E-03	ENSMUSG00000030619
Eef1akmt1	0.970097562	0.1479494	5.49E-11	3.39E-09	ENSMUSG00000021951
Eef1b2	-0.502093571	0.11219106	7.63E-06	1.16E-04	ENSMUSG00000025967
Eef1e1	-0.736625445	0.19275458	1.33E-04	1.26E-03	ENSMUSG00000001707
Eef1g	-0.408300566	0.13138387	1.89E-03	1.09E-02	ENSMUSG00000071644
Eepd1	1.160951008	0.28577465	4.86E-05	5.57E-04	ENSMUSG00000036611
Efemp2	0.549342779	0.13784962	6.75E-05	7.34E-04	ENSMUSG00000024909
Efna2	1.044464647	0.23554417	9.24E-06	1.35E-04	ENSMUSG00000003070
Efnb1	1.407602735	0.14500357	2.80E-22	7.10E-20	ENSMUSG00000031217
Efr3a	-0.608485364	0.20723959	3.32E-03	1.72E-02	ENSMUSG00000015002
Egln1	0.932989625	0.15774397	3.33E-09	1.31E-07	ENSMUSG00000031987
Egln2	0.640836759	0.18227032	4.38E-04	3.37E-03	ENSMUSG00000058709
Ehd2	0.860251485	0.14343558	2.00E-09	8.27E-08	ENSMUSG00000074364
Ehd3	1.098753894	0.1603653	7.30E-12	5.17E-10	ENSMUSG00000024065
Eid2b	0.912507298	0.28347094	1.29E-03	8.10E-03	ENSMUSG00000070705
Eif1	-0.442413786	0.12046096	2.40E-04	2.05E-03	ENSMUSG00000035530
Eif1a	-1.282653879	0.15633671	2.32E-16	2.93E-14	ENSMUSG00000057561
Eif1ad	-0.37755042	0.1442379	8.86E-03	3.75E-02	ENSMUSG00000024841
Eif1ax	-0.342025673	0.12147987	4.87E-03	2.35E-02	ENSMUSG00000067194
Eif1b	0.502805235	0.11916595	2.45E-05	3.12E-04	ENSMUSG00000006941
Eif2a	-1.059231627	0.26532787	6.55E-05	7.16E-04	ENSMUSG00000027810
Eif2ak4	-0.865833629	0.297312	3.59E-03	1.83E-02	ENSMUSG00000005102
Eif2b3	-0.583571936	0.20507516	4.43E-03	2.17E-02	ENSMUSG00000028683
Eif2s1	-0.772088715	0.17273947	7.83E-06	1.18E-04	ENSMUSG00000021116
Eif2s2	-1.400354296	0.42363264	9.48E-04	6.34E-03	ENSMUSG00000074656
Eif2s3y	-1.634796823	0.24449334	2.29E-11	1.50E-09	ENSMUSG00000069049
Eif3a	-1.397641572	0.41195194	6.92E-04	4.89E-03	ENSMUSG00000024991
Eif3c	-0.432115089	0.12123408	3.65E-04	2.89E-03	ENSMUSG00000030738
Eif3d	-0.360416472	0.11252027	1.36E-03	8.45E-03	ENSMUSG00000016554
Eif3e	-1.024185788	0.16463298	4.94E-10	2.44E-08	ENSMUSG00000022336
Eif3f	-0.397030423	0.1070903	2.09E-04	1.83E-03	ENSMUSG00000031029
Eif3g	-0.325479621	0.11631108	5.14E-03	2.44E-02	ENSMUSG00000070319
Eif3h	-0.47701311	0.12066725	7.71E-05	8.16E-04	ENSMUSG00000022312
Eif3m	-0.768933935	0.20145174	1.35E-04	1.28E-03	ENSMUSG00000027170
Eif4a2	-0.707568586	0.26478674	7.54E-03	3.31E-02	ENSMUSG00000022884
Eif4b	-0.508430603	0.1251353	4.84E-05	5.57E-04	ENSMUSG00000058655
Eif4e	-0.582224225	0.16003632	2.75E-04	2.29E-03	ENSMUSG00000028156
Eif4ebp1	-0.665637816	0.14080738	2.28E-06	4.16E-05	ENSMUSG00000031490
Eif4g2	-0.442911053	0.12336109	3.30E-04	2.67E-03	ENSMUSG00000005610
Eif5	-0.883500941	0.18619313	2.08E-06	3.85E-05	ENSMUSG00000021282

Eif5b	-1.292259734	0.45088924	4.16E-03	2.06E-02	ENSMUSG00000026083
Elk1	0.721299865	0.16029837	6.80E-06	1.05E-04	ENSMUSG00000009406
Ell2	-1.20248479	0.16426667	2.47E-13	2.23E-11	ENSMUSG00000001542
Eln	-0.95814797	0.3127319	2.19E-03	1.23E-02	ENSMUSG00000029675
Eloc	-0.564955731	0.13986305	5.36E-05	6.08E-04	ENSMUSG00000079658
Elof1	0.384123248	0.12541419	2.19E-03	1.23E-02	ENSMUSG00000013822
Elovl1	0.430161659	0.13267032	1.19E-03	7.63E-03	ENSMUSG00000006390
Elovl5	-0.384437967	0.13665379	4.90E-03	2.36E-02	ENSMUSG00000032349
Emb	-0.362804033	0.11392237	1.45E-03	8.88E-03	ENSMUSG00000021728
Emc10	0.855065666	0.14786154	7.34E-09	2.66E-07	ENSMUSG00000008140
Emc2	-0.59462494	0.20646152	3.98E-03	1.98E-02	ENSMUSG00000022337
Emc8	0.344957335	0.13409392	1.01E-02	4.15E-02	ENSMUSG00000031819
Emc9	0.904534544	0.31106324	3.64E-03	1.85E-02	ENSMUSG00000022217
Eme2	1.505708061	0.48304853	1.83E-03	1.07E-02	ENSMUSG00000073436
Emilin1	1.902793791	0.20434801	1.26E-20	2.48E-18	ENSMUSG00000029163
Emilin2	0.507133658	0.19206135	8.28E-03	3.55E-02	ENSMUSG00000024053
Eml3	0.827957028	0.16373749	4.27E-07	9.52E-06	ENSMUSG00000071647
Emp3	0.936244066	0.11347764	1.58E-16	2.02E-14	ENSMUSG00000040212
Emsy	-0.749172076	0.26802891	5.19E-03	2.46E-02	ENSMUSG00000035401
Endov	1.505211343	0.39482263	1.38E-04	1.30E-03	ENSMUSG00000039850
Eng	0.901538297	0.23487205	1.24E-04	1.19E-03	ENSMUSG00000026814
Engase	1.263726693	0.20894789	1.47E-09	6.31E-08	ENSMUSG00000033857
Enho	1.351789863	0.28873272	2.84E-06	5.00E-05	ENSMUSG00000028445
Eno1	0.899314704	0.26314669	6.32E-04	4.54E-03	ENSMUSG00000063524
Entpd3	1.834829978	0.44981846	4.52E-05	5.27E-04	ENSMUSG00000041608
Epb41	-0.807673348	0.29663728	6.47E-03	2.93E-02	ENSMUSG00000028906
Epb41l3	-0.681244314	0.23611001	3.91E-03	1.96E-02	ENSMUSG00000024044
Epb41l4aos	-1.365210806	0.13883581	8.09E-23	2.32E-20	ENSMUSG00000087590
Epha8	3.877720425	1.07081734	2.93E-04	2.41E-03	ENSMUSG00000028661
Ephb2	1.47842278	0.25874711	1.10E-08	3.89E-07	ENSMUSG00000028664
Ephb3	1.019041761	0.28447923	3.41E-04	2.74E-03	ENSMUSG00000005958
Ephb6	1.959044911	0.52976859	2.17E-04	1.89E-03	ENSMUSG00000029869
Ephx2	1.110026818	0.33889773	1.06E-03	6.90E-03	ENSMUSG00000022040
Epn2	-0.448438135	0.14811035	2.46E-03	1.36E-02	ENSMUSG00000001036
Eprs	-1.166656824	0.4267083	6.26E-03	2.85E-02	ENSMUSG00000026615
Ercc8	-0.929798145	0.29413651	1.57E-03	9.46E-03	ENSMUSG00000021694
Erg28	0.891116734	0.15089101	3.51E-09	1.36E-07	ENSMUSG00000021252
Ergic2	-0.68796187	0.21568346	1.42E-03	8.76E-03	ENSMUSG00000030304
Ergic3	0.482387661	0.10435359	3.79E-06	6.41E-05	ENSMUSG00000005881
Eri1	-0.571539014	0.19989129	4.25E-03	2.10E-02	ENSMUSG00000031527
Erlin2	0.868401134	0.27431703	1.55E-03	9.35E-03	ENSMUSG00000031483
Ern1	-0.71867351	0.18490442	1.02E-04	1.01E-03	ENSMUSG00000020715
Erp29	0.427894524	0.12650413	7.18E-04	5.06E-03	ENSMUSG00000029616

Erp44	-0.359459062	0.13356414	7.12E-03	3.17E-02	ENSMUSG00000028343
Esd	-0.962741844	0.11795867	3.30E-16	4.10E-14	ENSMUSG00000021996
Esf1	-1.628697168	0.25237717	1.09E-10	6.23E-09	ENSMUSG00000045624
Esyt1	0.45805782	0.17521707	8.94E-03	3.77E-02	ENSMUSG00000025366
Esyt2	-0.49708003	0.18206092	6.33E-03	2.88E-02	ENSMUSG00000021171
Etf1	-0.920925129	0.19643224	2.76E-06	4.90E-05	ENSMUSG00000024360
Etfb	0.688489908	0.11512938	2.23E-09	9.14E-08	ENSMUSG00000004610
Ets2	-0.422310851	0.13204822	1.38E-03	8.55E-03	ENSMUSG00000022895
Eva1b	1.05378177	0.10780866	1.45E-22	3.74E-20	ENSMUSG00000050212
Evc	0.448686032	0.17928754	1.23E-02	4.83E-02	ENSMUSG00000029122
Evi5l	1.112793706	0.25273776	1.07E-05	1.54E-04	ENSMUSG00000011832
Exd1	1.688452056	0.64410956	8.76E-03	3.71E-02	ENSMUSG00000048647
Exoc3l4	1.244658024	0.34304946	2.85E-04	2.36E-03	ENSMUSG00000021280
Exoc5	-0.971075118	0.22117458	1.13E-05	1.61E-04	ENSMUSG00000061244
Exoc6b	-0.911040121	0.27581407	9.56E-04	6.38E-03	ENSMUSG00000033769
Exoc7	0.736497483	0.18281058	5.61E-05	6.30E-04	ENSMUSG00000020792
Exoc8	0.779435624	0.28215165	5.74E-03	2.67E-02	ENSMUSG00000074030
Exosc1	-0.550379948	0.1457828	1.60E-04	1.46E-03	ENSMUSG00000034321
Exosc8	-0.821731149	0.14378264	1.10E-08	3.87E-07	ENSMUSG00000027752
Ext2	0.476618122	0.16561423	4.00E-03	2.00E-02	ENSMUSG00000027198
Extl3	1.044348038	0.19024483	4.03E-08	1.20E-06	ENSMUSG00000021978
Eya2	0.959045773	0.15673465	9.42E-10	4.31E-08	ENSMUSG00000017897
F5	-1.211752696	0.37750478	1.33E-03	8.29E-03	ENSMUSG00000026579
Fabp3	0.814222749	0.31378068	9.46E-03	3.94E-02	ENSMUSG00000028773
Faf1	-0.564990352	0.14335171	8.10E-05	8.49E-04	ENSMUSG00000010517
Fah	0.384692745	0.14237561	6.89E-03	3.09E-02	ENSMUSG00000030630
Fahd2a	1.35664289	0.36375272	1.92E-04	1.70E-03	ENSMUSG00000027371
Fam102a	0.98500737	0.19731542	5.97E-07	1.29E-05	ENSMUSG00000039157
Fam102b	-0.779808856	0.27477843	4.54E-03	2.21E-02	ENSMUSG00000040339
Fam111a	-1.32204069	0.37158431	3.74E-04	2.95E-03	ENSMUSG00000024691
Fam114a1	-0.792391177	0.12329044	1.30E-10	7.23E-09	ENSMUSG00000029185
Fam117a	0.866071514	0.29358798	3.18E-03	1.67E-02	ENSMUSG00000038893
Fam120aos	-1.343516142	0.18062771	1.02E-13	9.47E-12	ENSMUSG00000097039
Fam122a	0.638200193	0.24684277	9.73E-03	4.03E-02	ENSMUSG00000074922
Fam160a1	1.373135969	0.48632075	4.75E-03	2.30E-02	ENSMUSG00000051000
Fam162a	0.596185469	0.11939264	5.93E-07	1.28E-05	ENSMUSG00000003955
Fam173a	0.77736907	0.14292631	5.36E-08	1.53E-06	ENSMUSG00000057411
Fam185a	-0.888808142	0.28932828	2.13E-03	1.20E-02	ENSMUSG00000047221
Fam199x	-1.301451547	0.44043284	3.13E-03	1.64E-02	ENSMUSG00000042595
Fam204a	-1.560180224	0.40156963	1.02E-04	1.02E-03	ENSMUSG00000057858
Fam20a	1.18823072	0.24192826	9.04E-07	1.85E-05	ENSMUSG00000020614
Fam20b	0.616602725	0.23402586	8.42E-03	3.59E-02	ENSMUSG00000033557
Fam214b	0.513584362	0.19312521	7.83E-03	3.40E-02	ENSMUSG00000036002

Fam220a	0.443908442	0.17290118	1.02E-02	4.20E-02	ENSMUSG00000083012
Fam229b	0.671199038	0.26020698	9.89E-03	4.08E-02	ENSMUSG00000051736
Fam234a	0.724916502	0.15351984	2.34E-06	4.24E-05	ENSMUSG00000024187
Fam3a	0.724582835	0.17337922	2.93E-05	3.62E-04	ENSMUSG00000031399
Fam53b	0.893363767	0.21574712	3.46E-05	4.18E-04	ENSMUSG00000030956
Fam57a	0.769489218	0.29188978	8.38E-03	3.58E-02	ENSMUSG00000069808
Fam71f2	-2.062021195	0.61036622	7.29E-04	5.13E-03	ENSMUSG00000079652
Fam76b	-1.117168907	0.33780109	9.42E-04	6.32E-03	ENSMUSG00000037808
Fam8a1	-1.211246235	0.19500229	5.25E-10	2.57E-08	ENSMUSG00000069237
Fancc	1.583924987	0.1821329	3.42E-18	5.49E-16	ENSMUSG00000021461
Fap	0.627481773	0.20456933	2.16E-03	1.22E-02	ENSMUSG00000000392
Fasn	0.841273119	0.18723823	7.02E-06	1.08E-04	ENSMUSG00000025153
Fastkd2	-1.319245615	0.44425836	2.98E-03	1.58E-02	ENSMUSG00000025962
Fat1	0.908687967	0.206523	1.08E-05	1.55E-04	ENSMUSG00000070047
Fau	-0.559916963	0.19674714	4.43E-03	2.17E-02	ENSMUSG00000038274
Fbln2	0.551499677	0.13995016	8.12E-05	8.50E-04	ENSMUSG00000064080
Fbln7	1.005896819	0.22050425	5.07E-06	8.18E-05	ENSMUSG00000027386
Fbxl2	0.755757449	0.29847519	1.13E-02	4.53E-02	ENSMUSG00000032507
Fbxl5	-0.826121268	0.1678803	8.61E-07	1.78E-05	ENSMUSG00000039753
Fbxo2	0.71623565	0.24669149	3.69E-03	1.87E-02	ENSMUSG00000041556
Fbxo3	-0.437691252	0.14641678	2.80E-03	1.50E-02	ENSMUSG00000027180
Fbxo30	-1.115405574	0.2825531	7.89E-05	8.33E-04	ENSMUSG00000047648
Fbxo32	-0.824690651	0.32349208	1.08E-02	4.37E-02	ENSMUSG00000022358
Fbxo44	0.72341369	0.25001318	3.81E-03	1.92E-02	ENSMUSG00000029001
Fbxo46	0.618042012	0.23956195	9.88E-03	4.08E-02	ENSMUSG00000050428
Fbxw5	0.702445749	0.22917193	2.18E-03	1.23E-02	ENSMUSG00000015095
Fbxw8	0.435707758	0.15040314	3.77E-03	1.90E-02	ENSMUSG00000032867
Fbxw9	0.804238763	0.23010863	4.74E-04	3.59E-03	ENSMUSG00000008167
Fcf1	-0.813664503	0.14357673	1.45E-08	4.93E-07	ENSMUSG00000021243
Fcgrt	0.615238262	0.11195583	3.90E-08	1.17E-06	ENSMUSG00000003420
Fcor	1.533733902	0.52581454	3.54E-03	1.81E-02	ENSMUSG00000089665
Fdx1	-0.6295764	0.14588218	1.59E-05	2.17E-04	ENSMUSG00000032051
Fdxr	1.195072092	0.1913679	4.24E-10	2.12E-08	ENSMUSG00000018861
Fem1b	-0.789858197	0.30510566	9.63E-03	4.00E-02	ENSMUSG00000032244
Fem1c	-1.069464137	0.34342593	1.85E-03	1.08E-02	ENSMUSG00000033319
Fgd3	0.597346179	0.18553531	1.28E-03	8.09E-03	ENSMUSG00000037946
Fgf21	-1.9271031	0.54024991	3.61E-04	2.87E-03	ENSMUSG00000030827
Fgfr1	0.509676009	0.10788117	2.31E-06	4.21E-05	ENSMUSG00000031565
Fgfr1op	-0.795712351	0.31381296	1.12E-02	4.50E-02	ENSMUSG00000069135
Fgfr2	2.288143337	0.18355947	1.15E-35	8.93E-33	ENSMUSG00000030849
Fgfr3	2.197750295	0.2104227	1.55E-25	5.53E-23	ENSMUSG00000054252
Fgfrl1	0.470863153	0.11883881	7.43E-05	7.94E-04	ENSMUSG00000008090
Fhl1	0.588261707	0.17991621	1.08E-03	7.02E-03	ENSMUSG00000023092

Fhl2	0.380515108	0.14410463	8.28E-03	3.55E-02	ENSMUSG00000008136
Fhl3	0.761139782	0.29161235	9.05E-03	3.80E-02	ENSMUSG00000032643
Fhod1	1.664376581	0.66477294	1.23E-02	4.82E-02	ENSMUSG00000014778
Fibin	0.708454522	0.13255263	9.06E-08	2.43E-06	ENSMUSG00000074971
Fibp	0.567387543	0.13322695	2.06E-05	2.69E-04	ENSMUSG00000024911
Ficd	0.952148525	0.28566116	8.59E-04	5.88E-03	ENSMUSG00000053334
Fignl2	2.98478526	1.17692333	1.12E-02	4.49E-02	ENSMUSG00000095407
Fip1l1	-0.903380186	0.15137921	2.41E-09	9.84E-08	ENSMUSG00000029227
Fkbp10	0.693321557	0.11698673	3.09E-09	1.22E-07	ENSMUSG00000001555
Fkbp11	-0.497030054	0.13681058	2.80E-04	2.32E-03	ENSMUSG00000003355
Fkbp14	0.758221378	0.25465446	2.91E-03	1.55E-02	ENSMUSG00000038074
Fkbp2	0.823830401	0.17138307	1.53E-06	2.94E-05	ENSMUSG00000056629
Fkbp3	-0.728132354	0.18240842	6.56E-05	7.17E-04	ENSMUSG00000020949
Fkbp5	0.301773115	0.11326677	7.72E-03	3.37E-02	ENSMUSG00000024222
Fkbp8	0.70741211	0.1177411	1.88E-09	7.81E-08	ENSMUSG00000019428
Fkrp	1.098658541	0.22494438	1.04E-06	2.10E-05	ENSMUSG00000048920
Flcn	0.931255404	0.2408852	1.11E-04	1.08E-03	ENSMUSG00000032633
Flii	0.394016944	0.15496993	1.10E-02	4.43E-02	ENSMUSG00000002812
Flna	0.81819547	0.12581593	7.87E-11	4.64E-09	ENSMUSG00000031328
Flnb	0.660705678	0.16397417	5.59E-05	6.30E-04	ENSMUSG00000025278
Flot2	0.563469973	0.15453099	2.66E-04	2.23E-03	ENSMUSG00000061981
Flt3l	-1.286268889	0.37130129	5.32E-04	3.94E-03	ENSMUSG00000110185
Flywch2	1.001012289	0.25053889	6.46E-05	7.10E-04	ENSMUSG00000023911
Fmnl2	-0.974451417	0.2041087	1.80E-06	3.41E-05	ENSMUSG00000036053
Fmo1	1.021338429	0.17207389	2.93E-09	1.17E-07	ENSMUSG00000040181
Fmod	0.90347267	0.19344202	3.00E-06	5.24E-05	ENSMUSG00000041559
Fn3krp	0.945367165	0.32101551	3.23E-03	1.68E-02	ENSMUSG00000039253
Fndc1	1.122192042	0.20930675	8.25E-08	2.24E-06	ENSMUSG00000071984
Fndc3a	-0.735840969	0.26738404	5.92E-03	2.73E-02	ENSMUSG00000033487
Fndc3b	-0.502374445	0.18337005	6.15E-03	2.81E-02	ENSMUSG00000039286
Fnip1	-0.647908016	0.20240833	1.37E-03	8.50E-03	ENSMUSG00000035992
Fnip2	-0.730195289	0.26355388	5.60E-03	2.62E-02	ENSMUSG00000061175
Foxa2	1.737127389	0.34822151	6.08E-07	1.30E-05	ENSMUSG00000037025
Foxd1	2.177982782	0.5574284	9.34E-05	9.52E-04	ENSMUSG00000078302
Foxp1	-0.637205531	0.16143769	7.91E-05	8.33E-04	ENSMUSG00000030067
Frat2	-1.351001414	0.39090051	5.48E-04	4.04E-03	ENSMUSG00000047604
Frzb	0.949677829	0.1566092	1.33E-09	5.77E-08	ENSMUSG00000027004
Fscn1	0.50468065	0.12749097	7.54E-05	8.01E-04	ENSMUSG00000029581
Fsd1l	-3.128408113	1.11489536	5.02E-03	2.40E-02	ENSMUSG00000054752
Fst	0.957865286	0.18318764	1.71E-07	4.21E-06	ENSMUSG00000021765
Fstl1	0.317304762	0.11673472	6.56E-03	2.97E-02	ENSMUSG00000022816
Fstl4	2.759647946	0.70057524	8.18E-05	8.54E-04	ENSMUSG00000036264
Fth1	-0.721710208	0.10764775	2.02E-11	1.35E-09	ENSMUSG00000024661

Ftsj3	-0.783116695	0.18143036	1.59E-05	2.17E-04	ENSMUSG00000020706
Fubp1	-0.636347769	0.14337475	9.06E-06	1.33E-04	ENSMUSG00000028034
Fuca1	0.485948263	0.12032527	5.38E-05	6.10E-04	ENSMUSG00000028673
Fuca2	0.658913484	0.19785069	8.67E-04	5.93E-03	ENSMUSG00000019810
Furin	0.62952545	0.12270153	2.89E-07	6.71E-06	ENSMUSG00000030530
Fut11	0.788307772	0.20681299	1.38E-04	1.30E-03	ENSMUSG00000039357
Fuz	0.729402008	0.19364337	1.65E-04	1.51E-03	ENSMUSG00000011658
Fxr1	-1.320934026	0.24721439	9.13E-08	2.44E-06	ENSMUSG00000027680
Fxr2	-0.407260717	0.13043886	1.79E-03	1.05E-02	ENSMUSG00000018765
Fxyd1	0.749723756	0.10548304	1.18E-12	9.37E-11	ENSMUSG00000036570
Fxyd2	1.357949934	0.35730713	1.44E-04	1.35E-03	ENSMUSG00000059412
Fxyd3	0.941725143	0.16785279	2.02E-08	6.56E-07	ENSMUSG00000057092
Fzd1	1.615174053	0.20473915	3.05E-15	3.37E-13	ENSMUSG00000044674
Fzd2	0.795535524	0.15317349	2.06E-07	4.99E-06	ENSMUSG00000050288
Fzd5	2.108616692	0.27601437	2.18E-14	2.14E-12	ENSMUSG00000045005
Fzd8	1.196318291	0.18853947	2.22E-10	1.18E-08	ENSMUSG00000036904
Fzd9	1.247889055	0.27986067	8.24E-06	1.23E-04	ENSMUSG00000049551
Fzr1	0.681968422	0.20603458	9.33E-04	6.28E-03	ENSMUSG00000020235
G0s2	0.583036651	0.15110423	1.14E-04	1.11E-03	ENSMUSG00000009633
G3bp1	-0.289568127	0.11539523	1.21E-02	4.77E-02	ENSMUSG00000018583
G6pdx	0.416748273	0.14681996	4.53E-03	2.21E-02	ENSMUSG00000031400
Gaa	0.537970702	0.13556642	7.24E-05	7.77E-04	ENSMUSG00000025579
Gab2	-0.557836529	0.21724336	1.02E-02	4.19E-02	ENSMUSG00000004508
Gabarapl1	-0.352891415	0.10055417	4.49E-04	3.44E-03	ENSMUSG00000030161
Gabbr1	1.091812621	0.23028845	2.13E-06	3.90E-05	ENSMUSG00000024462
Gabpb1	-0.93116176	0.19072386	1.05E-06	2.11E-05	ENSMUSG00000027361
Gadd45a	-1.160523608	0.13448017	6.15E-18	9.53E-16	ENSMUSG00000036390
Gadd45g	0.466614328	0.14745756	1.55E-03	9.37E-03	ENSMUSG00000021453
Gadd45gip1	0.443566526	0.14276541	1.89E-03	1.10E-02	ENSMUSG00000033751
Gale	0.485882995	0.18921286	1.02E-02	4.19E-02	ENSMUSG00000028671
Galk1	0.393981218	0.11995042	1.02E-03	6.71E-03	ENSMUSG00000020766
Galns	0.708410523	0.26074886	6.59E-03	2.97E-02	ENSMUSG00000015027
Galnt10	0.606773509	0.18781379	1.23E-03	7.87E-03	ENSMUSG00000020520
Galnt15	-0.560772783	0.21879109	1.04E-02	4.24E-02	ENSMUSG00000021903
Galnt17	0.653210548	0.25516767	1.05E-02	4.27E-02	ENSMUSG00000034040
Galnt3	-1.679810743	0.63997846	8.67E-03	3.68E-02	ENSMUSG00000026994
Galnt7	-1.129839984	0.35228424	1.34E-03	8.34E-03	ENSMUSG00000031608
Galr2	1.671776342	0.45252758	2.20E-04	1.91E-03	ENSMUSG00000020793
Gamt	1.340278502	0.26987364	6.82E-07	1.43E-05	ENSMUSG00000020150
Ganab	0.453662612	0.14437353	1.68E-03	1.00E-02	ENSMUSG00000071650
Gapvd1	-0.8284284	0.24515826	7.27E-04	5.12E-03	ENSMUSG00000026867
Gars	-0.301855842	0.10373628	3.62E-03	1.84E-02	ENSMUSG00000029777
Gas1	1.750946152	0.1958616	3.90E-19	6.76E-17	ENSMUSG00000052957

Gas5	-1.03050047	0.11636854	8.33E-19	1.41E-16	ENSMUSG00000053332
Gas6	1.350744497	0.12037837	3.22E-29	1.70E-26	ENSMUSG00000031451
Gatad2b	-0.508234906	0.16475855	2.04E-03	1.16E-02	ENSMUSG00000042390
Gatd1	0.401668838	0.1453823	5.73E-03	2.67E-02	ENSMUSG00000051007
Gatd3a	0.530542897	0.14612784	2.83E-04	2.34E-03	ENSMUSG00000053329
Gba	0.630343298	0.170928	2.26E-04	1.95E-03	ENSMUSG00000028048
Gba2	1.418104734	0.52593413	7.01E-03	3.13E-02	ENSMUSG00000028467
Gbe1	-0.562809321	0.14361996	8.90E-05	9.14E-04	ENSMUSG00000022707
Gcc2	-0.988833877	0.33473834	3.14E-03	1.65E-02	ENSMUSG00000038039
Gch1	-1.886937256	0.2169692	3.41E-18	5.49E-16	ENSMUSG00000037580
Gclc	-1.13982781	0.17815411	1.57E-10	8.64E-09	ENSMUSG00000032350
Gclm	-1.043404314	0.12249869	1.63E-17	2.30E-15	ENSMUSG00000028124
Gcnt2	-0.673198395	0.23323182	3.90E-03	1.96E-02	ENSMUSG00000021360
Gdf10	1.773513736	0.35556037	6.10E-07	1.31E-05	ENSMUSG00000021943
Gdf15	-1.958417624	0.19862287	6.21E-23	1.82E-20	ENSMUSG00000038508
Gdi2	-0.446387203	0.12009087	2.02E-04	1.77E-03	ENSMUSG00000021218
Gdpd1	-0.765280324	0.2750284	5.39E-03	2.54E-02	ENSMUSG00000061666
Gdpd2	3.471785359	1.36447579	1.09E-02	4.42E-02	ENSMUSG00000019359
Gdpgp1	0.912506807	0.30144399	2.47E-03	1.36E-02	ENSMUSG00000050973
Gemin5	-0.86877473	0.23628044	2.36E-04	2.02E-03	ENSMUSG00000037275
Gfer	-0.542671965	0.12778335	2.17E-05	2.81E-04	ENSMUSG00000040888
Gfod2	1.479048147	0.38401342	1.17E-04	1.14E-03	ENSMUSG00000013150
Gfpt1	-0.611011217	0.18128196	7.50E-04	5.26E-03	ENSMUSG00000029992
Gfra2	-1.196733008	0.25763573	3.40E-06	5.82E-05	ENSMUSG00000022103
Gfra4	1.069979193	0.25117502	2.05E-05	2.69E-04	ENSMUSG00000027316
Ggact	0.806116052	0.20168598	6.42E-05	7.08E-04	ENSMUSG00000041625
Ggct	-1.392238423	0.29191869	1.85E-06	3.47E-05	ENSMUSG00000002797
Ggcx	0.674007588	0.16941423	6.94E-05	7.50E-04	ENSMUSG00000053460
Ggh	0.729666632	0.1870713	9.60E-05	9.72E-04	ENSMUSG00000073987
Ggnbp2	-1.510407387	0.20017892	4.51E-14	4.30E-12	ENSMUSG00000020530
Ggps1	-0.931550118	0.18168849	2.94E-07	6.79E-06	ENSMUSG00000021302
Ggt5	1.582416536	0.63324829	1.25E-02	4.87E-02	ENSMUSG00000006344
Ghitm	-0.776152828	0.10722826	4.54E-13	3.91E-11	ENSMUSG00000041028
Gid4	-0.5639312	0.14861987	1.48E-04	1.38E-03	ENSMUSG00000018415
Gid8	0.625455531	0.23140891	6.88E-03	3.08E-02	ENSMUSG00000027573
Gigyf2	-0.79106985	0.27743032	4.35E-03	2.14E-02	ENSMUSG00000048000
Ginm1	-0.554283767	0.15546973	3.64E-04	2.88E-03	ENSMUSG00000040006
Gipc1	0.669310274	0.18149139	2.26E-04	1.95E-03	ENSMUSG00000019433
Glce	-0.824341266	0.30605996	7.07E-03	3.15E-02	ENSMUSG00000032252
Gli1	1.450819444	0.25880172	2.07E-08	6.70E-07	ENSMUSG00000025407
Gli2	1.686540315	0.30429743	2.98E-08	9.26E-07	ENSMUSG00000048402
Glipr2	-0.5074266	0.15036131	7.39E-04	5.19E-03	ENSMUSG00000028480
Glis2	0.781856268	0.25271931	1.98E-03	1.13E-02	ENSMUSG00000014303

Glmn	-1.855737545	0.4651143	6.61E-05	7.21E-04	ENSMUSG00000029276
Glmp	0.851671955	0.13139651	9.07E-11	5.26E-09	ENSMUSG00000001418
Glrx	-0.661990868	0.14020499	2.34E-06	4.24E-05	ENSMUSG00000021591
Glrx3	-0.447032051	0.13861404	1.26E-03	7.98E-03	ENSMUSG00000031068
Gls	-0.48263084	0.19249672	1.22E-02	4.79E-02	ENSMUSG00000026103
Gm10076	-0.594494763	0.23584061	1.17E-02	4.65E-02	ENSMUSG00000060143
Gm10275	-0.606560224	0.21883706	5.58E-03	2.61E-02	ENSMUSG00000069682
Gm10689	-0.663181647	0.26484542	1.23E-02	4.82E-02	ENSMUSG00000074412
Gm11476	1.591009365	0.5668107	5.00E-03	2.40E-02	ENSMUSG00000085596
Gm11478	-0.540836113	0.18685427	3.80E-03	1.91E-02	ENSMUSG00000083992
Gm11747	3.059558584	0.64341809	1.98E-06	3.69E-05	ENSMUSG00000086514
Gm11826	-0.682237391	0.21980558	1.91E-03	1.10E-02	ENSMUSG00000083328
Gm11956	-0.550592554	0.16791383	1.04E-03	6.83E-03	ENSMUSG00000084012
Gm12022	-3.557534597	1.22828739	3.78E-03	1.90E-02	ENSMUSG00000085675
Gm13166	-1.482206815	0.38465898	1.17E-04	1.13E-03	ENSMUSG00000085525
Gm13205	-1.286140843	0.37416838	5.87E-04	4.29E-03	ENSMUSG00000086606
Gm13411	1.770675062	0.44453155	6.80E-05	7.38E-04	ENSMUSG00000085999
Gm13586	-1.257883691	0.43641501	3.95E-03	1.98E-02	ENSMUSG00000082931
Gm13594	1.857289228	0.57349803	1.20E-03	7.72E-03	ENSMUSG00000086881
Gm13815	-1.21990656	0.46297046	8.41E-03	3.59E-02	ENSMUSG00000080860
Gm13889	-1.047256483	0.20355738	2.68E-07	6.25E-06	ENSMUSG00000087006
Gm14138	-1.347593809	0.49435427	6.41E-03	2.91E-02	ENSMUSG00000081572
Gm15148	-0.855309101	0.33077361	9.72E-03	4.03E-02	ENSMUSG00000082423
Gm15464	-0.78616246	0.24396996	1.27E-03	8.03E-03	ENSMUSG00000082609
Gm15510	1.209210743	0.46357682	9.10E-03	3.82E-02	ENSMUSG00000086604
Gm16845	1.012210997	0.30637129	9.54E-04	6.37E-03	ENSMUSG00000096917
Gm17066	-0.939288815	0.36080654	9.23E-03	3.87E-02	ENSMUSG00000091509
Gm19353	0.510657869	0.17411104	3.36E-03	1.74E-02	ENSMUSG00000098332
Gm19412	1.12569857	0.43117981	9.03E-03	3.80E-02	ENSMUSG00000101013
Gm19705	1.389312963	0.35863037	1.07E-04	1.06E-03	ENSMUSG00000097080
Gm19739	-0.921489479	0.11943134	1.20E-14	1.23E-12	ENSMUSG00000104960
Gm19810	-0.611850575	0.19472522	1.68E-03	1.00E-02	ENSMUSG00000111361
Gm2000	-0.616631827	0.19152881	1.28E-03	8.09E-03	ENSMUSG00000078193
Gm2415	1.325369394	0.32137322	3.72E-05	4.45E-04	ENSMUSG00000086119
Gm24276	-1.045555276	0.39165595	7.59E-03	3.32E-02	ENSMUSG00000076135
Gm24951	-0.640138606	0.24965604	1.03E-02	4.23E-02	ENSMUSG00000076136
Gm26534	1.03214659	0.39491574	8.96E-03	3.78E-02	ENSMUSG00000097729
Gm26917	1.482425626	0.40201149	2.26E-04	1.95E-03	ENSMUSG00000097915
Gm2830	-0.580245993	0.20052749	3.81E-03	1.92E-02	ENSMUSG00000086567
Gm28730	1.149654933	0.44247526	9.37E-03	3.91E-02	ENSMUSG00000101438
Gm34006	1.440750582	0.41896015	5.84E-04	4.27E-03	ENSMUSG00000110980
Gm34342	1.173017877	0.39380735	2.90E-03	1.54E-02	ENSMUSG00000102101
Gm3550	-0.454130356	0.15623511	3.65E-03	1.86E-02	ENSMUSG00000078240

Gm3604	-0.824908975	0.27449788	2.65E-03	1.44E-02	ENSMUSG00000094870
Gm36378	-0.97288981	0.27106184	3.32E-04	2.68E-03	ENSMUSG00000106926
Gm37494	-1.863996503	0.53665573	5.14E-04	3.83E-03	ENSMUSG00000108609
Gm37510	-0.454263578	0.15574726	3.54E-03	1.81E-02	ENSMUSG00000103928
Gm37893	1.259812549	0.32345584	9.83E-05	9.90E-04	ENSMUSG00000103906
Gm3800	-1.54895206	0.49649234	1.81E-03	1.06E-02	ENSMUSG00000113643
Gm38220	3.370009775	0.7977875	2.40E-05	3.06E-04	ENSMUSG00000102158
Gm40598	1.578444685	0.38588542	4.31E-05	5.06E-04	ENSMUSG00000113581
Gm42815	-1.051467865	0.34794427	2.51E-03	1.38E-02	ENSMUSG00000104649
Gm42957	-0.508075708	0.1517262	8.12E-04	5.61E-03	ENSMUSG00000105814
Gm43088	-0.479084121	0.15263959	1.70E-03	1.01E-02	ENSMUSG00000105644
Gm43712	-2.001529068	0.76264744	8.68E-03	3.68E-02	ENSMUSG00000104378
Gm4468	-0.742856354	0.22872069	1.16E-03	7.50E-03	ENSMUSG00000092014
Gm45420	0.810346585	0.19602571	3.57E-05	4.28E-04	ENSMUSG00000110206
Gm45716	-0.949772052	0.27334958	5.12E-04	3.82E-03	ENSMUSG00000110313
Gm46224	-0.681576956	0.26930543	1.14E-02	4.54E-02	ENSMUSG00000111897
Gm4673	4.630077385	1.24348194	1.96E-04	1.74E-03	ENSMUSG00000097747
Gm47204	1.845315731	0.6191335	2.88E-03	1.54E-02	ENSMUSG00000110344
Gm47445	1.68407296	0.46495016	2.92E-04	2.41E-03	ENSMUSG00000111293
Gm49041	1.587507264	0.59950565	8.10E-03	3.49E-02	ENSMUSG00000115007
Gm49797	-1.109254627	0.26489054	2.82E-05	3.51E-04	ENSMUSG00000116564
Gm4997	-0.516469698	0.1270591	4.81E-05	5.55E-04	ENSMUSG00000045694
Gm5617	0.859977932	0.15039616	1.08E-08	3.81E-07	ENSMUSG00000042293
Gm5805	-0.439837908	0.15971818	5.89E-03	2.72E-02	ENSMUSG00000061848
Gm5963	-0.569043353	0.15858711	3.33E-04	2.69E-03	ENSMUSG00000052192
Gm6030	-0.912443557	0.20133598	5.84E-06	9.25E-05	ENSMUSG00000062284
Gm6977	-0.77865469	0.29866508	9.13E-03	3.83E-02	ENSMUSG00000084289
Gm7117	-0.644938785	0.19963598	1.24E-03	7.87E-03	ENSMUSG00000063442
Gm7424	-0.917055102	0.36543488	1.21E-02	4.77E-02	ENSMUSG00000108442
Gm7691	1.20590951	0.30704175	8.58E-05	8.85E-04	ENSMUSG00000115026
Gm8213	-0.587835012	0.15316967	1.24E-04	1.19E-03	ENSMUSG00000082474
Gm8292	0.874301704	0.27601932	1.54E-03	9.31E-03	ENSMUSG00000100017
Gm867	0.935887647	0.37632809	1.29E-02	5.00E-02	ENSMUSG00000050157
Gm9703	-1.109240564	0.41791628	7.95E-03	3.44E-02	ENSMUSG00000082741
Gm9776	1.171746953	0.46705496	1.21E-02	4.77E-02	ENSMUSG00000042857
Gm9794	-1.427406733	0.48862258	3.49E-03	1.79E-02	ENSMUSG00000107082
Gm9892	-2.136265556	0.54803749	9.70E-05	9.81E-04	ENSMUSG00000052825
Gmfb	-0.594743935	0.14364343	3.47E-05	4.18E-04	ENSMUSG00000062014
Gmppa	0.57731264	0.16201895	3.66E-04	2.90E-03	ENSMUSG00000033021
Gna11	0.348729286	0.11826628	3.19E-03	1.67E-02	ENSMUSG00000034781
Gna13	-0.755156589	0.15182109	6.56E-07	1.39E-05	ENSMUSG00000020611
Gnai2	0.395615486	0.09741805	4.89E-05	5.59E-04	ENSMUSG00000032562
Gnai3	-0.865116838	0.23247895	1.98E-04	1.75E-03	ENSMUSG00000000001

Gnb2	0.392580681	0.12567048	1.78E-03	1.05E-02	ENSMUSG00000029713
Gng11	1.208526614	0.12200579	3.94E-23	1.18E-20	ENSMUSG00000032766
Gng12	-0.319482157	0.11633082	6.03E-03	2.77E-02	ENSMUSG00000036402
Gnl2	-0.9684398	0.19688517	8.71E-07	1.80E-05	ENSMUSG00000028869
Gnl3	-1.489139728	0.20972024	1.24E-12	9.73E-11	ENSMUSG00000042354
Gnpda2	-1.415405982	0.55976319	1.15E-02	4.56E-02	ENSMUSG00000029209
Gnpnat1	-0.609714173	0.17424419	4.67E-04	3.55E-03	ENSMUSG00000037722
Gnptg	0.584903678	0.14892307	8.58E-05	8.85E-04	ENSMUSG00000035521
Gns	0.358340616	0.12740956	4.92E-03	2.37E-02	ENSMUSG00000034707
Golga3	-0.931571717	0.26420656	4.22E-04	3.27E-03	ENSMUSG00000029502
Golga4	-1.301854429	0.30274391	1.71E-05	2.30E-04	ENSMUSG00000038708
Golga7	-0.801966255	0.31933355	1.20E-02	4.75E-02	ENSMUSG00000015341
Golim4	-0.986125738	0.24225707	4.69E-05	5.43E-04	ENSMUSG00000034109
Golph3	-0.367456573	0.12222971	2.64E-03	1.44E-02	ENSMUSG00000022200
Gpaa1	0.699676768	0.17523592	6.53E-05	7.16E-04	ENSMUSG00000022561
Gpank1	-0.533485979	0.17294795	2.04E-03	1.16E-02	ENSMUSG00000092417
Gpbp1	-1.413059556	0.23510419	1.85E-09	7.76E-08	ENSMUSG00000032745
Gpc1	1.174039602	0.11190149	9.43E-26	3.55E-23	ENSMUSG00000034220
Gpc4	0.59039646	0.22393967	8.38E-03	3.58E-02	ENSMUSG00000031119
Gpd1	1.233791504	0.48352745	1.07E-02	4.35E-02	ENSMUSG00000023019
Gpd2	-0.954520383	0.19702388	1.27E-06	2.48E-05	ENSMUSG00000026827
Gper1	2.121419022	0.5031695	2.49E-05	3.16E-04	ENSMUSG00000053647
Gpi1	0.313137442	0.11295379	5.57E-03	2.61E-02	ENSMUSG00000036427
Gpr137	1.011539433	0.18161003	2.55E-08	8.07E-07	ENSMUSG00000024958
Gpr176	-0.692769548	0.21002291	9.72E-04	6.47E-03	ENSMUSG00000040133
Gpr89	0.619542516	0.21043421	3.24E-03	1.69E-02	ENSMUSG00000028096
Gprc5c	0.951584526	0.28008114	6.80E-04	4.82E-03	ENSMUSG00000051043
Gpsm3	0.764224536	0.1941621	8.28E-05	8.62E-04	ENSMUSG00000034786
Gpx3	0.644092611	0.08931353	5.53E-13	4.73E-11	ENSMUSG00000018339
Gpx7	0.454494345	0.18016	1.16E-02	4.63E-02	ENSMUSG00000028597
Gpx8	0.319307425	0.12408769	1.01E-02	4.14E-02	ENSMUSG00000021760
Grap	-1.054275592	0.27498421	1.26E-04	1.21E-03	ENSMUSG00000004837
Grasp	1.3426576	0.14339003	7.70E-21	1.56E-18	ENSMUSG00000000531
Grhpr	0.635887747	0.16347225	1.00E-04	1.00E-03	ENSMUSG00000035637
Grina	0.490345869	0.13458772	2.69E-04	2.25E-03	ENSMUSG00000022564
Grpel1	-0.882322032	0.13479349	5.92E-11	3.61E-09	ENSMUSG00000029198
Grpel2	-0.988342009	0.25198161	8.77E-05	9.03E-04	ENSMUSG00000024580
Grsf1	-0.628513492	0.16520551	1.42E-04	1.33E-03	ENSMUSG00000044221
Gsc	1.090630222	0.30803108	3.99E-04	3.12E-03	ENSMUSG00000021095
Gse1	1.534219329	0.26692651	9.05E-09	3.24E-07	ENSMUSG00000031822
Gsg1l	2.766545224	0.48632694	1.28E-08	4.45E-07	ENSMUSG00000046182
Gsk3b	-0.517550079	0.19621237	8.35E-03	3.57E-02	ENSMUSG00000022812
Gsn	0.891663533	0.10579014	3.50E-17	4.90E-15	ENSMUSG00000026879

Gspt1	-0.720361487	0.21807347	9.56E-04	6.38E-03	ENSMUSG00000062203
Gsta4	-1.322629238	0.19708795	1.93E-11	1.30E-09	ENSMUSG00000032348
Gstk1	0.752230634	0.17719167	2.18E-05	2.83E-04	ENSMUSG00000029864
Gstm1	0.274605448	0.09382385	3.42E-03	1.76E-02	ENSMUSG00000058135
Gstm2	0.946097545	0.17127633	3.32E-08	1.02E-06	ENSMUSG00000040562
Gstm4	0.674471386	0.20389344	9.40E-04	6.32E-03	ENSMUSG00000027890
Gstm7	2.374514581	0.44444368	9.16E-08	2.45E-06	ENSMUSG00000004035
Gstt3	0.836766784	0.17762266	2.47E-06	4.43E-05	ENSMUSG00000001665
Gtf2f2	-0.560652204	0.18719634	2.74E-03	1.48E-02	ENSMUSG00000067995
Gtf2h1	-1.046610162	0.30453531	5.89E-04	4.29E-03	ENSMUSG00000006599
Gtf2i	0.476118353	0.14886821	1.38E-03	8.55E-03	ENSMUSG00000060261
Gtpbp4	-1.324998691	0.24630358	7.47E-08	2.06E-06	ENSMUSG00000021149
Guf1	-0.90451875	0.35146166	1.01E-02	4.14E-02	ENSMUSG00000029208
Gusb	0.65729206	0.1658608	7.40E-05	7.92E-04	ENSMUSG00000025534
Gxylt1	-0.593435532	0.22397106	8.06E-03	3.48E-02	ENSMUSG00000036197
Gxylt2	0.591115287	0.19887426	2.96E-03	1.57E-02	ENSMUSG00000030074
Gypc	0.550014983	0.20953746	8.67E-03	3.68E-02	ENSMUSG00000090523
Gys1	0.858091185	0.18347325	2.91E-06	5.10E-05	ENSMUSG00000003865
H1f0	0.342759642	0.10908313	1.68E-03	1.00E-02	ENSMUSG00000096145
H1fx	0.722300321	0.25077125	3.97E-03	1.98E-02	ENSMUSG00000044927
H2-Aa	-0.567422626	0.22218666	1.07E-02	4.33E-02	ENSMUSG00000036594
H2-Eb1	-0.761275267	0.30506431	1.26E-02	4.91E-02	ENSMUSG00000060586
H2-K1	0.669096961	0.15499739	1.58E-05	2.17E-04	ENSMUSG00000061232
H2-T22	0.68575093	0.24634946	5.38E-03	2.54E-02	ENSMUSG00000056116
H2afj	0.44398734	0.1161139	1.31E-04	1.25E-03	ENSMUSG00000060032
H2afz	-0.449988554	0.14512354	1.93E-03	1.11E-02	ENSMUSG00000037894
Hacd1	0.540301633	0.13146816	3.96E-05	4.70E-04	ENSMUSG00000063275
Hacd4	1.349374774	0.39589083	6.53E-04	4.68E-03	ENSMUSG00000028497
Hadh	0.514007983	0.12388691	3.34E-05	4.07E-04	ENSMUSG00000027984
Hadha	0.466971441	0.13620886	6.07E-04	4.39E-03	ENSMUSG00000025745
Haghl	1.288439266	0.24527077	1.50E-07	3.77E-06	ENSMUSG00000061046
Hand2	1.281977089	0.42878842	2.79E-03	1.50E-02	ENSMUSG00000038193
Hapln1	1.006465698	0.16458629	9.65E-10	4.38E-08	ENSMUSG00000021613
Haus3	-0.868179381	0.32804541	8.13E-03	3.50E-02	ENSMUSG00000079555
Haus6	-1.520053695	0.43959404	5.44E-04	4.02E-03	ENSMUSG00000038047
Hbp1	-0.359185344	0.14429558	1.28E-02	4.97E-02	ENSMUSG00000002996
Hbs1l	-0.760264574	0.16504978	4.10E-06	6.83E-05	ENSMUSG00000019977
Hcfc1r1	0.304049507	0.09726844	1.77E-03	1.04E-02	ENSMUSG00000023904
Hcn2	1.784856115	0.58889633	2.44E-03	1.35E-02	ENSMUSG00000020331
Hdac10	1.12262574	0.35259968	1.45E-03	8.89E-03	ENSMUSG00000062906
Hdac2	-0.863900396	0.19008867	5.50E-06	8.75E-05	ENSMUSG00000019777
Hdac5	0.556112962	0.16491693	7.46E-04	5.23E-03	ENSMUSG00000008855
Hdhd3	1.334049245	0.41770001	1.40E-03	8.65E-03	ENSMUSG00000038422

Hdlbp	-0.379080043	0.13761052	5.87E-03	2.72E-02	ENSMUSG00000034088
Heatr1	-0.846625059	0.29019617	3.53E-03	1.81E-02	ENSMUSG00000050244
Hebp1	0.660649816	0.16852519	8.85E-05	9.09E-04	ENSMUSG00000042770
Hebp2	0.486037201	0.15174415	1.36E-03	8.45E-03	ENSMUSG00000019853
Heca	-0.888855438	0.25346904	4.54E-04	3.46E-03	ENSMUSG00000039879
Hectd1	-0.924929299	0.20167769	4.51E-06	7.44E-05	ENSMUSG00000035247
Hectd3	0.67351867	0.22234227	2.45E-03	1.35E-02	ENSMUSG00000046861
Heph	-1.528407648	0.45849755	8.58E-04	5.87E-03	ENSMUSG00000031209
Herc1	-1.050831836	0.30186476	4.99E-04	3.74E-03	ENSMUSG00000038664
Herc4	-0.999294742	0.26690803	1.81E-04	1.62E-03	ENSMUSG00000020064
Herpud2	-0.600333006	0.16644227	3.10E-04	2.53E-03	ENSMUSG00000008429
Hes1	1.49379532	0.27325663	4.59E-08	1.34E-06	ENSMUSG00000022528
Hexim1	1.077338158	0.19219597	2.08E-08	6.70E-07	ENSMUSG00000048878
Hey1	1.18584223	0.29806167	6.93E-05	7.50E-04	ENSMUSG00000040289
Hgsnat	0.739974012	0.15319517	1.36E-06	2.65E-05	ENSMUSG00000037260
Hhat	1.216595065	0.38981254	1.80E-03	1.06E-02	ENSMUSG00000037375
Hhipl1	0.927420353	0.21292957	1.33E-05	1.86E-04	ENSMUSG00000021260
Hif1a	-0.726362201	0.1580309	4.30E-06	7.12E-05	ENSMUSG00000021109
Hif1an	0.621595879	0.21417348	3.70E-03	1.88E-02	ENSMUSG00000036450
Hist1h1c	0.53271209	0.12858268	3.43E-05	4.15E-04	ENSMUSG00000036181
Hk1	0.378178166	0.13459928	4.96E-03	2.38E-02	ENSMUSG00000037012
Hltf	-1.677317478	0.53398972	1.68E-03	1.00E-02	ENSMUSG00000002428
Hlx	0.889663771	0.16606332	8.44E-08	2.28E-06	ENSMUSG00000039377
Hmga1	-2.445969573	0.5030763	1.16E-06	2.30E-05	ENSMUSG00000046711
Hmga2	-1.667200762	0.15075852	1.99E-28	9.03E-26	ENSMUSG00000056758
Hmgcr	0.65083468	0.26084313	1.26E-02	4.91E-02	ENSMUSG00000021670
Hmgcs2	3.283901158	0.24670399	2.00E-40	2.63E-37	ENSMUSG00000027875
Hmgn3	0.668687916	0.13112284	3.40E-07	7.76E-06	ENSMUSG00000066456
Hmgn5	-1.340602669	0.29783311	6.76E-06	1.05E-04	ENSMUSG00000031245
Hmox1	-1.015289098	0.119064	1.50E-17	2.14E-15	ENSMUSG00000005413
Hnrnpa3	-0.980211516	0.27083586	2.96E-04	2.43E-03	ENSMUSG00000059005
Hnrnpc	-0.359871916	0.1199328	2.69E-03	1.46E-02	ENSMUSG00000060373
Hnrnpdl	-0.953977753	0.13399141	1.08E-12	8.63E-11	ENSMUSG00000029328
Hnrnpl	0.612697221	0.13806035	9.08E-06	1.34E-04	ENSMUSG00000015165
Hnrnpu	-0.729246829	0.13703889	1.03E-07	2.70E-06	ENSMUSG00000039630
Hopx	0.826400923	0.28110931	3.28E-03	1.70E-02	ENSMUSG00000059325
Hoxa6-201	-1.117226476	0.30562094	2.57E-04	2.16E-03	ENSMUSG00000116858
Hpf1	-0.738415181	0.23207901	1.46E-03	8.93E-03	ENSMUSG00000038005
Hpgd	1.204463959	0.35849268	7.80E-04	5.43E-03	ENSMUSG00000031613
Hprt	-0.443771279	0.13730788	1.23E-03	7.84E-03	ENSMUSG00000025630
Hps1	0.793093411	0.23301135	6.65E-04	4.73E-03	ENSMUSG00000025188
Hps3	-1.477416347	0.41641953	3.88E-04	3.04E-03	ENSMUSG00000027615
Hps4	0.618979249	0.24829929	1.27E-02	4.93E-02	ENSMUSG00000042328

Hpse	1.517820402	0.40651279	1.89E-04	1.68E-03	ENSMUSG00000035273
Hr	1.628699932	0.16785364	2.92E-22	7.26E-20	ENSMUSG00000022096
Hsd17b11	0.676148699	0.17332496	9.58E-05	9.71E-04	ENSMUSG00000029311
Hsp90aa1	-1.207423544	0.24297772	6.72E-07	1.42E-05	ENSMUSG00000021270
Hspa4	-0.796639009	0.16420478	1.23E-06	2.40E-05	ENSMUSG00000020361
Hspa4l	-2.35394267	0.65742298	3.43E-04	2.74E-03	ENSMUSG00000025757
Hspa8	-0.3823279	0.1299568	3.26E-03	1.70E-02	ENSMUSG00000015656
Hspa9	-1.05435389	0.12721313	1.15E-16	1.50E-14	ENSMUSG00000024359
Hspb7	-0.496706446	0.17238768	3.96E-03	1.98E-02	ENSMUSG00000006221
Hspbp1	0.477201358	0.16192818	3.21E-03	1.68E-02	ENSMUSG00000063802
Hspd1	-1.099661516	0.2076328	1.18E-07	3.06E-06	ENSMUSG00000025980
Hspe1	-0.639563597	0.13342464	1.64E-06	3.12E-05	ENSMUSG00000073676
Hspg2	0.917804832	0.1444735	2.11E-10	1.13E-08	ENSMUSG00000028763
Hsph1	-1.124438945	0.15502766	4.07E-13	3.55E-11	ENSMUSG00000029657
Htatip2	-0.449339294	0.13077966	5.91E-04	4.30E-03	ENSMUSG00000039745
Htra3	1.308028873	0.14056288	1.33E-20	2.58E-18	ENSMUSG00000029096
Hyal2	0.976803003	0.24676086	7.54E-05	8.01E-04	ENSMUSG00000010047
Iah1	0.635132056	0.19088164	8.77E-04	5.97E-03	ENSMUSG00000062054
Iars	-0.794419447	0.15859016	5.46E-07	1.19E-05	ENSMUSG00000037851
Ibtk	-0.890197627	0.21915969	4.87E-05	5.58E-04	ENSMUSG00000035941
Id3	1.387649929	0.12345437	2.59E-29	1.42E-26	ENSMUSG00000007872
Ide	-0.945639414	0.23995125	8.12E-05	8.49E-04	ENSMUSG00000056999
Idh1	0.439999374	0.12662433	5.11E-04	3.82E-03	ENSMUSG00000025950
Idh3g	0.413844189	0.13756318	2.63E-03	1.43E-02	ENSMUSG00000002010
Ier3	-0.379742299	0.13971252	6.57E-03	2.97E-02	ENSMUSG00000003541
Ier5	-0.534074859	0.14058351	1.45E-04	1.36E-03	ENSMUSG00000056708
Ier5l	0.798251311	0.20098278	7.13E-05	7.69E-04	ENSMUSG00000089762
Ifi27	1.040220428	0.15414456	1.50E-11	1.03E-09	ENSMUSG00000064215
Ifitm2	0.49760406	0.12800656	1.01E-04	1.01E-03	ENSMUSG00000060591
Ifitm5	2.340187205	0.26988239	4.28E-18	6.79E-16	ENSMUSG00000025489
Ifrd1	-1.300862452	0.13781642	3.76E-21	7.86E-19	ENSMUSG00000001627
Ift22	0.729504614	0.21280516	6.08E-04	4.40E-03	ENSMUSG00000007987
Ift27	0.937525303	0.20562557	5.13E-06	8.26E-05	ENSMUSG00000016637
Ift46	0.477291225	0.16059177	2.96E-03	1.57E-02	ENSMUSG00000002031
Igf1	0.410651554	0.12672484	1.19E-03	7.67E-03	ENSMUSG00000020053
Igf2bp2	-0.935400475	0.24659297	1.49E-04	1.38E-03	ENSMUSG00000033581
Igfbp4	0.830074107	0.12016126	4.92E-12	3.59E-10	ENSMUSG00000017493
Igfbp5	0.719492154	0.13919151	2.35E-07	5.58E-06	ENSMUSG00000026185
Igfbp6	-0.4947326	0.11923509	3.34E-05	4.07E-04	ENSMUSG00000023046
Igfbp7	0.300608239	0.09878568	2.34E-03	1.30E-02	ENSMUSG00000036256
Igip	-0.786431621	0.23145177	6.79E-04	4.82E-03	ENSMUSG00000109901
Igsf3	0.709579762	0.14445031	9.00E-07	1.85E-05	ENSMUSG00000042035
Igtp	1.406542383	0.38770315	2.86E-04	2.36E-03	ENSMUSG00000078853

Ihh	3.068278703	0.3285714	9.79E-21	1.95E-18	ENSMUSG00000006538
Ik	-0.724201964	0.17888988	5.16E-05	5.88E-04	ENSMUSG00000024474
Ikbip	-0.672785633	0.18719145	3.26E-04	2.64E-03	ENSMUSG00000019975
Ikzf5	-0.804101855	0.28172021	4.31E-03	2.13E-02	ENSMUSG00000040167
Il11ra1	0.70646411	0.14473978	1.06E-06	2.12E-05	ENSMUSG00000073889
Il13ra1	-0.998481647	0.24498059	4.59E-05	5.33E-04	ENSMUSG00000017057
Il16	1.23581814	0.32299105	1.30E-04	1.24E-03	ENSMUSG00000001741
Il17rc	0.851839007	0.23575181	3.02E-04	2.48E-03	ENSMUSG00000030281
Il1rl1	-1.477838423	0.27582213	8.42E-08	2.28E-06	ENSMUSG00000026069
Il6st	-0.386251867	0.13004494	2.98E-03	1.58E-02	ENSMUSG00000021756
Ilk	0.498747997	0.12272995	4.83E-05	5.56E-04	ENSMUSG00000030890
Ilvbl	0.684156521	0.15862177	1.61E-05	2.20E-04	ENSMUSG00000032763
Imp3	-0.494353685	0.10698445	3.82E-06	6.46E-05	ENSMUSG00000032288
Impad1	-0.729115293	0.15165696	1.53E-06	2.93E-05	ENSMUSG00000066324
Inafm2	0.574885979	0.20826336	5.77E-03	2.68E-02	ENSMUSG00000074918
Inca1	1.306659786	0.4341834	2.62E-03	1.42E-02	ENSMUSG00000057054
Ing1	-0.447552424	0.14131857	1.54E-03	9.33E-03	ENSMUSG00000045969
Inhba	-1.048372226	0.20338018	2.54E-07	5.98E-06	ENSMUSG00000041324
Inhbb	0.819321492	0.24609707	8.71E-04	5.94E-03	ENSMUSG00000037035
Inmt	4.199283768	0.83431455	4.82E-07	1.06E-05	ENSMUSG00000003477
Inpp5e	0.696314828	0.26184933	7.83E-03	3.40E-02	ENSMUSG00000026925
Inppl1	0.387713035	0.12362611	1.71E-03	1.01E-02	ENSMUSG00000032737
Insc	3.234161774	0.62226975	2.02E-07	4.90E-06	ENSMUSG00000048782
Ints6	-0.719141721	0.24961617	3.96E-03	1.98E-02	ENSMUSG00000035161
Ipo5	-0.729935463	0.16905312	1.58E-05	2.16E-04	ENSMUSG00000030662
Ipo7	-1.530522079	0.2431288	3.07E-10	1.57E-08	ENSMUSG00000066232
Iqgap1	-0.561127001	0.12278784	4.88E-06	7.93E-05	ENSMUSG00000030536
Ireb2	-0.879960616	0.32190553	6.26E-03	2.86E-02	ENSMUSG00000032293
Irf2bp1	0.696697711	0.24608886	4.64E-03	2.25E-02	ENSMUSG00000044030
Irf7	1.372866824	0.40111184	6.20E-04	4.48E-03	ENSMUSG00000025498
Irs2	-0.73859508	0.19699687	1.77E-04	1.60E-03	ENSMUSG00000038894
Irx3	1.059364804	0.18183337	5.68E-09	2.10E-07	ENSMUSG00000031734
Irx5	1.424671707	0.37206776	1.29E-04	1.23E-03	ENSMUSG00000031737
Islr	1.842963219	0.38561171	1.76E-06	3.33E-05	ENSMUSG00000037206
Islr2	1.728065576	0.4007698	1.62E-05	2.21E-04	ENSMUSG00000051243
Ism1	1.016039358	0.32099928	1.55E-03	9.35E-03	ENSMUSG00000074766
Isoc2a	0.767335697	0.13978001	4.03E-08	1.20E-06	ENSMUSG00000086784
Itga10	0.812020436	0.15713943	2.37E-07	5.62E-06	ENSMUSG00000090210
Itga11	0.81828525	0.17359668	2.43E-06	4.39E-05	ENSMUSG00000032243
Itga7	0.555353302	0.22284064	1.27E-02	4.94E-02	ENSMUSG00000025348
Itga9	1.323047365	0.40558564	1.11E-03	7.18E-03	ENSMUSG00000039115
Itgb2	1.072368993	0.35824066	2.76E-03	1.48E-02	ENSMUSG00000000290
Itgb5	1.415836236	0.13930955	2.89E-24	9.52E-22	ENSMUSG00000022817

Itgbl1	0.746900653	0.16605769	6.86E-06	1.06E-04	ENSMUSG00000032925
Itm2b	0.548881774	0.091649	2.11E-09	8.69E-08	ENSMUSG00000022108
Itm2c	0.450831151	0.12202271	2.20E-04	1.91E-03	ENSMUSG00000026223
Itpk1	0.913891214	0.29241532	1.78E-03	1.04E-02	ENSMUSG00000057963
Itpr2	-0.758493131	0.2374122	1.40E-03	8.63E-03	ENSMUSG00000030287
Itpripl1	1.349432974	0.46580438	3.77E-03	1.90E-02	ENSMUSG00000074825
Ivd	1.058516277	0.23499618	6.66E-06	1.03E-04	ENSMUSG00000027332
Ivns1abp	-0.436115768	0.14783754	3.18E-03	1.67E-02	ENSMUSG00000023150
Izumo4	1.938239228	0.44702834	1.45E-05	2.01E-04	ENSMUSG00000055862
Jade1	-0.904831071	0.22143855	4.39E-05	5.13E-04	ENSMUSG00000025764
Jade2	0.944153452	0.20590618	4.53E-06	7.46E-05	ENSMUSG00000020387
Jak1	-0.650551443	0.12910434	4.68E-07	1.03E-05	ENSMUSG00000028530
Jak2	-0.986924836	0.29349337	7.72E-04	5.39E-03	ENSMUSG00000024789
Jaml	1.588729755	0.48966481	1.18E-03	7.57E-03	ENSMUSG00000048534
Jmjd4	1.247763268	0.45802039	6.44E-03	2.92E-02	ENSMUSG00000036819
Jmjd8	0.92903745	0.2079168	7.88E-06	1.19E-04	ENSMUSG00000025736
Jmy	-0.675141884	0.20702181	1.11E-03	7.20E-03	ENSMUSG00000021690
Jpt2	-0.674121559	0.1807956	1.93E-04	1.71E-03	ENSMUSG00000024165
Jup	0.997952188	0.24658568	5.19E-05	5.91E-04	ENSMUSG00000001552
Kank2	0.69489501	0.1499701	3.59E-06	6.13E-05	ENSMUSG00000032194
Kank3	1.157628923	0.36954383	1.73E-03	1.02E-02	ENSMUSG00000042099
Kat8	-0.573284764	0.17760299	1.25E-03	7.93E-03	ENSMUSG00000030801
Katna1	-0.674187955	0.18987592	3.84E-04	3.02E-03	ENSMUSG00000019794
Katnbl1	-0.891910156	0.25193461	4.00E-04	3.12E-03	ENSMUSG00000027132
Kazald1	1.01971407	0.34989875	3.56E-03	1.82E-02	ENSMUSG00000025213
Kbtbd11	1.564214405	0.59127938	8.16E-03	3.50E-02	ENSMUSG00000055675
Kcmf1	-0.601601445	0.11943057	4.72E-07	1.04E-05	ENSMUSG00000055239
Kcnab3	-1.127017259	0.44695135	1.17E-02	4.64E-02	ENSMUSG00000018470
Kcnd1	1.747256484	0.55634202	1.69E-03	1.00E-02	ENSMUSG00000009731
Kcne4	0.590720138	0.15320446	1.15E-04	1.12E-03	ENSMUSG00000047330
Kcnh2	2.06756816	0.79663848	9.45E-03	3.94E-02	ENSMUSG00000038319
Kcnk1	2.462259365	0.28512218	5.83E-18	9.14E-16	ENSMUSG00000033998
Kcnq1ot1	2.210611179	0.84972035	9.28E-03	3.88E-02	ENSMUSG00000101603
Kctd1	0.627850101	0.24388056	1.00E-02	4.13E-02	ENSMUSG00000036225
Kctd11	0.55887125	0.17052215	1.05E-03	6.85E-03	ENSMUSG00000046731
Kctd13	-0.6973356	0.21491653	1.18E-03	7.57E-03	ENSMUSG00000030685
Kctd17	0.867294729	0.15509226	2.24E-08	7.15E-07	ENSMUSG00000033287
Kdm4b	0.800124066	0.1830347	1.23E-05	1.74E-04	ENSMUSG00000024201
Kdm5a	-0.807284787	0.22024679	2.47E-04	2.10E-03	ENSMUSG00000030180
Kdm7a	-0.798192407	0.26217063	2.33E-03	1.30E-02	ENSMUSG00000042599
Keap1	0.440316859	0.15091178	3.53E-03	1.81E-02	ENSMUSG00000003308
Khdc4	-0.5169298	0.17970058	4.02E-03	2.00E-02	ENSMUSG00000028060
Khdrbs1	-0.792988176	0.2734524	3.73E-03	1.89E-02	ENSMUSG00000028790

Kif21a	-1.948502084	0.4220499	3.90E-06	6.54E-05	ENSMUSG00000022629
Kif2a	-1.543173136	0.23385813	4.15E-11	2.65E-09	ENSMUSG00000021693
Kif3a	-1.515288492	0.29147309	2.01E-07	4.87E-06	ENSMUSG00000018395
Kif5b	-0.910854342	0.22972341	7.34E-05	7.86E-04	ENSMUSG00000006740
Kifc3	0.666534936	0.15600926	1.93E-05	2.57E-04	ENSMUSG00000031788
Klf2	0.841293474	0.16275054	2.35E-07	5.58E-06	ENSMUSG00000055148
Klf3	-0.672946033	0.17413975	1.11E-04	1.09E-03	ENSMUSG00000029178
Klf6	-0.856633161	0.22070389	1.04E-04	1.03E-03	ENSMUSG00000000078
Klf7	-1.022459811	0.17500301	5.14E-09	1.93E-07	ENSMUSG00000025959
Klf9	-1.404029867	0.18141357	9.99E-15	1.04E-12	ENSMUSG00000033863
Klhdc2	-0.289614431	0.10535413	5.98E-03	2.75E-02	ENSMUSG00000020978
Klhdc8a	1.927632543	0.49681168	1.04E-04	1.03E-03	ENSMUSG00000042115
Klhl7	-0.824223514	0.31094439	8.03E-03	3.47E-02	ENSMUSG00000028986
Klk10	2.181772322	0.85570149	1.08E-02	4.37E-02	ENSMUSG00000030693
Kmt2a	-0.667483956	0.22493035	3.00E-03	1.59E-02	ENSMUSG00000002028
Kmt2e	-0.49606496	0.1776386	5.23E-03	2.48E-02	ENSMUSG00000029004
Knop1	-0.529400709	0.14779423	3.41E-04	2.74E-03	ENSMUSG00000030980
Kpna1	-1.249652899	0.22867924	4.64E-08	1.34E-06	ENSMUSG00000022905
Kpna4	-0.816862717	0.228451	3.49E-04	2.79E-03	ENSMUSG00000027782
Kptn	1.201913622	0.3676632	1.08E-03	7.03E-03	ENSMUSG00000006021
Kras	-0.8718097	0.23762558	2.44E-04	2.08E-03	ENSMUSG00000030265
Krba1	1.175880213	0.40572271	3.75E-03	1.90E-02	ENSMUSG00000042810
Kremen1	0.507519078	0.15960429	1.47E-03	8.98E-03	ENSMUSG00000020393
Krr1	-1.27537945	0.34327342	2.03E-04	1.78E-03	ENSMUSG00000063334
Krt23	2.737492087	0.98332449	5.37E-03	2.53E-02	ENSMUSG00000006777
Krtcap2	-0.374852068	0.1095035	6.19E-04	4.47E-03	ENSMUSG00000042747
Ksr1	0.855578207	0.1767023	1.29E-06	2.51E-05	ENSMUSG00000018334
Ktn1	-1.478414306	0.26299387	1.89E-08	6.25E-07	ENSMUSG00000021843
L3mbtl3	-0.920272127	0.34364565	7.41E-03	3.27E-02	ENSMUSG00000039089
Lactb2	-0.604334867	0.22907069	8.33E-03	3.57E-02	ENSMUSG00000025937
Lage3	0.769749238	0.13982054	3.69E-08	1.11E-06	ENSMUSG00000015289
Lamb2	0.380473626	0.12443058	2.23E-03	1.25E-02	ENSMUSG00000052911
Lamc1	-0.373691681	0.14252036	8.74E-03	3.71E-02	ENSMUSG00000026478
Lamc2	-1.599005356	0.35705858	7.52E-06	1.15E-04	ENSMUSG00000026479
Lamp1	0.370709517	0.09829034	1.62E-04	1.48E-03	ENSMUSG00000031447
Lamtor1	0.550040262	0.15596651	4.21E-04	3.26E-03	ENSMUSG00000030842
Lamtor4	0.50896734	0.12077448	2.51E-05	3.18E-04	ENSMUSG00000050552
Laptm4a	0.299839988	0.11064042	6.73E-03	3.03E-02	ENSMUSG00000020585
Larp4	-1.134597188	0.32074407	4.04E-04	3.14E-03	ENSMUSG00000023025
Larp4b	-0.938138348	0.21650077	1.47E-05	2.03E-04	ENSMUSG00000033499
Larp6	0.913492347	0.26543508	5.79E-04	4.23E-03	ENSMUSG00000034839
Larp7	-0.638948544	0.2295824	5.38E-03	2.54E-02	ENSMUSG00000027968
Lars	-0.810882627	0.18152544	7.93E-06	1.19E-04	ENSMUSG00000024493

Lasp1	0.337588677	0.12904327	8.89E-03	3.76E-02	ENSMUSG00000038366
Lats1	-0.674046863	0.24759923	6.48E-03	2.94E-02	ENSMUSG00000040021
Layn	-0.969080541	0.17627808	3.85E-08	1.16E-06	ENSMUSG00000060594
Lbh	0.672239928	0.1111906	1.49E-09	6.37E-08	ENSMUSG00000024063
Lbp	0.546960554	0.15798436	5.36E-04	3.97E-03	ENSMUSG00000016024
Lcmt2	1.1187462	0.288262	1.04E-04	1.03E-03	ENSMUSG00000074890
Lcn2	-1.744478595	0.15738897	1.50E-28	7.06E-26	ENSMUSG00000026822
Lcorl	-1.152512482	0.41694784	5.71E-03	2.66E-02	ENSMUSG00000015882
Ldha	1.070079546	0.11200038	1.24E-21	2.73E-19	ENSMUSG00000063229
Ldhb	0.790283346	0.28117646	4.94E-03	2.38E-02	ENSMUSG00000030246
Ldlrap1	0.805102215	0.17170672	2.75E-06	4.89E-05	ENSMUSG00000037295
Leng9	1.208914467	0.40590666	2.90E-03	1.54E-02	ENSMUSG00000043432
Leprotl1	-0.568359646	0.10848044	1.61E-07	4.02E-06	ENSMUSG00000031513
Lfng	1.192902433	0.37457327	1.45E-03	8.88E-03	ENSMUSG00000029570
Lgals1	0.667678766	0.12067014	3.15E-08	9.68E-07	ENSMUSG00000068220
Lgals2	2.812712714	0.84259021	8.43E-04	5.78E-03	ENSMUSG00000043501
Lgals3	-0.308523761	0.10983431	4.97E-03	2.39E-02	ENSMUSG00000050335
Lgals3bp	1.028124502	0.1752562	4.45E-09	1.69E-07	ENSMUSG00000033880
Lgalsl	-0.774615466	0.24104567	1.31E-03	8.21E-03	ENSMUSG00000042363
Lgi2	-1.379078331	0.55349409	1.27E-02	4.94E-02	ENSMUSG00000039252
Lgi4	1.266979934	0.23539168	7.35E-08	2.04E-06	ENSMUSG00000036560
Lgmn	0.418950996	0.1469005	4.35E-03	2.14E-02	ENSMUSG00000021190
Lhfpl2	-0.353499456	0.14110435	1.22E-02	4.81E-02	ENSMUSG00000045312
Lifr	0.665620106	0.20716569	1.31E-03	8.22E-03	ENSMUSG00000054263
Limd2	0.598321648	0.13231341	6.13E-06	9.60E-05	ENSMUSG00000040699
Lims1	-0.740770086	0.25232762	3.33E-03	1.72E-02	ENSMUSG00000019920
Lin7c	-1.087078376	0.21382444	3.70E-07	8.36E-06	ENSMUSG00000027162
Lipc	4.860301474	1.15794723	2.70E-05	3.39E-04	ENSMUSG00000032207
Lipe	0.862858937	0.27494497	1.70E-03	1.01E-02	ENSMUSG00000003123
Lix1l	0.414808651	0.14555884	4.38E-03	2.15E-02	ENSMUSG00000049288
Llgl1	0.768423739	0.22978997	8.26E-04	5.68E-03	ENSMUSG00000020536
Lman1	-0.596336405	0.13011105	4.58E-06	7.52E-05	ENSMUSG00000041891
Lman2	0.338247135	0.12886418	8.67E-03	3.68E-02	ENSMUSG00000021484
Lmcd1	-1.016581911	0.27410297	2.08E-04	1.82E-03	ENSMUSG00000057604
Lmf1	0.681335678	0.15708982	1.44E-05	2.00E-04	ENSMUSG00000002279
Lmf2	0.76899815	0.20132121	1.34E-04	1.27E-03	ENSMUSG00000022614
Lmna	0.452186791	0.10517214	1.71E-05	2.31E-04	ENSMUSG00000028063
Loxl1	0.644963169	0.14736585	1.21E-05	1.70E-04	ENSMUSG00000032334
Loxl2	0.590430125	0.11828149	5.98E-07	1.29E-05	ENSMUSG00000034205
Loxl3	0.320067997	0.12751176	1.21E-02	4.76E-02	ENSMUSG00000000693
Lpcat3	0.465273528	0.16380114	4.50E-03	2.20E-02	ENSMUSG00000004270
Lpgat1	-0.539216224	0.20080207	7.25E-03	3.21E-02	ENSMUSG00000026623
Lpin2	-1.238823637	0.28893743	1.81E-05	2.41E-04	ENSMUSG00000024052

Lpp	-0.739759247	0.2061794	3.33E-04	2.69E-03	ENSMUSG00000033306
Lppos	-1.868596254	0.21672045	6.57E-18	1.01E-15	ENSMUSG00000097772
Lrfn1	0.625713021	0.23326177	7.31E-03	3.23E-02	ENSMUSG00000030600
Lrg1	0.828678809	0.20497229	5.28E-05	6.00E-04	ENSMUSG00000037095
Lrig3	0.40338329	0.15465139	9.10E-03	3.82E-02	ENSMUSG00000020105
Lrp1	0.971128643	0.12952318	6.49E-14	6.06E-12	ENSMUSG00000040249
Lrp10	0.487577032	0.16082378	2.43E-03	1.34E-02	ENSMUSG00000022175
Lrp3	1.429385981	0.29440772	1.20E-06	2.37E-05	ENSMUSG00000001802
Lrp4	2.194168582	0.30299854	4.44E-13	3.84E-11	ENSMUSG00000027253
Lrp5	1.194765014	0.25190485	2.11E-06	3.87E-05	ENSMUSG00000024913
Lrp8	-0.838425997	0.32891186	1.08E-02	4.37E-02	ENSMUSG00000028613
Lrpap1	0.638910624	0.13387325	1.82E-06	3.44E-05	ENSMUSG00000029103
Lrrc14	0.850502718	0.1828733	3.31E-06	5.69E-05	ENSMUSG00000033728
Lrrc58	-0.753978348	0.21306225	4.02E-04	3.13E-03	ENSMUSG00000034158
Lrrc61	0.791433158	0.31453049	1.19E-02	4.70E-02	ENSMUSG00000073096
Lrrc73	1.223952819	0.37711108	1.17E-03	7.55E-03	ENSMUSG00000071073
Lrrfip1	-0.546068389	0.19115518	4.28E-03	2.11E-02	ENSMUSG00000026305
Lrrfip2	-1.018344605	0.19114127	9.95E-08	2.61E-06	ENSMUSG00000032497
Lrrn4cl	0.688213487	0.26264014	8.78E-03	3.72E-02	ENSMUSG00000071656
Lsm10	0.630903686	0.15698729	5.85E-05	6.51E-04	ENSMUSG00000050188
Lsm14a	-0.725685906	0.15168374	1.72E-06	3.26E-05	ENSMUSG00000066568
Lsm6	0.538852901	0.149845	3.23E-04	2.62E-03	ENSMUSG00000031683
Ltbp2	0.61662962	0.17827061	5.42E-04	4.01E-03	ENSMUSG00000002020
LTO1	0.847129931	0.25947084	1.10E-03	7.12E-03	ENSMUSG00000031072
Ltv1	-0.674514033	0.25405284	7.93E-03	3.44E-02	ENSMUSG00000019814
Luc7l	-0.456418842	0.15476527	3.19E-03	1.67E-02	ENSMUSG00000024188
Luc7l2	-0.529841711	0.16074924	9.80E-04	6.51E-03	ENSMUSG00000029823
Lxn	0.515935456	0.16054526	1.31E-03	8.21E-03	ENSMUSG00000047557
Ly6a	0.751877876	0.12907631	5.71E-09	2.11E-07	ENSMUSG00000075602
Ly6c1	0.479193525	0.13617769	4.33E-04	3.34E-03	ENSMUSG00000079018
Ly6e	0.493812885	0.1252418	8.05E-05	8.45E-04	ENSMUSG00000022587
Lyar	-1.080652978	0.22988817	2.59E-06	4.64E-05	ENSMUSG00000067367
Lynx1	1.066928019	0.163895	7.52E-11	4.48E-09	ENSMUSG00000022594
Lypla2	0.387625474	0.15263933	1.11E-02	4.47E-02	ENSMUSG00000028670
Lyrm2	0.399941915	0.1535561	9.20E-03	3.86E-02	ENSMUSG00000045854
Lysmd3	-1.233041392	0.36869715	8.25E-04	5.68E-03	ENSMUSG00000035840
Lyst	-1.024460301	0.33455707	2.20E-03	1.23E-02	ENSMUSG00000019726
Lyz2	1.103311703	0.16511899	2.36E-11	1.54E-09	ENSMUSG00000069516
Lztfl1	-1.011621671	0.30983505	1.09E-03	7.12E-03	ENSMUSG00000025245
M1ap	-0.897538057	0.23468721	1.31E-04	1.25E-03	ENSMUSG00000030041
Mad1l1	0.509805209	0.20062268	1.10E-02	4.45E-02	ENSMUSG00000029554
Maf	0.414049397	0.12365252	8.13E-04	5.61E-03	ENSMUSG00000055435
Mafb	0.829744127	0.18668786	8.81E-06	1.30E-04	ENSMUSG00000074622

Maff	-0.613597396	0.18482158	9.00E-04	6.11E-03	ENSMUSG00000042622
Maged1	0.401285658	0.11474374	4.70E-04	3.57E-03	ENSMUSG00000025151
Magee1	0.649543759	0.23450994	5.61E-03	2.62E-02	ENSMUSG00000031227
Magoh	-0.444634367	0.13851662	1.33E-03	8.29E-03	ENSMUSG00000028609
Mak16	-0.74299926	0.17206183	1.57E-05	2.16E-04	ENSMUSG00000031578
Malsu1	-0.462225722	0.15101459	2.21E-03	1.24E-02	ENSMUSG00000029815
Maml2	-0.691056984	0.26746379	9.77E-03	4.04E-02	ENSMUSG00000031925
Man1c1	0.791488921	0.19606354	5.42E-05	6.13E-04	ENSMUSG00000037306
Man2b1	0.879287491	0.17440095	4.61E-07	1.02E-05	ENSMUSG00000005142
Man2b2	0.858081857	0.2566063	8.26E-04	5.68E-03	ENSMUSG00000029119
Man2c1	0.665493451	0.22662075	3.32E-03	1.72E-02	ENSMUSG00000032295
Manf	0.302095787	0.11820245	1.06E-02	4.31E-02	ENSMUSG00000032575
Map11	0.818199874	0.23488867	4.95E-04	3.72E-03	ENSMUSG00000036948
Map1a	0.55430942	0.19448206	4.37E-03	2.15E-02	ENSMUSG00000027254
Map2k2	0.417722743	0.13460212	1.91E-03	1.11E-02	ENSMUSG00000035027
Map3k20	-0.692415374	0.21492832	1.27E-03	8.05E-03	ENSMUSG00000004085
Map3k8	-1.464890342	0.33615259	1.31E-05	1.84E-04	ENSMUSG00000024235
Map4k4	-0.648260926	0.10590014	9.27E-10	4.27E-08	ENSMUSG00000026074
Map7d1	0.584700111	0.13128879	8.45E-06	1.25E-04	ENSMUSG00000028849
Map7d2	-1.255585815	0.26943114	3.16E-06	5.48E-05	ENSMUSG00000041020
Mapk12	-0.996173734	0.31048835	1.33E-03	8.32E-03	ENSMUSG00000022610
Mapk3	0.385126995	0.11441401	7.62E-04	5.34E-03	ENSMUSG00000063065
Mapk6	-0.828267255	0.1984089	2.99E-05	3.69E-04	ENSMUSG00000042688
Mapk8ip1	1.219220512	0.34532472	4.15E-04	3.22E-03	ENSMUSG00000027223
1-Mar	1.10789273	0.44384643	1.26E-02	4.90E-02	ENSMUSG00000026621
2-Mar	0.330612313	0.13164263	1.20E-02	4.75E-02	ENSMUSG00000079557
7-Mar	-1.003224822	0.23915056	2.73E-05	3.42E-04	ENSMUSG00000026977
Marcks	0.422856322	0.13862486	2.29E-03	1.27E-02	ENSMUSG00000069662
Marf1	-1.115632361	0.30563139	2.62E-04	2.20E-03	ENSMUSG00000060657
Masp1	1.113342363	0.17588011	2.45E-10	1.30E-08	ENSMUSG00000022887
Matn4	0.4261557	0.17051684	1.24E-02	4.87E-02	ENSMUSG00000016995
Matr3	-0.956080468	0.25584359	1.86E-04	1.66E-03	ENSMUSG00000037236
Mavs	0.595654776	0.14597624	4.49E-05	5.24E-04	ENSMUSG00000037523
Mbnl2	-0.93208233	0.31502268	3.09E-03	1.62E-02	ENSMUSG00000022139
Mboat7	0.561620074	0.14830737	1.53E-04	1.41E-03	ENSMUSG00000035596
Mbtd1	-1.132224971	0.20848311	5.61E-08	1.59E-06	ENSMUSG00000059474
Mbtps1	0.727304199	0.11788591	6.85E-10	3.26E-08	ENSMUSG00000031835
Mcee	0.693067557	0.13419312	2.41E-07	5.69E-06	ENSMUSG00000033429
Mcm3	0.507110066	0.20304543	1.25E-02	4.89E-02	ENSMUSG00000041859
Mcm5	1.130876883	0.37859209	2.82E-03	1.51E-02	ENSMUSG00000005410
Mdfic	-0.80202127	0.19667495	4.54E-05	5.29E-04	ENSMUSG00000041390
Mdga1	1.052001813	0.29423967	3.50E-04	2.79E-03	ENSMUSG00000043557
Mdh1	0.390751912	0.12117768	1.26E-03	7.98E-03	ENSMUSG00000020321

Mdk	0.354201075	0.12981374	6.36E-03	2.89E-02	ENSMUSG00000027239
Mdm2	-0.880255318	0.14777112	2.57E-09	1.04E-07	ENSMUSG00000020184
Mdm4	-1.283926401	0.30346137	2.33E-05	2.99E-04	ENSMUSG00000054387
Mecp2	-0.538396489	0.2090602	1.00E-02	4.12E-02	ENSMUSG00000031393
Med1	-0.593622231	0.18916511	1.70E-03	1.01E-02	ENSMUSG00000018160
Med10	-0.550460879	0.12568383	1.19E-05	1.68E-04	ENSMUSG00000021598
Med13l	-0.553750738	0.1660981	8.56E-04	5.87E-03	ENSMUSG00000018076
Med25	0.615540953	0.18084763	6.65E-04	4.73E-03	ENSMUSG00000002968
Med27	0.561041347	0.15085972	2.00E-04	1.76E-03	ENSMUSG00000026799
Med7	-0.481696438	0.1838544	8.79E-03	3.72E-02	ENSMUSG00000020397
Medag	-1.090869501	0.1517471	6.54E-13	5.48E-11	ENSMUSG00000029659
Mef2b	-1.820440124	0.49663703	2.47E-04	2.10E-03	ENSMUSG00000079033
Mef2d	0.334185776	0.1314094	1.10E-02	4.43E-02	ENSMUSG00000001419
Meg3	-0.720208433	0.26377644	6.33E-03	2.88E-02	ENSMUSG00000021268
Megf6	1.403627088	0.3012112	3.16E-06	5.48E-05	ENSMUSG00000057751
Megf8	0.654224277	0.17784372	2.34E-04	2.01E-03	ENSMUSG00000045039
Meis3	0.660299937	0.17186671	1.22E-04	1.18E-03	ENSMUSG00000041420
Mest	1.224732177	0.18148775	1.50E-11	1.03E-09	ENSMUSG00000051855
Met	-2.291652306	0.42008763	4.89E-08	1.41E-06	ENSMUSG00000009376
Metap2	-0.441630333	0.15543481	4.49E-03	2.20E-02	ENSMUSG00000036112
Mettl1	-0.379632428	0.14883433	1.08E-02	4.36E-02	ENSMUSG00000006732
Mettl26	0.368806727	0.14524356	1.11E-02	4.47E-02	ENSMUSG00000025731
Mex3b	-2.129413249	0.43678628	1.09E-06	2.18E-05	ENSMUSG00000057706
Mfap1a	-0.984115746	0.36775155	7.45E-03	3.28E-02	ENSMUSG00000068479
Mfap1b	-0.704471602	0.26890733	8.80E-03	3.72E-02	ENSMUSG00000048222
Mfap2	0.939397757	0.24313738	1.12E-04	1.09E-03	ENSMUSG00000060572
Mfn2	0.558365122	0.18225166	2.19E-03	1.23E-02	ENSMUSG00000029020
Mfsd10	0.576161177	0.13301236	1.48E-05	2.04E-04	ENSMUSG00000001082
Mfsd11	0.749209413	0.17724729	2.37E-05	3.03E-04	ENSMUSG00000020818
Mfsd3	1.353700432	0.25371734	9.53E-08	2.53E-06	ENSMUSG00000019080
Mfsd4b4	0.314120434	0.10252361	2.18E-03	1.23E-02	ENSMUSG00000096210
Mfsd5	0.319642352	0.11282457	4.61E-03	2.24E-02	ENSMUSG00000045665
Mfsd6	-2.272846814	0.45678403	6.50E-07	1.38E-05	ENSMUSG00000041439
Mga	-1.367551904	0.27850203	9.09E-07	1.86E-05	ENSMUSG00000033943
Mgarp	1.208101982	0.20378894	3.06E-09	1.21E-07	ENSMUSG00000037161
Mgat1	0.516337279	0.16561006	1.82E-03	1.06E-02	ENSMUSG00000020346
Mgat2	0.641330353	0.25600191	1.22E-02	4.81E-02	ENSMUSG00000043998
Mgat3	2.295023058	0.65958736	5.02E-04	3.76E-03	ENSMUSG00000042428
Mgll	1.402846808	0.19613892	8.53E-13	6.93E-11	ENSMUSG00000033174
Mgmt	0.589393552	0.17072986	5.56E-04	4.09E-03	ENSMUSG00000054612
Mgp	0.540047664	0.11960671	6.33E-06	9.88E-05	ENSMUSG00000030218
Mgrn1	0.868415709	0.15121516	9.31E-09	3.33E-07	ENSMUSG00000022517
Mgst1	0.840119617	0.13752807	1.00E-09	4.49E-08	ENSMUSG00000008540

Mgst3	1.31802306	0.20474366	1.22E-10	6.81E-09	ENSMUSG00000026688
Mia2	-1.055383771	0.20849524	4.15E-07	9.29E-06	ENSMUSG00000021000
Mia3	-0.604004452	0.20698073	3.52E-03	1.81E-02	ENSMUSG00000056050
Miat	1.717083012	0.67614531	1.11E-02	4.47E-02	ENSMUSG00000097750
Mib1	-0.841263807	0.24654133	6.44E-04	4.62E-03	ENSMUSG00000024294
Mical1	0.58598089	0.20442341	4.15E-03	2.06E-02	ENSMUSG00000019823
Midn	0.503126419	0.12362745	4.71E-05	5.45E-04	ENSMUSG00000035621
Mier1	-0.596598228	0.22694405	8.57E-03	3.65E-02	ENSMUSG00000028522
Mif4gd	0.914100847	0.19792592	3.87E-06	6.51E-05	ENSMUSG00000020743
Minpp1	0.994208822	0.36914211	7.07E-03	3.15E-02	ENSMUSG00000024896
Mipol1	-1.342781674	0.32090836	2.86E-05	3.56E-04	ENSMUSG00000047022
Mir5136	0.674230003	0.24586882	6.10E-03	2.80E-02	ENSMUSG00000092486
Mir703	-0.710235595	0.22833744	1.87E-03	1.09E-02	ENSMUSG00000105748
Mkln1	-0.652659872	0.23040462	4.62E-03	2.24E-02	ENSMUSG00000025609
Mkrn2	-0.534875103	0.18615756	4.06E-03	2.02E-02	ENSMUSG00000000439
Mlec	0.549372021	0.12387973	9.22E-06	1.35E-04	ENSMUSG00000048578
Mlf1	-2.137699957	0.65096697	1.02E-03	6.72E-03	ENSMUSG00000048416
Mllt3	-1.008235081	0.3093341	1.12E-03	7.24E-03	ENSMUSG00000028496
Mlph	1.655538712	0.65405288	1.14E-02	4.54E-02	ENSMUSG00000026303
Mlst8	0.873266546	0.26418214	9.48E-04	6.34E-03	ENSMUSG00000024142
Mmgt1	-0.618255388	0.24300114	1.10E-02	4.42E-02	ENSMUSG00000061273
Mmp11	0.514592282	0.16030427	1.33E-03	8.29E-03	ENSMUSG00000000901
Mmp14	0.617248014	0.12905978	1.73E-06	3.28E-05	ENSMUSG00000000957
Mmp19	0.700778011	0.1307839	8.40E-08	2.28E-06	ENSMUSG00000025355
Mmp2	0.35751808	0.10264481	4.96E-04	3.72E-03	ENSMUSG00000031740
Mmp23	0.577083422	0.19851499	3.65E-03	1.85E-02	ENSMUSG00000029061
Mmp3	-1.638043581	0.40255746	4.72E-05	5.45E-04	ENSMUSG00000043613
Mob4	-0.573684499	0.14496057	7.57E-05	8.03E-04	ENSMUSG00000025979
Mocs3	0.687468308	0.23672968	3.68E-03	1.87E-02	ENSMUSG00000074576
Mon1a	0.868146941	0.22979763	1.58E-04	1.45E-03	ENSMUSG00000032583
Mon2	-0.609113582	0.1739643	4.63E-04	3.52E-03	ENSMUSG00000034602
Morc3	-1.074294981	0.33980069	1.57E-03	9.45E-03	ENSMUSG00000039456
Morc4	-0.581762546	0.21942908	8.02E-03	3.47E-02	ENSMUSG00000031434
Morf4l1	-1.300563995	0.2057452	2.59E-10	1.36E-08	ENSMUSG00000062270
Morn2	1.33210542	0.23561962	1.57E-08	5.27E-07	ENSMUSG00000045257
Mospd1	-1.07567301	0.26539817	5.06E-05	5.77E-04	ENSMUSG00000023074
Mpc2	0.349080478	0.1142606	2.25E-03	1.26E-02	ENSMUSG00000026568
Mpdu1	0.739912562	0.14142876	1.68E-07	4.17E-06	ENSMUSG00000018761
Mpdz	-1.259868176	0.42335905	2.92E-03	1.55E-02	ENSMUSG00000028402
Mpg	0.497881697	0.18810684	8.13E-03	3.49E-02	ENSMUSG00000020287
Mphosph10	-1.088123143	0.34394854	1.56E-03	9.39E-03	ENSMUSG00000030521
Mphosph8	-1.300916795	0.26330102	7.78E-07	1.62E-05	ENSMUSG00000079184
Mpp5	-1.438234226	0.30293363	2.06E-06	3.81E-05	ENSMUSG00000021112

Mpp6	-0.957454593	0.22052453	1.41E-05	1.96E-04	ENSMUSG00000038388
Mprip	0.385781941	0.10498975	2.38E-04	2.04E-03	ENSMUSG00000005417
Mrc2	0.758758395	0.13307759	1.19E-08	4.15E-07	ENSMUSG00000020695
Mrgprf	1.476031946	0.23484559	3.28E-10	1.66E-08	ENSMUSG00000031070
Mrpl1	-1.642138674	0.30447967	6.92E-08	1.93E-06	ENSMUSG00000029486
Mrpl12	-0.433120641	0.12476703	5.18E-04	3.85E-03	ENSMUSG00000039640
Mrpl13	-0.328207864	0.13051781	1.19E-02	4.71E-02	ENSMUSG00000022370
Mrpl20	0.545548074	0.11380549	1.64E-06	3.12E-05	ENSMUSG00000029066
Mrpl30	-0.530004056	0.11910973	8.60E-06	1.27E-04	ENSMUSG00000026087
Mrpl32	-0.447801057	0.15072309	2.97E-03	1.57E-02	ENSMUSG00000015672
Mrpl35	-0.397846753	0.12840448	1.95E-03	1.12E-02	ENSMUSG00000052962
Mrpl46	-0.537758849	0.19833958	6.70E-03	3.02E-02	ENSMUSG00000030612
Mrpl47	-0.997050635	0.18013705	3.11E-08	9.60E-07	ENSMUSG00000037531
Mrpl51	0.434902333	0.1436973	2.47E-03	1.36E-02	ENSMUSG00000030335
Mrpl54	0.404846303	0.13768316	3.28E-03	1.70E-02	ENSMUSG00000034932
Mrps11	0.646212921	0.21977137	3.28E-03	1.70E-02	ENSMUSG00000030611
Mrps12	0.558047475	0.14607879	1.33E-04	1.26E-03	ENSMUSG00000045948
Mrps22	-0.460553824	0.15969647	3.93E-03	1.97E-02	ENSMUSG00000032459
Mrps30	-0.369310452	0.13747519	7.22E-03	3.21E-02	ENSMUSG00000021731
Mrps9	-0.579397312	0.16756846	5.45E-04	4.02E-03	ENSMUSG00000060679
Mrto4	-0.576948706	0.13260946	1.36E-05	1.89E-04	ENSMUSG00000028741
Ms4a6b	2.346961374	0.53225824	1.04E-05	1.50E-04	ENSMUSG00000024677
Ms4a6c	2.107702538	0.48421938	1.34E-05	1.88E-04	ENSMUSG00000079419
Ms4a6d	1.536628317	0.24130015	1.91E-10	1.03E-08	ENSMUSG00000024679
Ms4a8a	2.638154861	0.77487031	6.63E-04	4.72E-03	ENSMUSG00000024730
Msh5	2.843699393	0.81942179	5.20E-04	3.87E-03	ENSMUSG00000007035
Msi2	-0.386755182	0.15232286	1.11E-02	4.47E-02	ENSMUSG00000069769
Msl2	-0.789290229	0.30916835	1.07E-02	4.34E-02	ENSMUSG00000066415
Msmo1	-0.397571981	0.1320902	2.61E-03	1.42E-02	ENSMUSG00000031604
Msn	-0.263437351	0.10389779	1.12E-02	4.50E-02	ENSMUSG00000031207
Msra	1.16927587	0.3670476	1.44E-03	8.87E-03	ENSMUSG00000054733
Msrb1	0.642789089	0.14489806	9.16E-06	1.35E-04	ENSMUSG00000075705
Msrb2	0.791598629	0.30540238	9.54E-03	3.97E-02	ENSMUSG00000023094
mt-Cytb	-0.341550829	0.13360509	1.06E-02	4.30E-02	ENSMUSG00000064370
mt-Nd4	-0.508718187	0.15118	7.65E-04	5.35E-03	ENSMUSG00000064363
mt-Nd6	-0.313128904	0.10695392	3.41E-03	1.76E-02	ENSMUSG00000064368
mt-Ti	0.439944562	0.16605233	8.06E-03	3.48E-02	ENSMUSG00000064342
mt-Tl2	0.334739741	0.11490253	3.58E-03	1.83E-02	ENSMUSG00000064366
Mt1	-1.196290924	0.09859964	7.08E-34	4.90E-31	ENSMUSG00000031765
Mt2	-1.215324929	0.09633213	1.72E-36	1.51E-33	ENSMUSG00000031762
Mt3	-1.596386335	0.49722397	1.32E-03	8.28E-03	ENSMUSG00000031760
Mtap	-0.576540344	0.1599999	3.14E-04	2.56E-03	ENSMUSG00000062937
Mtch1	0.61375214	0.11199806	4.25E-08	1.26E-06	ENSMUSG00000024012

Mtdh	-0.917546577	0.183573	5.78E-07	1.25E-05	ENSMUSG00000022255
Mthfd2	-0.354533286	0.13041346	6.56E-03	2.96E-02	ENSMUSG00000005667
Mthfr	0.7549149	0.23511691	1.32E-03	8.28E-03	ENSMUSG00000029009
Mthfs	0.839771365	0.29940303	5.03E-03	2.41E-02	ENSMUSG00000066442
Mthfsd	0.76728387	0.19314354	7.11E-05	7.66E-04	ENSMUSG00000031816
Mtln	-0.417103766	0.13228623	1.62E-03	9.68E-03	ENSMUSG00000051319
Mtm1	-1.308238777	0.48393814	6.87E-03	3.08E-02	ENSMUSG00000031337
Mtmr11	0.690717165	0.24431566	4.70E-03	2.28E-02	ENSMUSG00000045934
Mtmr6	-0.738176901	0.22325243	9.45E-04	6.33E-03	ENSMUSG00000021987
Mtpap	-0.752424157	0.29477357	1.07E-02	4.34E-02	ENSMUSG00000024234
Mtpn	-0.534177965	0.12915529	3.54E-05	4.25E-04	ENSMUSG00000029840
Mtrex	-0.883114437	0.17164209	2.67E-07	6.25E-06	ENSMUSG00000016018
Mtss1l	0.702225376	0.161691	1.41E-05	1.95E-04	ENSMUSG00000033763
Mturn	0.960903005	0.33962299	4.66E-03	2.26E-02	ENSMUSG00000038065
Mvk	1.077454103	0.2065177	1.82E-07	4.46E-06	ENSMUSG00000041939
Mxd4	0.804730601	0.1389076	6.90E-09	2.51E-07	ENSMUSG00000037235
Mxra8	0.942907874	0.12295904	1.74E-14	1.72E-12	ENSMUSG00000029070
Mycbp2	-1.17528879	0.22099357	1.05E-07	2.74E-06	ENSMUSG00000033004
Myh9	0.488693019	0.12560203	9.99E-05	1.00E-03	ENSMUSG00000022443
Myl6	0.723333314	0.19306294	1.79E-04	1.61E-03	ENSMUSG00000090841
Myl6b	1.465733449	0.5450745	7.17E-03	3.18E-02	ENSMUSG00000039824
Myl9	0.727193532	0.15296627	1.99E-06	3.71E-05	ENSMUSG00000067818
Mylk	1.202627066	0.20214955	2.69E-09	1.07E-07	ENSMUSG00000022836
Myo18a	0.4294078	0.16055845	7.48E-03	3.29E-02	ENSMUSG00000000631
Myo1d	0.711368188	0.12190403	5.36E-09	2.00E-07	ENSMUSG00000035441
Myo1e	0.621128414	0.15895453	9.32E-05	9.51E-04	ENSMUSG00000032220
Myo5a	-0.954259543	0.23702603	5.67E-05	6.36E-04	ENSMUSG00000034593
Myo6	-0.819970373	0.20706113	7.49E-05	7.99E-04	ENSMUSG00000033577
Myof	-0.500121993	0.19706196	1.12E-02	4.48E-02	ENSMUSG00000048612
Mzt1	-0.98923315	0.30589755	1.22E-03	7.80E-03	ENSMUSG00000033186
n-R5-8s1	-1.811125142	0.59629424	2.39E-03	1.32E-02	ENSMUSG00000065922
N4bp2l1	-1.010560045	0.2410467	2.76E-05	3.45E-04	ENSMUSG00000041132
N4bp2l2	-0.766593638	0.19723432	1.02E-04	1.01E-03	ENSMUSG00000029655
Naa15	-0.475342177	0.15108068	1.65E-03	9.89E-03	ENSMUSG00000063273
Naa16	-0.869067848	0.28500261	2.29E-03	1.28E-02	ENSMUSG00000022020
Naa25	-1.129270757	0.21677533	1.89E-07	4.63E-06	ENSMUSG00000042719
Naa30	-0.71909007	0.23863511	2.58E-03	1.41E-02	ENSMUSG00000036282
Naa50	-1.118275628	0.14550127	1.52E-14	1.52E-12	ENSMUSG00000022698
Naa60	0.392974402	0.15216483	9.81E-03	4.05E-02	ENSMUSG00000005982
Nab1	-1.238718047	0.24167276	2.97E-07	6.83E-06	ENSMUSG00000002881
Nab2	0.672403004	0.17945918	1.79E-04	1.61E-03	ENSMUSG00000025402
Naca	-0.65881557	0.1770575	1.99E-04	1.75E-03	ENSMUSG00000061315
Nacc2	0.967833056	0.17431204	2.82E-08	8.77E-07	ENSMUSG00000026932

Nae1	-0.805624901	0.22896083	4.34E-04	3.34E-03	ENSMUSG00000031878
Nagk	0.922556563	0.14333108	1.22E-10	6.82E-09	ENSMUSG00000034744
Naglu	0.642998206	0.1276662	4.74E-07	1.04E-05	ENSMUSG00000001751
Nagpa	0.798868489	0.17714439	6.49E-06	1.01E-04	ENSMUSG00000023143
Nampt	-0.786035706	0.15552276	4.32E-07	9.61E-06	ENSMUSG00000020572
Nap1l1	-0.91471332	0.14987443	1.04E-09	4.59E-08	ENSMUSG00000058799
Napb	-1.246801241	0.41977597	2.98E-03	1.58E-02	ENSMUSG00000027438
Nars	-0.637946587	0.12195908	1.69E-07	4.18E-06	ENSMUSG00000024587
Nat8f1	1.791397319	0.388881	4.09E-06	6.83E-05	ENSMUSG00000057103
Nbl1	0.532449127	0.13028445	4.37E-05	5.13E-04	ENSMUSG00000041120
Nbr1	-0.457719434	0.16966847	6.98E-03	3.12E-02	ENSMUSG00000017119
Ncam1	0.489001151	0.16848451	3.70E-03	1.88E-02	ENSMUSG00000039542
Ncbp2	0.501543064	0.18570242	6.92E-03	3.10E-02	ENSMUSG00000022774
Ncdn	0.655113475	0.21877424	2.75E-03	1.48E-02	ENSMUSG00000028833
Ncf1	1.683933559	0.329643	3.25E-07	7.45E-06	ENSMUSG00000015950
Nck2	-0.714073661	0.20185827	4.04E-04	3.14E-03	ENSMUSG00000066877
Nckap1	-0.735829597	0.17162814	1.81E-05	2.41E-04	ENSMUSG00000027002
Ncl	-1.149352595	0.17104113	1.82E-11	1.23E-09	ENSMUSG00000026234
Ncln	0.47657207	0.16899394	4.80E-03	2.32E-02	ENSMUSG00000020238
Ncoa7	-0.792311254	0.28581896	5.57E-03	2.61E-02	ENSMUSG00000039697
Ncor1	-0.86419996	0.14713313	4.26E-09	1.63E-07	ENSMUSG00000018501
Ncor2	0.642528568	0.14936893	1.70E-05	2.29E-04	ENSMUSG00000029478
Ncstn	0.649713562	0.23674364	6.06E-03	2.78E-02	ENSMUSG00000003458
Ndel1	-0.456369446	0.13981851	1.10E-03	7.14E-03	ENSMUSG00000018736
Ndn	0.779778005	0.17209303	5.87E-06	9.27E-05	ENSMUSG00000033585
Ndnf	3.854563605	1.01745967	1.52E-04	1.40E-03	ENSMUSG00000049001
Ndrg2	1.249687106	0.12458274	1.11E-23	3.58E-21	ENSMUSG00000004558
Ndrg3	0.64028398	0.15760691	4.85E-05	5.57E-04	ENSMUSG00000027634
Ndst1	0.489274355	0.15774692	1.92E-03	1.11E-02	ENSMUSG00000054008
Ndufa1	0.442312027	0.14371769	2.09E-03	1.18E-02	ENSMUSG00000016427
Ndufa13	0.242131825	0.09732195	1.28E-02	4.98E-02	ENSMUSG00000036199
Ndufa3	0.400926319	0.09618892	3.07E-05	3.79E-04	ENSMUSG00000035674
Ndufa4l2	1.934263135	0.10873028	8.52E-71	2.80E-67	ENSMUSG00000040280
Ndufaf4	-0.502276292	0.17840236	4.87E-03	2.35E-02	ENSMUSG00000028261
Ndufb2	0.658120971	0.12301213	8.79E-08	2.36E-06	ENSMUSG00000002416
Ndufb7	0.45058168	0.13272914	6.87E-04	4.86E-03	ENSMUSG00000033938
Ndufc1	0.330603432	0.10061319	1.02E-03	6.69E-03	ENSMUSG00000037152
Ndufv3	0.787667812	0.12109011	7.78E-11	4.61E-09	ENSMUSG00000024038
Nedd1	-1.000646627	0.33103255	2.50E-03	1.37E-02	ENSMUSG00000019988
Nedd4l	-0.71917325	0.17149796	2.75E-05	3.44E-04	ENSMUSG00000024589
Nek7	-0.742607152	0.22694997	1.07E-03	6.97E-03	ENSMUSG00000026393
Nemf	-1.098398979	0.28595828	1.22E-04	1.18E-03	ENSMUSG00000020982
Nenf	0.468875045	0.12517674	1.80E-04	1.61E-03	ENSMUSG00000037499

Neo1	0.596213071	0.15127189	8.10E-05	8.49E-04	ENSMUSG00000032340
Neurl2	1.94821265	0.53361902	2.61E-04	2.20E-03	ENSMUSG00000039873
Nfam1	1.899489768	0.57808365	1.02E-03	6.69E-03	ENSMUSG00000058099
Nfatc4	0.7232916	0.14365764	4.78E-07	1.05E-05	ENSMUSG00000023411
Nfe2l2	-0.899073974	0.14784347	1.19E-09	5.22E-08	ENSMUSG00000015839
Nfib	-0.584442472	0.1924242	2.39E-03	1.32E-02	ENSMUSG00000008575
Nfix	0.639618269	0.13328019	1.59E-06	3.05E-05	ENSMUSG00000001911
Nfkbib	-0.470872109	0.14533992	1.20E-03	7.68E-03	ENSMUSG00000030595
Nfkbie	1.20177609	0.43117363	5.32E-03	2.51E-02	ENSMUSG00000023947
Nfkbiz	-0.806872785	0.22683638	3.75E-04	2.95E-03	ENSMUSG00000035356
Ngf	-1.284907039	0.1910356	1.74E-11	1.18E-09	ENSMUSG00000027859
Nhej1	1.050586483	0.37296212	4.85E-03	2.34E-02	ENSMUSG00000026162
Nhp2	-0.447137277	0.12039182	2.04E-04	1.79E-03	ENSMUSG00000001056
Nhs	-1.336163488	0.37797787	4.08E-04	3.17E-03	ENSMUSG00000059493
Nhsl1	1.627871439	0.54303183	2.72E-03	1.47E-02	ENSMUSG00000039835
Nifk	-0.45297239	0.16927726	7.45E-03	3.28E-02	ENSMUSG00000026377
Nipa2	-0.662825677	0.22059543	2.66E-03	1.44E-02	ENSMUSG00000030452
Nipbl	-1.006823299	0.27490291	2.50E-04	2.11E-03	ENSMUSG00000022141
Nipsnap2	0.435633792	0.1401653	1.88E-03	1.09E-02	ENSMUSG00000029432
Nisch	0.355488584	0.11906235	2.83E-03	1.51E-02	ENSMUSG00000021910
Nkapd1	-1.266979677	0.30766721	3.82E-05	4.55E-04	ENSMUSG00000059820
Nkrf	-1.460197074	0.48666785	2.70E-03	1.46E-02	ENSMUSG00000044149
Nktr	-0.94464611	0.23859574	7.52E-05	8.00E-04	ENSMUSG00000032525
Nlk	-0.94530169	0.36263465	9.14E-03	3.84E-02	ENSMUSG00000017376
Nlrx1	0.927199516	0.29282719	1.54E-03	9.33E-03	ENSMUSG00000032109
Nmb	1.113014532	0.29245879	1.41E-04	1.33E-03	ENSMUSG00000025723
Nmd3	-1.011606443	0.2437535	3.32E-05	4.06E-04	ENSMUSG00000027787
Nme4	1.214005187	0.16457287	1.62E-13	1.48E-11	ENSMUSG00000024177
Nme5	1.295078499	0.43543491	2.94E-03	1.56E-02	ENSMUSG00000035984
Nmrk1	0.559691207	0.19380049	3.88E-03	1.95E-02	ENSMUSG00000037847
Nmt2	-0.974151979	0.22289821	1.24E-05	1.74E-04	ENSMUSG00000026643
Noc2l	1.059381315	0.34673877	2.25E-03	1.26E-02	ENSMUSG00000095440
Nol7	-0.52241657	0.19454885	7.25E-03	3.21E-02	ENSMUSG00000063200
Nol8	-1.287531967	0.31452055	4.25E-05	5.01E-04	ENSMUSG00000021392
Nolc1	-0.605687626	0.18026495	7.79E-04	5.43E-03	ENSMUSG00000015176
Nop14	-1.303243936	0.28135331	3.62E-06	6.17E-05	ENSMUSG00000036693
Nop16	-0.376225954	0.12248217	2.13E-03	1.20E-02	ENSMUSG00000025869
Nop58	-1.294291446	0.2170188	2.46E-09	1.00E-07	ENSMUSG00000026020
Notch4	2.035892046	0.64339544	1.55E-03	9.37E-03	ENSMUSG00000015468
Notum	0.986092195	0.28831382	6.26E-04	4.50E-03	ENSMUSG00000042988
Npdc1	0.337864676	0.12211237	5.66E-03	2.64E-02	ENSMUSG00000015094
Npepl1	0.506619365	0.18565358	6.36E-03	2.89E-02	ENSMUSG00000039263
Npm1	-1.136944425	0.15962679	1.06E-12	8.51E-11	ENSMUSG00000057113

Npr2	0.788984597	0.14102656	2.21E-08	7.07E-07	ENSMUSG00000028469
Npy	-1.033795995	0.28466594	2.82E-04	2.33E-03	ENSMUSG00000029819
Nr1d1	-1.093104758	0.17343363	2.92E-10	1.52E-08	ENSMUSG00000020889
Nr1d2	-1.122009588	0.18615167	1.67E-09	7.03E-08	ENSMUSG00000021775
Nr1h3	1.206894119	0.36449246	9.29E-04	6.27E-03	ENSMUSG00000002108
Nr2c2	-0.683914742	0.19767262	5.41E-04	4.00E-03	ENSMUSG00000005893
Nr2c2ap	-0.617408652	0.15351011	5.77E-05	6.44E-04	ENSMUSG00000071078
Nr2f6	0.495568801	0.17365286	4.32E-03	2.13E-02	ENSMUSG00000002393
Nradd	0.496278172	0.1668777	2.94E-03	1.56E-02	ENSMUSG00000032491
Nrbp1	0.586163574	0.19903719	3.23E-03	1.68E-02	ENSMUSG00000029148
Nrbp2	0.756013423	0.24285898	1.85E-03	1.08E-02	ENSMUSG00000075590
Nrd1	-0.674268217	0.1721278	8.96E-05	9.19E-04	ENSMUSG00000053510
Nrgn	2.060237796	0.26650287	1.07E-14	1.11E-12	ENSMUSG00000053310
Nrip2	1.055391129	0.20681038	3.34E-07	7.65E-06	ENSMUSG00000001520
Nrk	-1.605939345	0.53269228	2.57E-03	1.41E-02	ENSMUSG00000052854
Nrros	1.223940647	0.32231996	1.46E-04	1.36E-03	ENSMUSG00000052384
Nsd3	-0.603175171	0.16177217	1.93E-04	1.71E-03	ENSMUSG00000054823
Nsdhl	0.685603423	0.21621254	1.52E-03	9.22E-03	ENSMUSG00000031349
Nsmce3	0.588979799	0.21379409	5.87E-03	2.72E-02	ENSMUSG00000070520
Nsmf	0.459372665	0.18171566	1.15E-02	4.57E-02	ENSMUSG00000006476
Nsrp1	-1.182025091	0.28530778	3.43E-05	4.15E-04	ENSMUSG00000037958
Nsun2	-0.948712593	0.14223291	2.56E-11	1.66E-09	ENSMUSG00000021595
Nt5e	-1.416497249	0.21691136	6.56E-11	3.95E-09	ENSMUSG00000032420
Ntmt1	-0.520856363	0.15227565	6.25E-04	4.50E-03	ENSMUSG00000026857
Ntpcr	0.479881746	0.16649633	3.95E-03	1.98E-02	ENSMUSG00000031851
Nuak1	0.868703695	0.13312913	6.79E-11	4.06E-09	ENSMUSG00000020032
Nucb1	0.469828841	0.11941807	8.34E-05	8.67E-04	ENSMUSG00000030824
Nucks1	-0.501912035	0.17819248	4.85E-03	2.34E-02	ENSMUSG00000026434
Nudcd1	-1.114188801	0.33613833	9.18E-04	6.20E-03	ENSMUSG00000038736
Nudcd2	-0.766731906	0.1466158	1.70E-07	4.21E-06	ENSMUSG00000020328
Nudt1	0.609117361	0.21997415	5.62E-03	2.63E-02	ENSMUSG00000036639
Nudt16l1	0.634851843	0.18465507	5.86E-04	4.28E-03	ENSMUSG00000022516
Nudt18	0.687993867	0.23050392	2.84E-03	1.52E-02	ENSMUSG00000045211
Nufip1	-0.646214607	0.24993584	9.72E-03	4.03E-02	ENSMUSG00000022009
Nufip2	-0.836054538	0.15089394	3.01E-08	9.33E-07	ENSMUSG00000037857
Nup62	-0.745898182	0.29226649	1.07E-02	4.35E-02	ENSMUSG00000108621
Nupr1	-0.333745279	0.11402873	3.42E-03	1.76E-02	ENSMUSG00000030717
Nus1	-0.963590509	0.12032869	1.17E-15	1.40E-13	ENSMUSG00000023068
Nxt1	-0.452857019	0.15360432	3.20E-03	1.67E-02	ENSMUSG00000036992
Nynrin	1.134941644	0.17480718	8.44E-11	4.94E-09	ENSMUSG00000075592
Oaf	0.50371196	0.15875866	1.51E-03	9.18E-03	ENSMUSG00000032014
Ocel1	0.822510749	0.2632219	1.78E-03	1.05E-02	ENSMUSG00000002396
Odf2l	-1.905207162	0.75993584	1.22E-02	4.79E-02	ENSMUSG00000028256

Odr4	-1.008044449	0.27326383	2.25E-04	1.94E-03	ENSMUSG00000006010
Oga	-0.837773573	0.20203908	3.37E-05	4.11E-04	ENSMUSG00000025220
Ogdh	0.460129446	0.13663207	7.58E-04	5.31E-03	ENSMUSG00000020456
Ola1	-1.025315476	0.18567083	3.35E-08	1.02E-06	ENSMUSG00000027108
Olfml2a	1.585393149	0.40183933	7.97E-05	8.37E-04	ENSMUSG00000046618
Olfml2b	1.865613434	0.14393218	2.02E-38	2.41E-35	ENSMUSG00000038463
Olfml3	2.041730017	0.11136252	4.42E-75	1.94E-71	ENSMUSG00000027848
Omd	1.179664337	0.21586079	4.63E-08	1.34E-06	ENSMUSG00000048368
Onecut2	-2.657556517	0.83954388	1.55E-03	9.35E-03	ENSMUSG00000045991
Oplah	0.708608963	0.17194112	3.77E-05	4.50E-04	ENSMUSG00000022562
Orai1	0.681257544	0.2699056	1.16E-02	4.61E-02	ENSMUSG00000049686
Orai3	0.506018435	0.15654482	1.23E-03	7.83E-03	ENSMUSG00000043964
Orc4	-1.33134589	0.20635083	1.10E-10	6.25E-09	ENSMUSG00000026761
Orc6	-0.709806325	0.27882356	1.09E-02	4.41E-02	ENSMUSG00000031697
Os9	0.496675195	0.12009747	3.54E-05	4.26E-04	ENSMUSG00000040462
Osbpl2	-0.494555123	0.16464471	2.67E-03	1.45E-02	ENSMUSG00000039050
Osbpl7	1.018320825	0.3245796	1.70E-03	1.01E-02	ENSMUSG00000038534
Osbpl8	-0.836326353	0.30367435	5.89E-03	2.72E-02	ENSMUSG00000020189
Osbpl9	-0.693060694	0.18431815	1.70E-04	1.54E-03	ENSMUSG00000028559
Osmr	-1.018341803	0.24355653	2.90E-05	3.60E-04	ENSMUSG00000022146
Otud4	-0.868119322	0.22140915	8.82E-05	9.07E-04	ENSMUSG00000036990
Otud6b	-0.658226757	0.25277197	9.21E-03	3.86E-02	ENSMUSG00000040550
Ovgp1	-1.049911974	0.26876008	9.36E-05	9.54E-04	ENSMUSG00000074340
P2rx2	2.316714485	0.67331483	5.80E-04	4.24E-03	ENSMUSG00000029503
P2rx5	1.835303172	0.53003886	5.35E-04	3.97E-03	ENSMUSG00000005950
P2rx6	1.999018176	0.76568588	9.03E-03	3.80E-02	ENSMUSG00000022758
P3h4	0.372743479	0.11012886	7.13E-04	5.03E-03	ENSMUSG00000006931
P4ha2	0.725895772	0.11790276	7.43E-10	3.48E-08	ENSMUSG00000018906
P4ha3	0.678150175	0.19965497	6.82E-04	4.83E-03	ENSMUSG00000051048
P4hb	0.371423812	0.10888774	6.47E-04	4.64E-03	ENSMUSG00000025130
Pa2g4	-0.391354681	0.12990314	2.59E-03	1.41E-02	ENSMUSG00000025364
Pabpc1	-0.755844364	0.17433347	1.45E-05	2.01E-04	ENSMUSG00000022283
Pabpc4	-0.28163782	0.10218062	5.85E-03	2.71E-02	ENSMUSG00000011257
Pafah1b1	-0.473527911	0.12068456	8.72E-05	8.98E-04	ENSMUSG00000020745
Pafah1b3	0.72693915	0.17255994	2.52E-05	3.20E-04	ENSMUSG00000005447
Pafah2	1.030663774	0.179537	9.43E-09	3.36E-07	ENSMUSG00000037366
Paics	-0.378549957	0.14208704	7.72E-03	3.37E-02	ENSMUSG00000029247
Paip2	-0.502056789	0.15230897	9.80E-04	6.51E-03	ENSMUSG00000037058
Pak1ip1	-0.445296182	0.12928869	5.73E-04	4.20E-03	ENSMUSG00000038683
Pak2	-0.601535744	0.18598348	1.22E-03	7.79E-03	ENSMUSG00000022781
Panx3	2.946413505	0.20464351	5.34E-47	1.17E-43	ENSMUSG00000011118
Papola	-0.717020079	0.13912159	2.55E-07	6.00E-06	ENSMUSG00000021111
Papss2	-0.816571774	0.22217216	2.37E-04	2.03E-03	ENSMUSG00000024899

Paqr7	0.716890228	0.24066395	2.89E-03	1.54E-02	ENSMUSG00000037348
Parp3	0.530933778	0.18355306	3.82E-03	1.92E-02	ENSMUSG00000023249
Parva	0.331294527	0.11951803	5.57E-03	2.61E-02	ENSMUSG00000030770
Paxbp1	-1.162574637	0.27603296	2.53E-05	3.21E-04	ENSMUSG00000022974
Pbrm1	-0.703183742	0.22811272	2.05E-03	1.17E-02	ENSMUSG00000042323
Pbx1	-0.812338825	0.18927808	1.77E-05	2.38E-04	ENSMUSG00000052534
Pbxip1	0.94770475	0.12324065	1.47E-14	1.48E-12	ENSMUSG00000042613
Pcbd2	0.399165169	0.14634293	6.38E-03	2.90E-02	ENSMUSG00000021496
Pcbp4	0.679876767	0.11526421	3.67E-09	1.42E-07	ENSMUSG00000023495
Pccb	0.98804835	0.15677877	2.93E-10	1.52E-08	ENSMUSG00000032527
Pcf11	-1.344431182	0.23138263	6.23E-09	2.28E-07	ENSMUSG00000041328
Pcgf6	-1.322263967	0.45722784	3.83E-03	1.93E-02	ENSMUSG00000025050
Pck2	-0.329672448	0.12025064	6.12E-03	2.80E-02	ENSMUSG00000040618
Pcnp	-0.872445061	0.21839575	6.48E-05	7.11E-04	ENSMUSG00000071533
Pcnx3	0.851323593	0.19426005	1.17E-05	1.66E-04	ENSMUSG00000054874
Pcolce	1.098236659	0.11476575	1.08E-21	2.40E-19	ENSMUSG00000029718
Pcolce2	0.691374367	0.15814751	1.23E-05	1.74E-04	ENSMUSG00000015354
Pcp4l1	1.688645814	0.42793515	7.95E-05	8.35E-04	ENSMUSG00000038370
Pcsk6	1.245433666	0.21064631	3.37E-09	1.32E-07	ENSMUSG00000030513
Pcx	0.98567915	0.20164524	1.02E-06	2.06E-05	ENSMUSG00000024892
Pcyox1	0.451559495	0.15208184	2.99E-03	1.58E-02	ENSMUSG00000029998
Pcyox1l	1.409986179	0.37424652	1.65E-04	1.50E-03	ENSMUSG00000024579
Pdap1	-0.407416386	0.12425899	1.04E-03	6.83E-03	ENSMUSG00000029623
Pdcd10	-0.629574578	0.24420856	9.94E-03	4.10E-02	ENSMUSG00000027835
Pdcd2l	-0.574079777	0.17106279	7.91E-04	5.49E-03	ENSMUSG00000002635
Pdcd5	-0.562992411	0.17006242	9.31E-04	6.27E-03	ENSMUSG00000030417
Pde4b	-0.695116442	0.19214886	2.97E-04	2.44E-03	ENSMUSG00000028525
Pdgfrb	1.444042063	0.15268349	3.15E-21	6.68E-19	ENSMUSG00000024620
Pdgfrl	0.732850668	0.25729637	4.40E-03	2.16E-02	ENSMUSG00000031595
Pdha1	-0.486904595	0.13117613	2.06E-04	1.80E-03	ENSMUSG00000031299
Pdia6	0.439158082	0.10308856	2.04E-05	2.69E-04	ENSMUSG00000020571
Pdik1l	-1.063580136	0.3914618	6.59E-03	2.97E-02	ENSMUSG00000050890
Pdk1	1.075141087	0.19730078	5.06E-08	1.46E-06	ENSMUSG00000006494
Pdk2	0.617581151	0.20838051	3.04E-03	1.60E-02	ENSMUSG00000038967
Pdk4	-1.092203712	0.30672748	3.70E-04	2.92E-03	ENSMUSG00000019577
Pdlim2	0.793030019	0.11422848	3.85E-12	2.85E-10	ENSMUSG00000022090
Pdlim7	0.331698469	0.11846464	5.11E-03	2.43E-02	ENSMUSG00000021493
Pdpn	-0.38155909	0.12742125	2.75E-03	1.48E-02	ENSMUSG00000028583
Pdxdc1	-0.404291784	0.16204846	1.26E-02	4.91E-02	ENSMUSG00000022680
Pdxk	0.602016229	0.17280558	4.94E-04	3.71E-03	ENSMUSG00000032788
Pdzd8	-0.975717628	0.28518766	6.23E-04	4.49E-03	ENSMUSG00000074746
Pear1	0.80881652	0.32197847	1.20E-02	4.74E-02	ENSMUSG00000028073
Pecam1	-0.657121792	0.21349622	2.08E-03	1.18E-02	ENSMUSG00000020717

Peli1	-0.632785692	0.244144	9.55E-03	3.97E-02	ENSMUSG00000020134
Pepd	0.518040325	0.2001582	9.65E-03	4.01E-02	ENSMUSG00000063931
Pet100	1.025373998	0.12537776	2.88E-16	3.61E-14	ENSMUSG00000087687
Pex11g	1.518308864	0.40385973	1.70E-04	1.54E-03	ENSMUSG00000069633
Pex12	1.016181729	0.29197079	5.01E-04	3.75E-03	ENSMUSG00000018733
Pex19	0.441748157	0.13732326	1.30E-03	8.15E-03	ENSMUSG00000003464
Pex6	0.591357254	0.21103722	5.08E-03	2.42E-02	ENSMUSG00000002763
Pfdn4	-0.435689274	0.16889559	9.89E-03	4.08E-02	ENSMUSG00000052033
Pfdn6	-0.407343085	0.13812439	3.19E-03	1.67E-02	ENSMUSG00000024309
Pfkl	1.332971195	0.12682048	7.71E-26	2.99E-23	ENSMUSG00000020277
Pfkm	0.550155247	0.21915651	1.21E-02	4.76E-02	ENSMUSG00000033065
Pfn1	0.921417398	0.1658123	2.74E-08	8.58E-07	ENSMUSG00000018293
Pgd	0.376845384	0.13667882	5.83E-03	2.71E-02	ENSMUSG00000028961
Pgghg	0.867149355	0.3133147	5.65E-03	2.64E-02	ENSMUSG00000062031
Pgls	0.375438636	0.1174154	1.39E-03	8.56E-03	ENSMUSG00000031807
Pgm5	1.79852862	0.40131451	7.41E-06	1.13E-04	ENSMUSG00000041731
Phc2	0.559637215	0.14017304	6.54E-05	7.16E-04	ENSMUSG00000028796
Phc3	-0.912500856	0.33760199	6.87E-03	3.08E-02	ENSMUSG00000037652
Phf10	-0.477225546	0.13441188	3.85E-04	3.02E-03	ENSMUSG00000023883
Phf14	-0.738188018	0.27330481	6.91E-03	3.10E-02	ENSMUSG00000029629
Phf20	-0.682290016	0.23902062	4.31E-03	2.13E-02	ENSMUSG00000038116
Phf23	0.391638071	0.12295985	1.45E-03	8.88E-03	ENSMUSG00000018572
Phf3	-1.123478461	0.25395483	9.69E-06	1.41E-04	ENSMUSG00000048874
Phf6	-1.021992896	0.34349472	2.93E-03	1.56E-02	ENSMUSG00000025626
Phip	-1.054221902	0.23604558	7.96E-06	1.20E-04	ENSMUSG00000032253
Phldb1	0.407537201	0.16108468	1.14E-02	4.55E-02	ENSMUSG00000048537
Phldb2	-1.091083097	0.27646319	7.93E-05	8.34E-04	ENSMUSG00000033149
Phpt1	0.436800515	0.13720724	1.46E-03	8.89E-03	ENSMUSG00000036504
Phyhd1	2.330841253	0.81057385	4.03E-03	2.01E-02	ENSMUSG00000079484
Pias3	0.504444748	0.16771941	2.63E-03	1.43E-02	ENSMUSG00000028101
Picalm	-0.647001392	0.1430674	6.12E-06	9.60E-05	ENSMUSG00000039361
Pid1	0.900943291	0.19511794	3.89E-06	6.53E-05	ENSMUSG00000045658
Pidd1	1.918141945	0.60242506	1.45E-03	8.89E-03	ENSMUSG00000025507
Piezo1	0.775803215	0.14993627	2.29E-07	5.47E-06	ENSMUSG00000014444
Pigp	0.54300201	0.19028816	4.32E-03	2.13E-02	ENSMUSG00000022940
Pigq	0.646707051	0.16728537	1.11E-04	1.08E-03	ENSMUSG00000025728
Pigs	0.671365452	0.21959824	2.23E-03	1.25E-02	ENSMUSG00000041958
Pigyl	0.625132387	0.14696895	2.10E-05	2.75E-04	ENSMUSG00000010607
Pih1d1	0.731293383	0.16614069	1.07E-05	1.55E-04	ENSMUSG00000003423
Pik3c2a	-0.848576674	0.24931894	6.65E-04	4.73E-03	ENSMUSG00000030660
Pik3ca	-0.956962262	0.23928815	6.36E-05	7.02E-04	ENSMUSG00000027665
Pik3cb	-1.303940052	0.5023071	9.43E-03	3.93E-02	ENSMUSG00000032462
Pik3ip1	0.721292211	0.19369402	1.96E-04	1.74E-03	ENSMUSG00000034614

Pik3r2	0.372945623	0.13599524	6.10E-03	2.80E-02	ENSMUSG00000031834
Pim1	-1.291464523	0.182321	1.41E-12	1.08E-10	ENSMUSG00000024014
Pim3	-0.457005821	0.14834731	2.07E-03	1.17E-02	ENSMUSG00000035828
Pink1	0.549365495	0.1156144	2.02E-06	3.75E-05	ENSMUSG00000028756
Pirb	1.717284183	0.44754186	1.24E-04	1.19E-03	ENSMUSG00000058818
Pitpna	0.34740325	0.11365092	2.24E-03	1.25E-02	ENSMUSG00000017781
Pitpnb	-0.458808892	0.16476724	5.36E-03	2.53E-02	ENSMUSG00000050017
Pja2	-0.563462501	0.18550294	2.39E-03	1.32E-02	ENSMUSG00000024083
Pkd1	0.664922997	0.15062376	1.01E-05	1.46E-04	ENSMUSG00000032855
Pkdcc	0.908577592	0.15989305	1.33E-08	4.57E-07	ENSMUSG00000024247
Pkm	0.785526939	0.11965241	5.20E-11	3.24E-09	ENSMUSG00000032294
Pkn2	-0.896915575	0.26570526	7.37E-04	5.18E-03	ENSMUSG00000004591
Pla2g15	0.541464669	0.14776119	2.48E-04	2.10E-03	ENSMUSG00000031903
Pla2g4a	-1.553815333	0.29623862	1.56E-07	3.91E-06	ENSMUSG00000056220
Pla2g5	2.35853449	0.51795598	5.28E-06	8.44E-05	ENSMUSG00000041193
Pla2g7	0.85495334	0.22190951	1.17E-04	1.14E-03	ENSMUSG00000023913
Plaa	-1.001417433	0.17835332	1.97E-08	6.44E-07	ENSMUSG00000028577
Plagl1	-0.515605733	0.13629122	1.55E-04	1.43E-03	ENSMUSG00000019817
Plau	1.36752205	0.25938381	1.35E-07	3.44E-06	ENSMUSG00000021822
Plaur	0.439669461	0.15745082	5.23E-03	2.48E-02	ENSMUSG00000046223
Plbd2	0.710632273	0.13482762	1.36E-07	3.46E-06	ENSMUSG00000029598
Plcb3	0.741643112	0.29759547	1.27E-02	4.94E-02	ENSMUSG00000024960
Plcd1	0.879312002	0.1477005	2.63E-09	1.05E-07	ENSMUSG00000010660
Plcxd2	-3.887566417	1.00060642	1.02E-04	1.02E-03	ENSMUSG00000087141
Pld3	0.519674384	0.13922519	1.90E-04	1.69E-03	ENSMUSG00000003363
Plec	0.813364005	0.15259279	9.81E-08	2.59E-06	ENSMUSG00000022565
Plekha1	-0.592708122	0.21568597	6.00E-03	2.76E-02	ENSMUSG00000040268
Plekhb2	-0.385280717	0.13366546	3.95E-03	1.98E-02	ENSMUSG00000026123
Plekhg4	2.571990385	0.39206432	5.38E-11	3.34E-09	ENSMUSG00000014782
Plekhg5	1.040117872	0.1722816	1.57E-09	6.63E-08	ENSMUSG00000039713
Plekhh2	1.35476971	0.3786657	3.47E-04	2.77E-03	ENSMUSG00000040852
Plekhh3	0.806508913	0.21497418	1.76E-04	1.58E-03	ENSMUSG00000035172
Plin1	1.922120176	0.52284197	2.37E-04	2.03E-03	ENSMUSG00000030546
Plin2	-0.93147697	0.13017353	8.33E-13	6.81E-11	ENSMUSG00000028494
Plin4	1.269388493	0.36408599	4.89E-04	3.68E-03	ENSMUSG00000002831
Plk1	1.358443	0.3103707	1.20E-05	1.70E-04	ENSMUSG00000030867
Plk3	-0.647050561	0.24631314	8.62E-03	3.66E-02	ENSMUSG00000028680
Plod1	0.999787586	0.10178041	8.96E-23	2.51E-20	ENSMUSG00000019055
Plpp1	0.820205046	0.11459099	8.21E-13	6.75E-11	ENSMUSG00000021759
Plpp6	1.05911356	0.27961645	1.52E-04	1.41E-03	ENSMUSG00000040105
Plpp7	1.173557106	0.29660899	7.60E-05	8.05E-04	ENSMUSG00000051373
Plrg1	-0.366606753	0.14529055	1.16E-02	4.62E-02	ENSMUSG00000027998
Pls3	-0.678978508	0.21781056	1.83E-03	1.07E-02	ENSMUSG00000016382

Pltp	1.138565408	0.15348058	1.19E-13	1.09E-11	ENSMUSG00000017754
Plxna2	1.044415627	0.4141237	1.17E-02	4.63E-02	ENSMUSG00000026640
Plxnb2	0.667021771	0.1296514	2.68E-07	6.25E-06	ENSMUSG00000036606
Plxnd1	1.034306348	0.27720132	1.91E-04	1.69E-03	ENSMUSG00000030123
Pmepa1	0.810294832	0.12891026	3.26E-10	1.66E-08	ENSMUSG00000038400
Pmp22	0.635846764	0.11410334	2.51E-08	7.96E-07	ENSMUSG00000018217
Pnkp	0.432401429	0.17105392	1.15E-02	4.57E-02	ENSMUSG00000002963
Pnn	-1.032871548	0.24884839	3.32E-05	4.06E-04	ENSMUSG00000020994
Pno1	-0.598895646	0.15290034	8.97E-05	9.19E-04	ENSMUSG00000020116
Pnpla2	-0.499355966	0.13383904	1.91E-04	1.70E-03	ENSMUSG00000025509
Pnpla8	-0.907426782	0.26290879	5.58E-04	4.10E-03	ENSMUSG00000036257
Podnl1	1.896748943	0.17883562	2.79E-26	1.15E-23	ENSMUSG00000012889
Podxl2	1.184668149	0.43094419	5.98E-03	2.75E-02	ENSMUSG00000033152
Pofut1	0.707036372	0.24777389	4.32E-03	2.13E-02	ENSMUSG00000046020
Pogk	-0.938482575	0.25969237	3.02E-04	2.47E-03	ENSMUSG00000040596
Poglut1	0.554181334	0.17629834	1.67E-03	9.97E-03	ENSMUSG00000034064
Polb	-0.478716922	0.17023416	4.92E-03	2.37E-02	ENSMUSG00000031536
Pole3	-0.430874922	0.14815373	3.63E-03	1.85E-02	ENSMUSG00000028394
Polm	0.963357742	0.36111279	7.64E-03	3.34E-02	ENSMUSG00000020474
Polr1c	-0.537744191	0.15486913	5.16E-04	3.85E-03	ENSMUSG00000067148
Polr1d	-0.35344716	0.11866963	2.90E-03	1.54E-02	ENSMUSG00000029642
Polr2a	0.639406081	0.18230497	4.53E-04	3.46E-03	ENSMUSG00000005198
Polr2c	-0.371465363	0.1277036	3.63E-03	1.85E-02	ENSMUSG00000031783
Pomt1	0.864967841	0.28682939	2.56E-03	1.40E-02	ENSMUSG00000039254
Postn	-0.569710421	0.17727739	1.31E-03	8.21E-03	ENSMUSG00000027750
Ppat	-0.858130914	0.30401728	4.76E-03	2.30E-02	ENSMUSG00000029246
Ppid	-0.703123627	0.18351909	1.27E-04	1.22E-03	ENSMUSG00000027804
Ppil4	-0.632728875	0.23093602	6.15E-03	2.81E-02	ENSMUSG00000015757
Ppm1a	-0.386252514	0.11515597	7.96E-04	5.52E-03	ENSMUSG00000021096
Ppm1j	-1.253302788	0.3370838	2.01E-04	1.77E-03	ENSMUSG00000002228
Ppme1	0.383602139	0.14217653	6.97E-03	3.12E-02	ENSMUSG00000030718
Ppp1ca	0.51257695	0.12030279	2.04E-05	2.69E-04	ENSMUSG00000040385
Ppp1r10	0.671279013	0.1891342	3.86E-04	3.03E-03	ENSMUSG00000039220
Ppp1r12a	-0.884927327	0.21919822	5.41E-05	6.13E-04	ENSMUSG00000019907
Ppp1r15a	-0.683334108	0.16019545	1.99E-05	2.64E-04	ENSMUSG00000040435
Ppp1r1a	1.45381736	0.46962163	1.96E-03	1.13E-02	ENSMUSG00000022490
Ppp1r1b	1.812779692	0.16176259	3.79E-29	1.92E-26	ENSMUSG00000061718
Ppp1r3c	0.815241294	0.2526008	1.25E-03	7.93E-03	ENSMUSG00000067279
Ppp1r9b	0.602875742	0.15097885	6.52E-05	7.15E-04	ENSMUSG00000038976
Ppp2ca	-0.393476609	0.11532769	6.45E-04	4.63E-03	ENSMUSG00000020349
Ppp2r2a	-0.699131808	0.15487736	6.36E-06	9.92E-05	ENSMUSG00000022052
Ppp2r2d	-0.492452184	0.17370378	4.58E-03	2.23E-02	ENSMUSG00000041769
Ppp3ca	-0.626189418	0.18237527	5.96E-04	4.32E-03	ENSMUSG00000028161

Ppp3cb	-0.513706041	0.17028364	2.55E-03	1.40E-02	ENSMUSG00000021816
Ppp4r2	-0.671651	0.20817462	1.25E-03	7.95E-03	ENSMUSG00000052144
Ppp4r3a	-0.691516792	0.26277437	8.50E-03	3.62E-02	ENSMUSG00000041846
Ppp4r3b	-0.948992681	0.27581059	5.80E-04	4.24E-03	ENSMUSG00000020463
Ppp6c	-0.457163641	0.14884965	2.13E-03	1.20E-02	ENSMUSG00000026753
Ppt1	0.73497727	0.17843081	3.80E-05	4.53E-04	ENSMUSG00000028657
Ppt2	0.770507389	0.19743222	9.51E-05	9.66E-04	ENSMUSG00000015474
Pptc7	-0.757326202	0.1830076	3.50E-05	4.22E-04	ENSMUSG00000038582
Pqlc2	1.139687224	0.24753692	4.14E-06	6.89E-05	ENSMUSG00000028744
Prdm4	-0.906554332	0.3060452	3.05E-03	1.61E-02	ENSMUSG00000035529
Prdx1	-0.360976193	0.10022636	3.16E-04	2.57E-03	ENSMUSG00000028691
Prdx4	0.304427321	0.12008777	1.12E-02	4.50E-02	ENSMUSG00000025289
Prdx6	-0.780049042	0.10376611	5.59E-14	5.25E-12	ENSMUSG00000026701
Prelid1	0.689876203	0.14476708	1.88E-06	3.53E-05	ENSMUSG00000021486
Prelid3b	-0.461545996	0.1297247	3.74E-04	2.95E-03	ENSMUSG00000016257
Prelp	1.469671937	0.16871234	3.01E-18	4.95E-16	ENSMUSG00000041577
Prex1	1.094431815	0.2357017	3.43E-06	5.86E-05	ENSMUSG00000039621
Prkaa1	-0.479818233	0.18241355	8.53E-03	3.63E-02	ENSMUSG00000050697
Prkacb	-0.46719958	0.16965457	5.89E-03	2.72E-02	ENSMUSG00000005034
Prkar2b	-1.097466076	0.24847566	1.00E-05	1.45E-04	ENSMUSG00000002997
Prkd1	-0.732080599	0.25669721	4.35E-03	2.14E-02	ENSMUSG00000002688
Prkg2	-1.084198617	0.2345255	3.78E-06	6.41E-05	ENSMUSG00000029334
Prkx	-0.940481041	0.30007871	1.72E-03	1.02E-02	ENSMUSG00000035725
Procr	-0.976139361	0.13607155	7.30E-13	6.08E-11	ENSMUSG00000027611
Prpf18	-0.855837855	0.32150324	7.77E-03	3.38E-02	ENSMUSG00000039449
Prpf3	-0.805531718	0.23713077	6.81E-04	4.83E-03	ENSMUSG00000015748
Prpf38b	-1.332843947	0.22873815	5.65E-09	2.09E-07	ENSMUSG00000027881
Prpf39	-1.246589756	0.2898295	1.70E-05	2.30E-04	ENSMUSG00000035597
Prpf40a	-1.290824425	0.21871589	3.59E-09	1.39E-07	ENSMUSG00000061136
Prpf4b	-0.921377234	0.25318742	2.74E-04	2.28E-03	ENSMUSG00000021413
Prr13	0.363737644	0.13520523	7.14E-03	3.17E-02	ENSMUSG00000023048
Prr3	1.053786485	0.29102548	2.94E-04	2.41E-03	ENSMUSG00000038500
Prr5	0.868354606	0.19634561	9.75E-06	1.41E-04	ENSMUSG00000036106
Prr7	1.03621852	0.35262016	3.30E-03	1.71E-02	ENSMUSG00000034686
Prrc1	-0.474220191	0.13511835	4.49E-04	3.44E-03	ENSMUSG00000024594
Prrc2a	0.398614675	0.14987591	7.82E-03	3.40E-02	ENSMUSG00000024393
Prrc2c	-1.226193682	0.43416706	4.74E-03	2.29E-02	ENSMUSG00000040225
Prrg3	1.106165781	0.42027648	8.49E-03	3.62E-02	ENSMUSG00000033361
Prrt1	1.891456193	0.45806251	3.64E-05	4.36E-04	ENSMUSG00000015476
Prrx2	1.364135356	0.14194091	7.21E-22	1.67E-19	ENSMUSG00000039476
Psat1	-0.525265977	0.13166909	6.63E-05	7.22E-04	ENSMUSG00000024640
Psd4	2.59285526	0.4277976	1.35E-09	5.86E-08	ENSMUSG00000026979
Psip1	-0.996875386	0.3246606	2.14E-03	1.21E-02	ENSMUSG00000028484

Pskh1	0.530138757	0.16979037	1.79E-03	1.05E-02	ENSMUSG00000048310
Psma1	-0.456546602	0.1328925	5.92E-04	4.30E-03	ENSMUSG00000030751
Psma2	-0.387113962	0.10965597	4.15E-04	3.22E-03	ENSMUSG00000015671
Psma4	-0.370133799	0.13859822	7.57E-03	3.32E-02	ENSMUSG00000032301
Psmb1	-0.333412109	0.10345309	1.27E-03	8.02E-03	ENSMUSG00000014769
Psmb6	-0.314127671	0.10039441	1.75E-03	1.03E-02	ENSMUSG00000018286
Psmb9	1.707791575	0.62013132	5.89E-03	2.72E-02	ENSMUSG00000096687
Psmc2	-0.588519802	0.12121513	1.20E-06	2.37E-05	ENSMUSG00000028932
Psmc5	0.325617131	0.13052362	1.26E-02	4.91E-02	ENSMUSG00000020708
Psmc6	-1.17283202	0.17890083	5.54E-11	3.41E-09	ENSMUSG00000021832
Psmd1	-0.641187876	0.18256139	4.44E-04	3.41E-03	ENSMUSG00000026229
Psmd11	-0.350237931	0.13003773	7.07E-03	3.15E-02	ENSMUSG00000017428
Psmd12	-0.459199387	0.16674514	5.89E-03	2.72E-02	ENSMUSG00000020720
Psmd14	-0.807749679	0.13442678	1.87E-09	7.81E-08	ENSMUSG00000026914
Psmd4	-0.331482525	0.12105351	6.18E-03	2.82E-02	ENSMUSG00000005625
Psmd8	-0.501954059	0.11842461	2.25E-05	2.90E-04	ENSMUSG00000030591
Psme4	-0.67425058	0.21588133	1.79E-03	1.05E-02	ENSMUSG00000040850
Psrc1	-0.564088453	0.20126865	5.07E-03	2.42E-02	ENSMUSG00000068744
Ptbp3	-0.834541812	0.1690117	7.90E-07	1.65E-05	ENSMUSG00000028382
Ptch1	1.6043346	0.19071452	4.02E-17	5.57E-15	ENSMUSG00000021466
Ptch2	1.74526278	0.52173731	8.23E-04	5.67E-03	ENSMUSG00000028681
Ptges3	-0.493935242	0.14558463	6.92E-04	4.89E-03	ENSMUSG00000071072
Ptgis	1.001056967	0.19128677	1.67E-07	4.14E-06	ENSMUSG00000017969
Ptgs1	0.587934336	0.20969767	5.05E-03	2.41E-02	ENSMUSG00000047250
Ptgs2	-2.320295078	0.19682447	4.47E-32	2.94E-29	ENSMUSG00000032487
Ptgs2os	1.08964309	0.27993962	9.92E-05	9.97E-04	ENSMUSG00000096981
Pth1r	2.793351112	0.11894713	5.95E-122	7.83E-118	ENSMUSG00000032492
Ptk2	-0.662385606	0.2160365	2.17E-03	1.22E-02	ENSMUSG00000022607
Ptk2b	1.07926004	0.39397344	6.15E-03	2.81E-02	ENSMUSG00000059456
Ptk7	0.717254081	0.14420041	6.56E-07	1.39E-05	ENSMUSG00000023972
Ptms	0.729395744	0.11938784	1.00E-09	4.49E-08	ENSMUSG00000030122
Ptov1	0.595652749	0.14019246	2.15E-05	2.79E-04	ENSMUSG00000038502
Ptp4a1	-0.658848698	0.21179986	1.87E-03	1.09E-02	ENSMUSG00000026064
Ptp4a2	-0.528067358	0.12516748	2.46E-05	3.12E-04	ENSMUSG00000028788
Ptpa	0.348815372	0.13410855	9.30E-03	3.88E-02	ENSMUSG00000039515
Ptpn12	-0.679913683	0.19460335	4.76E-04	3.60E-03	ENSMUSG00000028771
Ptpn2	-0.791881439	0.2803903	4.74E-03	2.29E-02	ENSMUSG00000024539
Ptpre	-1.171336051	0.39112984	2.75E-03	1.48E-02	ENSMUSG00000041836
Ptprf	0.602088157	0.17153865	4.48E-04	3.44E-03	ENSMUSG00000033295
Ptprv	0.625888966	0.16738999	1.85E-04	1.65E-03	ENSMUSG00000097971
Ptprz1	3.147168795	0.85421745	2.29E-04	1.97E-03	ENSMUSG00000068748
Pts	0.773008412	0.25029459	2.01E-03	1.15E-02	ENSMUSG00000032067
Pum1	-0.405293615	0.14573049	5.42E-03	2.55E-02	ENSMUSG00000028580

Pum2	-0.643172333	0.20064914	1.35E-03	8.39E-03	ENSMUSG00000020594
Pum3	-0.889289845	0.28086104	1.54E-03	9.33E-03	ENSMUSG00000041360
Purb	-0.535422166	0.19564509	6.21E-03	2.84E-02	ENSMUSG00000093904
Pvr	-0.455674231	0.16681787	6.30E-03	2.87E-02	ENSMUSG00000040511
Pvt1	-1.428540632	0.50852547	4.97E-03	2.39E-02	ENSMUSG00000097028
Pwwp2b	1.070755316	0.40274881	7.85E-03	3.41E-02	ENSMUSG00000060260
Pwwp3a	0.766374565	0.23149288	9.31E-04	6.27E-03	ENSMUSG00000020156
Pxmp4	0.612351262	0.16851289	2.79E-04	2.32E-03	ENSMUSG00000000876
Pygl	0.568989907	0.16010499	3.80E-04	2.99E-03	ENSMUSG00000021069
Pyroxd2	1.591768757	0.57849664	5.93E-03	2.74E-02	ENSMUSG00000060224
Pyurf	0.670097362	0.21106281	1.50E-03	9.12E-03	ENSMUSG00000043162
Qpctl	0.699671243	0.22509998	1.88E-03	1.09E-02	ENSMUSG00000030407
Qser1	-0.923725623	0.18168946	3.69E-07	8.36E-06	ENSMUSG00000074994
R3hdm4	0.324249365	0.12373716	8.78E-03	3.72E-02	ENSMUSG00000035781
Rab10	-0.742112717	0.12561889	3.47E-09	1.35E-07	ENSMUSG00000020671
Rab11fip4	1.471590103	0.37432703	8.45E-05	8.75E-04	ENSMUSG00000017639
Rab14	-0.610358783	0.14652248	3.10E-05	3.82E-04	ENSMUSG00000026878
Rab18	-0.555814168	0.12279689	6.00E-06	9.45E-05	ENSMUSG00000073639
Rab1a	-0.67648607	0.13838584	1.02E-06	2.06E-05	ENSMUSG00000020149
Rab1b	0.604624221	0.1284222	2.50E-06	4.48E-05	ENSMUSG00000024870
Rab24	0.389530071	0.12146901	1.34E-03	8.35E-03	ENSMUSG00000034789
Rab27b	-1.550997663	0.60045555	9.79E-03	4.05E-02	ENSMUSG00000024511
Rab2a	-0.353160637	0.1095019	1.26E-03	7.98E-03	ENSMUSG00000047187
Rab33b	-0.563227199	0.18608795	2.47E-03	1.36E-02	ENSMUSG00000027739
Rab34	0.498729842	0.1176287	2.24E-05	2.89E-04	ENSMUSG00000002059
Rab38	1.913882976	0.47034432	4.72E-05	5.45E-04	ENSMUSG00000030559
Rab3il1	0.633373857	0.23650659	7.41E-03	3.27E-02	ENSMUSG00000024663
Rab3ip	-1.170390862	0.28643486	4.39E-05	5.13E-04	ENSMUSG00000064181
Rab6a	-0.403777917	0.13787688	3.41E-03	1.76E-02	ENSMUSG00000030704
Rab7b	-0.677498072	0.17442217	1.03E-04	1.02E-03	ENSMUSG00000052688
Rab9	-0.814694215	0.15730262	2.23E-07	5.35E-06	ENSMUSG00000079316
Rabep1	-0.703172316	0.2658471	8.17E-03	3.51E-02	ENSMUSG00000020817
Rabggtb	-1.016337046	0.12772066	1.76E-15	2.03E-13	ENSMUSG00000038975
Rack1	-0.5161137	0.11027236	2.86E-06	5.03E-05	ENSMUSG00000020372
Rad1	-0.434325851	0.16933975	1.03E-02	4.22E-02	ENSMUSG00000022248
Rad17	-0.868564322	0.28190868	2.06E-03	1.17E-02	ENSMUSG00000021635
Rad18	-1.051488766	0.38441944	6.23E-03	2.85E-02	ENSMUSG00000030254
Rad21	-0.686420426	0.15410758	8.42E-06	1.25E-04	ENSMUSG00000022314
Rad23b	-0.354736234	0.10588432	8.07E-04	5.59E-03	ENSMUSG00000028426
Rad50	-1.007023334	0.29488637	6.38E-04	4.58E-03	ENSMUSG00000020380
Rala	-0.610127522	0.11629959	1.55E-07	3.89E-06	ENSMUSG00000008859
Ralbp1	-0.361512999	0.12623878	4.19E-03	2.07E-02	ENSMUSG00000024096
Ralgapb	-0.974329189	0.24288753	6.03E-05	6.71E-04	ENSMUSG00000027652

Ramp1	1.436166367	0.21982602	6.44E-11	3.89E-09	ENSMUSG00000034353
Ramp2	1.96638718	0.16913326	3.03E-31	1.73E-28	ENSMUSG00000001240
Ranbp1	-0.339504187	0.1335883	1.10E-02	4.44E-02	ENSMUSG00000005732
Ranbp2	-0.7814936	0.3073114	1.10E-02	4.43E-02	ENSMUSG00000003226
Ranbp9	-0.690190833	0.22377943	2.04E-03	1.16E-02	ENSMUSG00000038546
Rap1b	-0.56832975	0.12456855	5.06E-06	8.17E-05	ENSMUSG00000052681
Rap1gap	1.943161247	0.61223853	1.50E-03	9.15E-03	ENSMUSG00000041351
Rapgef6	-1.637233886	0.35505845	4.00E-06	6.69E-05	ENSMUSG00000037533
Rara	0.771817487	0.23329699	9.39E-04	6.31E-03	ENSMUSG00000037992
Rarres2	0.993614393	0.30143136	9.80E-04	6.51E-03	ENSMUSG00000009281
Rars	-0.954429724	0.19552339	1.05E-06	2.12E-05	ENSMUSG00000018848
Rasa1	-0.918734159	0.24913141	2.26E-04	1.95E-03	ENSMUSG00000021549
Rasa2	-1.069908368	0.30551757	4.62E-04	3.52E-03	ENSMUSG00000032413
Rasd1	1.52137844	0.52205718	3.57E-03	1.82E-02	ENSMUSG00000049892
Rasd2	-1.506320319	0.60452383	1.27E-02	4.94E-02	ENSMUSG00000034472
Rasgef1b	-2.974495726	0.98874661	2.63E-03	1.43E-02	ENSMUSG00000089809
Rasgrp2	1.510473137	0.25362046	2.59E-09	1.04E-07	ENSMUSG00000032946
Rasl11b	1.084990085	0.21484376	4.41E-07	9.80E-06	ENSMUSG00000049907
Rassf1	-0.50945346	0.16622277	2.18E-03	1.23E-02	ENSMUSG00000010067
Rassf2	1.477188736	0.24437372	1.50E-09	6.39E-08	ENSMUSG00000027339
Rb1cc1	-0.847642418	0.32937	1.01E-02	4.14E-02	ENSMUSG00000025907
Rbbp8	-1.140902951	0.42524291	7.30E-03	3.23E-02	ENSMUSG00000041238
Rbm25	-1.09494785	0.37327395	3.35E-03	1.73E-02	ENSMUSG00000010608
Rbm28	-0.885274361	0.20553183	1.65E-05	2.25E-04	ENSMUSG00000029701
Rbm33	-0.924393568	0.26360621	4.54E-04	3.46E-03	ENSMUSG00000048271
Rbm34	-0.939973683	0.3357121	5.11E-03	2.43E-02	ENSMUSG00000033931
Rbm39	-0.737981164	0.16074903	4.41E-06	7.28E-05	ENSMUSG00000027620
Rbm45	1.063780678	0.27426516	1.05E-04	1.04E-03	ENSMUSG00000042369
Rbm6	-0.911621724	0.30856078	3.13E-03	1.64E-02	ENSMUSG00000032582
Rbm7	-0.77533578	0.17483811	9.22E-06	1.35E-04	ENSMUSG00000042396
Rbm8a	-0.379433594	0.11801515	1.30E-03	8.19E-03	ENSMUSG00000038374
Rbms1	-0.9311876	0.1773295	1.51E-07	3.80E-06	ENSMUSG00000026970
Rbms2	0.387603075	0.15038446	9.95E-03	4.10E-02	ENSMUSG00000040043
Rbms3	-0.53217279	0.20568047	9.67E-03	4.02E-02	ENSMUSG00000039607
Rbpj	-1.462084153	0.18703659	5.41E-15	5.74E-13	ENSMUSG00000039191
Rcan2	1.714428186	0.28089261	1.04E-09	4.59E-08	ENSMUSG00000039601
Rcc1l	-0.51886031	0.18874692	5.98E-03	2.75E-02	ENSMUSG00000061979
Rcn3	0.282274959	0.10059549	5.02E-03	2.40E-02	ENSMUSG00000019539
Rcor2	0.613791339	0.20517712	2.78E-03	1.49E-02	ENSMUSG00000024968
Rcsd1	1.066024985	0.35161889	2.43E-03	1.34E-02	ENSMUSG00000040723
Rdh13	-0.690557899	0.25375733	6.50E-03	2.94E-02	ENSMUSG00000008435
Rdx	-1.007982257	0.19832196	3.72E-07	8.41E-06	ENSMUSG00000032050
Reep3	-0.441990817	0.15649021	4.74E-03	2.29E-02	ENSMUSG00000019873

Rell1	-0.769314566	0.17641104	1.30E-05	1.82E-04	ENSMUSG00000047881
Rer1	-0.383255354	0.13008522	3.22E-03	1.68E-02	ENSMUSG00000029048
Retn	1.067395614	0.42129333	1.13E-02	4.51E-02	ENSMUSG00000012705
Retreg1	-0.896397909	0.29755482	2.59E-03	1.41E-02	ENSMUSG00000022270
Retsat	0.877389222	0.22545554	9.96E-05	1.00E-03	ENSMUSG00000056666
Rev3l	-0.923609961	0.33507584	5.84E-03	2.71E-02	ENSMUSG00000019841
Rex1bd	0.601180056	0.1721444	4.79E-04	3.62E-03	ENSMUSG00000058833
Rexo2	0.554772566	0.09874429	1.93E-08	6.35E-07	ENSMUSG00000032026
Rfc1	-0.628415324	0.24418749	1.01E-02	4.14E-02	ENSMUSG00000029191
Rfc5	0.897580597	0.25530115	4.38E-04	3.37E-03	ENSMUSG00000029363
Rffl	-1.404767737	0.39912444	4.32E-04	3.33E-03	ENSMUSG00000020696
Rflna	1.244433117	0.35689756	4.89E-04	3.68E-03	ENSMUSG00000037962
Rflnb	-0.431689649	0.14879151	3.72E-03	1.88E-02	ENSMUSG00000020846
Rfng	0.766123812	0.26466422	3.80E-03	1.91E-02	ENSMUSG00000025158
Rftn1	0.616988832	0.24254029	1.10E-02	4.42E-02	ENSMUSG00000039316
Rgs1	-3.180803336	0.47568806	2.28E-11	1.50E-09	ENSMUSG00000026358
Rgs10	-0.4492275	0.15830513	4.54E-03	2.21E-02	ENSMUSG00000030844
Rgs16	-1.058882946	0.25546161	3.40E-05	4.13E-04	ENSMUSG00000026475
Rgs17	-1.080297098	0.19290186	2.14E-08	6.89E-07	ENSMUSG00000019775
Rgs2	0.873144431	0.1570209	2.69E-08	8.44E-07	ENSMUSG00000026360
Rgs4	-0.745837878	0.25678925	3.68E-03	1.87E-02	ENSMUSG00000038530
Rgs5	-0.592316917	0.2047123	3.81E-03	1.92E-02	ENSMUSG00000026678
Rhbdd1	-0.76262743	0.16012384	1.91E-06	3.57E-05	ENSMUSG00000026142
Rhbdd2	1.913035498	0.34358171	2.58E-08	8.12E-07	ENSMUSG00000039917
Rheb	-0.705244427	0.15406196	4.70E-06	7.69E-05	ENSMUSG00000028945
Rhob	0.684962645	0.12630525	5.86E-08	1.65E-06	ENSMUSG00000054364
Rhobtb3	-0.634330156	0.23994275	8.20E-03	3.52E-02	ENSMUSG00000021589
Rhoq	-0.67839276	0.16358996	3.37E-05	4.10E-04	ENSMUSG00000024143
Rhot1	-0.622549548	0.19873198	1.73E-03	1.02E-02	ENSMUSG00000017686
Rictor	-1.087862825	0.27800575	9.11E-05	9.32E-04	ENSMUSG00000050310
Rif1	-0.955225725	0.37654331	1.12E-02	4.49E-02	ENSMUSG00000036202
Rilpl2	0.437161595	0.14131223	1.98E-03	1.13E-02	ENSMUSG00000029401
Riok2	0.522062367	0.16777597	1.86E-03	1.08E-02	ENSMUSG00000116138
Riok3	-0.899370484	0.16737541	7.73E-08	2.12E-06	ENSMUSG00000024404
Rit1	0.332464068	0.11210991	3.02E-03	1.59E-02	ENSMUSG00000028057
Rlf	-1.016805935	0.2493632	4.55E-05	5.30E-04	ENSMUSG00000049878
Rlim	-0.943472582	0.1941702	1.18E-06	2.33E-05	ENSMUSG00000056537
Rmnd5a	-0.615119895	0.24249191	1.12E-02	4.49E-02	ENSMUSG00000002222
Rnase4	0.721037252	0.15280023	2.37E-06	4.29E-05	ENSMUSG00000021876
Rnaseh2a	-0.569905019	0.17920258	1.47E-03	8.97E-03	ENSMUSG00000052926
Rnd1	-2.290912808	0.49850769	4.32E-06	7.14E-05	ENSMUSG00000054855
Rnd2	0.710115652	0.12524192	1.43E-08	4.86E-07	ENSMUSG00000001313
Rnf11	-0.746387923	0.12417143	1.84E-09	7.76E-08	ENSMUSG00000028557

Rnf123	0.548326282	0.18911148	3.74E-03	1.89E-02	ENSMUSG00000041528
Rnf126	0.464703222	0.1580197	3.27E-03	1.70E-02	ENSMUSG00000035890
Rnf138	-1.133138443	0.28154179	5.70E-05	6.38E-04	ENSMUSG00000024317
Rnf141	-0.622690994	0.22344504	5.32E-03	2.52E-02	ENSMUSG00000030788
Rnf181	0.335909432	0.1114485	2.58E-03	1.41E-02	ENSMUSG00000055850
Rnf2	-0.484743284	0.17002457	4.36E-03	2.14E-02	ENSMUSG00000026484
Rnf20	-0.732958204	0.18079734	5.03E-05	5.75E-04	ENSMUSG00000028309
Rnf219	-0.80875264	0.32387474	1.25E-02	4.89E-02	ENSMUSG00000022120
Rnf4	-0.438960781	0.16462761	7.67E-03	3.35E-02	ENSMUSG00000029110
Rnf6	-1.029156956	0.35915152	4.16E-03	2.06E-02	ENSMUSG00000029634
Rnmt	-1.198615517	0.18237682	4.96E-11	3.14E-09	ENSMUSG00000009535
Ro60	-1.17442696	0.26163906	7.16E-06	1.10E-04	ENSMUSG00000018199
Rock1	-1.211669108	0.42760438	4.60E-03	2.24E-02	ENSMUSG00000024290
Rom1	1.391894212	0.27890101	6.02E-07	1.29E-05	ENSMUSG00000071648
Ropn1l	1.530225491	0.576583	7.96E-03	3.45E-02	ENSMUSG00000022236
Rora	-1.219032466	0.27882352	1.23E-05	1.74E-04	ENSMUSG00000032238
Rpain	-0.554100566	0.17168054	1.25E-03	7.93E-03	ENSMUSG00000018449
Rpf2	-1.020861841	0.24773704	3.78E-05	4.50E-04	ENSMUSG00000038510
Rpl11	-0.542388616	0.12584609	1.63E-05	2.22E-04	ENSMUSG00000059291
Rpl12	-0.643524046	0.1517774	2.24E-05	2.89E-04	ENSMUSG00000038900
Rpl13	-0.51664394	0.09949586	2.07E-07	5.01E-06	ENSMUSG00000000740
Rpl13a	-0.47532047	0.11933181	6.80E-05	7.38E-04	ENSMUSG00000074129
Rpl13a-ps1	-0.671416496	0.26949394	1.27E-02	4.95E-02	ENSMUSG00000062083
Rpl14	-0.570968764	0.09774106	5.17E-09	1.93E-07	ENSMUSG00000025794
Rpl15-ps5	-1.432753012	0.14654761	1.42E-22	3.73E-20	ENSMUSG00000108414
Rpl17	-0.813339436	0.14339538	1.41E-08	4.81E-07	ENSMUSG00000062328
Rpl18	-0.48810616	0.11114822	1.13E-05	1.61E-04	ENSMUSG00000059070
Rpl18-ps1	-0.460003385	0.16743353	6.01E-03	2.76E-02	ENSMUSG00000081382
Rpl18a-ps1	-1.61388468	0.63383998	1.09E-02	4.40E-02	ENSMUSG00000059033
Rpl19	-0.483474573	0.16700473	3.79E-03	1.91E-02	ENSMUSG00000017404
Rpl22	-0.627434838	0.12763096	8.83E-07	1.82E-05	ENSMUSG00000028936
Rpl22l1	-0.463611305	0.13552454	6.24E-04	4.50E-03	ENSMUSG00000039221
Rpl23	-0.708571704	0.1561893	5.72E-06	9.06E-05	ENSMUSG00000071415
Rpl23a	-0.578227329	0.21934789	8.39E-03	3.58E-02	ENSMUSG00000058546
Rpl26	-0.73027447	0.11786653	5.80E-10	2.81E-08	ENSMUSG00000060938
Rpl26-ps2	-1.512889752	0.53522301	4.70E-03	2.28E-02	ENSMUSG00000066489
Rpl28-ps1	-1.305158088	0.50969476	1.04E-02	4.26E-02	ENSMUSG00000058603
Rpl3	-0.446749395	0.17278628	9.72E-03	4.03E-02	ENSMUSG00000060036
Rpl31	-0.573056685	0.10479136	4.54E-08	1.33E-06	ENSMUSG00000073702
Rpl31-ps8	-0.73149624	0.14358175	3.49E-07	7.96E-06	ENSMUSG00000067870
Rpl32	-0.595675999	0.10788993	3.37E-08	1.03E-06	ENSMUSG00000057841
Rpl34	-0.47619969	0.11899446	6.28E-05	6.95E-04	ENSMUSG00000062006
Rpl35a	-0.688168409	0.19538877	4.28E-04	3.31E-03	ENSMUSG00000060636

Rpl36-ps12	-0.730203473	0.25294389	3.89E-03	1.96E-02	ENSMUSG00000091845
Rpl36a	-1.047494779	0.39531973	8.06E-03	3.48E-02	ENSMUSG00000079435
Rpl37	-0.568505606	0.12178184	3.04E-06	5.29E-05	ENSMUSG00000041841
Rpl37a	-0.423559139	0.12253655	5.47E-04	4.03E-03	ENSMUSG00000046330
Rpl37rt	-0.612176564	0.19802535	1.99E-03	1.14E-02	ENSMUSG00000072692
Rpl38	-0.596425333	0.17437652	6.25E-04	4.50E-03	ENSMUSG00000057322
Rpl39	-0.557788514	0.13833788	5.53E-05	6.24E-04	ENSMUSG00000079641
Rpl4	-0.726084399	0.11706589	5.56E-10	2.70E-08	ENSMUSG00000032399
Rpl6	-0.459594943	0.14568171	1.61E-03	9.63E-03	ENSMUSG00000029614
Rpl7-ps7	1.401255984	0.44021625	1.46E-03	8.90E-03	ENSMUSG00000106447
Rpl7a	-0.819301966	0.3146413	9.22E-03	3.86E-02	ENSMUSG00000062647
Rpl8	-0.41112138	0.10187992	5.45E-05	6.16E-04	ENSMUSG00000003970
Rplp1	-0.363965746	0.09359496	1.01E-04	1.01E-03	ENSMUSG00000007892
Rplp2	-0.522989885	0.17800726	3.30E-03	1.71E-02	ENSMUSG00000025508
Rpn2	0.297673629	0.10628733	5.10E-03	2.43E-02	ENSMUSG00000027642
Rpp21	0.374942365	0.14995087	1.24E-02	4.86E-02	ENSMUSG00000024446
Rpp30	-0.709011565	0.20678172	6.06E-04	4.39E-03	ENSMUSG00000024800
Rps10	-0.490411801	0.13679393	3.37E-04	2.72E-03	ENSMUSG00000052146
Rps12	-0.828773397	0.2299975	3.14E-04	2.56E-03	ENSMUSG00000061983
Rps13-ps2	-0.570780131	0.13447131	2.19E-05	2.83E-04	ENSMUSG00000069972
Rps13-ps5	-0.966678762	0.29871668	1.21E-03	7.76E-03	ENSMUSG00000082626
Rps14	-0.548884448	0.12800091	1.80E-05	2.41E-04	ENSMUSG00000024608
Rps15	-0.452303154	0.14385121	1.67E-03	9.95E-03	ENSMUSG00000063457
Rps15a	-0.61153283	0.11157932	4.24E-08	1.25E-06	ENSMUSG00000008683
Rps18	-0.44473195	0.1671257	7.79E-03	3.39E-02	ENSMUSG00000008668
Rps19	-0.509665316	0.13343127	1.34E-04	1.27E-03	ENSMUSG00000040952
Rps19-ps11	-0.584875963	0.21942068	7.69E-03	3.36E-02	ENSMUSG00000089839
Rps19bp1	-0.452298648	0.14461475	1.76E-03	1.04E-02	ENSMUSG00000051518
Rps20	-0.509914461	0.10528311	1.28E-06	2.49E-05	ENSMUSG00000028234
Rps21	-0.496476257	0.12539888	7.52E-05	8.00E-04	ENSMUSG00000039001
Rps24	-0.618795092	0.11733179	1.34E-07	3.42E-06	ENSMUSG00000025290
Rps24-ps3	-0.799969954	0.29545092	6.78E-03	3.05E-02	ENSMUSG00000081049
Rps25-ps1	-0.789689277	0.19629546	5.75E-05	6.42E-04	ENSMUSG00000067344
Rps26	-0.453904492	0.11147598	4.67E-05	5.41E-04	ENSMUSG00000025362
Rps27l	-0.47287932	0.10915913	1.48E-05	2.03E-04	ENSMUSG00000036781
Rps28	-0.409068522	0.12552667	1.12E-03	7.25E-03	ENSMUSG00000067288
Rps3	-0.691208597	0.10990898	3.20E-10	1.63E-08	ENSMUSG00000030744
Rps3a1	-0.884796886	0.22343776	7.50E-05	7.99E-04	ENSMUSG00000028081
Rps4l	0.467628425	0.17133097	6.35E-03	2.89E-02	ENSMUSG00000063171
Rps5	-0.474074664	0.11127704	2.04E-05	2.69E-04	ENSMUSG00000012848
Rps6ka3	-0.652802797	0.24057687	6.66E-03	3.00E-02	ENSMUSG00000031309
Rps6ka4	0.454387421	0.17752018	1.05E-02	4.27E-02	ENSMUSG00000024952
Rps6kb1	-0.59320228	0.23757153	1.25E-02	4.89E-02	ENSMUSG00000020516

Rps8	-0.534046792	0.10798441	7.59E-07	1.59E-05	ENSMUSG00000047675
Rps9	-0.321923116	0.10693336	2.61E-03	1.42E-02	ENSMUSG00000006333
Rras2	-0.366934829	0.13835797	8.00E-03	3.46E-02	ENSMUSG00000055723
Rrp15	-0.953287751	0.24947305	1.33E-04	1.26E-03	ENSMUSG00000001305
Rsbn1	-0.904710371	0.18206734	6.73E-07	1.42E-05	ENSMUSG00000044098
Rsbn1l	-0.897433692	0.20234585	9.20E-06	1.35E-04	ENSMUSG00000039968
Rsf1	-1.01290802	0.29762146	6.66E-04	4.73E-03	ENSMUSG00000035623
Rsl1d1	-1.060359825	0.15439495	6.52E-12	4.64E-10	ENSMUSG00000005846
Rsph9	1.842697285	0.40421793	5.15E-06	8.27E-05	ENSMUSG00000023966
Rspry1	-0.785571992	0.27272948	3.97E-03	1.98E-02	ENSMUSG00000050079
Rsrc2	-0.709446155	0.18305819	1.06E-04	1.05E-03	ENSMUSG00000029422
Rtf1	-0.724666475	0.23146483	1.74E-03	1.03E-02	ENSMUSG00000027304
Rtf2	0.528084767	0.13990205	1.60E-04	1.47E-03	ENSMUSG00000027502
Rtl8b	0.739694011	0.1663178	8.69E-06	1.29E-04	ENSMUSG00000067924
Rtn4	-0.463265057	0.11557791	6.12E-05	6.79E-04	ENSMUSG00000020458
Rtn4rl1	1.963969271	0.40146616	9.98E-07	2.03E-05	ENSMUSG00000045287
Rtraf	-0.690360665	0.1356148	3.57E-07	8.12E-06	ENSMUSG00000021807
Runx1	-0.91639793	0.16480735	2.69E-08	8.44E-07	ENSMUSG00000022952
Runx2	0.736228725	0.25738099	4.23E-03	2.09E-02	ENSMUSG00000039153
Runx3	0.467046725	0.15921774	3.35E-03	1.73E-02	ENSMUSG00000070691
Rusc2	-0.446025249	0.14452445	2.03E-03	1.16E-02	ENSMUSG00000035969
Ruvbl2	-0.548138258	0.1608376	6.54E-04	4.68E-03	ENSMUSG00000003868
Rxra	0.389483882	0.14104836	5.76E-03	2.68E-02	ENSMUSG00000015846
S100a1	0.923666397	0.14316669	1.11E-10	6.25E-09	ENSMUSG00000044080
S100a10	0.50427844	0.10033086	5.00E-07	1.09E-05	ENSMUSG00000041959
S100a11	0.703705548	0.10735921	5.58E-11	3.41E-09	ENSMUSG00000027907
S100a13	0.964749135	0.16072018	1.94E-09	8.06E-08	ENSMUSG00000042312
S100a16	0.517999966	0.11309727	4.65E-06	7.62E-05	ENSMUSG00000074457
S100a4	1.866932931	0.13847073	1.98E-41	3.26E-38	ENSMUSG00000001020
S100a6	0.894258581	0.09281678	5.71E-22	1.34E-19	ENSMUSG00000001025
S100b	0.890088649	0.16283799	4.60E-08	1.34E-06	ENSMUSG00000033208
S1pr1	1.38171321	0.28199534	9.59E-07	1.96E-05	ENSMUSG00000045092
Saa1	-2.42544854	0.80084394	2.46E-03	1.35E-02	ENSMUSG00000074115
Saa3	-2.192345565	0.20136116	1.32E-27	5.79E-25	ENSMUSG00000040026
Sacm1l	-0.906500477	0.25214986	3.24E-04	2.63E-03	ENSMUSG00000025240
Sacs	-2.089977877	0.66674991	1.72E-03	1.02E-02	ENSMUSG00000048279
Samd4	-0.605137299	0.20663969	3.41E-03	1.76E-02	ENSMUSG00000021838
Samhd1	0.407761446	0.16087996	1.13E-02	4.50E-02	ENSMUSG00000027639
Sar1a	-0.472488821	0.12590104	1.75E-04	1.58E-03	ENSMUSG00000020088
Sar1b	-0.709989927	0.11832725	1.97E-09	8.16E-08	ENSMUSG00000020386
Sardh	1.273506799	0.26012821	9.80E-07	1.99E-05	ENSMUSG00000009614
Sars	-0.429561157	0.12019014	3.52E-04	2.80E-03	ENSMUSG00000068739
Sat1	-0.631863751	0.16831041	1.74E-04	1.57E-03	ENSMUSG00000025283

Sat2	1.392742317	0.30040784	3.55E-06	6.06E-05	ENSMUSG00000069835
Sav1	-0.411325918	0.13463144	2.25E-03	1.26E-02	ENSMUSG00000021067
Sbf2	-0.802463697	0.21118015	1.45E-04	1.35E-03	ENSMUSG00000038371
Sbno1	-0.778736366	0.2545159	2.22E-03	1.24E-02	ENSMUSG00000038095
Scaf11	-0.894441122	0.24235053	2.24E-04	1.93E-03	ENSMUSG00000033228
Scara3	0.644759427	0.18341197	4.39E-04	3.37E-03	ENSMUSG00000034463
Scara5	0.53936577	0.16130278	8.26E-04	5.68E-03	ENSMUSG00000022032
Scarb1	-0.381835325	0.1318213	3.77E-03	1.90E-02	ENSMUSG00000037936
Scarf2	1.079293865	0.19935198	6.16E-08	1.73E-06	ENSMUSG00000012017
Scd2	0.384879492	0.13738202	5.09E-03	2.43E-02	ENSMUSG00000025203
Scfd1	-0.771017826	0.26192803	3.24E-03	1.69E-02	ENSMUSG00000020952
Scmh1	-0.565024421	0.13228277	1.94E-05	2.58E-04	ENSMUSG00000000085
Scn1b	0.615557469	0.13651176	6.51E-06	1.01E-04	ENSMUSG00000019194
Sco1	-0.898952457	0.33412942	7.14E-03	3.17E-02	ENSMUSG00000069844
Scoc	-0.766959033	0.16287882	2.49E-06	4.47E-05	ENSMUSG00000063253
Scrg1	0.908968152	0.1081767	4.37E-17	5.99E-15	ENSMUSG00000031610
Scrn1	1.304319052	0.33953629	1.22E-04	1.18E-03	ENSMUSG00000019124
Scube1	1.035386656	0.2783832	2.00E-04	1.76E-03	ENSMUSG00000016763
Scyl2	-0.887309725	0.32342415	6.08E-03	2.79E-02	ENSMUSG00000069539
Sdad1	-1.033862472	0.23490879	1.08E-05	1.55E-04	ENSMUSG00000029415
Sdc1	0.479850606	0.12541184	1.30E-04	1.24E-03	ENSMUSG00000020592
Sdc3	0.931394098	0.17121231	5.33E-08	1.52E-06	ENSMUSG00000025743
Sdcbp	-0.452401277	0.12085993	1.82E-04	1.63E-03	ENSMUSG00000028249
Sde2	-0.43824252	0.17623004	1.29E-02	5.00E-02	ENSMUSG00000038806
Sdf4	0.327579372	0.11033972	2.99E-03	1.58E-02	ENSMUSG00000029076
Sdhaf4	0.577012477	0.14617466	7.90E-05	8.33E-04	ENSMUSG00000026154
Sdhc	0.560418276	0.13652753	4.05E-05	4.79E-04	ENSMUSG00000058076
Sdr39u1	0.878666254	0.19092026	4.18E-06	6.95E-05	ENSMUSG00000022223
Sdr42e1	1.901151531	0.48280333	8.23E-05	8.57E-04	ENSMUSG00000034308
Sec11c	0.435358494	0.14336668	2.39E-03	1.32E-02	ENSMUSG00000024516
Sec14l1	0.420933141	0.1556523	6.84E-03	3.07E-02	ENSMUSG00000020823
Sec22b	-0.592528475	0.10737244	3.42E-08	1.04E-06	ENSMUSG00000027879
Sec23a	-0.557237809	0.14728936	1.55E-04	1.43E-03	ENSMUSG00000020986
Sec23ip	-0.55124814	0.19791266	5.35E-03	2.53E-02	ENSMUSG00000055319
Sec24a	-0.872726212	0.20538842	2.15E-05	2.79E-04	ENSMUSG00000036391
Sec62	-1.088949651	0.32450814	7.92E-04	5.50E-03	ENSMUSG00000027706
Sec63	-0.63545383	0.22386391	4.53E-03	2.21E-02	ENSMUSG00000019802
Selenbp1	1.540333254	0.28143506	4.42E-08	1.30E-06	ENSMUSG00000068874
Selenom	0.496409588	0.11982791	3.43E-05	4.15E-04	ENSMUSG00000075702
Selenon	0.577025804	0.16561721	4.94E-04	3.71E-03	ENSMUSG00000050989
Sem1	0.304244439	0.11499156	8.15E-03	3.50E-02	ENSMUSG00000042541
Sema4b	0.912959847	0.22933245	6.86E-05	7.44E-04	ENSMUSG00000030539
Sema7a	1.465756002	0.21910689	2.24E-11	1.48E-09	ENSMUSG00000038264

Senp5	-0.86762482	0.25485375	6.63E-04	4.73E-03	ENSMUSG00000022772
Sept1	0.974869188	0.31220247	1.79E-03	1.05E-02	ENSMUSG00000000486
Sept7	-0.971061698	0.18378912	1.27E-07	3.28E-06	ENSMUSG00000001833
Sept8	0.272300146	0.10530949	9.72E-03	4.03E-02	ENSMUSG00000018398
Sept9	0.812475878	0.29883361	6.55E-03	2.96E-02	ENSMUSG00000059248
Serbp1	-0.494105875	0.10021906	8.21E-07	1.71E-05	ENSMUSG00000036371
Serhl	0.826307093	0.18954213	1.30E-05	1.83E-04	ENSMUSG00000058586
Serinc1	-0.441266587	0.14060758	1.70E-03	1.01E-02	ENSMUSG00000019877
Serp1	-0.497710195	0.10298179	1.34E-06	2.62E-05	ENSMUSG00000027808
Serpina12	2.637114661	0.37121323	1.21E-12	9.55E-11	ENSMUSG00000041567
Serpina3m	-0.780831652	0.11661678	2.15E-11	1.43E-09	ENSMUSG00000079012
Serpina3n	-0.435521163	0.12844497	6.97E-04	4.93E-03	ENSMUSG00000021091
Serpinf1	0.398622707	0.12098362	9.85E-04	6.52E-03	ENSMUSG00000000753
Serpinh1	0.691206747	0.09756268	1.39E-12	1.07E-10	ENSMUSG00000070436
Sertad3	1.170187297	0.2502299	2.92E-06	5.10E-05	ENSMUSG00000055200
Sertad4	0.879910892	0.17531696	5.19E-07	1.13E-05	ENSMUSG00000016262
Setd1b	0.679206657	0.17470916	1.01E-04	1.01E-03	ENSMUSG00000038384
Setd2	-0.879473535	0.23971808	2.44E-04	2.08E-03	ENSMUSG00000044791
Setd5	-0.607659788	0.19004283	1.39E-03	8.56E-03	ENSMUSG00000034269
Setx	-1.329363644	0.42466546	1.75E-03	1.03E-02	ENSMUSG00000043535
Sez6	-0.602292829	0.19555104	2.07E-03	1.18E-02	ENSMUSG00000000632
Sf3b1	-0.882619654	0.19655417	7.11E-06	1.09E-04	ENSMUSG00000025982
Sf3b5	-0.315348522	0.10843413	3.64E-03	1.85E-02	ENSMUSG00000078348
Sfr1	-0.473802151	0.11857775	6.45E-05	7.10E-04	ENSMUSG00000025066
Sfrp5	1.29678053	0.5040886	1.01E-02	4.15E-02	ENSMUSG00000018822
Sft2d3	0.76108168	0.24449599	1.85E-03	1.08E-02	ENSMUSG00000044982
Sfxn2	0.877882212	0.32751306	7.35E-03	3.25E-02	ENSMUSG00000025036
Sfxn3	0.583603115	0.16045251	2.76E-04	2.30E-03	ENSMUSG00000025212
Sfxn4	2.288342785	0.73079432	1.74E-03	1.03E-02	ENSMUSG00000063698
Sgk1	-0.71895072	0.11144609	1.11E-10	6.25E-09	ENSMUSG00000019970
Sgsh	1.664843703	0.37347953	8.29E-06	1.24E-04	ENSMUSG00000005043
Sh2b2	1.000002907	0.27301839	2.50E-04	2.11E-03	ENSMUSG00000005057
Sh3bgrl	-1.107098755	0.44008158	1.19E-02	4.70E-02	ENSMUSG00000031246
Sh3bp1	0.769584353	0.22893974	7.75E-04	5.41E-03	ENSMUSG00000022436
Sh3bp4	1.363254733	0.2002334	9.87E-12	6.91E-10	ENSMUSG00000036206
Sh3bp5	0.606433287	0.22676982	7.49E-03	3.29E-02	ENSMUSG00000021892
Sh3bp5l	0.699224254	0.18759935	1.94E-04	1.72E-03	ENSMUSG00000013646
Sh3gl1	0.547745079	0.1561953	4.54E-04	3.46E-03	ENSMUSG00000003200
Sh3glb1	-0.73272548	0.14193352	2.44E-07	5.75E-06	ENSMUSG00000037062
Sh3pxd2a	0.301872586	0.10929864	5.75E-03	2.67E-02	ENSMUSG00000053617
Sh3pxd2b	1.018139461	0.16587779	8.36E-10	3.86E-08	ENSMUSG00000040711
Shank1	0.671626433	0.21007333	1.39E-03	8.57E-03	ENSMUSG00000038738
Sharpin	0.464900074	0.18539899	1.22E-02	4.78E-02	ENSMUSG00000022552

Shc1	0.687886442	0.15483034	8.88E-06	1.31E-04	ENSMUSG00000042626
Shc2	0.726815409	0.28695215	1.13E-02	4.52E-02	ENSMUSG00000020312
Shisa4	0.791776214	0.15565415	3.64E-07	8.27E-06	ENSMUSG00000041889
Shld1	0.770573084	0.27983033	5.89E-03	2.72E-02	ENSMUSG00000044991
Shroom3	1.229772874	0.41497957	3.04E-03	1.60E-02	ENSMUSG00000029381
Siae	1.005134196	0.21281656	2.32E-06	4.23E-05	ENSMUSG00000001942
Sigmar1	0.445548016	0.13768755	1.21E-03	7.76E-03	ENSMUSG00000036078
Sin3b	0.312961687	0.10964908	4.31E-03	2.13E-02	ENSMUSG00000031622
Sirpa	0.550708877	0.19611772	4.98E-03	2.39E-02	ENSMUSG00000037902
Sirt2	0.42569648	0.1395728	2.29E-03	1.28E-02	ENSMUSG00000015149
Six1	0.623883814	0.18943847	9.90E-04	6.55E-03	ENSMUSG00000051367
Skil	-1.046151587	0.2505	2.96E-05	3.67E-04	ENSMUSG00000027660
Skiv2l	1.295260139	0.18577411	3.12E-12	2.35E-10	ENSMUSG00000040356
Slain2	-0.921911679	0.17977857	2.93E-07	6.77E-06	ENSMUSG00000036087
Slc10a3	1.059131244	0.24929413	2.15E-05	2.79E-04	ENSMUSG00000032806
Slc10a6	-1.17189289	0.25373003	3.86E-06	6.51E-05	ENSMUSG00000029321
Slc11a2	-0.460080116	0.17333264	7.95E-03	3.44E-02	ENSMUSG00000023030
Slc12a4	0.358737822	0.13058171	6.01E-03	2.76E-02	ENSMUSG00000017765
Slc13a5	3.388839725	0.28992467	1.46E-31	8.71E-29	ENSMUSG00000020805
Slc16a3	1.007811021	0.11722767	8.18E-18	1.20E-15	ENSMUSG00000025161
Slc1a5	0.630317662	0.11688787	6.95E-08	1.93E-06	ENSMUSG00000001918
Slc22a17	0.674016603	0.24203893	5.36E-03	2.53E-02	ENSMUSG00000022199
Slc22a18	2.148545422	0.7494868	4.15E-03	2.06E-02	ENSMUSG00000000154
Slc22a4	-2.381078482	0.47538736	5.48E-07	1.19E-05	ENSMUSG00000020334
Slc25a1	0.401552928	0.14946974	7.22E-03	3.21E-02	ENSMUSG00000003528
Slc25a10	0.977732532	0.29647308	9.74E-04	6.48E-03	ENSMUSG00000025792
Slc25a11	0.590592867	0.13949142	2.30E-05	2.96E-04	ENSMUSG00000014606
Slc25a13	1.036724327	0.39562027	8.78E-03	3.72E-02	ENSMUSG00000015112
Slc25a20	0.429634473	0.15531762	5.67E-03	2.64E-02	ENSMUSG00000032602
Slc25a22	0.55783303	0.22274534	1.23E-02	4.81E-02	ENSMUSG00000019082
Slc25a3	0.579825959	0.1059578	4.44E-08	1.31E-06	ENSMUSG00000061904
Slc25a30	-1.120243366	0.14090152	1.86E-15	2.13E-13	ENSMUSG00000022003
Slc25a33	-0.640780814	0.17801758	3.19E-04	2.59E-03	ENSMUSG00000028982
Slc25a35	1.763898817	0.55265139	1.41E-03	8.70E-03	ENSMUSG00000018740
Slc25a36	-0.604037258	0.23204261	9.24E-03	3.87E-02	ENSMUSG00000032449
Slc25a37	-1.305426774	0.13904606	6.09E-21	1.25E-18	ENSMUSG00000034248
Slc25a45	1.46581049	0.33424846	1.16E-05	1.65E-04	ENSMUSG00000024818
Slc25a46	-0.734458201	0.19274047	1.39E-04	1.31E-03	ENSMUSG00000024259
Slc27a4	0.825747506	0.213609	1.11E-04	1.08E-03	ENSMUSG00000059316
Slc29a1	0.840613004	0.11499326	2.67E-13	2.39E-11	ENSMUSG00000023942
Slc29a3	0.510396605	0.19375489	8.43E-03	3.60E-02	ENSMUSG00000020100
Slc2a13	1.737388997	0.48519168	3.43E-04	2.74E-03	ENSMUSG00000036298
Slc2a4	1.388045065	0.51088363	6.59E-03	2.97E-02	ENSMUSG00000018566

Slc2a8	0.589511444	0.16421405	3.31E-04	2.67E-03	ENSMUSG00000026791
Slc30a4	-0.800044811	0.24188049	9.41E-04	6.32E-03	ENSMUSG00000005802
Slc30a9	-0.482952417	0.18228421	8.06E-03	3.48E-02	ENSMUSG00000029221
Slc35a2	0.87888894	0.15022095	4.90E-09	1.85E-07	ENSMUSG00000031156
Slc35b2	0.653405926	0.12561176	1.97E-07	4.80E-06	ENSMUSG00000037089
Slc35b4	0.853319548	0.19420736	1.11E-05	1.59E-04	ENSMUSG00000018999
Slc36a1	0.769212083	0.2572275	2.79E-03	1.50E-02	ENSMUSG00000020261
Slc37a4	0.778540237	0.29322627	7.93E-03	3.44E-02	ENSMUSG00000032114
Slc38a10	0.464180363	0.11074521	2.77E-05	3.46E-04	ENSMUSG00000061306
Slc38a2	-0.575430894	0.11714672	9.01E-07	1.85E-05	ENSMUSG00000022462
Slc38a3	0.977015826	0.29739279	1.02E-03	6.70E-03	ENSMUSG00000010064
Slc38a4	1.149907538	0.28607604	5.83E-05	6.50E-04	ENSMUSG00000022464
Slc39a1	0.594110067	0.14860007	6.39E-05	7.05E-04	ENSMUSG00000052310
Slc39a13	0.772106787	0.16598833	3.29E-06	5.68E-05	ENSMUSG00000002105
Slc39a8	-1.436666185	0.39518798	2.78E-04	2.31E-03	ENSMUSG00000053897
Slc3a2	-0.370525003	0.11797459	1.69E-03	1.00E-02	ENSMUSG00000010095
Slc40a1	-0.435756021	0.15966609	6.35E-03	2.89E-02	ENSMUSG00000025993
Slc44a2	0.448845927	0.13480578	8.70E-04	5.94E-03	ENSMUSG00000057193
Slc49a4	0.39793974	0.14746972	6.97E-03	3.12E-02	ENSMUSG00000022848
Slc4a2	1.347928644	0.25080399	7.68E-08	2.12E-06	ENSMUSG00000028962
Slc4a7	-1.52338876	0.24606167	5.98E-10	2.88E-08	ENSMUSG00000021733
Slc50a1	0.507500183	0.18992537	7.54E-03	3.31E-02	ENSMUSG00000027953
Slc6a6	1.084667619	0.19980616	5.68E-08	1.61E-06	ENSMUSG00000030096
Slc7a11	-1.563545955	0.25648673	1.09E-09	4.79E-08	ENSMUSG00000027737
Slc7a8	1.6599332	0.60147405	5.78E-03	2.69E-02	ENSMUSG00000022180
Slc8a3	2.436045455	0.45922381	1.13E-07	2.94E-06	ENSMUSG00000079055
Slc9a1	0.600770233	0.22831669	8.51E-03	3.62E-02	ENSMUSG00000028854
Slc9a9	1.376092548	0.41785898	9.91E-04	6.55E-03	ENSMUSG00000031129
Slco4a1	1.310355593	0.36717399	3.59E-04	2.85E-03	ENSMUSG00000038963
Slf2	-0.916457294	0.28812083	1.47E-03	8.96E-03	ENSMUSG00000036097
Slfn2	-0.877705344	0.33956693	9.74E-03	4.03E-02	ENSMUSG00000072620
Slit3	0.766601133	0.25343228	2.49E-03	1.37E-02	ENSMUSG00000056427
Slpi	-0.95961713	0.11608697	1.38E-16	1.78E-14	ENSMUSG00000017002
Sltm	-1.082954495	0.25258906	1.81E-05	2.41E-04	ENSMUSG00000032212
Slu7	-0.818310865	0.24012847	6.55E-04	4.68E-03	ENSMUSG00000020409
Slx1b	1.005243279	0.2851789	4.24E-04	3.28E-03	ENSMUSG00000059772
Smad3	0.571794593	0.13831828	3.57E-05	4.28E-04	ENSMUSG00000032402
Smad4	-0.465810125	0.15595118	2.82E-03	1.51E-02	ENSMUSG00000024515
Smad6	1.119573409	0.25345111	9.99E-06	1.45E-04	ENSMUSG00000036867
Smap1	-0.489721202	0.15838826	1.99E-03	1.14E-02	ENSMUSG00000026155
Smarca2	-0.565009523	0.21960079	1.01E-02	4.14E-02	ENSMUSG00000024921
Smarca5	-0.825115095	0.24945436	9.41E-04	6.32E-03	ENSMUSG00000031715
Smarcc1	-0.882874834	0.22807177	1.08E-04	1.06E-03	ENSMUSG00000032481

Smarcc2	1.035208164	0.16010964	1.01E-10	5.80E-09	ENSMUSG00000025369
Smarcd3	1.449781277	0.1813439	1.30E-15	1.53E-13	ENSMUSG00000028949
Smc1a	-0.789334231	0.21570281	2.53E-04	2.13E-03	ENSMUSG00000041133
Smc2	-1.105268329	0.43920041	1.19E-02	4.70E-02	ENSMUSG00000028312
Smc3	-0.91595101	0.28314442	1.22E-03	7.78E-03	ENSMUSG00000024974
Smc4	-1.04503155	0.25534467	4.27E-05	5.02E-04	ENSMUSG00000034349
Smc5	-1.7323778	0.39144695	9.62E-06	1.40E-04	ENSMUSG00000024943
Smc6	-1.174445385	0.44409842	8.18E-03	3.51E-02	ENSMUSG00000020608
Smg1	-1.199909996	0.28556391	2.65E-05	3.33E-04	ENSMUSG00000030655
Smim1	0.933762425	0.14364481	8.01E-11	4.70E-09	ENSMUSG00000078350
Smim10l1	-0.484127553	0.15988867	2.46E-03	1.36E-02	ENSMUSG00000072704
Smim12	0.423056709	0.14956547	4.68E-03	2.27E-02	ENSMUSG00000042380
Smim14	0.342422552	0.100337	6.43E-04	4.62E-03	ENSMUSG00000037822
Smim19	0.446181274	0.16282493	6.14E-03	2.81E-02	ENSMUSG00000031534
Smim26	-0.383967893	0.14513568	8.16E-03	3.50E-02	ENSMUSG00000074754
Smim27	0.726992491	0.15700316	3.65E-06	6.21E-05	ENSMUSG00000028407
Smim4	0.859674074	0.14430343	2.56E-09	1.04E-07	ENSMUSG00000058351
Smim5	3.341570327	0.34079615	1.07E-22	2.93E-20	ENSMUSG00000048442
Smndc1	-0.688376955	0.15379893	7.61E-06	1.15E-04	ENSMUSG00000025024
Smo	0.559072562	0.19533659	4.21E-03	2.08E-02	ENSMUSG00000001761
Smoc1	1.31592565	0.23129167	1.27E-08	4.44E-07	ENSMUSG00000021136
Smox	-0.57358204	0.14603055	8.57E-05	8.85E-04	ENSMUSG00000027333
Smpd1	0.567489751	0.15109401	1.73E-04	1.56E-03	ENSMUSG00000037049
Smpd2	0.97869633	0.18544733	1.31E-07	3.37E-06	ENSMUSG00000019822
Smpd3	2.29901889	0.14710055	4.63E-55	1.22E-51	ENSMUSG00000031906
Sms	-0.937050506	0.36764279	1.08E-02	4.37E-02	ENSMUSG00000071708
Smtn	0.782709838	0.13825868	1.50E-08	5.06E-07	ENSMUSG00000020439
Smu1	-0.321107693	0.12435202	9.82E-03	4.06E-02	ENSMUSG00000028409
Snai1	1.286607615	0.13578135	2.65E-21	5.72E-19	ENSMUSG00000042821
Snap23	-0.848195506	0.17591236	1.42E-06	2.75E-05	ENSMUSG00000027287
Snap47	0.864696201	0.10801513	1.19E-15	1.41E-13	ENSMUSG00000009894
Snapc3	-0.624570506	0.2299321	6.60E-03	2.98E-02	ENSMUSG00000028483
Snapc5	0.797880481	0.19920622	6.19E-05	6.87E-04	ENSMUSG00000032398
Snapin	0.751273585	0.17697939	2.19E-05	2.83E-04	ENSMUSG00000001018
Sned1	1.176943124	0.21646868	5.42E-08	1.54E-06	ENSMUSG00000047793
Snhg1	1.83341581	0.41079853	8.08E-06	1.21E-04	ENSMUSG00000107994
Snhg11	-1.475571093	0.45813715	1.28E-03	8.07E-03	ENSMUSG00000044349
Snhg12	-1.320000006	0.1428115	2.40E-20	4.57E-18	ENSMUSG00000086290
Snhg14	-0.573917326	0.138209	3.29E-05	4.03E-04	ENSMUSG00000100215
Snhg15	-1.277160242	0.15263396	5.89E-17	7.91E-15	ENSMUSG00000085156
Snhg5	1.17990649	0.30190285	9.30E-05	9.50E-04	ENSMUSG00000097180
Snhg8	-2.096868188	0.77820187	7.05E-03	3.14E-02	ENSMUSG00000104876
Snrnp48	-1.649483731	0.20751907	1.89E-15	2.14E-13	ENSMUSG00000021431

Snrpa1	-1.082864478	0.16745819	1.00E-10	5.79E-09	ENSMUSG00000030512
Snrpb2	-0.344147456	0.11122555	1.97E-03	1.13E-02	ENSMUSG00000008333
Snrpd1	-0.365113286	0.1221899	2.81E-03	1.50E-02	ENSMUSG00000002477
Snrpd2	-0.318358443	0.12700064	1.22E-02	4.79E-02	ENSMUSG00000040824
Snrpd3	-0.478668171	0.13038558	2.41E-04	2.06E-03	ENSMUSG00000020180
Snrpg	-0.491181354	0.16840105	3.54E-03	1.81E-02	ENSMUSG00000057278
Snta1	0.99267688	0.13028661	2.55E-14	2.47E-12	ENSMUSG00000027488
Snw1	-0.770881105	0.14979718	2.66E-07	6.24E-06	ENSMUSG00000021039
Snx10	-1.08128463	0.28574802	1.54E-04	1.42E-03	ENSMUSG00000038301
Snx16	-1.002300656	0.27364608	2.50E-04	2.11E-03	ENSMUSG00000027534
Snx18	-0.666009159	0.15514228	1.76E-05	2.37E-04	ENSMUSG00000042364
Snx32	1.022172842	0.34131787	2.75E-03	1.48E-02	ENSMUSG00000056185
Snx33	0.5501379	0.17833347	2.04E-03	1.16E-02	ENSMUSG00000032733
Snx5	-0.397986927	0.14492994	6.03E-03	2.77E-02	ENSMUSG00000027423
Snx7	0.668801566	0.25577231	8.93E-03	3.77E-02	ENSMUSG00000028007
Snx8	0.378415455	0.14604193	9.57E-03	3.98E-02	ENSMUSG00000029560
Socs2	-0.820821396	0.16094605	3.40E-07	7.76E-06	ENSMUSG00000020027
Socs4	-1.18879348	0.28370848	2.79E-05	3.48E-04	ENSMUSG00000048379
Sod3	0.497081013	0.18061221	5.92E-03	2.73E-02	ENSMUSG00000072941
Son	-0.398889972	0.13734753	3.68E-03	1.87E-02	ENSMUSG00000022961
Sort1	0.805601538	0.22326949	3.08E-04	2.52E-03	ENSMUSG00000068747
Sos1	-0.997929087	0.37485293	7.76E-03	3.38E-02	ENSMUSG00000024241
Sowahc	2.28819436	0.61612325	2.04E-04	1.79E-03	ENSMUSG00000097993
Sox11	-2.225946976	0.83878448	7.96E-03	3.45E-02	ENSMUSG00000063632
Sox4	-0.822537832	0.14061446	4.93E-09	1.86E-07	ENSMUSG00000076431
Sp3	-0.967904278	0.27688154	4.73E-04	3.58E-03	ENSMUSG00000027109
Sp3os	0.838523048	0.23310992	3.22E-04	2.62E-03	ENSMUSG00000063714
Sp7	3.158145914	0.23483849	3.16E-41	4.62E-38	ENSMUSG00000060284
Spa17	0.858932971	0.22577068	1.42E-04	1.33E-03	ENSMUSG00000001948
Spag9	-0.91196375	0.26464285	5.69E-04	4.17E-03	ENSMUSG00000020859
Sparc	0.471709921	0.09483655	6.56E-07	1.39E-05	ENSMUSG00000018593
Spata13	0.743139187	0.24555113	2.47E-03	1.36E-02	ENSMUSG00000021990
Spata24	0.788667747	0.3032445	9.30E-03	3.88E-02	ENSMUSG00000024352
Spc24	0.963977763	0.36008889	7.43E-03	3.27E-02	ENSMUSG00000074476
Spc25	-0.771602954	0.24736737	1.81E-03	1.06E-02	ENSMUSG00000005233
Spg7	0.438600063	0.15734277	5.31E-03	2.51E-02	ENSMUSG00000000738
Spice1	-1.049422508	0.39155087	7.36E-03	3.25E-02	ENSMUSG00000043065
Spink10	-0.670749998	0.2110908	1.49E-03	9.04E-03	ENSMUSG00000044176
Spns1	0.541720976	0.21138279	1.04E-02	4.24E-02	ENSMUSG00000030741
Spns2	2.359918114	0.30171939	5.22E-15	5.58E-13	ENSMUSG00000040447
Spock2	0.861081091	0.22072847	9.58E-05	9.71E-04	ENSMUSG00000058297
Spon1	0.652221048	0.25789511	1.14E-02	4.56E-02	ENSMUSG00000038156
Spop	-0.452280162	0.14081952	1.32E-03	8.25E-03	ENSMUSG00000057522

Spopl	-0.973125519	0.34814732	5.19E-03	2.46E-02	ENSMUSG00000026771
Spp1	-0.823071491	0.14670688	2.02E-08	6.56E-07	ENSMUSG00000029304
Sppl2b	0.807453171	0.20533953	8.41E-05	8.72E-04	ENSMUSG00000035206
Sppl3	0.376236621	0.13704151	6.04E-03	2.78E-02	ENSMUSG00000029550
Spr	0.339438406	0.12679006	7.42E-03	3.27E-02	ENSMUSG00000033735
Spry1	1.009270646	0.25265738	6.48E-05	7.11E-04	ENSMUSG00000037211
Spsb2	0.536603178	0.18249824	3.28E-03	1.70E-02	ENSMUSG00000038451
Sptbn1	-0.514975438	0.1526901	7.44E-04	5.23E-03	ENSMUSG00000020315
Sptlc1	0.453719481	0.17537046	9.68E-03	4.02E-02	ENSMUSG00000021468
Sptlc2	-0.616443711	0.18459694	8.40E-04	5.77E-03	ENSMUSG00000021036
Sptssa	0.547248686	0.21354655	1.04E-02	4.24E-02	ENSMUSG00000044408
Spty2d1	-0.754399673	0.26539872	4.48E-03	2.19E-02	ENSMUSG00000049516
Sqstm1	-0.278834386	0.10501763	7.93E-03	3.44E-02	ENSMUSG00000015837
Sra1	-0.438553603	0.11236702	9.51E-05	9.66E-04	ENSMUSG00000006050
Srd5a3	0.47196225	0.18559243	1.10E-02	4.43E-02	ENSMUSG00000029233
Srebf1	0.636908408	0.24848411	1.04E-02	4.24E-02	ENSMUSG00000020538
Srek1	-0.796721051	0.25011136	1.45E-03	8.87E-03	ENSMUSG00000032621
Srfbp1	-1.457231295	0.31474842	3.66E-06	6.22E-05	ENSMUSG00000024528
Srgap3	-0.9726802	0.22469695	1.50E-05	2.06E-04	ENSMUSG00000030257
Srgn	-0.560736027	0.22168498	1.14E-02	4.56E-02	ENSMUSG00000020077
Sri	0.369725345	0.11297631	1.07E-03	6.96E-03	ENSMUSG00000003161
Srm	-0.343003713	0.11446894	2.73E-03	1.47E-02	ENSMUSG00000006442
Srp19	-0.686813572	0.1086165	2.56E-10	1.34E-08	ENSMUSG00000014504
Srp72	-0.747183427	0.15042881	6.80E-07	1.43E-05	ENSMUSG00000036323
Srp9	-0.411710448	0.16517466	1.27E-02	4.94E-02	ENSMUSG00000026511
Srpk1	-0.488143258	0.18243399	7.46E-03	3.28E-02	ENSMUSG00000004865
Srpk2	-0.768273352	0.24325633	1.59E-03	9.53E-03	ENSMUSG00000062604
Srpr	-0.614850666	0.20123496	2.25E-03	1.26E-02	ENSMUSG00000032042
Srpx	0.838618229	0.19638786	1.95E-05	2.59E-04	ENSMUSG00000090084
Srpx2	0.715740034	0.11491947	4.72E-10	2.34E-08	ENSMUSG00000031253
Srsf10	-0.93083313	0.19612655	2.07E-06	3.84E-05	ENSMUSG00000028676
Srsf11	-1.038433626	0.16999777	1.01E-09	4.49E-08	ENSMUSG00000055436
Srsf2	0.777402716	0.14041137	3.08E-08	9.53E-07	ENSMUSG00000034120
Srsf9	0.532971097	0.10934714	1.09E-06	2.18E-05	ENSMUSG00000029538
Srxn1	-0.300489795	0.09969291	2.58E-03	1.41E-02	ENSMUSG00000032802
Ssb	-1.052537781	0.39194733	7.24E-03	3.21E-02	ENSMUSG00000068882
Ssbp3	0.448682269	0.12593921	3.67E-04	2.90E-03	ENSMUSG00000061887
Ssbp4	0.427297168	0.14218251	2.65E-03	1.44E-02	ENSMUSG00000070003
Ssh2	-0.645851305	0.14559698	9.17E-06	1.35E-04	ENSMUSG00000037926
Sspn	0.565979974	0.19903682	4.46E-03	2.18E-02	ENSMUSG00000030255
Ssr3	-0.305442151	0.1097711	5.39E-03	2.54E-02	ENSMUSG00000027828
St13	-0.344091994	0.11084993	1.91E-03	1.10E-02	ENSMUSG00000022403
St3gal1	0.405075058	0.13505993	2.71E-03	1.46E-02	ENSMUSG00000013846

St3gal2	0.514425791	0.15997942	1.30E-03	8.18E-03	ENSMUSG00000031749
St3gal5	1.121369339	0.27610475	4.88E-05	5.59E-04	ENSMUSG00000056091
St5	0.720359659	0.22619	1.45E-03	8.88E-03	ENSMUSG00000031024
St6galnac4	1.061902809	0.13297375	1.40E-15	1.63E-13	ENSMUSG00000079442
St6galnac6	0.388037729	0.15464644	1.21E-02	4.77E-02	ENSMUSG00000026811
St8sia2	1.89464104	0.33731953	1.95E-08	6.39E-07	ENSMUSG00000025789
Stab1	1.527754187	0.4962379	2.08E-03	1.18E-02	ENSMUSG00000042286
Stag2	-0.841854346	0.22104786	1.40E-04	1.31E-03	ENSMUSG00000025862
Stam	-0.823665195	0.2182912	1.61E-04	1.47E-03	ENSMUSG00000026718
Stard5	-0.37856634	0.15100561	1.22E-02	4.79E-02	ENSMUSG00000046027
Stat1	1.066114536	0.31735954	7.81E-04	5.44E-03	ENSMUSG00000026104
Stat5a	0.917566991	0.29420188	1.82E-03	1.06E-02	ENSMUSG00000004043
Stat5b	0.7786034	0.22894734	6.72E-04	4.77E-03	ENSMUSG00000020919
Stat6	0.536722378	0.14704658	2.62E-04	2.20E-03	ENSMUSG00000002147
Stc2	0.95872359	0.21623992	9.27E-06	1.35E-04	ENSMUSG00000020303
Steap1	-0.963965653	0.16314452	3.45E-09	1.35E-07	ENSMUSG00000015652
Steap2	-1.027058579	0.27518617	1.90E-04	1.69E-03	ENSMUSG00000015653
Stim1	0.803418142	0.1675504	1.63E-06	3.11E-05	ENSMUSG00000030987
Stimate	0.453456994	0.17842888	1.10E-02	4.44E-02	ENSMUSG00000006526
Stip1	-0.49462284	0.13401569	2.24E-04	1.93E-03	ENSMUSG00000024966
Stk25	0.615213017	0.15428034	6.67E-05	7.27E-04	ENSMUSG00000026277
Stk3	-1.047722323	0.29244101	3.40E-04	2.73E-03	ENSMUSG00000022329
Stk39	-0.770590564	0.26776397	4.00E-03	2.00E-02	ENSMUSG00000027030
Stk40	0.579417988	0.13840649	2.83E-05	3.53E-04	ENSMUSG00000042608
Strada	0.815709182	0.28297205	3.94E-03	1.98E-02	ENSMUSG00000069631
Strap	-0.516294145	0.13987991	2.23E-04	1.93E-03	ENSMUSG00000030224
Strbp	-0.849007631	0.21551402	8.17E-05	8.53E-04	ENSMUSG00000026915
Strn3	-0.885945278	0.22863598	1.07E-04	1.05E-03	ENSMUSG00000020954
Stx12	-0.503469228	0.15964482	1.61E-03	9.66E-03	ENSMUSG00000028879
Stx5a	-0.375709129	0.14551647	9.83E-03	4.06E-02	ENSMUSG00000010110
Stx7	-0.380870327	0.14372274	8.05E-03	3.47E-02	ENSMUSG00000019998
Stxbp2	0.997382481	0.25595912	9.75E-05	9.85E-04	ENSMUSG00000004626
Suclg1	0.498514641	0.12688637	8.54E-05	8.83E-04	ENSMUSG00000052738
Suco	-0.933466293	0.21963369	2.14E-05	2.78E-04	ENSMUSG00000040297
Sugct	1.012163604	0.31644878	1.38E-03	8.55E-03	ENSMUSG00000055137
Sugt1	-0.520237668	0.13860934	1.75E-04	1.58E-03	ENSMUSG00000022024
Sulf2	0.385823189	0.12692529	2.37E-03	1.31E-02	ENSMUSG00000006800
Sult1a1	2.002849039	0.20060446	1.79E-23	5.61E-21	ENSMUSG00000030711
Sult5a1	1.299663676	0.1328947	1.38E-22	3.70E-20	ENSMUSG00000000739
Sumf1	0.510161234	0.19183929	7.83E-03	3.40E-02	ENSMUSG00000030101
Sun2	0.541143708	0.12000802	6.51E-06	1.01E-04	ENSMUSG00000042524
Susd5	1.14816965	0.24690022	3.31E-06	5.69E-05	ENSMUSG00000086596
Suz12	-1.109529542	0.39004603	4.45E-03	2.18E-02	ENSMUSG00000017548

Svbp	0.506443183	0.14265578	3.85E-04	3.02E-03	ENSMUSG00000028643
Svep1	0.917552346	0.30138173	2.33E-03	1.30E-02	ENSMUSG00000028369
Swt1	-1.278622144	0.27950036	4.77E-06	7.79E-05	ENSMUSG00000052748
Syncrip	-0.693441006	0.16726965	3.39E-05	4.12E-04	ENSMUSG00000032423
Syngr2	0.556798991	0.1444844	1.16E-04	1.13E-03	ENSMUSG00000048277
Synpo	0.399832262	0.13499536	3.06E-03	1.61E-02	ENSMUSG00000043079
Syt12	0.86766784	0.27251599	1.45E-03	8.89E-03	ENSMUSG00000049303
Syt3	2.061536463	0.5208501	7.56E-05	8.02E-04	ENSMUSG00000030731
Syt7	-1.638934781	0.58058546	4.76E-03	2.30E-02	ENSMUSG00000024743
Syt8	2.030573366	0.428034	2.10E-06	3.86E-05	ENSMUSG00000031098
Syvn1	0.581286815	0.20210948	4.03E-03	2.00E-02	ENSMUSG00000024807
Tab2	-0.711245606	0.19061079	1.90E-04	1.69E-03	ENSMUSG00000015755
Taco1os	1.041247704	0.39484508	8.36E-03	3.58E-02	ENSMUSG00000085255
Taf1	-0.820330348	0.30634522	7.41E-03	3.27E-02	ENSMUSG00000031314
Taf15	-0.832699294	0.21777826	1.32E-04	1.25E-03	ENSMUSG00000020680
Taf1a	-1.588233183	0.41894772	1.50E-04	1.39E-03	ENSMUSG00000072258
Taf1d	-1.655103397	0.20508228	7.00E-16	8.46E-14	ENSMUSG00000031939
Taf6l	0.747530686	0.11644648	1.37E-10	7.53E-09	ENSMUSG00000003680
Tagln2	0.481469342	0.13028061	2.19E-04	1.90E-03	ENSMUSG00000026547
Taldo1	-0.778407768	0.11201525	3.68E-12	2.73E-10	ENSMUSG00000025503
Tank	-0.78808256	0.23684564	8.77E-04	5.97E-03	ENSMUSG00000064289
Taok1	-0.649503149	0.19714076	9.86E-04	6.52E-03	ENSMUSG00000017291
Taok2	0.89217484	0.24305408	2.42E-04	2.06E-03	ENSMUSG00000059981
Tardbp	-1.023934417	0.14909242	6.52E-12	4.64E-10	ENSMUSG00000041459
Tars	-0.723225564	0.12966697	2.44E-08	7.76E-07	ENSMUSG00000022241
Tatdn1	-0.840740929	0.21493909	9.17E-05	9.37E-04	ENSMUSG00000050891
Tax1bp1	-1.14496295	0.20130004	1.29E-08	4.46E-07	ENSMUSG00000004535
Taz	0.554327522	0.19266421	4.01E-03	2.00E-02	ENSMUSG00000009995
Tbc1d15	-1.548260881	0.17107966	1.43E-19	2.62E-17	ENSMUSG00000020130
Tbc1d23	-1.212793545	0.33582351	3.05E-04	2.49E-03	ENSMUSG00000022749
Tbc1d31	-1.698411071	0.51673782	1.01E-03	6.67E-03	ENSMUSG00000022364
Tbc1d7	0.786018961	0.24331746	1.24E-03	7.87E-03	ENSMUSG00000021368
Tbc1d9b	0.618992564	0.13310791	3.31E-06	5.69E-05	ENSMUSG00000036644
Tbca	-0.532747673	0.1242085	1.79E-05	2.40E-04	ENSMUSG00000042043
Tbcd	0.422247795	0.16077561	8.63E-03	3.67E-02	ENSMUSG00000039230
Tbl1x	-0.388336968	0.15341389	1.14E-02	4.54E-02	ENSMUSG00000025246
Tbpl1	-0.787770458	0.14046582	2.04E-08	6.63E-07	ENSMUSG00000071359
Tbrg1	-0.62179029	0.11166503	2.57E-08	8.12E-07	ENSMUSG00000011114
Tbx2	1.042835743	0.38015081	6.08E-03	2.79E-02	ENSMUSG00000000093
Tcaim	-1.126034687	0.37651976	2.78E-03	1.49E-02	ENSMUSG00000046603
Tcea1	-1.247650216	0.26858153	3.40E-06	5.82E-05	ENSMUSG00000033813
Tceal9	-0.69997658	0.12231638	1.05E-08	3.72E-07	ENSMUSG00000042712
Tcerg1	-1.44822234	0.25875873	2.18E-08	6.99E-07	ENSMUSG00000024498

Tcf19	0.889133425	0.24269434	2.49E-04	2.11E-03	ENSMUSG00000050410
Tcf25	0.405453249	0.10351197	8.97E-05	9.19E-04	ENSMUSG00000001472
Tcf7	1.136825955	0.3385383	7.85E-04	5.46E-03	ENSMUSG00000000782
Tcf7l1	0.798392492	0.1742087	4.58E-06	7.52E-05	ENSMUSG00000055799
Tcn2	0.612041918	0.14800548	3.55E-05	4.26E-04	ENSMUSG00000020432
Tcp1	-0.538164521	0.13135098	4.18E-05	4.94E-04	ENSMUSG00000068039
Tcta	0.482529245	0.18904501	1.07E-02	4.34E-02	ENSMUSG00000039461
Tctn2	0.923330904	0.35486265	9.27E-03	3.88E-02	ENSMUSG00000029386
Tdp2	-1.012921138	0.29611885	6.25E-04	4.50E-03	ENSMUSG00000035958
Tead1	-0.755443217	0.21623717	4.77E-04	3.60E-03	ENSMUSG00000055320
Tecr	0.489790537	0.12957582	1.57E-04	1.44E-03	ENSMUSG00000031708
Tent4b	-1.586434246	0.42808759	2.11E-04	1.84E-03	ENSMUSG00000036779
Tent5c	1.859307603	0.31811283	5.07E-09	1.91E-07	ENSMUSG00000044468
Tex261	0.646264073	0.12391827	1.84E-07	4.50E-06	ENSMUSG00000014748
Tex264	0.471746118	0.14663062	1.29E-03	8.15E-03	ENSMUSG00000040813
Tex30	-0.770757628	0.28866587	7.58E-03	3.32E-02	ENSMUSG00000026049
Tfip11	0.5385594	0.163356	9.78E-04	6.50E-03	ENSMUSG00000029345
Tgfb1	0.963246221	0.12627356	2.38E-14	2.32E-12	ENSMUSG00000002603
Tgfb1i1	0.711227107	0.1779636	6.43E-05	7.08E-04	ENSMUSG00000030782
Tgfb3	0.451617199	0.17354211	9.26E-03	3.87E-02	ENSMUSG00000021253
Tgif1	-0.903884739	0.15994037	1.59E-08	5.32E-07	ENSMUSG00000047407
Tgoln1	-0.789335569	0.15722397	5.15E-07	1.13E-05	ENSMUSG00000056429
Tgs1	-1.082132536	0.32841182	9.84E-04	6.52E-03	ENSMUSG00000028233
Thbd	-0.612838899	0.21553388	4.46E-03	2.18E-02	ENSMUSG00000074743
Thbs1	0.724834667	0.18272016	7.28E-05	7.81E-04	ENSMUSG00000040152
Thbs2	0.628073692	0.19063561	9.86E-04	6.52E-03	ENSMUSG00000023885
Thbs4	0.899744835	0.15994598	1.85E-08	6.12E-07	ENSMUSG00000021702
Thnsl2	0.976807991	0.29559456	9.51E-04	6.36E-03	ENSMUSG00000054474
Thoc1	-1.215048944	0.30138581	5.54E-05	6.25E-04	ENSMUSG00000024287
Thoc6	0.729843113	0.2037686	3.41E-04	2.74E-03	ENSMUSG00000041319
Thoc7	-0.422362713	0.15243293	5.59E-03	2.62E-02	ENSMUSG00000053453
Thra	0.522266914	0.11998373	1.34E-05	1.88E-04	ENSMUSG00000058756
Thrsp	1.502719305	0.33643707	7.95E-06	1.19E-04	ENSMUSG00000035686
Thsd4	2.284924753	0.51419581	8.84E-06	1.31E-04	ENSMUSG00000032289
Thumpd1	-0.704504205	0.24650211	4.26E-03	2.10E-02	ENSMUSG00000030942
Thy1	0.786511491	0.1461908	7.45E-08	2.06E-06	ENSMUSG00000032011
Thyn1	-0.399824676	0.15216284	8.60E-03	3.66E-02	ENSMUSG00000035443
Tial1	-0.95561719	0.18098257	1.29E-07	3.33E-06	ENSMUSG00000030846
Tigar	-0.726176822	0.28273796	1.02E-02	4.19E-02	ENSMUSG00000038028
Tigd2	-0.679378207	0.18334734	2.11E-04	1.84E-03	ENSMUSG00000049232
Tigd5	-0.682845856	0.17598678	1.04E-04	1.03E-03	ENSMUSG00000103558
Timm10	-0.590945234	0.14191886	3.13E-05	3.84E-04	ENSMUSG00000027076
Timm22	0.560168739	0.17829408	1.68E-03	1.00E-02	ENSMUSG00000020843

Timp1	0.516004012	0.09672361	9.56E-08	2.53E-06	ENSMUSG00000001131
Timp2	0.686193465	0.11641042	3.76E-09	1.45E-07	ENSMUSG00000017466
Timp3	0.564570758	0.11846657	1.88E-06	3.53E-05	ENSMUSG00000020044
Timp4	1.02304261	0.18115532	1.63E-08	5.43E-07	ENSMUSG00000030317
Tiprl	-0.670576579	0.17461733	1.23E-04	1.18E-03	ENSMUSG00000040843
Tk1	1.126081502	0.26334802	1.90E-05	2.53E-04	ENSMUSG00000025574
Tkt	-0.442143179	0.15326982	3.92E-03	1.97E-02	ENSMUSG00000021957
Tlcd1	0.583161626	0.19533309	2.83E-03	1.51E-02	ENSMUSG00000019437
Tlcd2	0.641301057	0.14362699	8.01E-06	1.20E-04	ENSMUSG00000038217
Tle2	1.559530021	0.53844674	3.78E-03	1.90E-02	ENSMUSG00000034771
Tlk2	-1.073078103	0.2092514	2.93E-07	6.77E-06	ENSMUSG00000020694
Tln1	0.616385702	0.11760762	1.60E-07	3.99E-06	ENSMUSG00000028465
Tln2	0.734786342	0.17281396	2.12E-05	2.77E-04	ENSMUSG00000052698
Tlr4	-1.079644765	0.26750352	5.44E-05	6.15E-04	ENSMUSG00000039005
Tm2d2	0.461660083	0.12972947	3.73E-04	2.94E-03	ENSMUSG00000031556
Tma16	-1.125685029	0.41725007	6.98E-03	3.12E-02	ENSMUSG00000025591
Tmbim4	0.380761043	0.11836619	1.30E-03	8.15E-03	ENSMUSG00000020225
Tmed1	0.608340079	0.16936496	3.28E-04	2.66E-03	ENSMUSG00000032180
Tmed2	-0.465365998	0.13666738	6.61E-04	4.72E-03	ENSMUSG00000029390
Tmed5	-1.198985471	0.28375455	2.38E-05	3.05E-04	ENSMUSG00000063406
Tmeff1	-1.158157239	0.29298943	7.72E-05	8.16E-04	ENSMUSG00000028347
Tmem100	1.303269109	0.28995583	6.97E-06	1.07E-04	ENSMUSG00000069763
Tmem104	1.049598294	0.22928328	4.70E-06	7.69E-05	ENSMUSG00000045980
Tmem109	0.520640192	0.12299185	2.30E-05	2.96E-04	ENSMUSG00000034659
Tmem11	-0.79182258	0.13640987	6.45E-09	2.35E-07	ENSMUSG00000043284
Tmem119	2.757364627	0.13862939	4.95E-88	3.26E-84	ENSMUSG00000054675
Tmem123	-0.363106704	0.14129363	1.02E-02	4.17E-02	ENSMUSG00000050912
Tmem127	0.823057241	0.16218397	3.88E-07	8.71E-06	ENSMUSG00000034850
Tmem132a	0.882843965	0.32120305	5.99E-03	2.76E-02	ENSMUSG00000024736
Tmem143	0.914038491	0.24051225	1.44E-04	1.35E-03	ENSMUSG00000002781
Tmem147	0.273425063	0.10748348	1.10E-02	4.42E-02	ENSMUSG00000006315
Tmem14a	1.311989584	0.32576269	5.64E-05	6.33E-04	ENSMUSG00000025933
Tmem150a	0.750283297	0.14046192	9.22E-08	2.45E-06	ENSMUSG00000055912
Tmem158	0.917840701	0.21711157	2.36E-05	3.02E-04	ENSMUSG00000054871
Tmem159	-0.367896646	0.14707402	1.24E-02	4.84E-02	ENSMUSG00000030917
Tmem160	0.361214297	0.12526097	3.93E-03	1.97E-02	ENSMUSG00000019158
Tmem167	-0.495769628	0.11811537	2.70E-05	3.39E-04	ENSMUSG00000012422
Tmem173	0.638738436	0.23434632	6.42E-03	2.91E-02	ENSMUSG00000024349
Tmem175	1.016421837	0.21698911	2.81E-06	4.97E-05	ENSMUSG00000013495
Tmem19	0.662977598	0.18536466	3.48E-04	2.78E-03	ENSMUSG00000069520
Tmem198b	1.016969322	0.22663211	7.21E-06	1.10E-04	ENSMUSG00000047090
Tmem200c	0.700517012	0.25009786	5.09E-03	2.43E-02	ENSMUSG00000095041
Tmem205	0.361835431	0.14322965	1.15E-02	4.59E-02	ENSMUSG00000040883

Tmem208	0.435604294	0.14109814	2.02E-03	1.15E-02	ENSMUSG00000014856
Tmem219	0.504059537	0.14988248	7.71E-04	5.39E-03	ENSMUSG00000060538
Tmem220	1.761552292	0.53885189	1.08E-03	7.03E-03	ENSMUSG00000050270
Tmem229b	1.296134999	0.31916002	4.88E-05	5.59E-04	ENSMUSG00000046157
Tmem238	0.732742791	0.23096971	1.51E-03	9.18E-03	ENSMUSG00000030431
Tmem245	-0.763133503	0.28284961	6.98E-03	3.12E-02	ENSMUSG00000055296
Tmem246	1.842564861	0.44786346	3.89E-05	4.62E-04	ENSMUSG00000039611
Tmem248	-0.310452026	0.12052686	1.00E-02	4.12E-02	ENSMUSG00000053094
Tmem250-ps	0.594318008	0.16866079	4.25E-04	3.29E-03	ENSMUSG00000087679
Tmem259	0.486581566	0.15060373	1.23E-03	7.87E-03	ENSMUSG00000013858
Tmem263	-0.564315244	0.20223658	5.26E-03	2.49E-02	ENSMUSG00000060935
Tmem33	-0.367072326	0.12709388	3.87E-03	1.95E-02	ENSMUSG00000037720
Tmem38b	-0.613649796	0.18727905	1.05E-03	6.87E-03	ENSMUSG00000028420
Tmem42	1.540174919	0.24807756	5.35E-10	2.61E-08	ENSMUSG00000066233
Tmem45a	0.565100384	0.12709173	8.73E-06	1.29E-04	ENSMUSG00000022754
Tmem47	-1.067798194	0.27265302	8.99E-05	9.20E-04	ENSMUSG00000025666
Tmem59	0.413168373	0.10094	4.25E-05	5.01E-04	ENSMUSG00000028618
Tmem63b	0.58902105	0.16376872	3.22E-04	2.62E-03	ENSMUSG00000036026
Tmem70	-0.432764467	0.14048146	2.07E-03	1.17E-02	ENSMUSG00000025940
Tmem8	1.98127534	0.28487458	3.53E-12	2.64E-10	ENSMUSG00000024180
Tmem86a	1.294202838	0.22776759	1.33E-08	4.57E-07	ENSMUSG00000010307
Tmem87b	-0.831695092	0.2159443	1.17E-04	1.14E-03	ENSMUSG00000014353
Tmem98	0.496622116	0.1760854	4.80E-03	2.32E-02	ENSMUSG00000035413
Tmem9b	0.610822149	0.14368714	2.13E-05	2.77E-04	ENSMUSG00000031021
Tmf1	-0.68266918	0.24952816	6.22E-03	2.84E-02	ENSMUSG00000030059
Tmpo	-0.652275654	0.20640023	1.58E-03	9.48E-03	ENSMUSG00000019961
Tmsb4x	0.709950412	0.11745547	1.50E-09	6.39E-08	ENSMUSG00000049775
Tnc	1.962599377	0.39472974	6.63E-07	1.40E-05	ENSMUSG00000028364
Tnfrsf12a	-0.779497866	0.24242389	1.30E-03	8.18E-03	ENSMUSG00000023905
Tnfsf12	-1.170684879	0.3747782	1.79E-03	1.05E-02	ENSMUSG00000097277
Tnks	-0.601000039	0.22467102	7.47E-03	3.28E-02	ENSMUSG00000031529
Tnks2	-0.561326295	0.18609163	2.56E-03	1.40E-02	ENSMUSG00000024811
Tnn	0.710973233	0.19845798	3.40E-04	2.73E-03	ENSMUSG00000026725
Tnpo1	-0.773843523	0.23554271	1.02E-03	6.70E-03	ENSMUSG00000009470
Tnpo3	-0.727091124	0.17306077	2.65E-05	3.34E-04	ENSMUSG00000012535
Tnrc6a	-0.423929531	0.15294329	5.57E-03	2.61E-02	ENSMUSG00000052707
Tnrc6b	-0.848519519	0.29147798	3.60E-03	1.84E-02	ENSMUSG00000047888
Tns1	0.964990126	0.13295989	3.94E-13	3.45E-11	ENSMUSG00000055322
Tns2	0.675157277	0.1716591	8.38E-05	8.70E-04	ENSMUSG00000037003
Tns3	0.489037282	0.13167113	2.04E-04	1.79E-03	ENSMUSG00000020422
Togaram1	-1.451377731	0.25447865	1.17E-08	4.12E-07	ENSMUSG00000035614
Tom1l2	0.641284694	0.14596309	1.12E-05	1.59E-04	ENSMUSG00000000538
Tomm20	1.728883791	0.52273478	9.42E-04	6.32E-03	ENSMUSG00000093424

Tomm7	-0.43826706	0.11445607	1.29E-04	1.23E-03	ENSMUSG00000028998
Tomm70a	-0.662941408	0.16858172	8.41E-05	8.72E-04	ENSMUSG00000022752
Top2b	-0.754275329	0.17968946	2.70E-05	3.39E-04	ENSMUSG00000017485
Topors	-1.249141286	0.26998056	3.71E-06	6.30E-05	ENSMUSG00000036822
Tor1aip1	0.865667801	0.22060253	8.71E-05	8.97E-04	ENSMUSG00000026466
Tpcn1	0.964547636	0.18584729	2.10E-07	5.06E-06	ENSMUSG00000032741
Tpcn2	1.212652581	0.30291524	6.25E-05	6.92E-04	ENSMUSG00000048677
Tpd52	-1.203576355	0.22218484	6.06E-08	1.70E-06	ENSMUSG00000027506
Tpgs1	0.561278633	0.14872095	1.61E-04	1.47E-03	ENSMUSG00000020308
Tpi1	0.619972006	0.10741711	7.85E-09	2.84E-07	ENSMUSG00000023456
Tpm1	0.393578856	0.09895089	6.96E-05	7.52E-04	ENSMUSG00000032366
Tpm4	-0.336270702	0.12580874	7.52E-03	3.30E-02	ENSMUSG00000031799
Tpp2	-0.711822547	0.26964763	8.29E-03	3.55E-02	ENSMUSG00000041763
Tpr	-0.93721217	0.23166592	5.22E-05	5.94E-04	ENSMUSG00000006005
Tpst1	1.05790761	0.16730121	2.56E-10	1.34E-08	ENSMUSG00000034118
Tpst2	0.879634142	0.13396329	5.16E-11	3.23E-09	ENSMUSG00000029344
Tpt1	-0.626522433	0.22316996	4.99E-03	2.39E-02	ENSMUSG00000060126
Tra2a	-0.650988415	0.17597222	2.16E-04	1.88E-03	ENSMUSG00000029817
Tra2b	-1.270420343	0.18640777	9.41E-12	6.62E-10	ENSMUSG00000022858
Trabd2b	1.652948291	0.14076538	7.71E-32	4.83E-29	ENSMUSG00000070867
Tradd	0.442931033	0.16841815	8.54E-03	3.63E-02	ENSMUSG00000031887
Trafd1	0.807142332	0.18863222	1.88E-05	2.50E-04	ENSMUSG00000042726
Tram2	0.870931598	0.19255458	6.10E-06	9.58E-05	ENSMUSG00000041779
Trappc1	0.356442459	0.1387003	1.02E-02	4.17E-02	ENSMUSG00000049299
Trappc3	0.384051299	0.11577233	9.09E-04	6.15E-03	ENSMUSG00000028847
Trappc6a	0.469089681	0.12932192	2.86E-04	2.36E-03	ENSMUSG00000002043
Trappc9	0.975287315	0.25035108	9.79E-05	9.88E-04	ENSMUSG00000047921
Trdc	2.009149064	0.69860863	4.03E-03	2.00E-02	ENSMUSG00000104686
Trerf1	-0.680048636	0.24608643	5.72E-03	2.67E-02	ENSMUSG00000064043
Trim2	-0.420992547	0.15540331	6.75E-03	3.03E-02	ENSMUSG00000027993
Trim24	-1.036767443	0.28374186	2.58E-04	2.18E-03	ENSMUSG00000029833
Trim27	-0.393503001	0.13549006	3.68E-03	1.87E-02	ENSMUSG00000021326
Trim33	-0.874562997	0.32650192	7.39E-03	3.27E-02	ENSMUSG00000033014
Trim37	-0.903521131	0.27448438	9.96E-04	6.58E-03	ENSMUSG00000018548
Trim44	-0.679975183	0.20410866	8.64E-04	5.91E-03	ENSMUSG00000027189
Trim47	0.453024076	0.15094452	2.69E-03	1.46E-02	ENSMUSG00000020773
Trim56	-0.958143763	0.27159865	4.19E-04	3.25E-03	ENSMUSG00000043279
Trim63	-1.745248461	0.27720018	3.05E-10	1.57E-08	ENSMUSG00000028834
Trim65	1.908154668	0.67311373	4.59E-03	2.23E-02	ENSMUSG00000054517
Triobp	0.650875086	0.18955753	5.96E-04	4.32E-03	ENSMUSG00000033088
Trip12	-0.611063129	0.16451598	2.04E-04	1.79E-03	ENSMUSG00000026219
Trir	0.404605786	0.10511813	1.19E-04	1.15E-03	ENSMUSG00000041203
Trmt1	-0.364942199	0.13826648	8.31E-03	3.56E-02	ENSMUSG00000001909

Trmt10a	-1.013577026	0.26671318	1.45E-04	1.35E-03	ENSMUSG00000004127
Trmt10c	-1.551004595	0.41611042	1.93E-04	1.72E-03	ENSMUSG00000044763
Trmt12	1.400473156	0.41832397	8.15E-04	5.62E-03	ENSMUSG00000037085
Trmt13	-1.484686315	0.53290316	5.34E-03	2.52E-02	ENSMUSG00000033439
Trnp1	0.556764538	0.14614396	1.39E-04	1.31E-03	ENSMUSG00000056596
Trnt1	-1.454234722	0.56799267	1.05E-02	4.26E-02	ENSMUSG00000013736
Trp53inp1	-0.797764014	0.1447571	3.57E-08	1.08E-06	ENSMUSG00000028211
Trp53inp2	0.592553593	0.15435642	1.24E-04	1.19E-03	ENSMUSG00000038375
Trpm4	1.213917211	0.3308989	2.44E-04	2.08E-03	ENSMUSG00000038260
Trpv4	0.782675187	0.24482992	1.39E-03	8.58E-03	ENSMUSG00000014158
Tsc22d2	-1.081196353	0.22013393	9.04E-07	1.85E-05	ENSMUSG00000027806
Tsc22d3	-0.318525259	0.122974	9.59E-03	3.99E-02	ENSMUSG00000031431
Tsc22d4	0.691591802	0.18136812	1.37E-04	1.30E-03	ENSMUSG00000029723
Tsen34	0.793061653	0.1624607	1.05E-06	2.12E-05	ENSMUSG00000035585
Tspan11	1.231628468	0.28932249	2.07E-05	2.71E-04	ENSMUSG00000030351
Tspan13	0.680890506	0.21572624	1.60E-03	9.59E-03	ENSMUSG00000020577
Tspan14	0.6460647	0.20043749	1.27E-03	8.01E-03	ENSMUSG00000037824
Tspan17	1.189939039	0.1540449	1.12E-14	1.15E-12	ENSMUSG00000025875
Tspan18	1.343240046	0.30292194	9.24E-06	1.35E-04	ENSMUSG00000027217
Tspan3	0.369151016	0.12583144	3.35E-03	1.73E-02	ENSMUSG00000032324
Tspan4	0.65923779	0.11134852	3.21E-09	1.27E-07	ENSMUSG00000025511
Tspan6	0.487979514	0.1451926	7.77E-04	5.42E-03	ENSMUSG00000067377
Tspan9	0.518225902	0.15087022	5.93E-04	4.31E-03	ENSMUSG00000030352
Tsr1	-0.846589422	0.18846089	7.05E-06	1.08E-04	ENSMUSG00000038335
Ttc1	-0.378701366	0.14191629	7.62E-03	3.33E-02	ENSMUSG00000041278
Ttc17	-0.726919587	0.24521815	3.03E-03	1.60E-02	ENSMUSG00000027194
Ttc28	0.476987289	0.18637739	1.05E-02	4.27E-02	ENSMUSG00000033209
Ttc3	-1.056734838	0.21078319	5.35E-07	1.16E-05	ENSMUSG00000040785
Ttc30b	2.096568238	0.71862519	3.53E-03	1.81E-02	ENSMUSG00000075273
Ttc38	0.884754149	0.35337988	1.23E-02	4.82E-02	ENSMUSG00000035944
Ttl	-0.563415745	0.20707755	6.51E-03	2.95E-02	ENSMUSG00000027394
Ttyh2	1.264831917	0.34564581	2.53E-04	2.13E-03	ENSMUSG00000034714
Ttyh3	0.985103244	0.24926604	7.75E-05	8.19E-04	ENSMUSG00000036565
Tuba1a	1.057672995	0.12334719	9.93E-18	1.44E-15	ENSMUSG00000072235
Tuba1b	0.867073214	0.14066668	7.09E-10	3.35E-08	ENSMUSG00000023004
Tuba1c	1.193032065	0.15857559	5.34E-14	5.05E-12	ENSMUSG00000043091
Tuba4a	0.353711346	0.13051208	6.72E-03	3.03E-02	ENSMUSG00000026202
Tubb2a	0.449716135	0.12426504	2.96E-04	2.43E-03	ENSMUSG00000058672
Tubb4b	0.656515987	0.15699147	2.89E-05	3.59E-04	ENSMUSG00000036752
Tubb5	0.277066903	0.11041884	1.21E-02	4.77E-02	ENSMUSG00000001525
Tubb6	0.946535667	0.12996971	3.27E-13	2.89E-11	ENSMUSG00000001473
Tubg2	1.025273518	0.28782737	3.68E-04	2.91E-03	ENSMUSG00000045007
Tubgcp4	-0.558746947	0.20581093	6.63E-03	2.99E-02	ENSMUSG00000027263

Tuft1	-1.168951185	0.3847345	2.38E-03	1.32E-02	ENSMUSG00000005968
Tulp3	0.384057177	0.14366099	7.51E-03	3.30E-02	ENSMUSG00000001521
Tusc3	0.501089954	0.17113076	3.41E-03	1.76E-02	ENSMUSG00000039530
Tut7	-0.836808482	0.20784111	5.67E-05	6.36E-04	ENSMUSG00000035248
Tvp23b	-0.551298838	0.16069146	6.02E-04	4.36E-03	ENSMUSG00000014177
Twf2	0.636613587	0.19750746	1.27E-03	8.01E-03	ENSMUSG00000023277
Twist2	0.797029506	0.25239609	1.59E-03	9.54E-03	ENSMUSG00000007805
Twistnb	-1.037940144	0.26146363	7.20E-05	7.73E-04	ENSMUSG00000020561
Txn1	-0.540767421	0.10442783	2.24E-07	5.36E-06	ENSMUSG00000028367
Txndc12	-0.734930631	0.11328288	8.72E-11	5.08E-09	ENSMUSG00000028567
Txndc15	0.577019826	0.16149371	3.53E-04	2.81E-03	ENSMUSG00000021497
Txndc5	0.3828893	0.11691155	1.06E-03	6.90E-03	ENSMUSG00000038991
Txndc9	-0.328627427	0.12926113	1.10E-02	4.44E-02	ENSMUSG00000058407
Txnip	-0.758775242	0.13156069	8.05E-09	2.90E-07	ENSMUSG00000038393
Txnl1	-0.446802445	0.13685527	1.10E-03	7.12E-03	ENSMUSG00000024583
Txnrd1	-0.936099889	0.12716173	1.82E-13	1.65E-11	ENSMUSG00000020250
Txnrd2	0.712730952	0.26426619	7.00E-03	3.13E-02	ENSMUSG00000075704
Tyk2	0.664908832	0.23833742	5.27E-03	2.50E-02	ENSMUSG00000032175
U2af2	0.458682597	0.13768441	8.64E-04	5.91E-03	ENSMUSG00000030435
U2surp	-1.210810692	0.27625051	1.17E-05	1.66E-04	ENSMUSG00000032407
Uap1l1	0.495357384	0.18353521	6.96E-03	3.12E-02	ENSMUSG00000026956
Uba3	-1.242590682	0.26051387	1.84E-06	3.47E-05	ENSMUSG00000030061
Uba5	-0.958991878	0.1777382	6.83E-08	1.91E-06	ENSMUSG00000032557
Uba6	-0.812421543	0.28458965	4.31E-03	2.12E-02	ENSMUSG00000035898
Ube2b	-0.350382284	0.11965839	3.41E-03	1.76E-02	ENSMUSG00000020390
Ube2d3	-0.699893164	0.12321516	1.34E-08	4.61E-07	ENSMUSG00000078578
Ube2f	-0.407363156	0.13832115	3.23E-03	1.68E-02	ENSMUSG00000034343
Ube2m	0.421481942	0.13913121	2.45E-03	1.35E-02	ENSMUSG00000005575
Ube2ql1	-1.03051561	0.34493118	2.81E-03	1.50E-02	ENSMUSG00000052981
Ube2w	-0.516319701	0.18219204	4.60E-03	2.24E-02	ENSMUSG00000025939
Ube3a	-0.705462331	0.2058144	6.09E-04	4.40E-03	ENSMUSG00000025326
Ube3b	0.635434427	0.15363052	3.53E-05	4.25E-04	ENSMUSG00000029577
Ube4b	-0.528660795	0.13323702	7.25E-05	7.78E-04	ENSMUSG00000028960
Ubqln1	-0.441994717	0.12751992	5.28E-04	3.92E-03	ENSMUSG00000005312
Ubr2	-0.660601857	0.16550541	6.57E-05	7.17E-04	ENSMUSG00000023977
Ubr5	-0.715287704	0.21426014	8.43E-04	5.78E-03	ENSMUSG00000037487
Ubtd1	-0.462793211	0.18458715	1.22E-02	4.79E-02	ENSMUSG00000025171
Ubtf	0.826961754	0.21929408	1.63E-04	1.48E-03	ENSMUSG00000020923
Ubxn2a	-1.052473786	0.39455709	7.64E-03	3.34E-02	ENSMUSG00000020634
Ubxn4	-0.787596796	0.16171513	1.11E-06	2.22E-05	ENSMUSG00000026353
Ubxn7	-0.790781502	0.2689638	3.28E-03	1.70E-02	ENSMUSG00000053774
Ubxn8	0.99924687	0.25796758	1.07E-04	1.06E-03	ENSMUSG00000052906
Uchl5	-1.148942193	0.28502847	5.55E-05	6.26E-04	ENSMUSG00000018189

Uck2	-0.408327601	0.12776734	1.39E-03	8.60E-03	ENSMUSG00000026558
Ucma	0.452305428	0.15917719	4.49E-03	2.19E-02	ENSMUSG00000026668
Ucp2	1.434257675	0.11108298	3.87E-38	4.25E-35	ENSMUSG00000033685
Ugcg	-0.990710989	0.24866927	6.78E-05	7.36E-04	ENSMUSG00000028381
Uhmk1	-0.642181896	0.20705689	1.93E-03	1.11E-02	ENSMUSG00000026667
Uhrf1	1.483949151	0.56774917	8.96E-03	3.78E-02	ENSMUSG00000001228
Uhrf1bp1l	-1.184383038	0.28952227	4.30E-05	5.05E-04	ENSMUSG00000019951
Uhrf2	-0.710730395	0.14936531	1.95E-06	3.64E-05	ENSMUSG00000024817
Uimc1	-0.861211089	0.22100005	9.74E-05	9.84E-04	ENSMUSG00000025878
Unc13a	3.317016271	1.00158611	9.27E-04	6.26E-03	ENSMUSG00000034799
Unc45a	0.445338227	0.1765226	1.16E-02	4.63E-02	ENSMUSG00000030533
Unc93b1	0.568610372	0.16101202	4.13E-04	3.21E-03	ENSMUSG00000036908
Upf2	-1.058274175	0.28026926	1.59E-04	1.46E-03	ENSMUSG00000043241
Upf3b	-1.202625044	0.31824671	1.58E-04	1.45E-03	ENSMUSG00000036572
Uqcr11	0.43519116	0.11218645	1.05E-04	1.04E-03	ENSMUSG00000020163
Uqcrc1	0.390708702	0.12494284	1.77E-03	1.04E-02	ENSMUSG00000025651
Uri1	-0.874156761	0.21071292	3.35E-05	4.08E-04	ENSMUSG00000030421
Urm1	0.39340552	0.14035518	5.06E-03	2.42E-02	ENSMUSG00000069020
Urod	0.392336084	0.12403968	1.56E-03	9.40E-03	ENSMUSG00000028684
Usf3	-0.982917944	0.36526647	7.12E-03	3.17E-02	ENSMUSG00000068284
Uso1	-0.894877897	0.25313357	4.07E-04	3.17E-03	ENSMUSG00000029407
Usp14	-0.57951216	0.17999646	1.28E-03	8.09E-03	ENSMUSG00000047879
Usp15	-1.214221553	0.37301957	1.13E-03	7.34E-03	ENSMUSG00000020124
Usp16	-0.986468819	0.23692789	3.13E-05	3.85E-04	ENSMUSG00000025616
Usp2	0.481568627	0.18556287	9.45E-03	3.94E-02	ENSMUSG00000032010
Usp20	0.577902141	0.21798916	8.02E-03	3.47E-02	ENSMUSG00000026854
Usp25	-0.735061367	0.25807611	4.40E-03	2.16E-02	ENSMUSG00000022867
Usp3	-0.644462087	0.17459847	2.23E-04	1.93E-03	ENSMUSG00000032376
Usp33	-1.040379433	0.36139367	3.99E-03	1.99E-02	ENSMUSG00000025437
Usp34	-0.774813975	0.24467309	1.54E-03	9.33E-03	ENSMUSG00000056342
Usp39	-0.432961961	0.13930589	1.88E-03	1.09E-02	ENSMUSG00000056305
Usp4	0.492173508	0.17549722	5.04E-03	2.41E-02	ENSMUSG00000032612
Usp47	-0.955562648	0.28867735	9.32E-04	6.28E-03	ENSMUSG00000059263
Usp48	-0.526477156	0.18265584	3.95E-03	1.98E-02	ENSMUSG00000043411
Usp7	-0.652309828	0.19738645	9.51E-04	6.36E-03	ENSMUSG00000022710
Usp8	-0.655414804	0.24535762	7.56E-03	3.31E-02	ENSMUSG00000027363
Utp11	-0.675615882	0.11905167	1.39E-08	4.74E-07	ENSMUSG00000028907
Utp14a	-1.030107404	0.37206818	5.63E-03	2.63E-02	ENSMUSG00000063785
Utp18	-0.78504631	0.26813014	3.41E-03	1.76E-02	ENSMUSG00000054079
Utp3	-0.50300396	0.1625562	1.97E-03	1.13E-02	ENSMUSG00000070697
Uvssa	-1.127456327	0.3598225	1.73E-03	1.02E-02	ENSMUSG00000037355
Vamp2	0.625114339	0.18694569	8.26E-04	5.68E-03	ENSMUSG00000020894
Vamp4	-0.640660472	0.16948379	1.57E-04	1.44E-03	ENSMUSG00000026696

Vamp8	0.521835959	0.11491774	5.60E-06	8.88E-05	ENSMUSG00000050732
Vapa	-0.522748778	0.1147035	5.18E-06	8.30E-05	ENSMUSG00000024091
Vasn	1.071115505	0.12123592	1.00E-18	1.67E-16	ENSMUSG00000039646
Vasp	0.477655818	0.14353334	8.75E-04	5.96E-03	ENSMUSG00000030403
Vav2	0.972203012	0.34608283	4.97E-03	2.39E-02	ENSMUSG00000009621
Vbp1	-0.913017384	0.25045703	2.67E-04	2.23E-03	ENSMUSG00000031197
Vcam1	0.782069656	0.23106831	7.13E-04	5.03E-03	ENSMUSG00000027962
Vdac3	-0.46495852	0.16951628	6.09E-03	2.79E-02	ENSMUSG00000008892
Vdr	1.873657483	0.20916352	3.31E-19	5.81E-17	ENSMUSG00000022479
Vegfd	0.656019803	0.21350948	2.12E-03	1.20E-02	ENSMUSG00000031380
Vezf1	-0.600323459	0.21350855	4.93E-03	2.37E-02	ENSMUSG00000018377
Vit	1.589543686	0.26689572	2.59E-09	1.04E-07	ENSMUSG00000024076
Vkorc1	-1.764910171	0.28607139	6.85E-10	3.26E-08	ENSMUSG00000096001
Vmac	0.734933759	0.27824767	8.26E-03	3.54E-02	ENSMUSG00000054723
Vmp1	-0.48750489	0.12855003	1.49E-04	1.39E-03	ENSMUSG00000018171
Vps13a	-1.765994433	0.38648534	4.89E-06	7.94E-05	ENSMUSG00000046230
Vps13d	-0.753758408	0.28162067	7.44E-03	3.28E-02	ENSMUSG00000020220
Vps26a	-0.66342672	0.20131806	9.83E-04	6.52E-03	ENSMUSG00000020078
Vps28	-0.949077086	0.31437397	2.54E-03	1.39E-02	ENSMUSG00000115604
Vps35	-1.086082171	0.19174538	1.48E-08	5.00E-07	ENSMUSG00000031696
Vps35l	0.898002768	0.21242071	2.36E-05	3.02E-04	ENSMUSG00000030982
Vps37a	-0.803843531	0.17153099	2.78E-06	4.94E-05	ENSMUSG00000031600
Vps37b	-0.576855567	0.21686893	7.82E-03	3.40E-02	ENSMUSG00000066278
Vps4b	-0.539870454	0.18467589	3.46E-03	1.78E-02	ENSMUSG00000009907
Vps50	-0.877693934	0.34367807	1.07E-02	4.33E-02	ENSMUSG00000001376
Vps52	0.941585538	0.18878134	6.11E-07	1.31E-05	ENSMUSG00000024319
Vps53	0.620697092	0.1909273	1.15E-03	7.43E-03	ENSMUSG00000017288
Vps9d1	0.762267714	0.28530542	7.55E-03	3.31E-02	ENSMUSG00000001062
Vstm4	1.397244577	0.38434742	2.78E-04	2.31E-03	ENSMUSG00000050666
Wac	-1.009467626	0.28833152	4.63E-04	3.53E-03	ENSMUSG00000024283
Wapl	-0.752544205	0.29100076	9.71E-03	4.03E-02	ENSMUSG00000041408
Washc2	-0.685396561	0.22805803	2.65E-03	1.44E-02	ENSMUSG00000024104
Washc3	0.483429762	0.12897604	1.78E-04	1.60E-03	ENSMUSG00000020056
Washc4	-1.227111626	0.28822032	2.07E-05	2.71E-04	ENSMUSG00000034560
Wasl	-0.782304183	0.23596517	9.15E-04	6.19E-03	ENSMUSG00000029684
Wbp1	0.459777531	0.14366191	1.37E-03	8.51E-03	ENSMUSG00000030035
Wbp1l	0.544552911	0.15570785	4.70E-04	3.57E-03	ENSMUSG00000047731
Wbp4	-0.61356195	0.2375749	9.81E-03	4.05E-02	ENSMUSG00000022023
Wdr27	1.068225562	0.27748827	1.18E-04	1.15E-03	ENSMUSG00000046991
Wdr3	-0.612241678	0.22199553	5.82E-03	2.70E-02	ENSMUSG00000033285
Wdr4	-0.618642653	0.22842061	6.76E-03	3.04E-02	ENSMUSG00000024037
Wdr43	-0.938907989	0.16382838	9.98E-09	3.55E-07	ENSMUSG00000041057
Wdr6	0.966659901	0.15053111	1.35E-10	7.46E-09	ENSMUSG00000066357

Wdr60	-1.596426539	0.33065543	1.38E-06	2.67E-05	ENSMUSG00000042050
Wdr75	-1.075180361	0.28609298	1.71E-04	1.55E-03	ENSMUSG00000025995
Wdr81	0.862280254	0.25005282	5.64E-04	4.14E-03	ENSMUSG00000045374
Wdr89	-0.467834734	0.17753798	8.41E-03	3.59E-02	ENSMUSG00000045690
Wfs1	0.711385102	0.2701923	8.47E-03	3.61E-02	ENSMUSG00000039474
Wif1	1.305876588	0.16582195	3.40E-15	3.73E-13	ENSMUSG00000020218
Wnk1	-0.749434596	0.17561902	1.98E-05	2.62E-04	ENSMUSG00000045962
Wnk4	1.07480165	0.29635126	2.87E-04	2.37E-03	ENSMUSG00000035112
Wnt4	0.930586284	0.20678422	6.79E-06	1.05E-04	ENSMUSG00000036856
Wnt5a	0.636756776	0.25121331	1.13E-02	4.50E-02	ENSMUSG00000021994
Wnt6	2.44014649	0.67400741	2.94E-04	2.42E-03	ENSMUSG00000033227
Wrap53	0.880828469	0.29369371	2.71E-03	1.46E-02	ENSMUSG00000041346
Wwtr1	-0.487513691	0.12174229	6.22E-05	6.89E-04	ENSMUSG00000027803
Xpo1	-0.969760215	0.17673066	4.08E-08	1.21E-06	ENSMUSG00000020290
Xpo7	-0.516272849	0.15866509	1.14E-03	7.37E-03	ENSMUSG00000022100
Xrn2	-0.95245432	0.18743469	3.74E-07	8.43E-06	ENSMUSG00000027433
Xylt2	0.879578631	0.28524576	2.05E-03	1.17E-02	ENSMUSG00000020868
Yars	-0.408402615	0.13304466	2.14E-03	1.21E-02	ENSMUSG00000028811
Ybx1	-0.358149049	0.12049166	2.95E-03	1.57E-02	ENSMUSG00000028639
Ybx3	-0.506026993	0.10320765	9.44E-07	1.93E-05	ENSMUSG00000030189
Yipf1	0.411786332	0.14402047	4.25E-03	2.10E-02	ENSMUSG00000057375
Yipf2	0.869504153	0.23745373	2.50E-04	2.12E-03	ENSMUSG00000032182
Yipf5	-0.614822582	0.11260027	4.76E-08	1.38E-06	ENSMUSG00000024487
Yme1l1	-1.056712611	0.25081122	2.52E-05	3.19E-04	ENSMUSG00000026775
Ypel5	-0.365033234	0.11750884	1.89E-03	1.10E-02	ENSMUSG00000039770
Ythdc1	-0.889747435	0.24026778	2.13E-04	1.85E-03	ENSMUSG00000035851
Ythdf3	-0.662275052	0.19953115	9.03E-04	6.12E-03	ENSMUSG00000047213
Ywhae	-0.515565777	0.10048233	2.88E-07	6.71E-06	ENSMUSG00000020849
Zadh2	-0.700612945	0.19754299	3.90E-04	3.06E-03	ENSMUSG00000049090
Zbtb11	-1.524495508	0.28017571	5.29E-08	1.52E-06	ENSMUSG00000022601
Zbtb11os1	-1.090116063	0.41725835	8.99E-03	3.78E-02	ENSMUSG00000101609
Zbtb20	-0.537273247	0.19790269	6.63E-03	2.99E-02	ENSMUSG00000022708
Zbtb22	0.692538522	0.2719888	1.09E-02	4.40E-02	ENSMUSG00000051390
Zbtb38	-0.673109486	0.22448152	2.71E-03	1.46E-02	ENSMUSG00000040433
Zbtb44	-0.675227711	0.23976284	4.86E-03	2.34E-02	ENSMUSG00000047412
Zbtb8os	0.677397155	0.21849282	1.93E-03	1.11E-02	ENSMUSG00000057572
Zc2hc1a	-0.639144963	0.22356436	4.25E-03	2.10E-02	ENSMUSG00000043542
Zc3h13	-0.751283475	0.23101434	1.15E-03	7.41E-03	ENSMUSG00000022000
Zc3h15	-1.092627502	0.21099356	2.24E-07	5.36E-06	ENSMUSG00000027091
Zc3h6	-0.984224139	0.36107354	6.41E-03	2.91E-02	ENSMUSG00000042851
Zcchc17	0.334199285	0.13370882	1.24E-02	4.87E-02	ENSMUSG00000028772
Zcchc24	0.420769344	0.12509255	7.69E-04	5.38E-03	ENSMUSG00000055538
Zcchc7	-0.863157932	0.33242311	9.42E-03	3.93E-02	ENSMUSG00000035649

Zcchc9	-0.752193471	0.23450647	1.34E-03	8.34E-03	ENSMUSG00000021621
Zcrb1	-0.551729108	0.11700535	2.41E-06	4.36E-05	ENSMUSG00000022635
Zdhhc12	0.557532906	0.16748557	8.72E-04	5.95E-03	ENSMUSG00000015335
Zdhhc20	-0.885920332	0.20450292	1.48E-05	2.03E-04	ENSMUSG00000021969
Zdhhc3	0.487233363	0.17533864	5.46E-03	2.56E-02	ENSMUSG00000025786
Zdhhc5	0.505125809	0.17621106	4.15E-03	2.06E-02	ENSMUSG00000034075
Zeb1	-0.814879775	0.19478189	2.87E-05	3.57E-04	ENSMUSG00000024238
Zer1	0.663582575	0.22361748	3.00E-03	1.59E-02	ENSMUSG00000039686
Zfand5	-0.910975969	0.17308994	1.42E-07	3.59E-06	ENSMUSG00000024750
Zfand6	-0.528619927	0.14981467	4.18E-04	3.24E-03	ENSMUSG00000030629
Zfas1	-1.548531619	0.12687742	2.93E-34	2.14E-31	ENSMUSG00000074578
Zfc3h1	-0.860413163	0.27578256	1.81E-03	1.06E-02	ENSMUSG00000034163
Zfp106	-0.576713403	0.19601795	3.26E-03	1.70E-02	ENSMUSG00000027288
Zfp110	-0.879588752	0.25840375	6.64E-04	4.73E-03	ENSMUSG00000058638
Zfp120	-0.989242055	0.32556643	2.38E-03	1.32E-02	ENSMUSG00000068134
Zfp131	-0.666037298	0.18362676	2.87E-04	2.36E-03	ENSMUSG00000094483
Zfp148	-0.722898191	0.26103044	5.62E-03	2.63E-02	ENSMUSG00000022811
Zfp160	-0.973864253	0.36708942	7.98E-03	3.45E-02	ENSMUSG00000067942
Zfp260	-0.83439728	0.2100942	7.14E-05	7.69E-04	ENSMUSG00000049421
Zfp266	-0.699439504	0.27058158	9.74E-03	4.03E-02	ENSMUSG00000060510
Zfp292	-0.803288393	0.24328033	9.60E-04	6.40E-03	ENSMUSG00000039967
Zfp317	-1.000809206	0.36166492	5.65E-03	2.64E-02	ENSMUSG00000057551
Zfp324	1.105108047	0.4111291	7.19E-03	3.19E-02	ENSMUSG00000004500
Zfp326	-0.760178114	0.2770944	6.08E-03	2.79E-02	ENSMUSG00000029290
Zfp330	-1.102773502	0.19648338	1.99E-08	6.51E-07	ENSMUSG00000031711
Zfp354a	-2.497161219	0.97163467	1.02E-02	4.17E-02	ENSMUSG00000020364
Zfp354c	-0.949626573	0.28619393	9.06E-04	6.14E-03	ENSMUSG00000044807
Zfp358	0.629982308	0.19775973	1.44E-03	8.87E-03	ENSMUSG00000047264
Zfp362	0.691629909	0.20894144	9.32E-04	6.28E-03	ENSMUSG00000028799
Zfp367	-1.530598334	0.48806908	1.71E-03	1.01E-02	ENSMUSG00000044934
Zfp386	-0.863080568	0.27745961	1.87E-03	1.09E-02	ENSMUSG00000042063
Zfp395	1.511341753	0.24803805	1.11E-09	4.86E-08	ENSMUSG00000034522
Zfp397	-1.73207955	0.36677343	2.33E-06	4.24E-05	ENSMUSG00000024276
Zfp414	1.177107236	0.25982848	5.89E-06	9.28E-05	ENSMUSG00000073423
Zfp420	-1.459499933	0.55656235	8.73E-03	3.70E-02	ENSMUSG00000058402
Zfp445	-1.453409938	0.34110718	2.04E-05	2.69E-04	ENSMUSG00000047036
Zfp451	-0.698786936	0.25835911	6.84E-03	3.07E-02	ENSMUSG00000042197
Zfp467	0.730082528	0.20489926	3.66E-04	2.90E-03	ENSMUSG00000068551
Zfp503	0.574922537	0.17281992	8.79E-04	5.98E-03	ENSMUSG00000039081
Zfp51	-1.298803158	0.38695231	7.89E-04	5.49E-03	ENSMUSG00000023892
Zfp511	-0.574949543	0.18702069	2.11E-03	1.20E-02	ENSMUSG00000025470
Zfp512b	0.932506147	0.2701167	5.56E-04	4.09E-03	ENSMUSG00000000823
Zfp580	1.112987204	0.2252111	7.73E-07	1.62E-05	ENSMUSG00000055633

Zfp607a	-1.650425259	0.64684834	1.07E-02	4.35E-02	ENSMUSG00000020420
Zfp62	-1.330032269	0.51045	9.17E-03	3.85E-02	ENSMUSG00000046311
Zfp68	-1.536325777	0.35052572	1.17E-05	1.66E-04	ENSMUSG00000058291
Zfp703	1.236142955	0.13602723	1.01E-19	1.91E-17	ENSMUSG00000085795
Zfp715	-0.927258566	0.34792797	7.70E-03	3.36E-02	ENSMUSG00000012640
Zfp758	-1.754131647	0.69796365	1.20E-02	4.73E-02	ENSMUSG00000044501
Zfp771	0.456945837	0.16579738	5.85E-03	2.71E-02	ENSMUSG00000054716
Zfp780b	-1.219767215	0.48896598	1.26E-02	4.91E-02	ENSMUSG00000063047
Zfp800	-1.049409608	0.35301074	2.95E-03	1.57E-02	ENSMUSG00000039841
Zfp809	-0.792751619	0.28438843	5.31E-03	2.51E-02	ENSMUSG00000057982
Zfp81	-1.566541073	0.51213444	2.22E-03	1.25E-02	ENSMUSG00000003929
Zfp850	1.349787701	0.42270773	1.41E-03	8.66E-03	ENSMUSG00000096727
Zfp874a	-1.904555249	0.55496283	5.99E-04	4.35E-03	ENSMUSG00000069206
Zfp9	-1.211309243	0.30664017	7.81E-05	8.24E-04	ENSMUSG00000072623
Zfp91	-0.423905494	0.15729663	7.04E-03	3.14E-02	ENSMUSG00000024695
Zfp930	-1.722481432	0.45562511	1.57E-04	1.44E-03	ENSMUSG00000059897
Zfp958	-2.082625515	0.31839124	6.11E-11	3.70E-09	ENSMUSG00000058748
Zfr	-0.66187345	0.15224813	1.38E-05	1.92E-04	ENSMUSG00000022201
Zhx3	0.926206905	0.19771009	2.80E-06	4.96E-05	ENSMUSG00000035877
Zmat3	-0.368175764	0.13474378	6.29E-03	2.87E-02	ENSMUSG00000027663
Zmat5	0.501768485	0.14504681	5.41E-04	4.01E-03	ENSMUSG00000009076
Zmym2	-0.75538299	0.2075347	2.73E-04	2.28E-03	ENSMUSG00000021945
Zmym5	-0.940081639	0.30590265	2.12E-03	1.20E-02	ENSMUSG00000040123
Zmynd11	-0.595326298	0.1935806	2.10E-03	1.19E-02	ENSMUSG00000021156
Zmynd19	-0.692966764	0.22716885	2.29E-03	1.27E-02	ENSMUSG00000026974
Zmynd8	0.570680787	0.21225004	7.17E-03	3.19E-02	ENSMUSG00000039671
Znhit6	-0.528317353	0.20916781	1.15E-02	4.59E-02	ENSMUSG00000074182
Znrf3	1.143346022	0.43414885	8.45E-03	3.60E-02	ENSMUSG00000041961
Zpr1	-0.465252933	0.15005839	1.93E-03	1.11E-02	ENSMUSG00000032078
Zranb1	-0.744512115	0.19618008	1.48E-04	1.37E-03	ENSMUSG00000030967
Zranb2	-0.88701585	0.20815608	2.03E-05	2.68E-04	ENSMUSG00000028180
Zrsr1	-0.857783172	0.23208835	2.19E-04	1.90E-03	ENSMUSG00000044068
Zrsr2	-0.728147114	0.16022442	5.51E-06	8.75E-05	ENSMUSG00000031370
Zswim3	1.124316363	0.40538461	5.55E-03	2.60E-02	ENSMUSG00000045822
Zswim6	-0.542062846	0.20239075	7.40E-03	3.27E-02	ENSMUSG00000032846
Zswim8	0.572131696	0.18453749	1.93E-03	1.11E-02	ENSMUSG00000021819
Zwint	-0.445651749	0.13532832	9.91E-04	6.55E-03	ENSMUSG00000019923
Zzz3	-1.326163174	0.34885384	1.44E-04	1.35E-03	ENSMUSG00000039068
0610040B10Rik	1.257069655	0.34572358	2.77E-04	2.31E-03	ENSMUSG00000089889
1110019D14Rik	-0.768306799	0.30537762	1.19E-02	4.70E-02	ENSMUSG00000097415
1110032A03Rik	0.569671794	0.21516976	8.11E-03	3.49E-02	ENSMUSG00000037971
1110038B12Rik	-0.669168641	0.12831873	1.84E-07	4.50E-06	ENSMUSG00000092203
1110051M20Rik	1.08692665	0.17614852	6.81E-10	3.26E-08	ENSMUSG00000040591

1500015O10Rik	0.427728136	0.13791955	1.93E-03	1.11E-02	ENSMUSG00000026051
1600014C10Rik	0.885000957	0.21709291	4.57E-05	5.31E-04	ENSMUSG00000054676
1700003E16Rik	1.668727627	0.66420055	1.20E-02	4.74E-02	ENSMUSG00000030030
1700021F05Rik	-0.793286258	0.19319281	4.02E-05	4.77E-04	ENSMUSG00000019797
1700086O06Rik	3.672936437	0.98339781	1.88E-04	1.68E-03	ENSMUSG00000097059
1700088E04Rik	0.848278141	0.33519914	1.14E-02	4.54E-02	ENSMUSG00000033029
1700124L16Rik	-0.765228317	0.16037082	1.83E-06	3.45E-05	ENSMUSG00000098492
1810010H24Rik	3.477534803	0.85532783	4.79E-05	5.53E-04	ENSMUSG00000078607
1810021B22Rik	1.165000132	0.25593521	5.32E-06	8.49E-05	ENSMUSG00000087331
1810022K09Rik	-0.574972489	0.14371899	6.32E-05	6.98E-04	ENSMUSG00000078784
1810024B03Rik	1.231649199	0.19817726	5.14E-10	2.52E-08	ENSMUSG00000044145
1810032O08Rik	-1.822808706	0.27015477	1.51E-11	1.03E-09	ENSMUSG00000020812
2010001A14Rik	0.732209766	0.23636277	1.95E-03	1.12E-02	ENSMUSG00000087165
2310009A05Rik	-1.053979287	0.29623487	3.74E-04	2.95E-03	ENSMUSG00000098188
2310015A10Rik	-2.00806139	0.65696791	2.24E-03	1.25E-02	ENSMUSG00000097637
2310022B05Rik	0.672504093	0.10754359	4.02E-10	2.03E-08	ENSMUSG00000031983
2310033P09Rik	0.738826365	0.2421379	2.28E-03	1.27E-02	ENSMUSG00000020441
2410006H16Rik	-1.08015586	0.13652028	2.53E-15	2.82E-13	ENSMUSG00000086841
2410022M11Rik	1.298329863	0.39712543	1.08E-03	7.03E-03	ENSMUSG00000099966
2500002B13Rik	-5.685215683	1.06200192	8.64E-08	2.32E-06	ENSMUSG00000096916
2510039O18Rik	0.526779873	0.13374759	8.20E-05	8.55E-04	ENSMUSG00000044496
2610008E11Rik	-1.114686431	0.33272382	8.08E-04	5.59E-03	ENSMUSG00000060301
2610301B20Rik	-0.971430833	0.23134672	2.68E-05	3.37E-04	ENSMUSG00000059482
2610528A11Rik	-0.543328756	0.14167729	1.26E-04	1.20E-03	ENSMUSG00000095567
2700038G22Rik	1.039899703	0.40442101	1.01E-02	4.16E-02	ENSMUSG00000097113
2810001G20Rik	1.069454588	0.23263423	4.28E-06	7.11E-05	ENSMUSG00000087497
2810006K23Rik	0.782523605	0.28155959	5.45E-03	2.56E-02	ENSMUSG00000047635
2810402E24Rik	-2.28696825	0.89709645	1.08E-02	4.37E-02	ENSMUSG00000099354
2900076A07Rik	-1.450523426	0.16230266	3.99E-19	6.83E-17	ENSMUSG00000097195
3110070M22Rik	1.872534412	0.4667877	6.03E-05	6.71E-04	ENSMUSG00000074635
4921524J17Rik	-0.746498807	0.25563091	3.50E-03	1.80E-02	ENSMUSG00000036934
4931428F04Rik	1.032900424	0.3053712	7.18E-04	5.06E-03	ENSMUSG00000014837
4932438A13Rik	-0.81522675	0.30284184	7.10E-03	3.16E-02	ENSMUSG00000037270
4933404O12Rik	0.683762672	0.2310251	3.08E-03	1.62E-02	ENSMUSG00000097867
4933417D19Rik	1.813321427	0.38666026	2.74E-06	4.87E-05	ENSMUSG00000097616
5430416N02Rik	2.437539046	0.56072497	1.38E-05	1.92E-04	ENSMUSG00000097767
5730480H06Rik	0.707074956	0.28311148	1.25E-02	4.89E-02	ENSMUSG00000029089
5830448L01Rik	-0.895301067	0.28527116	1.70E-03	1.01E-02	ENSMUSG00000114194
6330562C20Rik	-0.750437729	0.16478572	5.26E-06	8.43E-05	ENSMUSG00000093314
6430548M08Rik	1.222884035	0.41328514	3.09E-03	1.62E-02	ENSMUSG00000031824
6720427I07Rik	0.875196686	0.21387677	4.28E-05	5.03E-04	ENSMUSG00000097328
6820431F20Rik	-2.265425062	0.72902943	1.89E-03	1.09E-02	ENSMUSG00000071796
9130024F11Rik	1.939121566	0.61128481	1.51E-03	9.19E-03	ENSMUSG00000087022

9530068E07Rik	0.532159662	0.12259031	1.42E-05	1.97E-04	ENSMUSG00000036275
9930012K11Rik	2.142224523	0.70620348	2.42E-03	1.34E-02	ENSMUSG00000044551
9930021J03Rik	-1.027872346	0.26778694	1.24E-04	1.19E-03	ENSMUSG00000046138
